# Automated evaluation for rapid implementation of knowledge‐based radiotherapy planning models

**DOI:** 10.1002/acm2.14152

**Published:** 2023-09-13

**Authors:** Joseph Harms, Joel A. Pogue, Carlos E. Cardenas, Dennis N. Stanley, Rex Cardan, Richard Popple

**Affiliations:** ^1^ Department of Radiation Oncology University of Alabama at Birmingham Birmingham USA

**Keywords:** automation, autoplanning, clinical scripting, knowledge‐based planning, VMAT

## Abstract

**Purpose:**

Knowledge‐based planning (KBP) offers the ability to predict dose‐volume metrics based on information extracted from previous plans, reducing plan variability and improving plan quality. As clinical integration of KBP is increasing there is a growing need for quantitative evaluation of KBP models. A .NET‐based application, RapidCompare, was created for automated plan creation and analysis of Varian RapidPlan models.

**Methods:**

RapidCompare was designed to read calculation parameters and a list of reference plans. The tool copies the reference plan field geometry and structure set, applies the RapidPlan model, optimizes the KBP plan, and generates data for quantitative evaluation of dose‐volume metrics. A cohort of 85 patients, divided into training (50), testing (10), and validation (25) groups, was used to demonstrate the utility of RapidCompare. After training and tuning, the KBP model was paired with three different optimization templates to compare various planning strategies in the validation cohort. All templates used the same set of constraints for the planning target volume (PTV). For organs‐at‐risk, the optimization template provided constraints using the whole dose‐volume histogram (DVH), fixed‐dose/volume points, or generalized equivalent uniform dose (gEUD). The resulting plans from each optimization approach were compared using DVH metrics.

**Results:**

RapidCompare allowed for the automated generation of 75 total plans for comparison with limited manual intervention. In comparing optimization techniques, the Dose/Volume and Lines optimization templates generated plans with similar DVH metrics, with a slight preference for the Lines technique with reductions in heart V30Gy and spinal cord max dose. The gEUD model produced high target heterogeneity.

**Conclusion:**

Automated evaluation allowed for the exploration of multiple optimization templates in a larger validation cohort than would have been feasible using a manual approach. A final KBP model using line optimization objectives produced the highest quality plans without human intervention.

## 1 INTRODUCTION

Radiation treatment plan quality across institutions or even across planners within a given institution can be highly heterogeneous, partially due to planner experience.^1^, ^2^ Knowledge‐based planning (KBP) is an approach that has been shown to improve plan quality in multi‐institutional clinical trials,^3^, ^4^ and identify low‐quality treatment plans during peer review.^5^, ^6^, ^7^

There is great interest within radiation oncology for developing high‐quality KBP models, as evidenced by the 2020 OpenKBP AAPM Grand Challenge, which included 195 participants from 28 countries.^8^ KBP models are typically used to either directly estimate a dose distribution^9^, ^10^, ^11^, ^12^ or predict a dose‐volume histogram.^13^, ^14^ RapidPlan (Varian Medical Systems) is a KBP tool that has received support from the community and is being integrated into many radiotherapy clinics, partially because it streamlines the model training process by incorporating many aspects of model training within the Eclipse Treatment Planning System (TPS, Varian Medical Systems). Many studies have showcased validation and clinical implementation by using the model to generate new plans for retrospective evaluation against reference plans.^15^ However, this process is labor intensive and subjective. Additionally, within the RapidPlan model, output plan quality is largely driven by the optimization objectives used to drive automated plan generation. As specific optimization objective templates are paired with models in the RapidPlan workspace, it can be difficult to decouple the impact of the specific optimization template on output plan quality. Without an automated solution, testing of multiple optimization templates can be prohibitively time‐consuming.

In this study, we developed an application, RapidCompare, which automatically generates radiation treatment plans for a list of previously treated patients using a RapidPlan model and associated objective template. After plan optimization and calculation, the tool creates a report describing various plan quality metrics by comparing the RapidPlan‐generated plans to a set of reference plans. To show the utility of RapidCompare, we analyze three different optimization templates from the same RapidPlan model and compare output plan quality from the different planning approaches.

## 2 METHODS

### 2.1 RapidCompare

**FIGURE 1:**
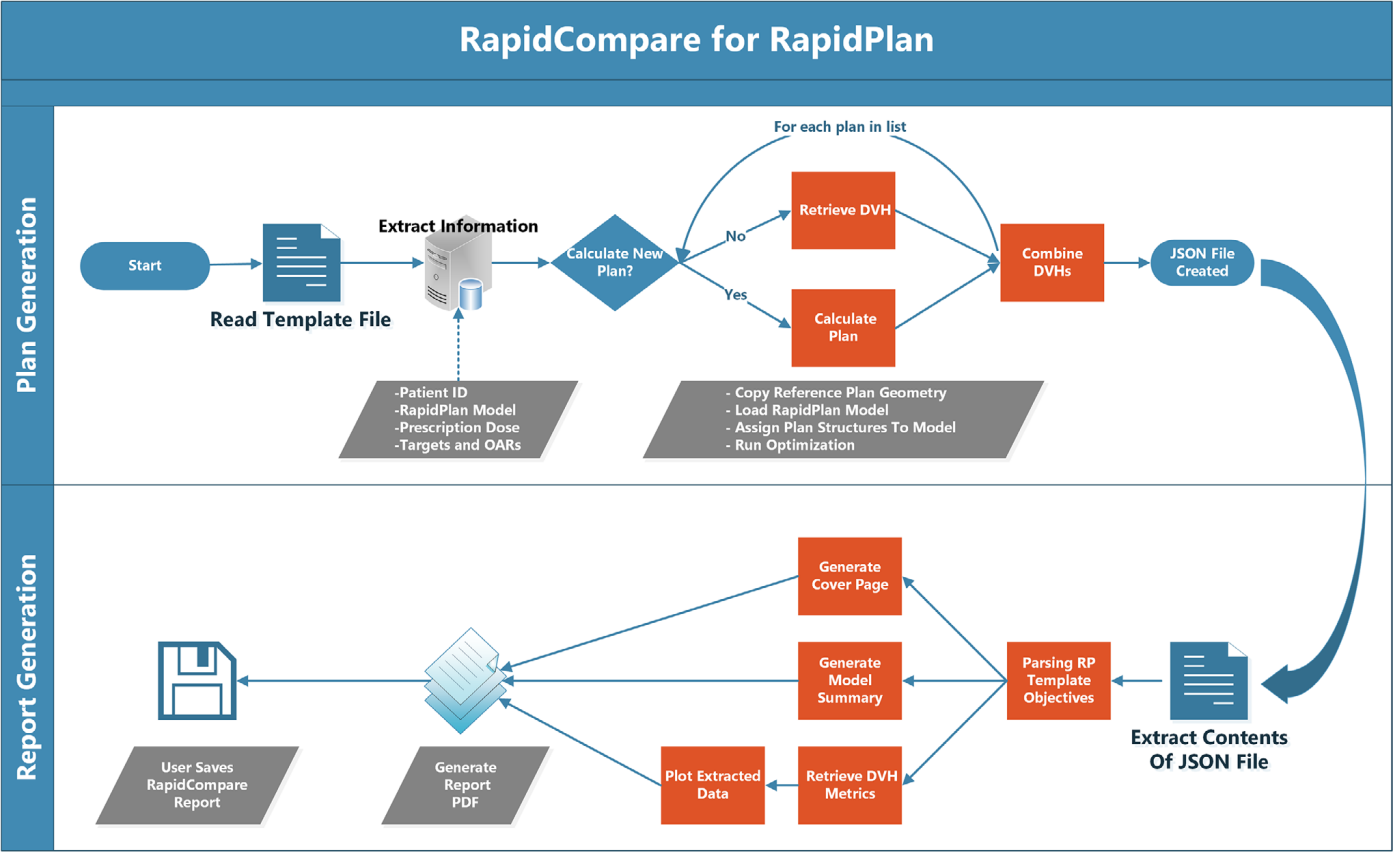
Flowchart describing the RapidCompare tool. The script is modular with two components, plan generation and DVH extraction (top) and then report generation (bottom). User intervention is required to select the file to read to initiate each process and to specify whether new plans should be calculated or prior plan DVH metrics extracted, but all other steps shown in the figure are automated.

The Eclipse Scripting Application Programming Interface (ESAPI, Varian Medical Systems) was used to create a .NET‐based application, RapidCompare, that allows for automated validation of RapidPlan models. The workflow of the application is shown in Figure 1. RapidCompare is split into two modules, first for plan generation and DVH extraction and then for final report generation.

The plan generation process starts by reading an initial template file. In the file header, the user has the option to specify the dose calculation algorithm, optimizer calculation options, and the RapidPlan model to be used for optimization. The body of the template file contains a list of reference plans, organized by medical record number, plan, and course name. For each patient, the user also specifies the new plan name and matches the structures from the patient dataset to the structures in the RapidPlan model. After structure matching, any target structures and their corresponding prescription doses are specified, with an option to specify multiple targets and multiple dose levels. After the template file has been parsed, the user is asked to specify if new plans are to be calculated. If “yes” is selected, new plans will be automatically calculated using the plan geometry from the reference plan. If “no” is selected, the script extracts the DVHs for all listed structures for a given patient for both the reference plan and the RapidPlan plan name specified in the list. As the module iterates through each patient in the list, it extracts DVH data and writes to a .JSON file. The RapidPlan model and optimization objective templates are also written in the file.

To begin the report generation process, the user launches the application and is prompted to select the .JSON file to read. RapidCompare then parses the RapidPlan template information and generates a cover page and model summary. It also retrieves DVH metrics matching the dose/volume pairs included in the optimization objective template. Metrics are extracted for both the reference plan and the RapidPlan plan. Using these DVH metrics, the tool creates box plots and bar charts. It also generates plots of cohort‐averaged DVH for each structure in the model in addition to OAR DVHs overlaid with DVH estimates for each patient. RapidCompare does not require any special support or setup in Eclipse beyond a standard ESAPI environment. The base application was set to run on our Eclipse thick client and plans were calculated on our clinical distributed calculation framework (DCF) with 14 servers.

### 2.2 Patient cohort

In this IRB‐approved ([IRB number redacted for peer review]) retrospective study, a RapidPlan model trained with conventionally fractionated radiotherapy plans in the lungs was used for all testing. The cohort included datasets from 85 patients treated with IMRT or VMAT at our institution between 2018 and 2021. The patient datasets were divided into training (50 cases), validation (10), and testing (25) cohorts. Most patients in the training cohort (44/50) were treated to 60 Gy in 30 fractions, three patients were treated to 45 Gy in 15 fractions, and three patients to 66 Gy in 33 fractions. All patients in the testing cohort were treated with 60 Gy in 30 fractions. In the validation cohort, 23 patients received 60 Gy in 30 fractions. Two patients who received 60 Gy in 30 fractions total but were adaptively replanned midway through treatment due to tumor shrinkage were also included. These replans were prescribed 2 Gy per fraction to a total of 30 and 36 Gy. As the size of the target volume is a driving factor behind DVH estimation, each of the cohorts was randomly sampled so that the distribution of PTVs in each cohort was preserved, as shown in Table 1.

**TABLE 1:** Laterality and target volumes for each cohort.

	Training	Testing	Validation
Median [IQR] PTV	422.2 cc [235.9–588.9 cc]	439.5 cc [241.2–696.6 cc]	421.4 cc [342.0–638.3 cc]
N left (%)	25 (50%)	4 (40%)	11 (44%)
N right (%)	25 (50%)	6 (60%)	14 (56%)

### 2.3 Optimization template comparison

A RapidPlan model is trained using a list of radiation treatment plans. From this list of plans, a correlation between achieved plan DVHs, patient anatomy, and beam geometry is mapped, and after training, the model can predict a DVH with upper and lower confidence bounds using only beam geometry, CT image, and structure set. Optimization constraints can be added as simple dose/volume, generalized equivalent uniform dose (gEUD), or line constraints. In the case of dose/volume pairs and gEUD, these are calculated directly from the lower bound of the estimated DVH. A line constraint is defined as a series of dose/volume constraints applied across the entire lower estimate of the DVH. The user can specify the priority for any constraint to be generated based on the patient's anatomy or have a fixed value.

To independently test the impact of the optimization template, a model was trained using the same 50 cases and then duplicated and associated with three different optimization templates. The structures included for all patients in the model were ipsilateral and contralateral lung, heart, spinal cord, and esophagus. The ipsilateral brachial plexus was included for 25 patients, as our clinical practice guidelines only mandate contouring this structure when target volumes are in proximity. Similarly, the trachea and main stem bronchi were included in the model but were excluded from analysis because very few cases in the validation cohort had these structures contoured. All templates shared the same metrics for the high dose target volume (PTV) and had the same normal tissue optimization (NTO) parameters. OAR constraints were given generated priorities and the NTO had a priority of 80. All PTVs had a maximum dose goal of 102% and a minimum dose goal of 101%, with a priority of 100 on each goal.

The OAR objectives for each of the three templates used for testing are shown in Table 2. The first model included dose/volume pairs at points pertaining to constraints often used in clinical practice. The second model included only gEUD constraints. When specifying the gEUD, a high exponential value was used for serial organs and a low value for parallel organs.^16^ For organs where both high and intermediate doses are known to cause toxicity, multiple gEUDs were applied. The final template for comparison included only line constraints.

**TABLE 2:** Optimization templates for the RapidPlan models tested in this study.

	Comparison model		
Structure	Dose/volume	gEUD alpha	Line
Ipsilateral brachial plexus	V100%	*a* = 40	Prefer target
Esophagus	V100% V35 Gy V50 Gy	*a* = 1 *a* = 40	Prefer target
Heart	V30 Gy V45 Gy V60 Gy	*a* = 1 *a* = 40	Prefer target
Contralateral lung	V5 Gy V20 Gy	*a* = 1 *a* = 20	Prefer target
Ipsilateral lung	V5 Gy V20 Gy	*a* = 1 *a* = 20	Prefer target
Spinal cord	V45 Gy	*a* = 40	Prefer OAR
Spinal cord + 3 mm	V45 Gy	*a* = 40	Prefer target
Spinal cord + 5 mm	V50 Gy	*a* = 40	Prefer target

The reference plans in this study were the clinically approved and delivered treatment plans. All comparison plans were calculated using the geometry from the reference (clinical) plan. During optimization, all VMAT plans were calculated with convergence mode and second‐order air cavity corrections, and intermediate‐dose calculation was selected. The final dose is calculated using the Analytical Anisotropic Algorithm version 13.6.23. All plans were normalized so that 95% of the target was covered by 100% of the prescription dose. All comparison plans were automatically created with no human intervention.

In addition to creating plans and generating a report with plan dosimetry, the RapidCompare tool reports a timestamp for each step in the plan generation and optimization process. It also reports the final objective function value for each structure. Using this information, we calculated the time for plan generation with each model and the average distribution of the objective function. For a fair comparison of objective weights between model types, the final objective values for a given plan were normalized so that the total optimization objective weight across the PTV, body, esophagus, heart, and ipsilateral and contralateral lungs was 100.

Plan metrics and dose volume histograms (DVH) were extracted using ESAPI. The Wilcoxon paired, non‐parametric test was utilized to test the difference between reference and KBP plans. P‐values were calculated, without removal of outliers, using the SciPy library in Python (version 3). When conducting multiple tests on the same dependent variable, the likelihood of observing a significant result by pure chance increases. A Bonferroni correction was therefore applied to adjust for multiple testing (*n* = 12); thus, *p* < 0.004 (0.05/12) is considered significant when comparing metrics among different models.

## 3 RESULTS

RapidCompare allowed for the generation of 75 plans of comparison (3 arms of 25 patients each). The required manual intervention was minimal and consisted of making the template file, creating optimization objective templates with each model, and running the application. A separate script was developed for creating the template in an automated manner, so the time spent on the other activities, including training the model and selecting cases for the model was less than 2 h.

**FIGURE 2:**
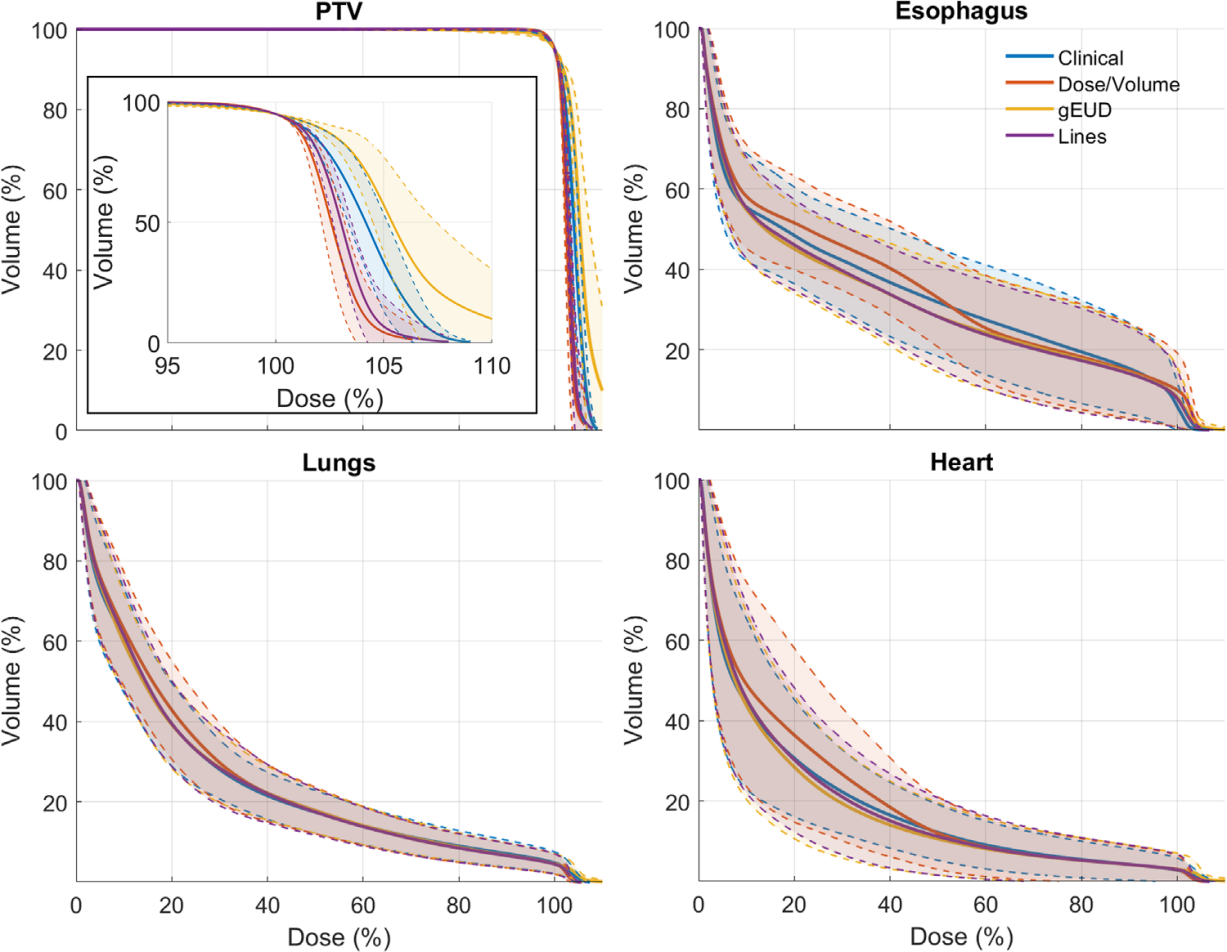
The validation cohort averaged DVHs for the PTV, esophagus, lungs, and heart. The shaded regions show ±1 standard deviation, and the inset on the PTV plot shows the high dose region of the PTV DVH.

**FIGURE 3:**
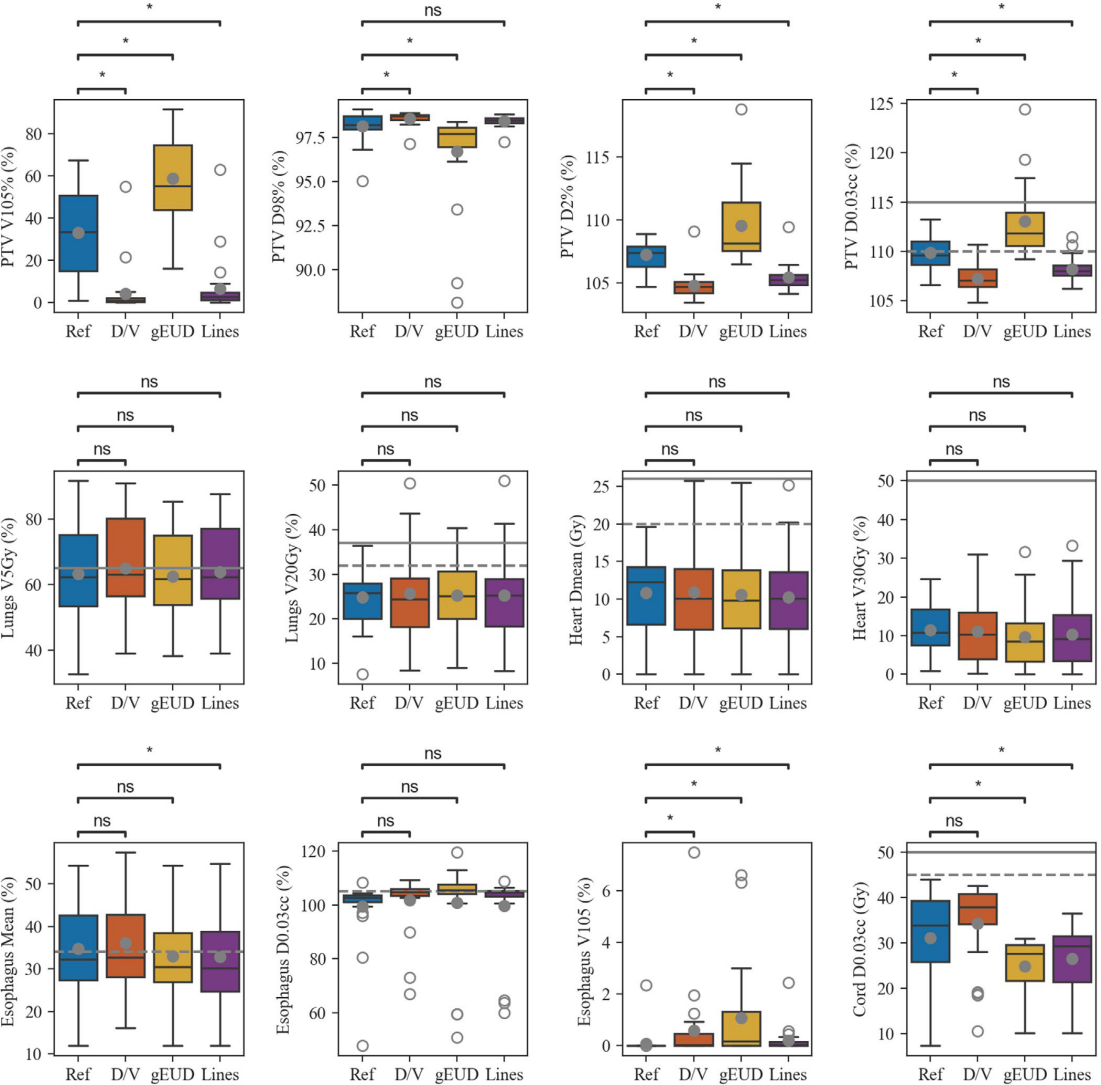
Boxplots for evaluated dose metrics for the reference plans and all Rapidplans. Open and closed circles indicate outlier and mean values, respectively. Dashed and solid lines indicate soft and hard constraints, respectively. Significance values obtained via the Wilcoxon signed rank test are annotated as follows: ns (not significant): (0.004, *p*, 1.00]; *: (0, *p*, 0.004].

The average validation cohort DVHs from the clinical and automated plans are presented in Figure 2. Figure 3 shows boxplots of evaluated DVH metrics from each model type. Both the Dose/Volume and Lines models had more homogenous PTV coverage than the clinical plans or the gEUD plans. In terms of OAR sparing, the Lines model most closely reflected the clinical plans in both the lungs and the heart. It also showed improvement at most lower esophagus doses, but had a slightly higher max dose, as seen in Figure 3 and Table 3. While gEUD plans had slightly improved OAR sparing compared with both the clinical and Lines plans, the target coverage was more heterogeneous. While all the RapidPlan models showed statistically significant reductions in esophageal mean dose (*p* = 0.02, *p* = 0.01, and *p* = 0.001 for Dose/Volume, gEUD, and Lines, respectively), they also all had increases in both esophageal max dose and V105% (*p* ≤ 0.01 for all). For the Dose/Volume, gEUD, and Lines models, three plans violated the hard constraint of lung V20Gy < 37% according to our standard clinical protocol, as seen in Table 3. In addition to the V20Gy violations, the gEUD RapidPlan had an unacceptably high D0.03cc (> 115%) in the PTV for 6 cases.

**TABLE 3:** Number (percentage) of cases that met constraints for each model.

Structure	Constraint	Clinical	Dose/Volume	gEUD	Lines
PTV	D0.03cc < 110%	13 (52%)	23 (92%)	4 (16%)	23 (92%)
	**D0.03cc < 115%**	**25 (100%)**	**25 (100%)**	**19 (76%)**	**25 (100%)**
Esophagus	D0.03cc < 105%	24 (96%)	15 (60%)	9 (36%)	15 (60%)
	DMean < 34 Gy	25 (100%)	25 (100%)	25 (100%)	25 (100%)
Heart	DMean < 20 Gy	25 (100%)	23 (92%)	23 (92%)	23 (92%)
	**DMean < 26** **Gy**	**25 (100%)**	**25 (100%)**	**25 (100%)**	**25 (100%)**
Lungs	V20Gy < 33%	20 (80%)	20 (80%)	21 (84%)	21 (84%)
	**V20Gy < 37%**	**25 (100%)**	**22 (88%)**	**22 (88%)**	**22 (88%)**
	V5Gy < 65%	15 (60%)	15 (60%)	16 (64%)	15 (60%)
Spinal cord	D0.03cc < 45 Gy	25 (100%)	25 (100%)	25 (100%)	25 (100%)
	**D0.03cc < 50** **Gy**	**25 (100%)**	**25 (100%)**	**25 (100%)**	**25 (100%)**

*Note*: Constraints come from our institutional clinical protocols which were developed based on consensus among disease site‐specific physician groups. Hard constraints are bolded.

**FIGURE 4:**
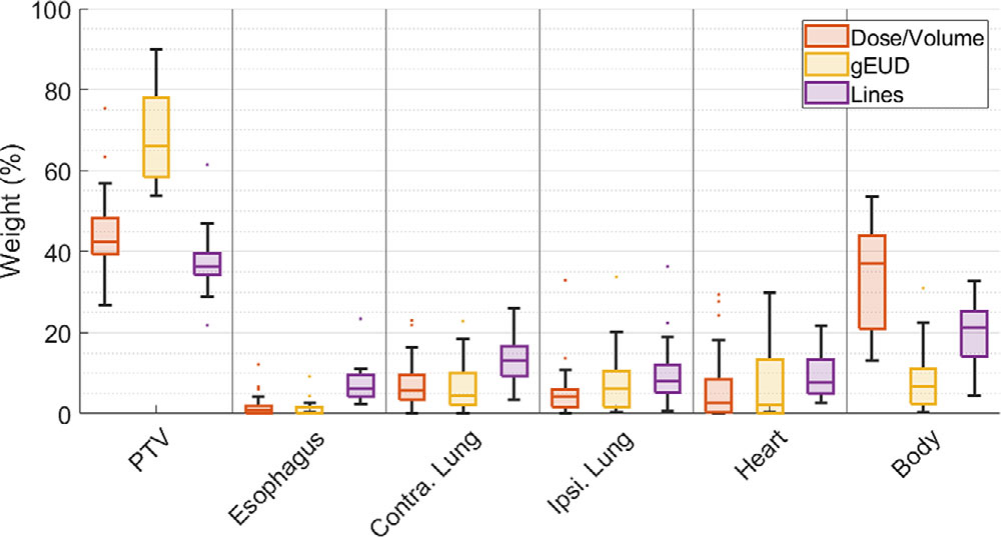
Boxplot of the final optimization objective weights for each of the RapidPlan models across all 25 patients in the validation cohort. The weights are normalized such that the final objective weight across the six structures shown in the plot adds up to 100 for a given plan.

The optimization objective weight for each structure with each of the models is shown in Figure 4. The weights are normalized objective function values at the end of the optimization and essentially show how hard the optimizer is working to meet the constraints specified for each structure. In analysis of objective function weights, the body and PTV were the two most highly weighted structures for the Lines and Dose/Volume models. The gEUD model had the highest average PTV weight, 69 ± 11% versus 45 ± 10% for the Dose/Volume model and 37 ± 7% for the Lines model. Overall, the Lines models had the most even distribution across all structures, attributing more equal weight to each OAR.

## 4 DISCUSSION

Knowledge‐based planning, and RapidPlan specifically, allows for automatic generation of high‐quality plans when models are trained and implemented appropriately. In this study, we characterized RapidCompare, an efficient tool for quantitatively evaluating RapidPlan models. We then used the tool to compare the differences between output plan quality when using different optimization objective templates. We found through a systematic analysis that optimization approaches using both Dose/Volume constraints and Line constraints produced clinically acceptable plans for conventionally fractionated lung, with the Lines model being preferred. The use of gEUD constraints alone led to unacceptable target heterogeneity. Based on these findings, the clinical protocol for conventionally fractionated lung RapidPlan at our institution includes Lines‐based models which are used on every case with convergence mode and air cavity corrections selected.

An important component of releasing any automated process to the clinic is commissioning, including quantitative analysis. To our knowledge, the RapidCompare tool is the first solution introduced to provide an automated means for full quantitative evaluation of a RapidPlan model, greatly decreasing the active workload for the physics and dosimetry teams ahead of the clinical release of RapidPlan models. Furthermore, the final report generated by this tool (see Supplemental Materials) serves as a commissioning report that can be systematically reviewed by other radiation oncology team members prior to the clinical release of individual RapidPlan models, ensuring that resulting automated strategies are consistent with institutional practice guidelines. We anticipate that, in addition to model development and commissioning, RapidCompare will be useful for ongoing assessment of RapidPlan model performance. It could be used to quickly generate comparison plans for more recent cases, to ensure that a given RapidPlan model is still producing plans that are consistent with changes in practice patterns over time.

One of the key limitations in incorporating RapidCompare into a RapidPlan commissioning timeline is that it does not solve the barrier of finding appropriate cases. We have developed helper ESAPI scripts to search for cases for testing models by filtering the Eclipse database based on fractionation; however, this is still an imperfect solution. Additionally, the template file needs to be built for each model, and this can still be labor‐intensive. Finally, when calculating plans, the shared clinical DCF is used, so this can limit usability during business hours in a busy clinical environment. For this study, all batch runs of the plan generation phase of RapidCompare were run overnight to mitigate the impact on daily clinical workflow.

The source code for RapidCompare can be shared by request to the corresponding author and will also be available for download on GitHub at https://github.com/UAB‐RO.

## 5 CONCLUSION

In this study, RapidCompare, an automated tool for rapid, quantitative assessment of RapidPlan models was developed and tested. Using this tool, we were able to easily test the impact of varying optimization parameters on automated plan quality, comparing three different optimization approaches across a cohort of 25 patients. We found that the use of line constraints, which constrain all points along the DVH during optimization produced plans with the most similar quality to previously treated clinical plans.

## AUTHOR CONTRIBUTIONS

Joseph Harms and Richard Popple contributed to the conception of this work and designed the experiments. All authors put forth significant effort in the acquisition and analysis of the data, contributed to the interpretation of the data, as well as the writing, preparation, and editing of the manuscript. All authors have given final approval of this submission and agree to be accountable for all aspects of the work in ensuring that questions related to the accuracy or integrity of any part of the work are appropriately investigated and resolved.

## CONFLICT OF INTEREST STATEMENT

The authors' institution, the University of Alabama at Birmingham, has product evaluation agreements and research grants with Varian Medical Systems. Varian Medical Systems provides equipment to UAB as a part of a product evaluation agreement. Richard Popple has a patent licensed by the UAB Research Foundation to Varian Medical Systems. He has received honoraria for presentations on behalf of Varian Medical Systems and a stipend to speak at Sun Nuclear meetings, not related to this work. Dennis Stanley received honoraria from Varian Medical Systems to present on their behalf and is an educational consultant speaker for Varian Medical Systems. He received support through a clinical trial sponsored by Varian Medical Systems. Rex Cardan has received honoraria from Varian Medical Systems and compensation as a consultant, not related to this work.

## Supporting information

Supporting information
