# Supplementary material for: Automated evaluation for rapid implementation of knowledge‐based radiotherapy planning models
Source: J Appl Clin Med Phys. 2023 Sep 13;24(10):e14152. doi: 10.1002/acm2.14152 (PMC10562024; doi:10.1002/acm2.14152)
Supplement: Supplementary file 1 — Supporting information [file ACM2-24-e14152-s001.pdf]

Example of an input file for RapidCompare. The columns selecting courses for the reference and rapidplan have been omitted for visualization. This file is read into the application as a tab-delimited text file.

| | | |
|---|---|---|
| RapidPlanModelID | z_Lung_T_Clinical | |
| DVHEstimation | DVH Estimation Algorithm [16.1.0] | |
| PhotonVMATOptimization | PO_1610 | |
| PhotonVMATOptimizationOptions | VMAT/ConvergenceMode | On |
| PhotonVMATOptimizationOptions | General/OptimizerSettings/UseGPU | No |
| PhotonVMATOptimizationOptions | General/OptimizerSettings/AirCavityCorrection | On |
| PhotonVMATOptimizationOptions | General/OptimizerSettings/AutomaticIntermediateDose | On |
| PhotonIMRTOptimization | PO_1610 | |
| PhotonLeafMotions | Varian Leaf Motion Calculator [16.1.0] | |
| PhotonVolumeDose | AAA_13623 | |

| **PatientID** | **ReferencePlanID** | **RapidPlanID** | **BrachPlex_Ipsi** | **Esophagus** | **Heart** | **Lung_Contra** | **Lung_Ipsi** | **SpinalCord** | **PTV** | **Dose[cGy]** |
|---|---|---|---|---|---|---|---|---|---|---|
| Anon1 | 1.1 R Lung | RP_LungVal | | Esophagus | Heart | Lung_L | Lung_R | SpinalCord | PTV | 6000 |
| Anon2 | 1.1 Lung | RP_LungVal | BrachialPlex_L | Esophagus | Heart | Lung_R | Lung_L | SpinalCord | PTV | 6000 |
| Anon3 | 1.1 L Lung | RP_LungVal | BrachialPlex_L | Esophagus | Heart | Lung_R | Lung_L | SpinalCord | PTV | 6000 |
| Anon4 | 2.1 R Lung | RP_LungVal | BrachialPlex_R | Esophagus | Heart | Lung_L | Lung_R | SpinalCord | PTV | 6000 |
| Anon5 | 1.1 R Lung | RP_LungVal | | Esophagus | Heart | Lung_L | Lung_R | SpinalCord | PTV | 6000 |
| Anon6 | 1.1R1 L Lung | RP_LungValR1 | | Esophagus | Heart | Lung_R | Lung_L | SpinalCord | PTV | 3600 |
| Anon7 | 1.1 L Lung | RP_LungVal | BrachialPlex_L | Esophagus | Heart | Lung_R | Lung_L | SpinalCord | PTV | 6000 |
| Anon8 | 2.1 R Lung | RP_LungVal | | Esophagus | Heart | Lung_L | Lung_R | SpinalCord | PTV1 | 6000 |
| Anon9 | 1.1 L Lung | RP_LungVal | BrachialPlex_L | Esophagus | Heart | Lung_R | Lung_L | SpinalCord | PTV | 6000 |
| Anon10 | 1.1 R Lung | RP_LungVal | BrachialPlex_R | Esophagus | Heart | Lung_L | Lung_R | SpinalCord | PTV | 6000 |
| Anon11 | 1.1 R Lung | RP_LungVal | | Esophagus | Heart | Lung_L | Lung_R | SpinalCord | PTV | 6000 |
| Anon12 | 1.1 Lung | RP_LungVal | BrachialPlex_R | Esophagus | Heart | Lung_L | Lung_R | SpinalCord | PTV | 6000 |
| Anon13 | 1.1 R Lung FB | RP_LungVal | BrachialPlex_R | Esophagus | Heart | Lung_L | Lung_R | SpinalCord | PTV | 6000 |
| Anon14 | 2.1 L Lung | RP_LungVal | | Esophagus | Heart | Lung_R | Lung_L | SpinalCord | PTV | 6000 |
| Anon15 | 2.1 R Lung | RP_LungVal | BrachialPlex_R | Esophagus | Heart | Lung_L | Lung_R | SpinalCord | PTV | 6000 |
| Anon16 | 1.1 L Lung | RP_LungVal | | Esophagus | Heart | Lung_R | Lung_L | SpinalCord | PTV | 6000 |
| Anon17 | 1.1 R Lung | RP_LungVal | BrachialPlex_R | Esophagus | Heart | Lung_L | Lung_R | SpinalCord | PTV | 6000 |
| Anon18 | 1.1 R Lung | RP_LungVal | | Esophagus | Heart | Lung_L | Lung_R | SpinalCord | PTV | 6000 |
| Anon19 | 2.1 L Lung | RP_LungVal | BrachialPlex_L | Esophagus | Heart | Lung_R | Lung_L | SpinalCord | PTV | 6000 |
| Anon20 | 2.1R1 L Lung | RP_LungValR1 | BrachialPlex_L | Esophagus | Heart | Lung_R | Lung_L | SpinalCord | PTV^R1 | 3000 |
| Anon21 | 1.1 R Lung | RP_LungVal | | Esophagus | Heart | Lung_L | Lung_R | SpinalCord | PTV | 6000 |
| Anon22 | 1.1 R Lung | RP_LungVal | BrachialPlex_R | Esophagus | Heart | Lung_L | Lung_R | SpinalCord | PTV_High | 6000 |
| Anon23 | 1.1 L Lung | RP_LungVal | | Esophagus | Heart | Lung_R | Lung_L | SpinalCord | PTV_Lung | 6000 |

Example of the PDF report generated by RapidCompare to assist in model commissioning. Note that although the Lines model was used to generate this report, some structures have been removed for brevity in the report, and thus some OAR and target statistics in the following report are slightly different from those reported in the manuscript.

# RapidPlan evaluation
# zLung_Clinical

April 18, 2023

# Model Summary

Model ID: zLung_Clinical
Model Version: 16.1.0
Description: Lines model for automated planning in conventionally-fractionated lung treatments
Number of training cases: 50

### Optimization objectives and optimization settings

| Structure | Objective type | Relative volume (%) | Dose | Priority |
|---|---|---|---|---|
| BrachPlex_Ipsi | Line (preferring target) | Generated | Generated | Generated |
| | Upper | 0% | 66 Gy | 0 |
| Esophagus | Upper | 0% | 105 Percent | 0 |
| | Mean | Generated | 34 Gy | 0 |
| | Line (preferring OAR) | Generated | Generated | Generated |
| Heart | Line (preferring target) | Generated | Generated | Generated |
| | Upper | 46% | 30 Gy | 0 |
| | Mean | Generated | 26 Gy | 0 |
| Lung_Contra | Line (preferring target) | Generated | Generated | Generated |
| | Upper | 30% | 20 Gy | 0 |
| | Upper | 60% | 5 Gy | 0 |
| | Mean | Generated | 20 Gy | 0 |
| Lung_Ipsi | Line (preferring target) | Generated | Generated | Generated |
| | Upper | 30% | 20 Gy | 0 |
| | Upper | 60% | 5 Gy | 0 |
| | Mean | Generated | 20 Gy | 0 |
| PTV | Upper | 0% | 102 Percent | 100 |
| | Upper | 2% | 105 Percent | 0 |
| | Lower | 100% | 101 Percent | 100 |
| | Lower | 98% | 98 Percent | 0 |
| SpinalCord | Line (preferring OAR) | Generated | Generated | Generated |
| | Upper | 0% | 50 Gy | 0 |
| SpinalCord_PRV03 | Line (preferring target) | Generated | Generated | Generated |
| SpinalCord_PRV0 | Line (preferring target) | Generated | Generated | Generated |

### Structure codes

| Structure | Codes |
|---|---|
| BrachPlex_Ipsi | 45245 (FMA) |
| Esophagus | 7131 (FMA) |
| Heart | 7088 (FMA) |

| | |
|---|---|
| Lung_Contra | 68877 (FMA) |
| Lung_Ipsi | 68877 (FMA) |
| PTV | PTV_High (99VMS_STRUCTCODE) |
| SpinalCord | 7647 (FMA) |
| SpinalCord_PRV03 | PRV (99VMS_STRUCTCODE) |
| SpinalCord_PRV0 | PRV (99VMS_STRUCTCODE) |

# Dose-volume metric summary

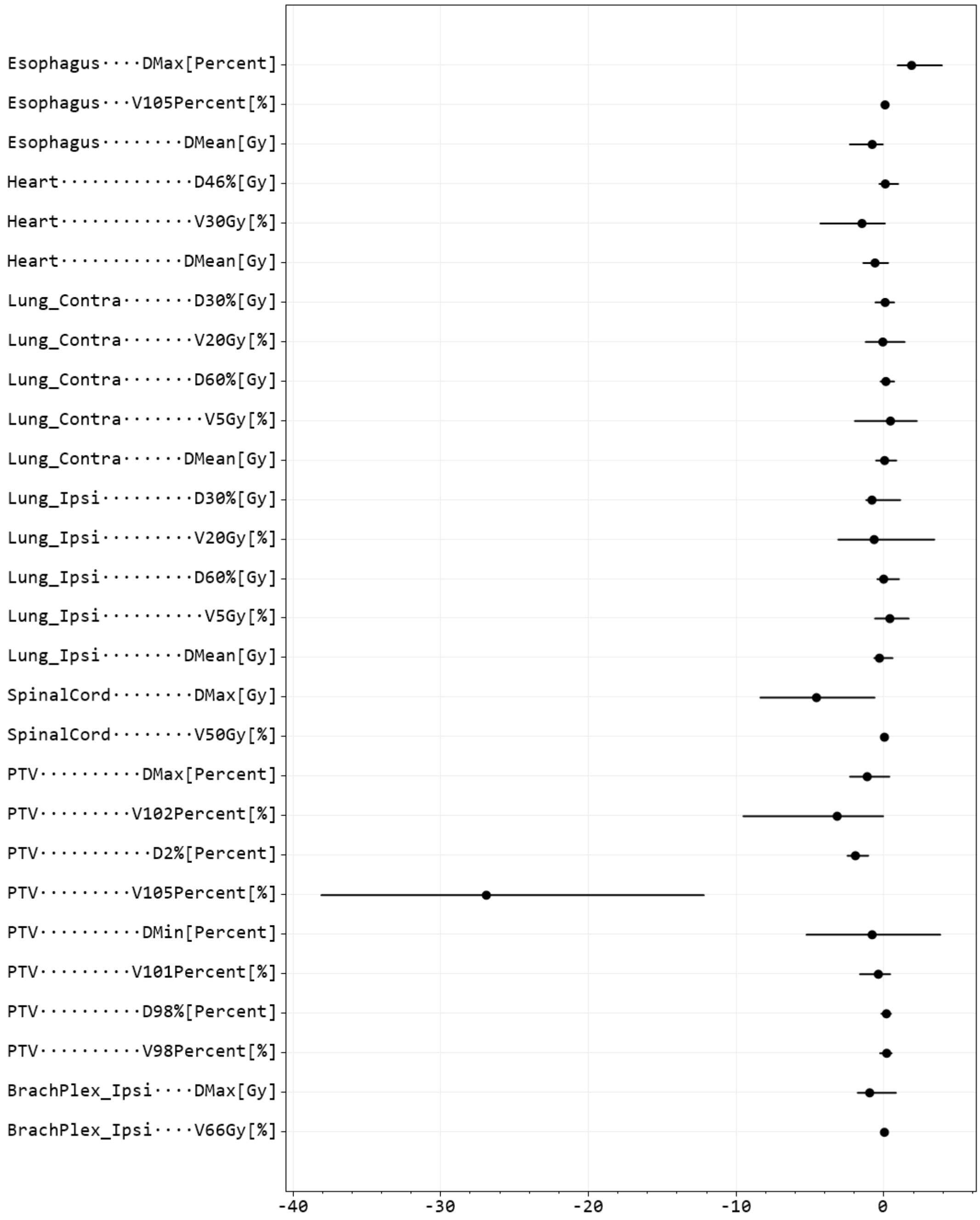

# Esophagus

## DVH Volume difference

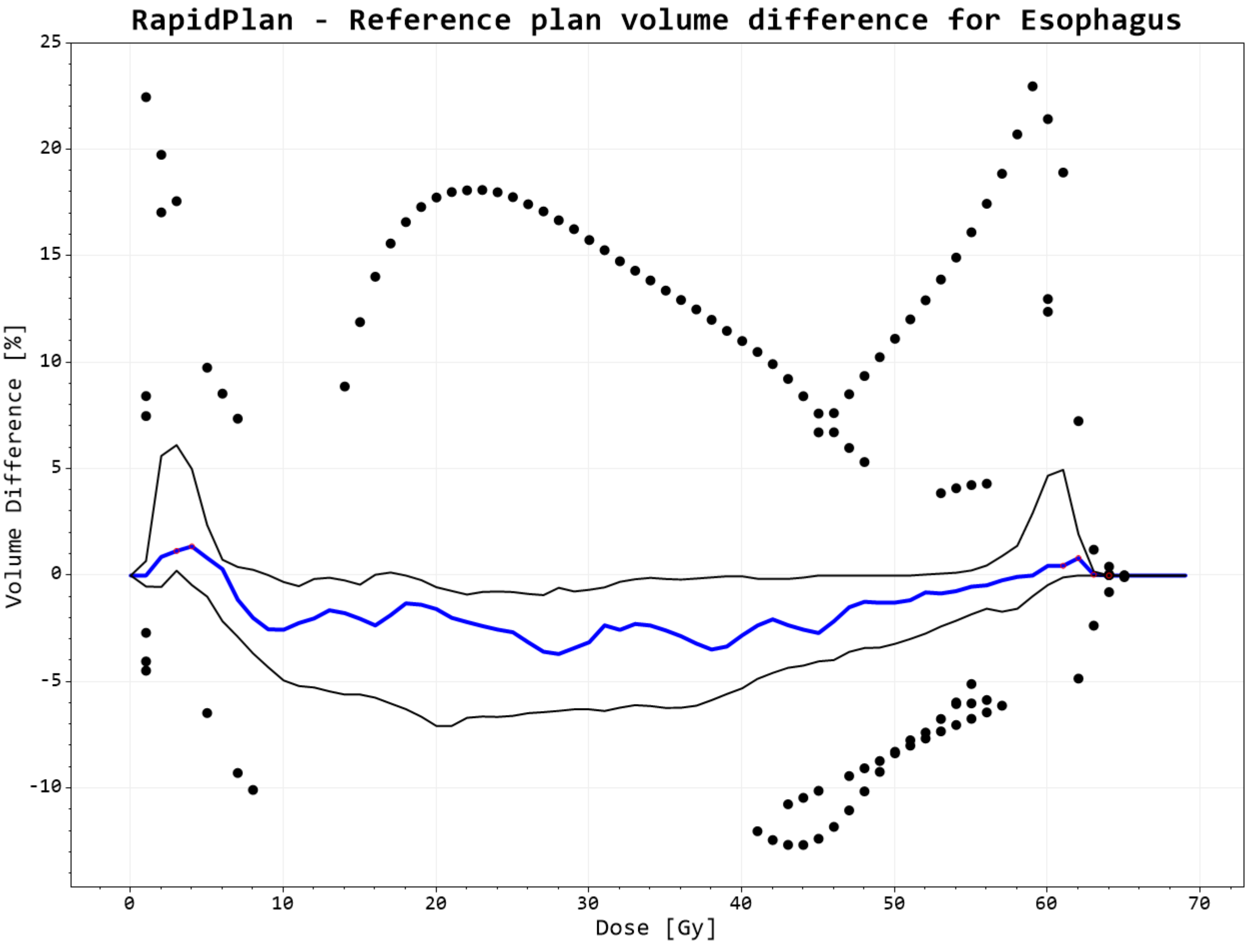


## Dose-volume metric summary table

| Metric | Reference Plan | RapidPlan | Difference |
|---|---|---|---|
| DMax[Percent] | 104.7 [102.5,105.0] | 105.8 [104.6,107.6] | 1.8 [0.9,3.9] (p = 0.000) (2) |
| V105Percent[%] | 0.0 [0.0,0.0] | 0.0 [0.0,0.2] | 0.0 [0.0,0.2] (p = 0.000) (0) |
| DMean[Gy] | 19.4 [16.2,25.8] | 18.1 [14.7,23.8] | -0.8 [-2.3,-0.1] (p = 1.000) (1) |

## Dose-volume metric box whisker plots

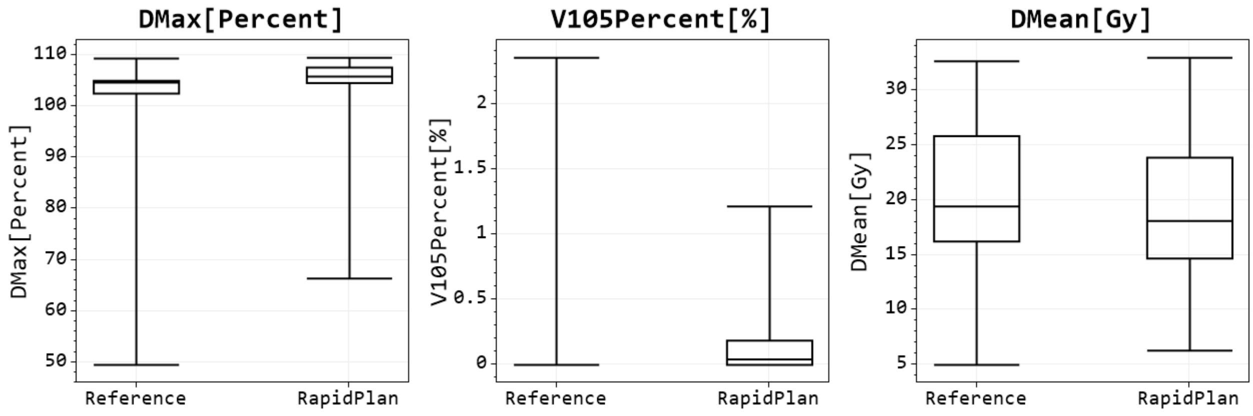

## Dose-volume metric differences by plan

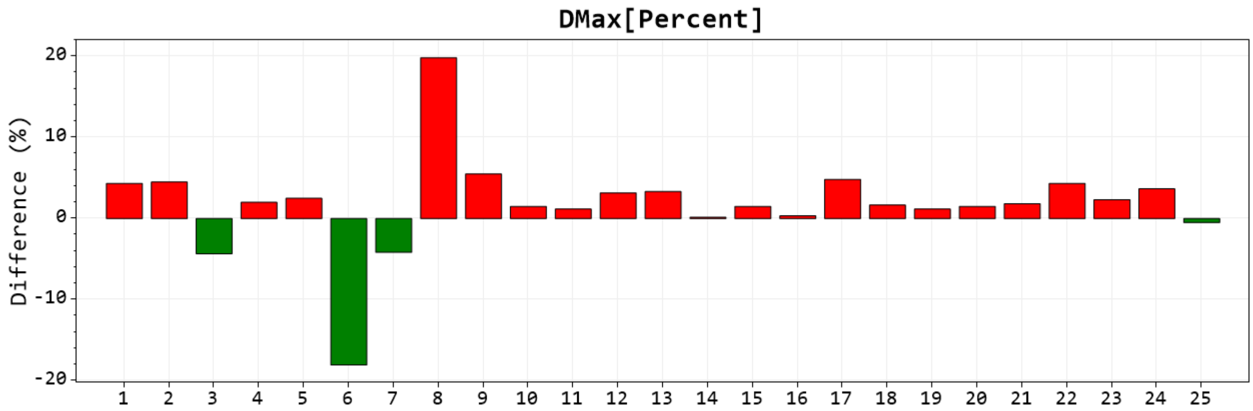


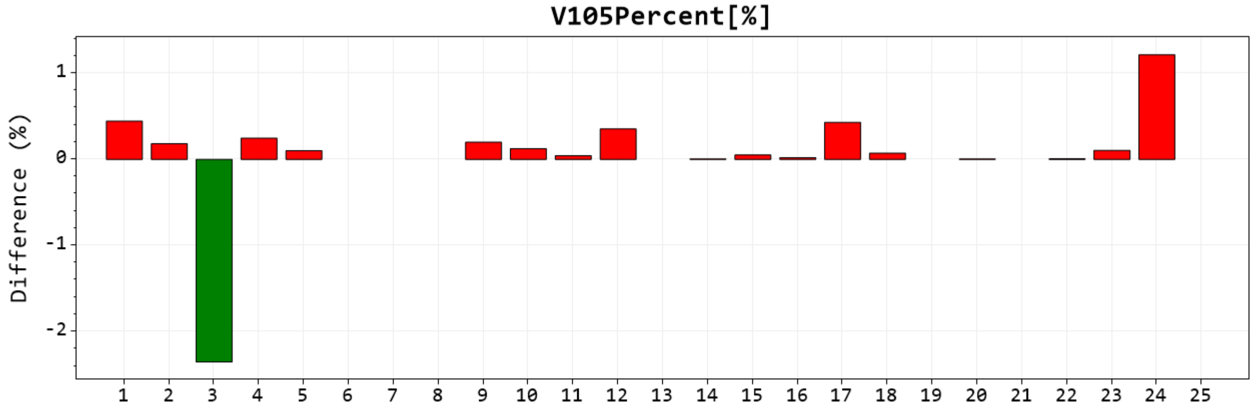


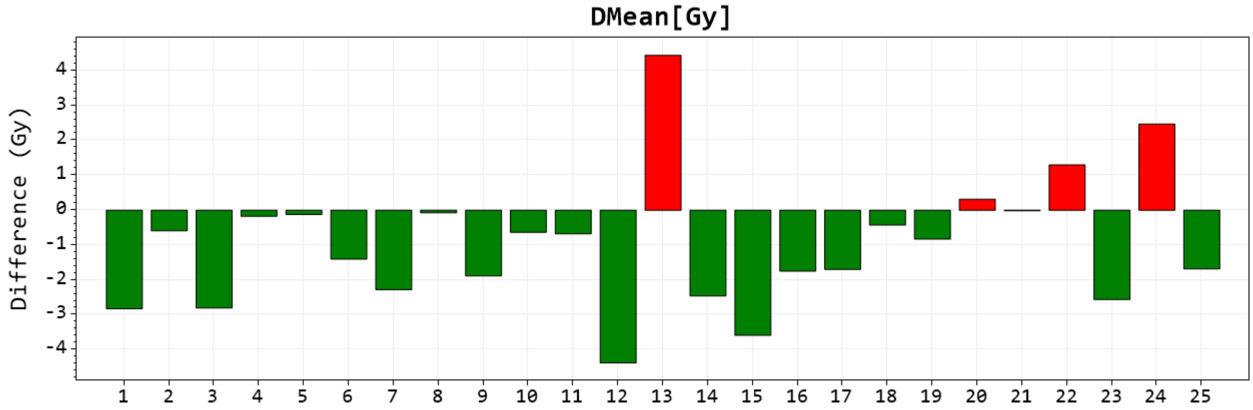

## Dose-volume histograms

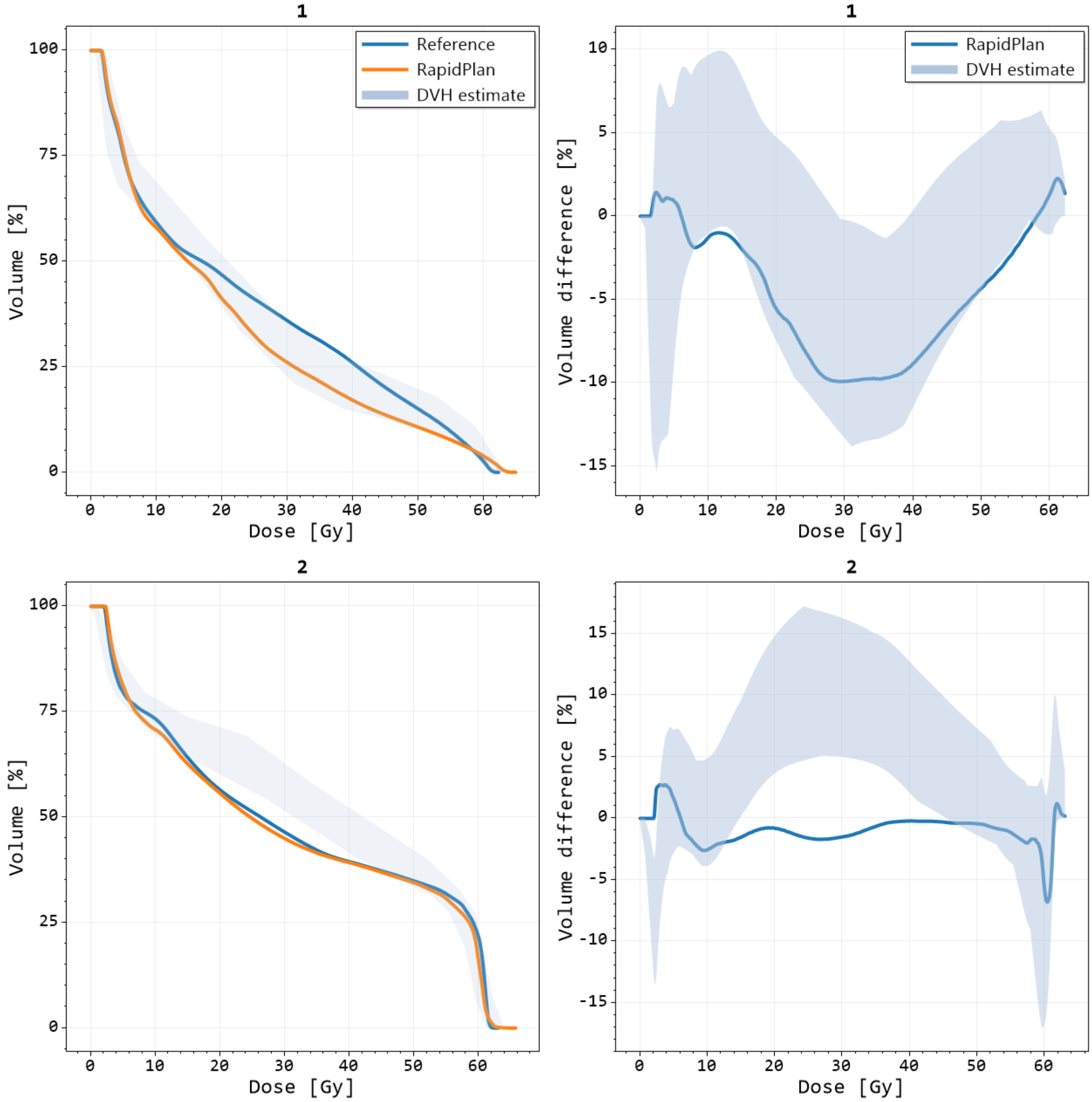

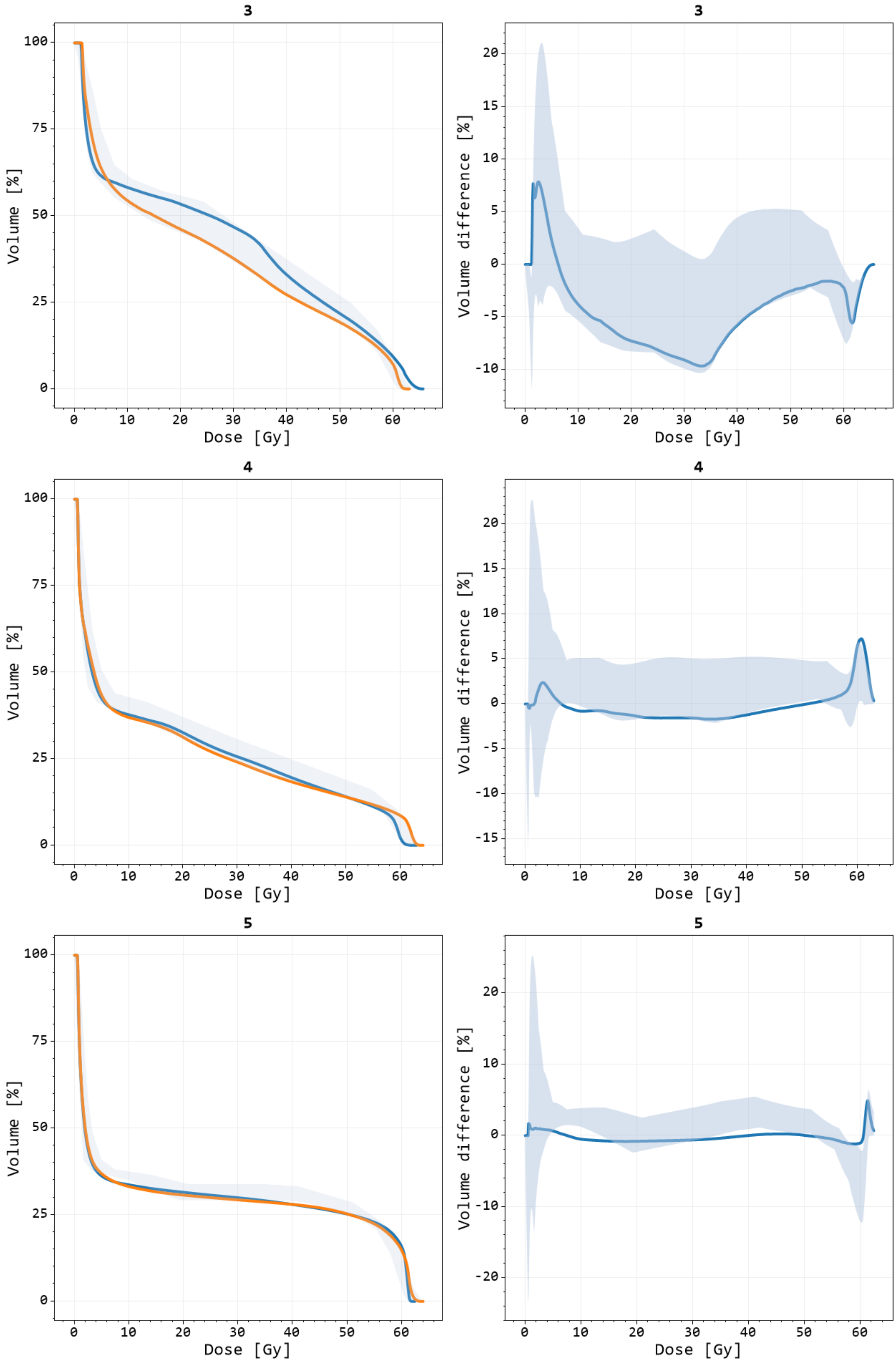
3
Volume [%]
Dose [Gy]
Volume difference [%]
4
Volume [%]
Dose [Gy]
Volume difference [%]
5
Volume [%]
Dose [Gy]
Volume difference [%]

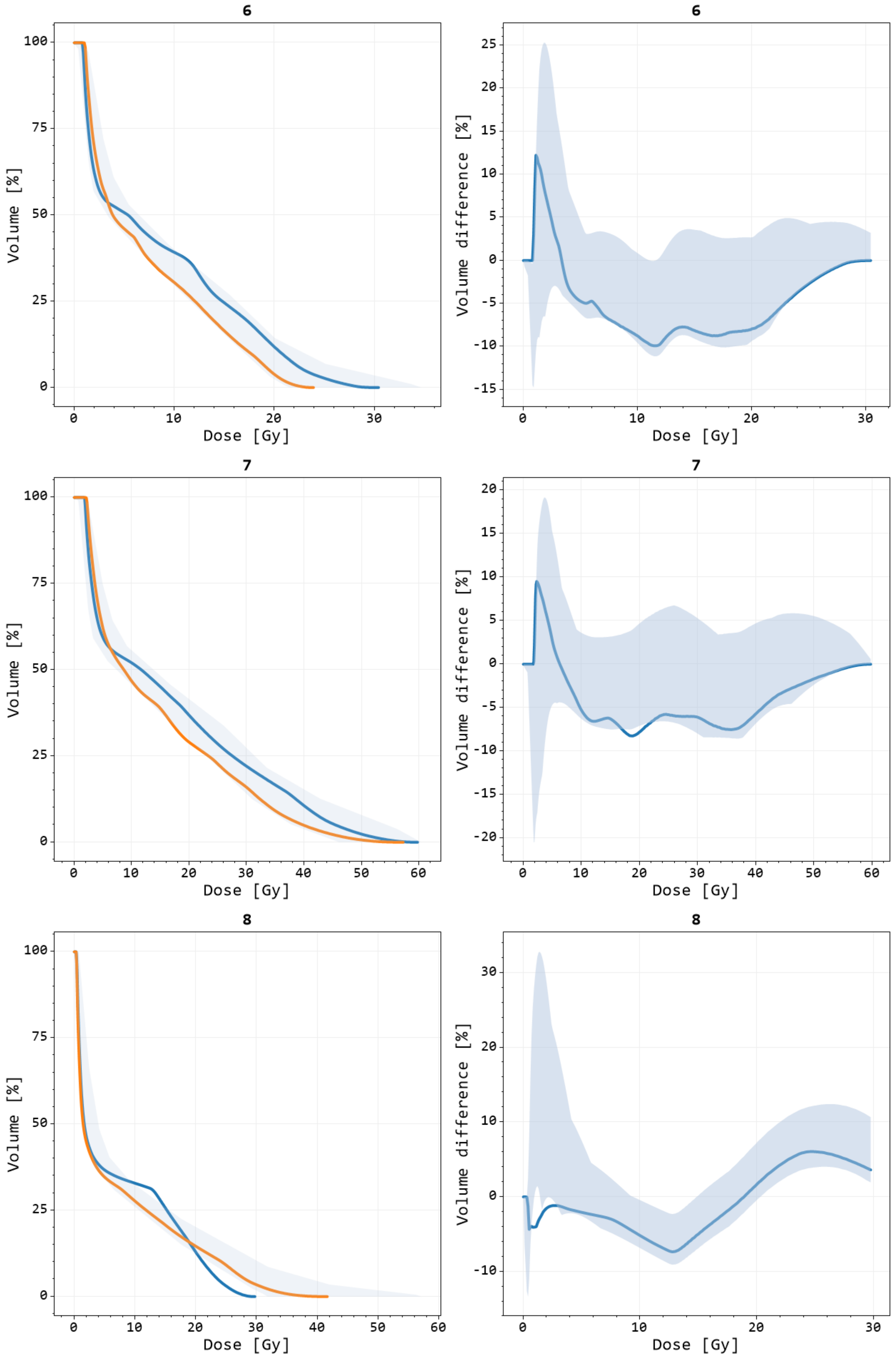
6
Volume [%]
Dose [Gy]
6
Volume difference [%]
Dose [Gy]
7
Volume [%]
Dose [Gy]
7
Volume difference [%]
Dose [Gy]
8
Volume [%]
Dose [Gy]
8
Volume difference [%]
Dose [Gy]

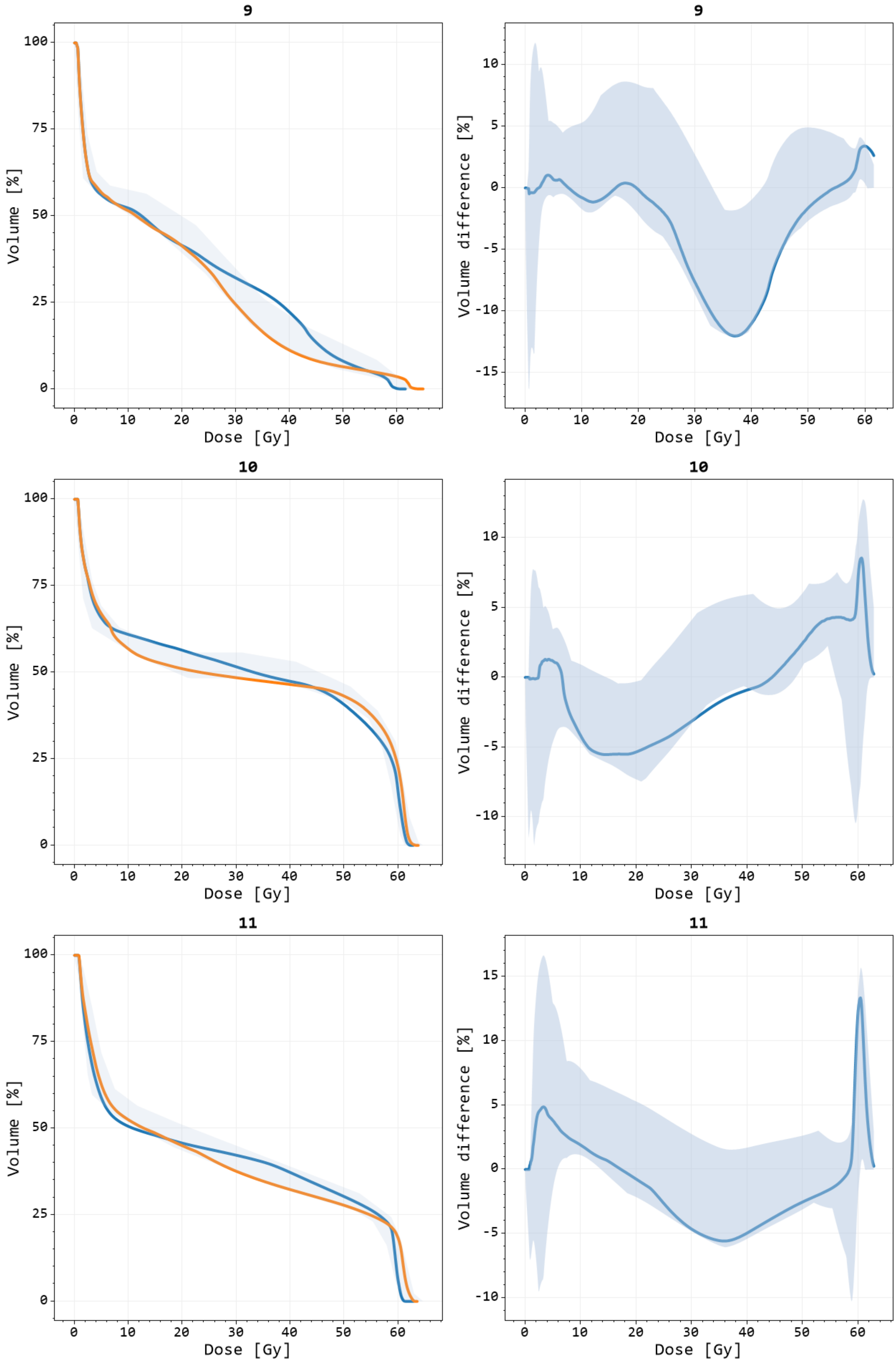
9
Volume [%]
Dose [Gy]
Volume difference [%]
10
Volume [%]
Dose [Gy]
Volume difference [%]
11
Volume [%]
Dose [Gy]
Volume difference [%]

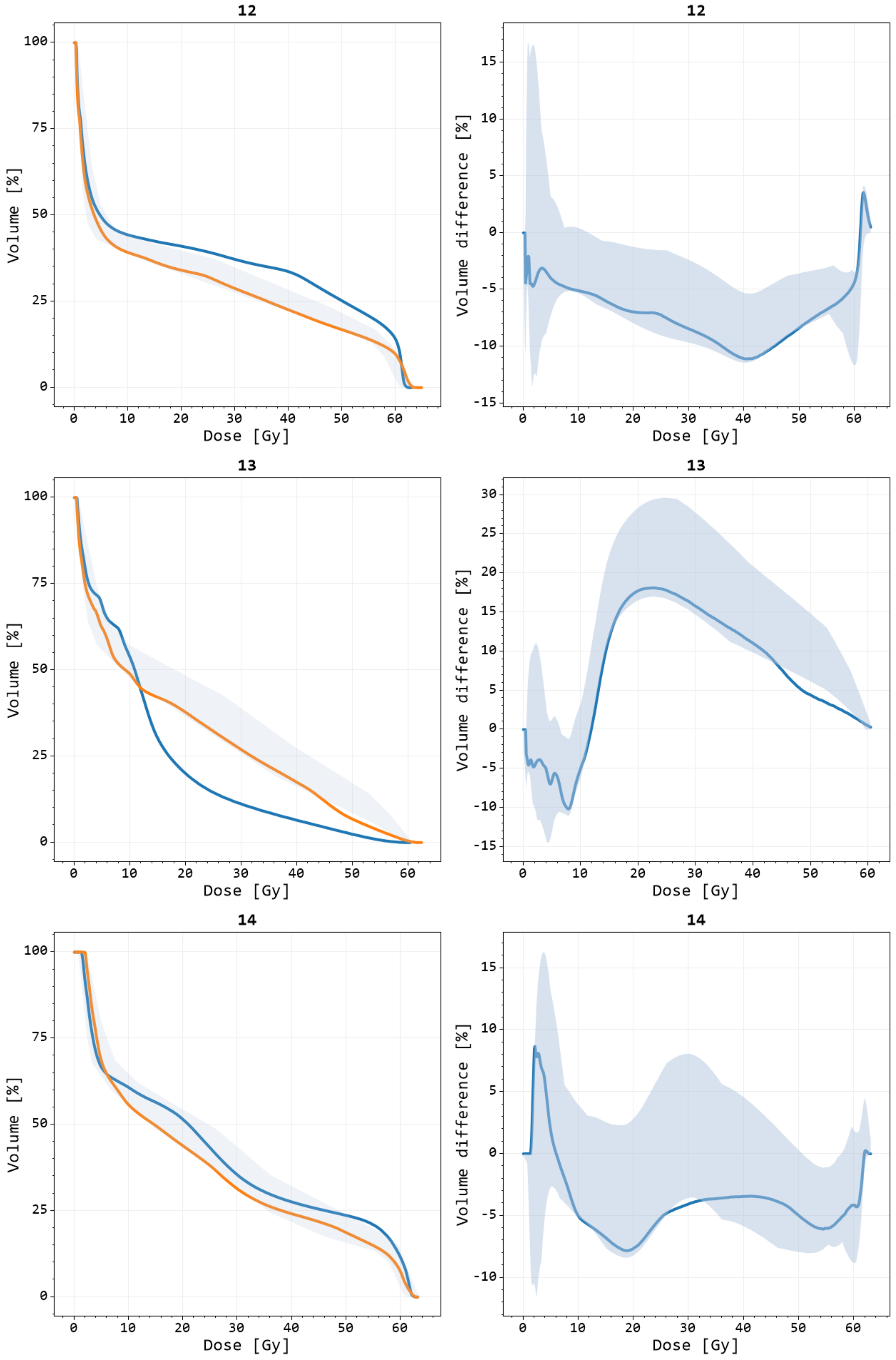

12
Volume [%]
Dose [Gy]
12
Volume difference [%]
Dose [Gy]
13
Volume [%]
Dose [Gy]
13
Volume difference [%]
Dose [Gy]
14
Volume [%]
Dose [Gy]
14
Volume difference [%]
Dose [Gy]

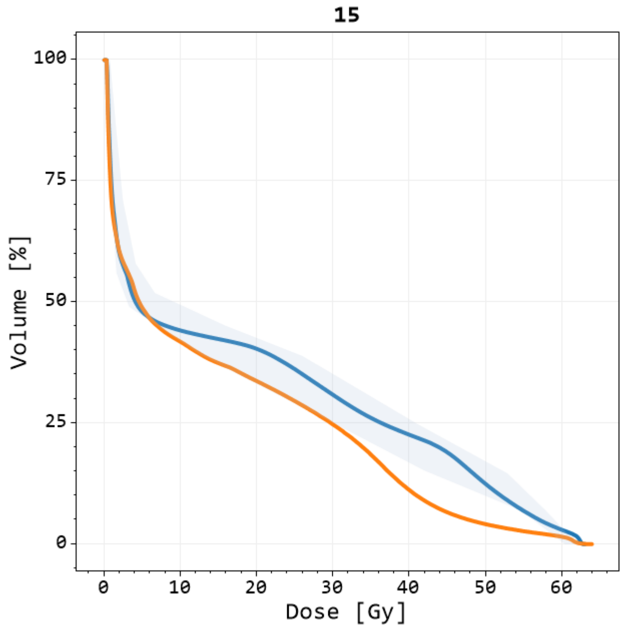
15
Volume [%]
Dose [Gy]

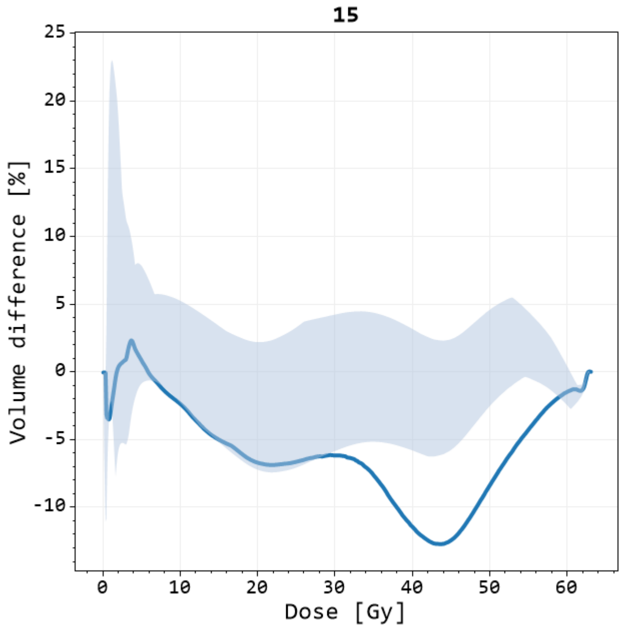
15
Volume difference [%]
Dose [Gy]

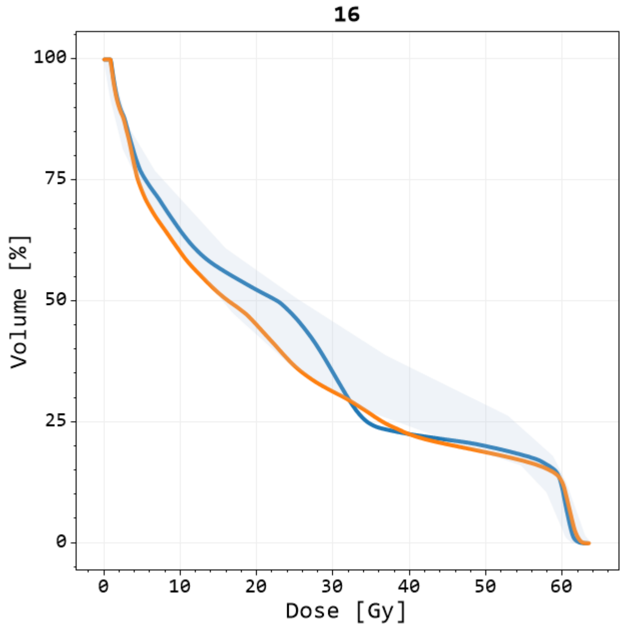
16
Volume [%]
Dose [Gy]

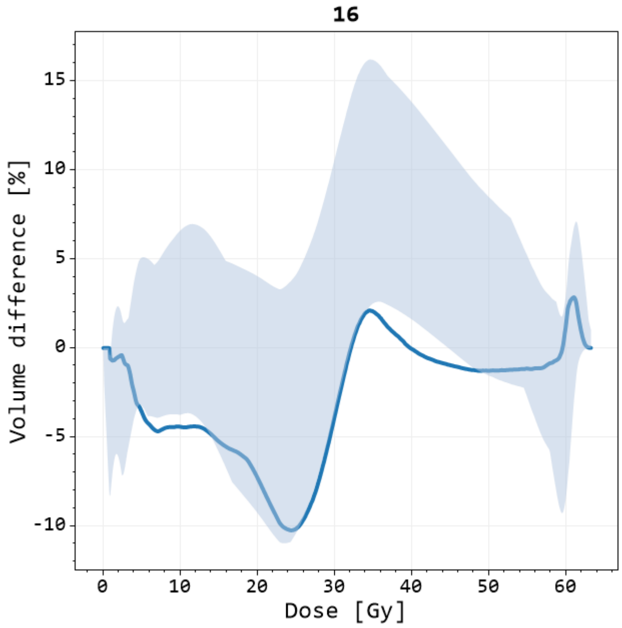
16
Volume difference [%]
Dose [Gy]

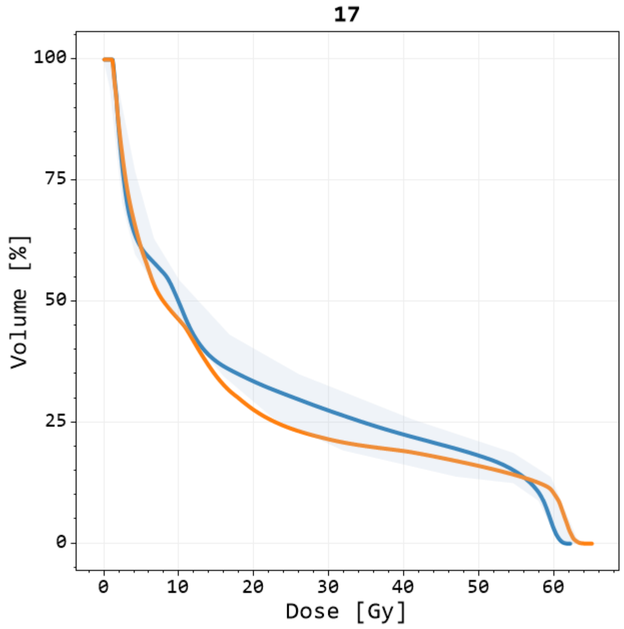
17
Volume [%]
Dose [Gy]

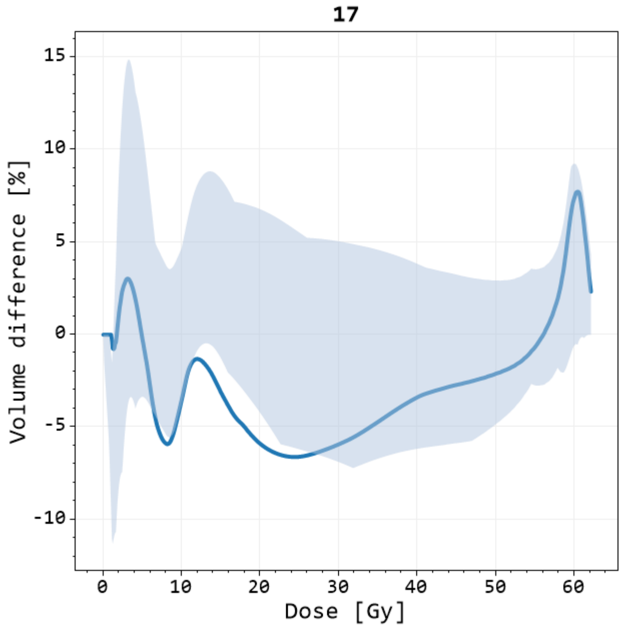
17
Volume difference [%]
Dose [Gy]

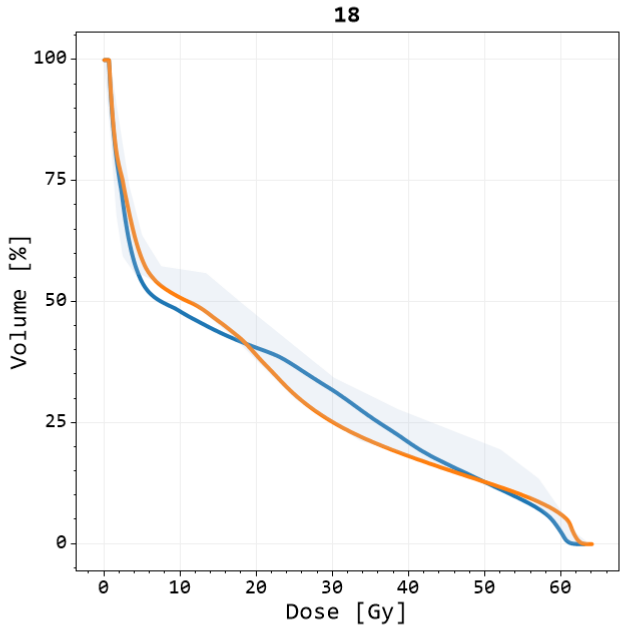
18
Volume [%]
Dose [Gy]
0
25
50
75
100
0
10
20
30
40
50
60

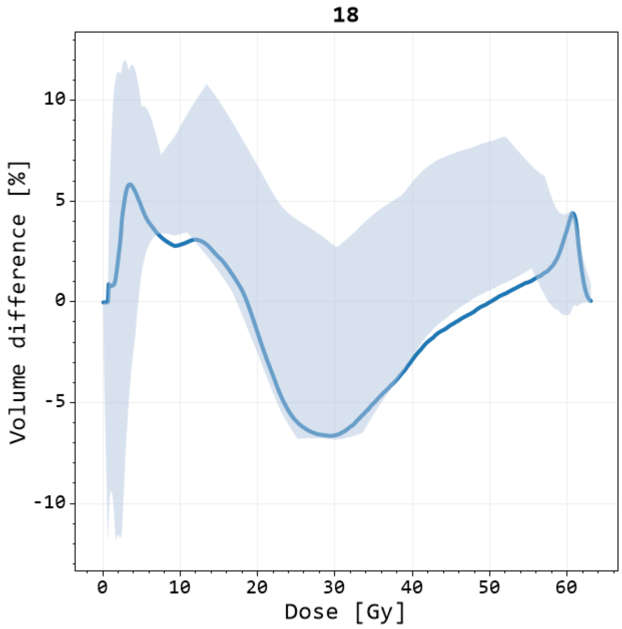
18
Volume difference [%]
Dose [Gy]
-10
-5
0
5
10
0
10
20
30
40
50
60

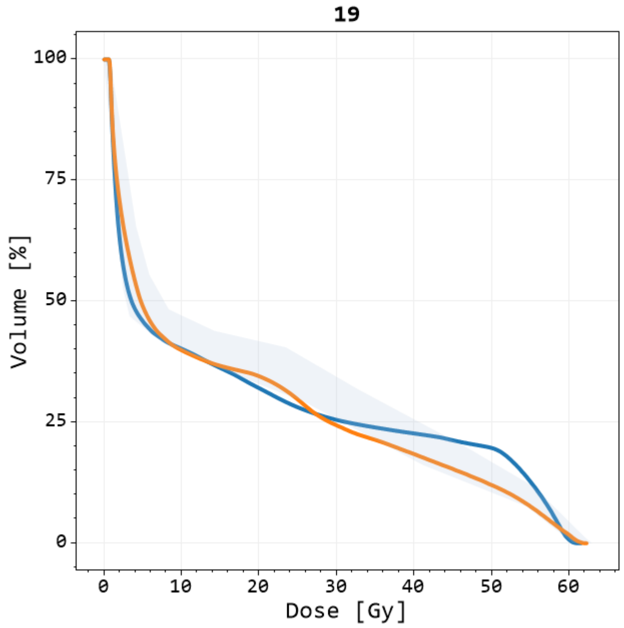
19
Volume [%]
Dose [Gy]
0
25
50
75
100
0
10
20
30
40
50
60

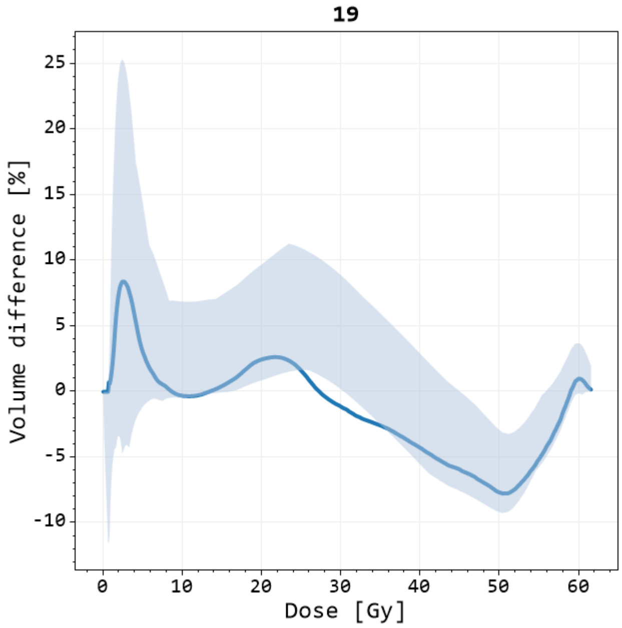
19
Volume difference [%]
Dose [Gy]
-10
-5
0
5
10
15
20
25
0
10
20
30
40
50
60

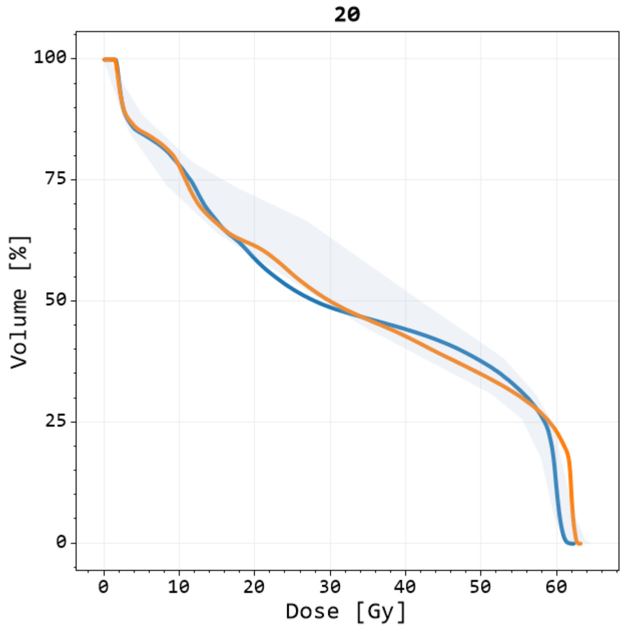
20
Volume [%]
Dose [Gy]
0
25
50
75
100
0
10
20
30
40
50
60

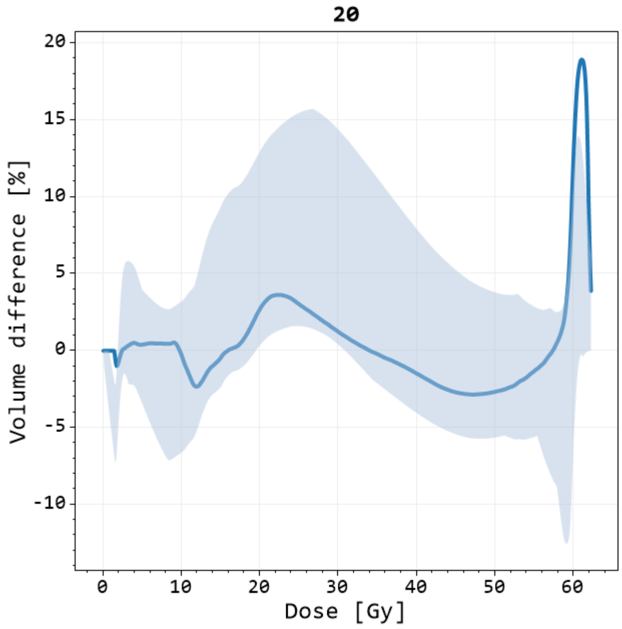
20
Volume difference [%]
Dose [Gy]
-10
-5
0
5
10
15
20
0
10
20
30
40
50
60

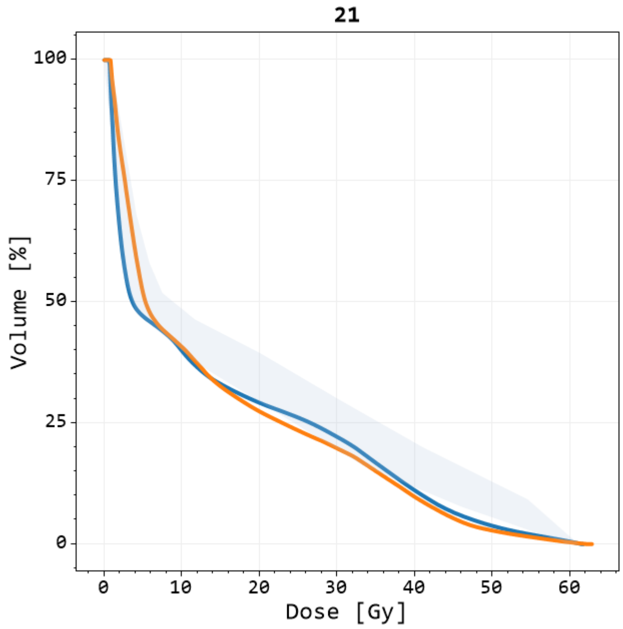
21
100
75
50
25
0
Volume [%]
0
10
20
30
40
50
60
Dose [Gy]

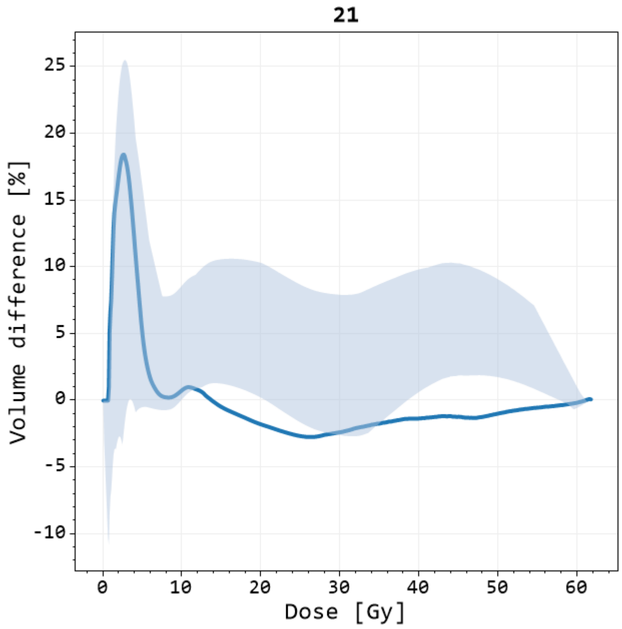
21
25
20
15
10
5
0
-5
-10
Volume difference [%]
0
10
20
30
40
50
60
Dose [Gy]

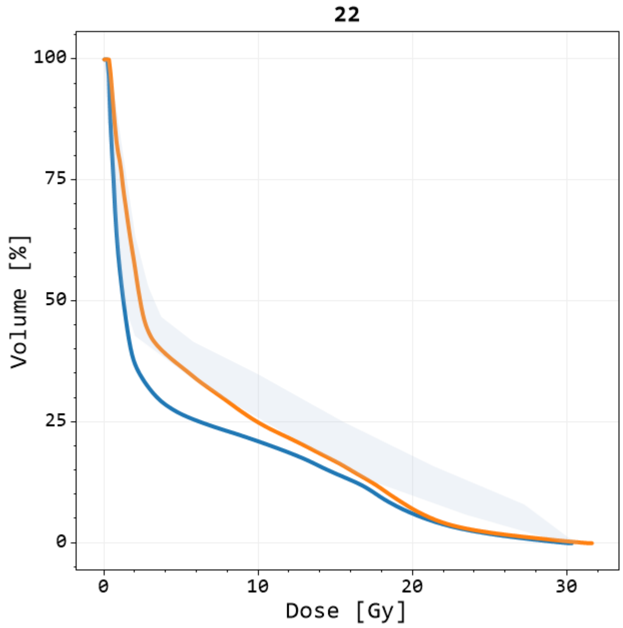
22
100
75
50
25
0
Volume [%]
0
10
20
30
Dose [Gy]

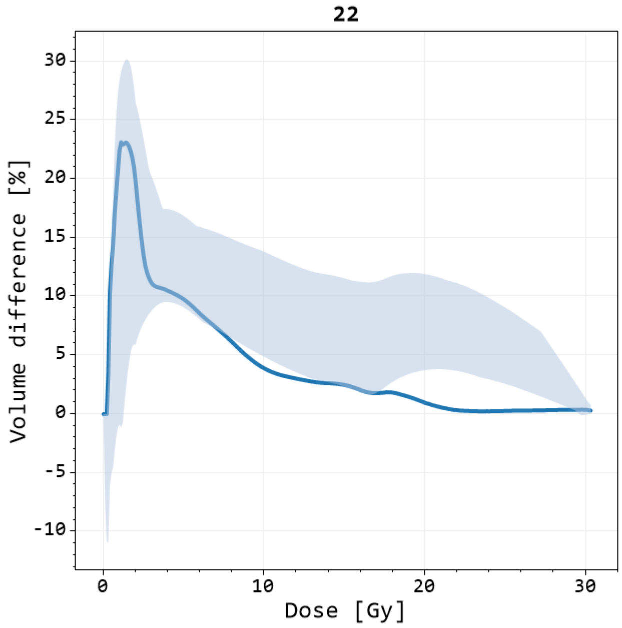
22
30
25
20
15
10
5
0
-5
-10
Volume difference [%]
0
10
20
30
Dose [Gy]

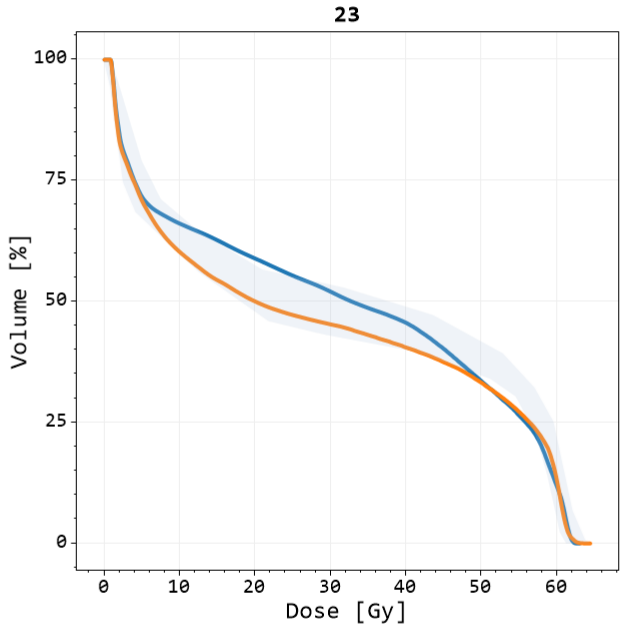
23
100
75
50
25
0
Volume [%]
0
10
20
30
40
50
60
Dose [Gy]

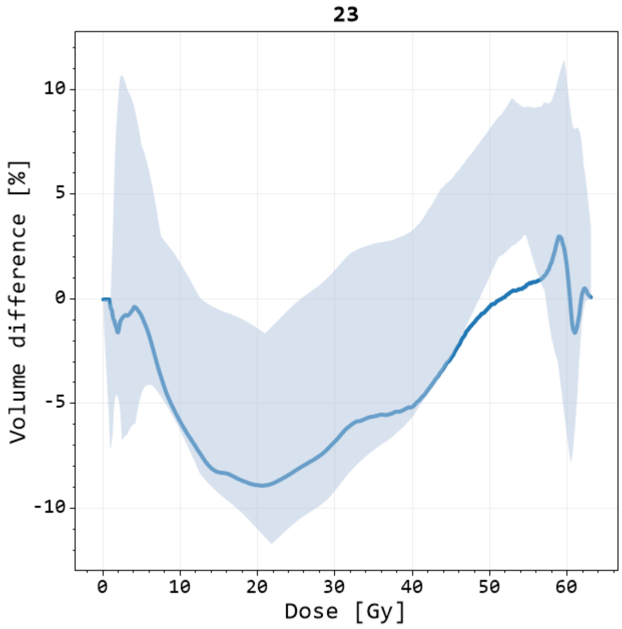
23
10
5
0
-5
-10
Volume difference [%]
0
10
20
30
40
50
60
Dose [Gy]

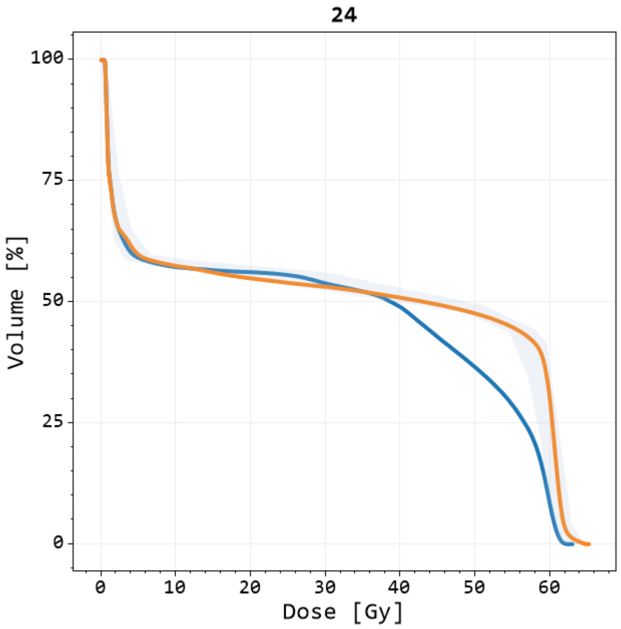
24
Volume [%]
Dose [Gy]
100
75
50
25
0
0 10 20 30 40 50 60

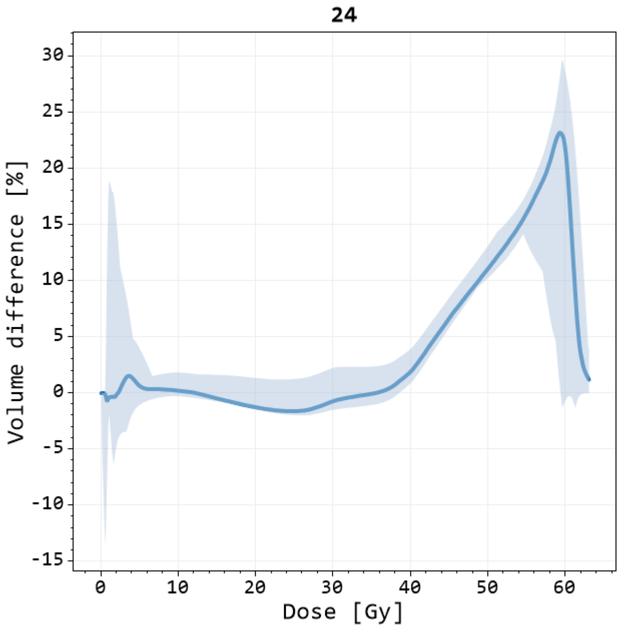
24
Volume difference [%]
Dose [Gy]
30
25
20
15
10
5
0
-5
-10
-15
0 10 20 30 40 50 60

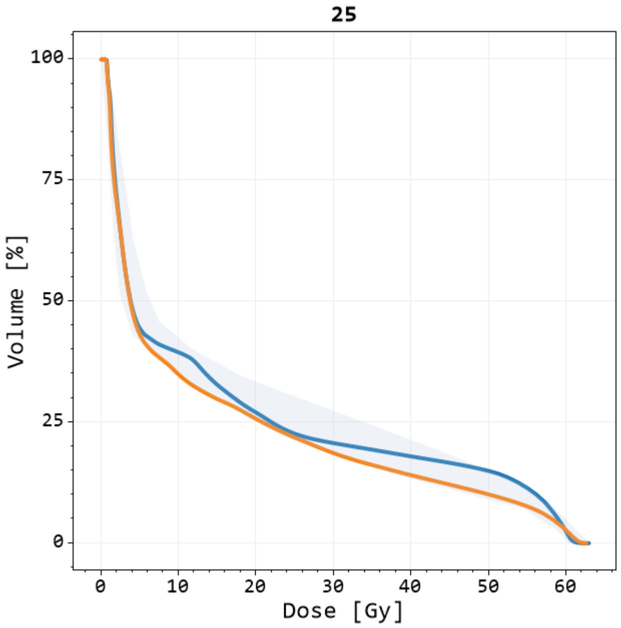
25
Volume [%]
Dose [Gy]
100
75
50
25
0
0 10 20 30 40 50 60

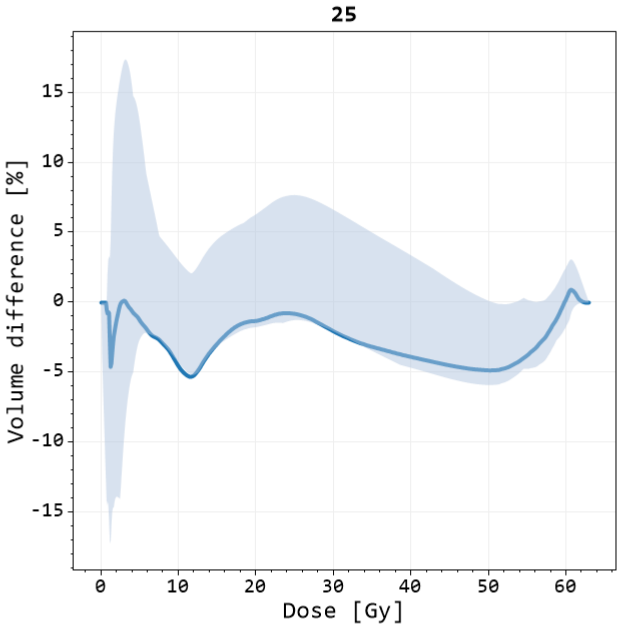
25
Volume difference [%]
Dose [Gy]
15
10
5
0
-5
-10
-15
0 10 20 30 40 50 60

# Heart

### DVH Volume difference

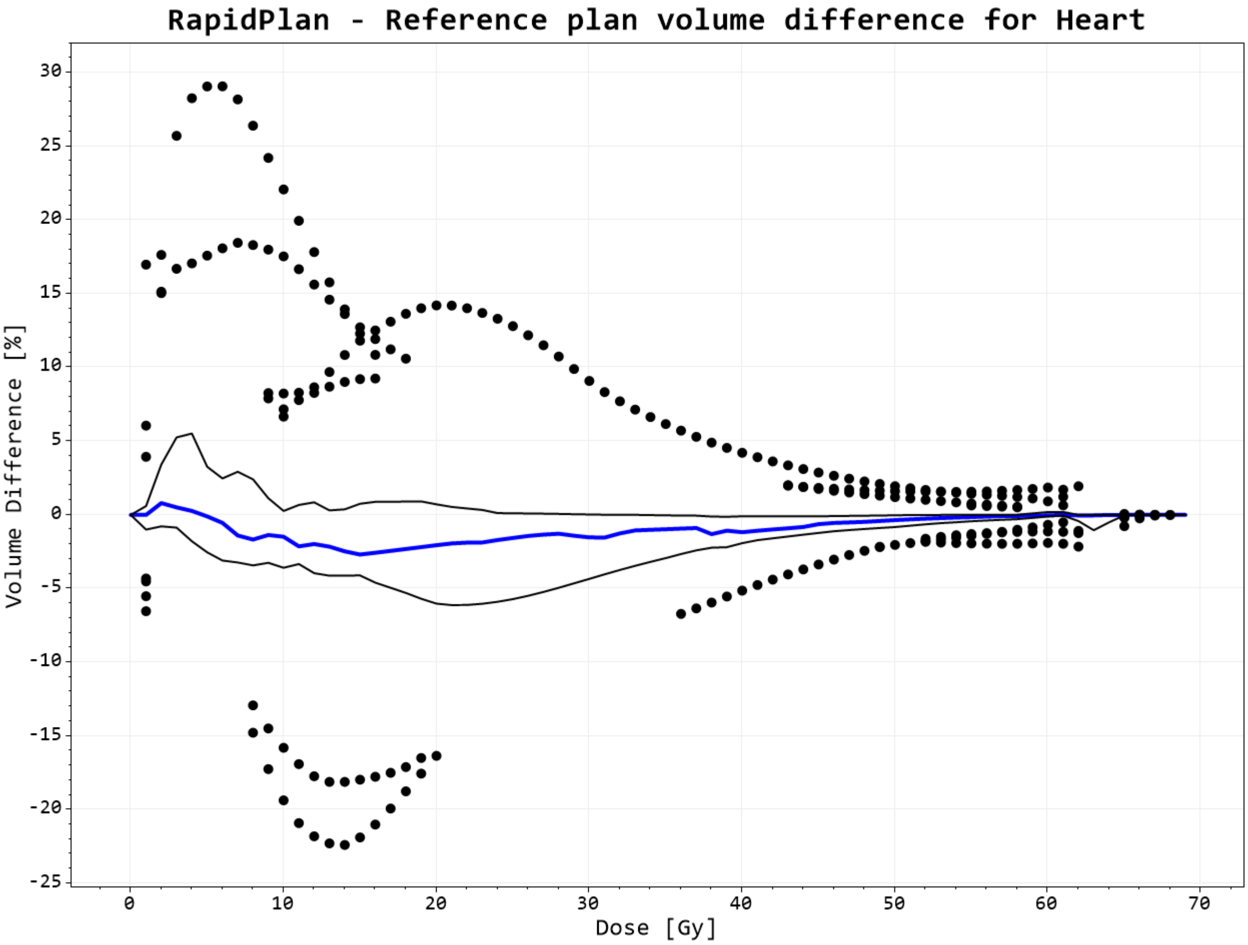


### Dose-volume metric summary table

| Metric | Reference Plan | RapidPlan | Difference |
|---|---|---|---|
| D46%[Gy] | 4.4 [1.9,9.9] | 4.4 [2.0,10.0] | 0.1 [-0.3,0.9] (p = 0.115) (3) |
| V30Gy[%] | 10.9 [6.6,17.0] | 8.9 [3.0,16.4] | -1.5 [-4.3,0.0] (p = 0.998) (1) |
| DMean[Gy] | 11.7 [6.6,14.4] | 9.7 [5.6,14.4] | -0.6 [-1.4,0.3] (p = 0.998) (5) |

## Dose-volume metric box whisker plots

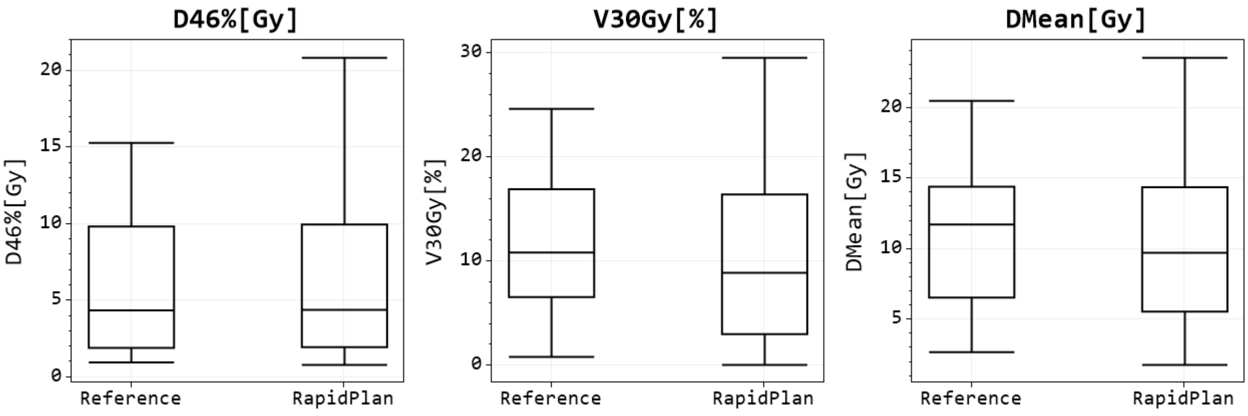

## Dose-volume metric differences by plan

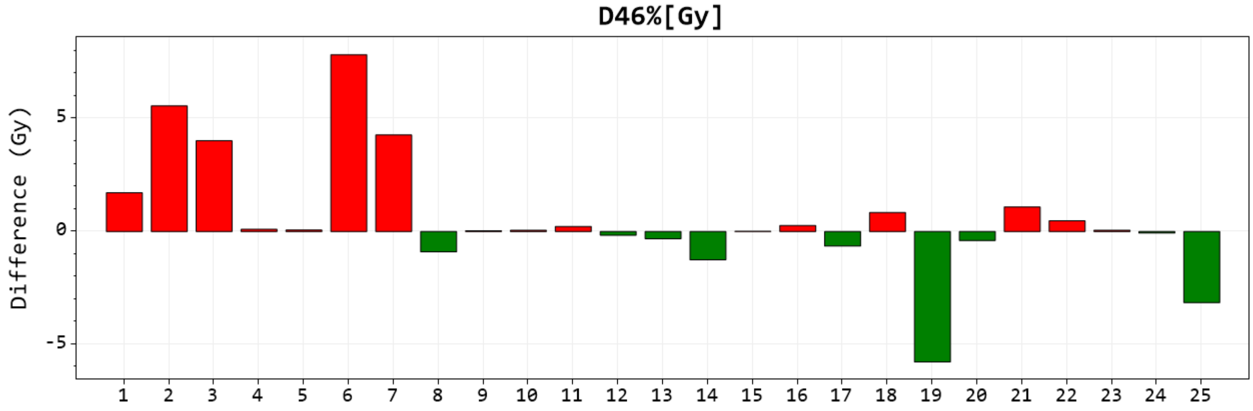


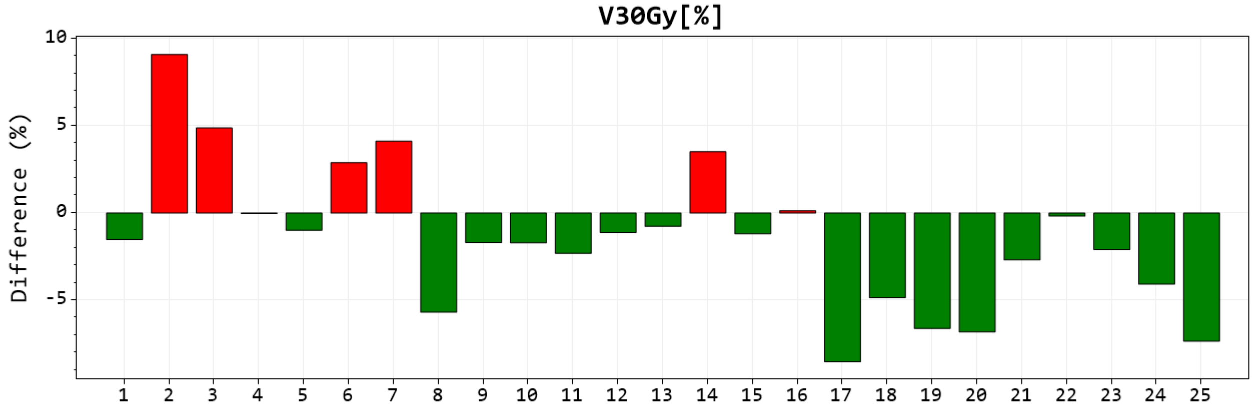


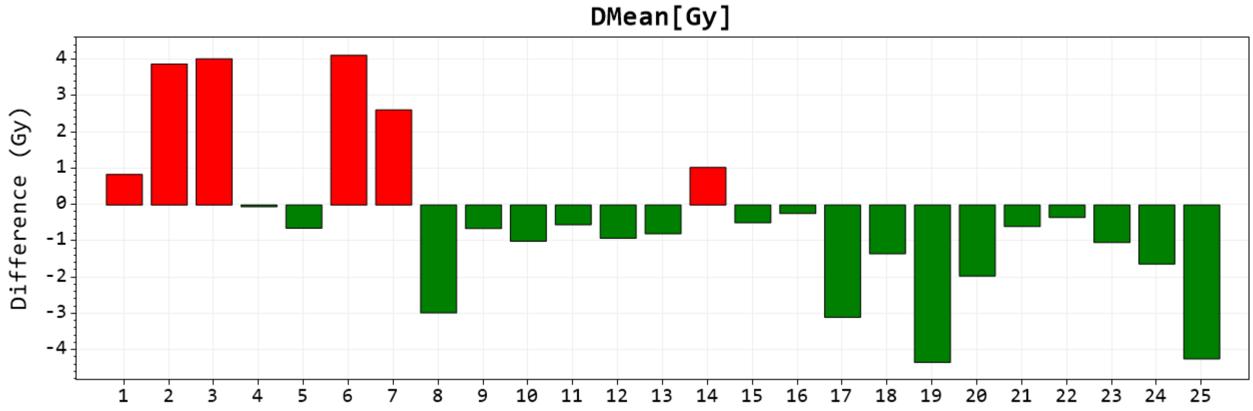

## Dose-volume histograms

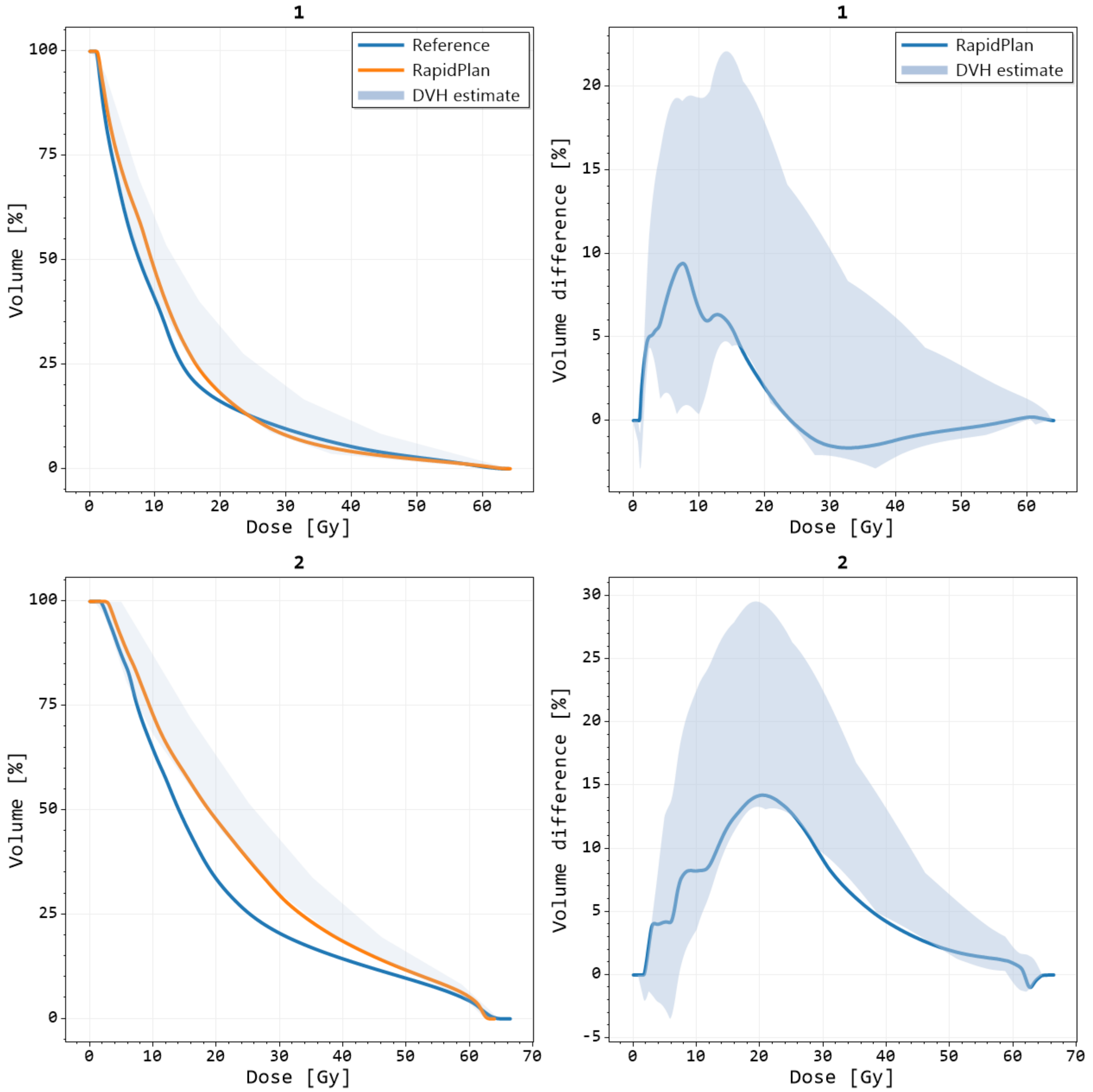

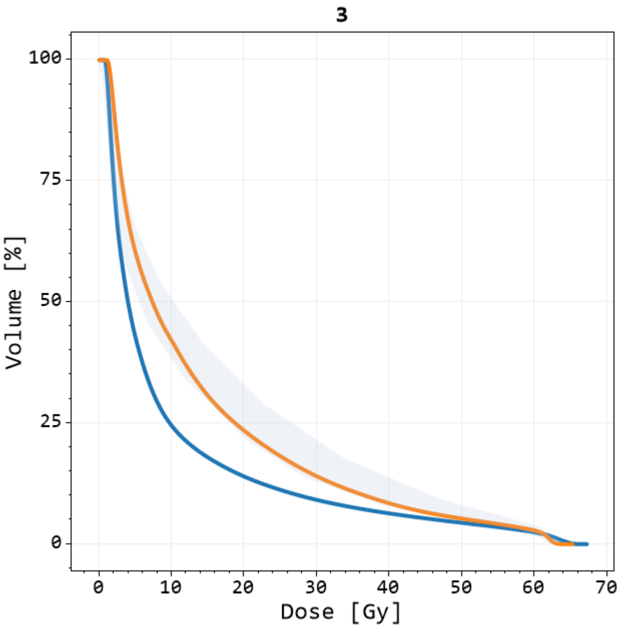
3
100
75
50
25
0
Volume [%]
0 10 20 30 40 50 60 70
Dose [Gy]

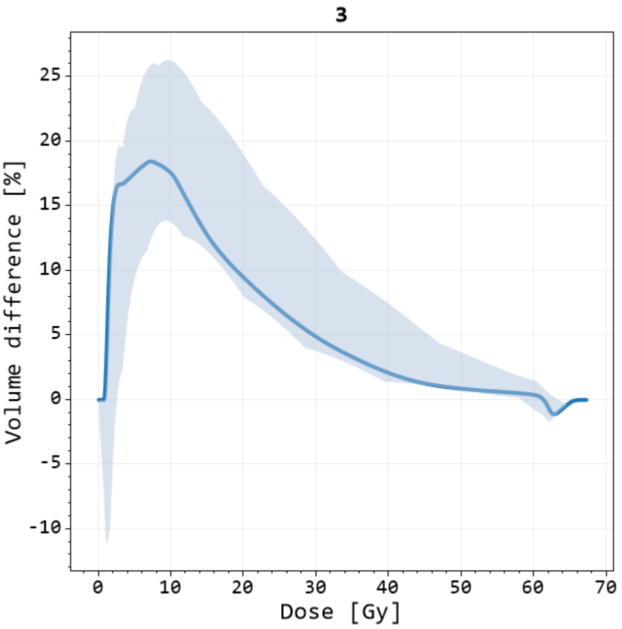
3
25
20
15
10
5
0
-5
-10
Volume difference [%]
0 10 20 30 40 50 60 70
Dose [Gy]

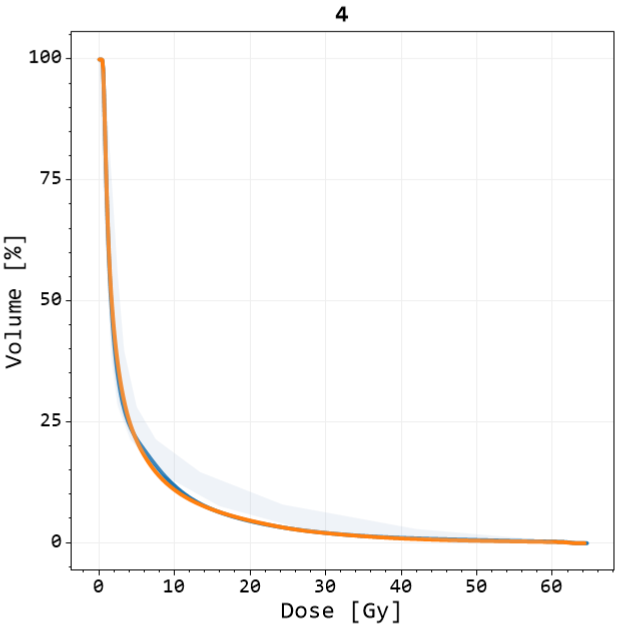
4
100
75
50
25
0
Volume [%]
0 10 20 30 40 50 60
Dose [Gy]

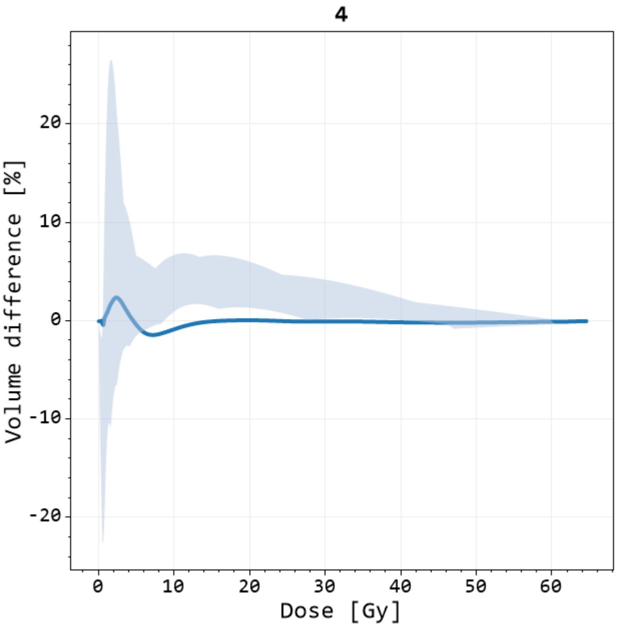
4
20
10
0
-10
-20
Volume difference [%]
0 10 20 30 40 50 60
Dose [Gy]

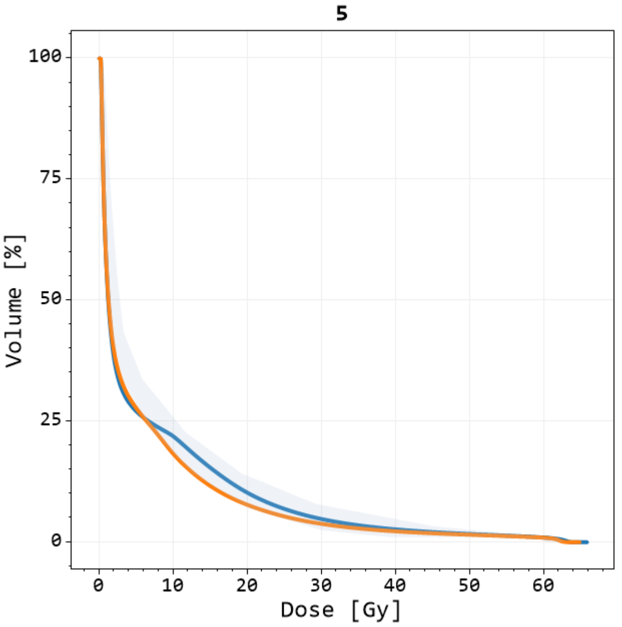
5
100
75
50
25
0
Volume [%]
0 10 20 30 40 50 60
Dose [Gy]

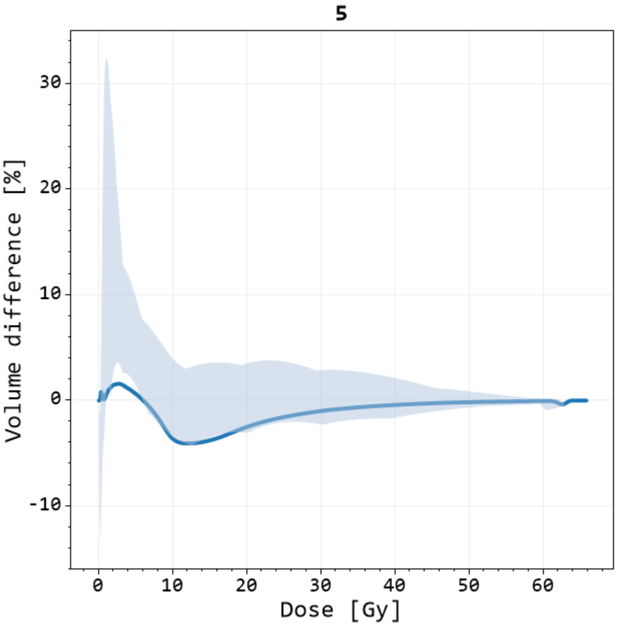
5
30
20
10
0
-10
Volume difference [%]
0 10 20 30 40 50 60
Dose [Gy]

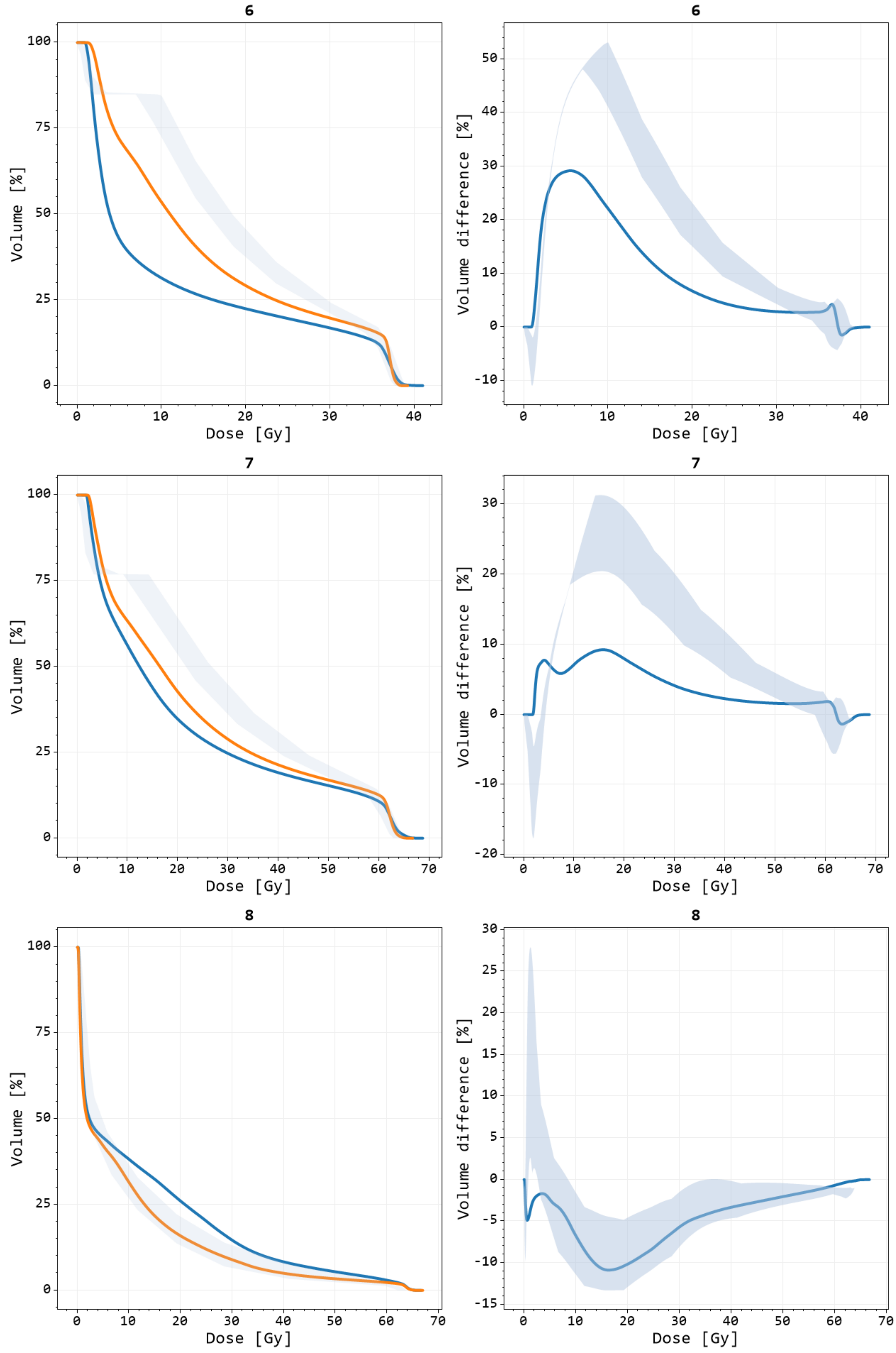
6
Volume [%]
Dose [Gy]
6
Volume difference [%]
Dose [Gy]
7
Volume [%]
Dose [Gy]
7
Volume difference [%]
Dose [Gy]
8
Volume [%]
Dose [Gy]
8
Volume difference [%]
Dose [Gy]

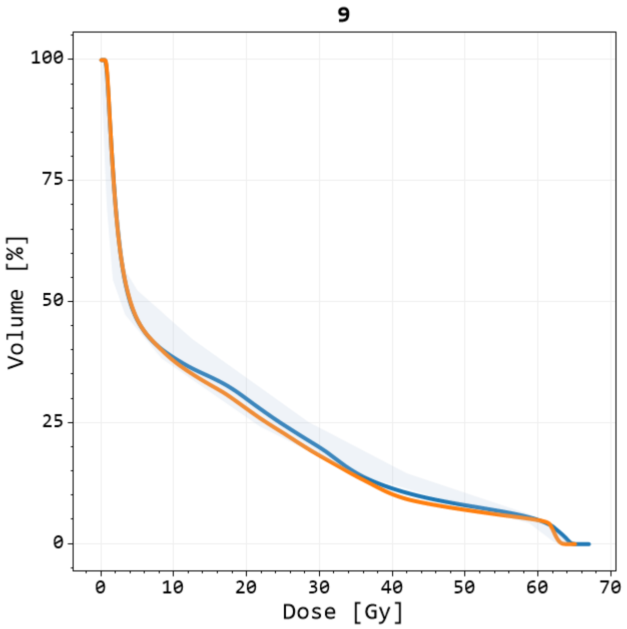
9
Volume [%]
Dose [Gy]

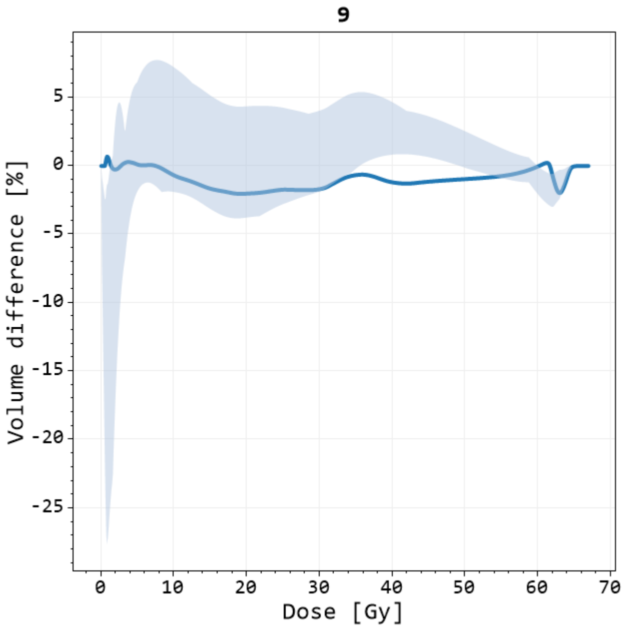
9
Volume difference [%]
Dose [Gy]

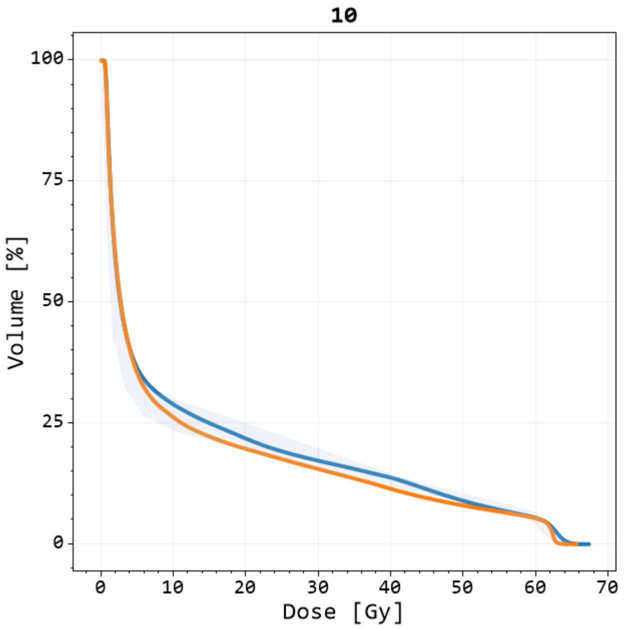
10
Volume [%]
Dose [Gy]

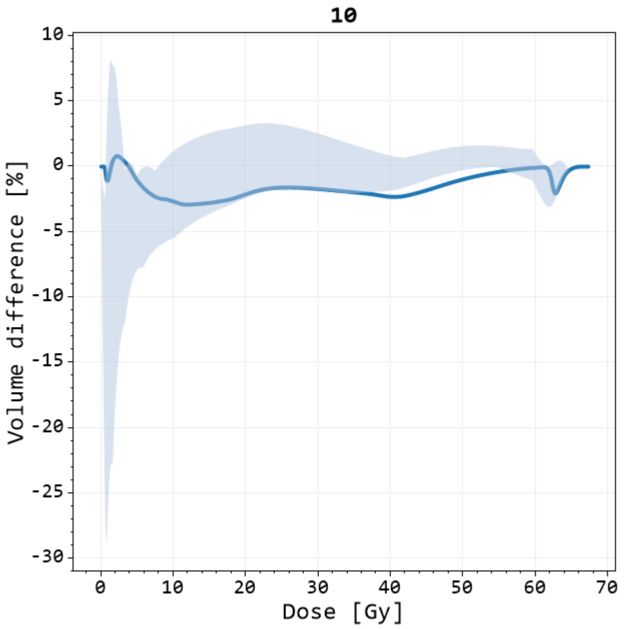
10
Volume difference [%]
Dose [Gy]

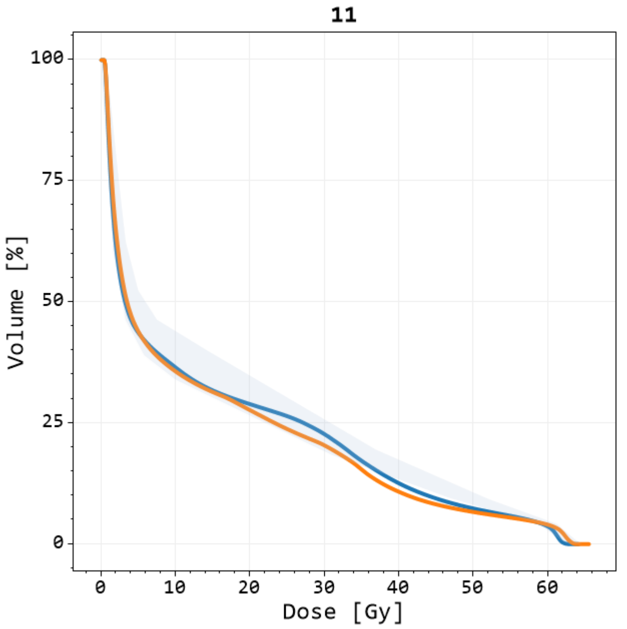
11
Volume [%]
Dose [Gy]

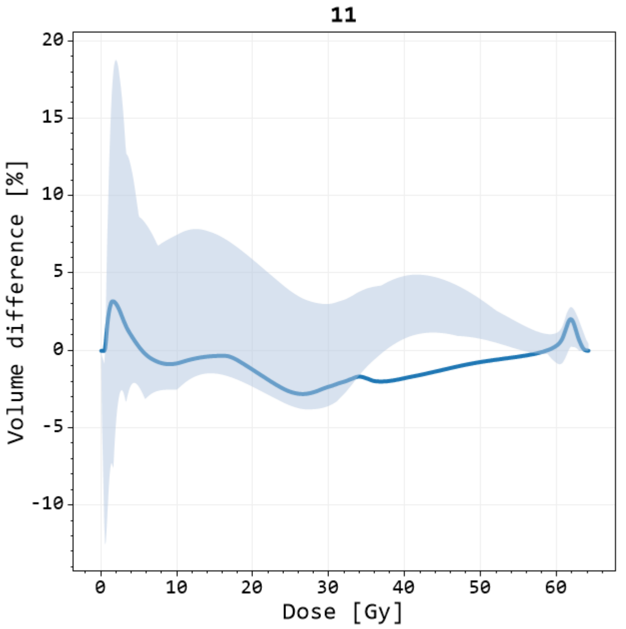
11
Volume difference [%]
Dose [Gy]

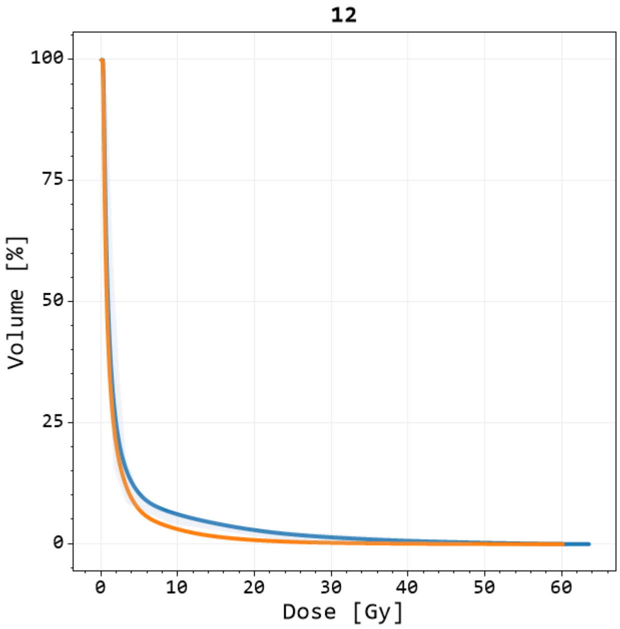
12
Volume [%]
Dose [Gy]

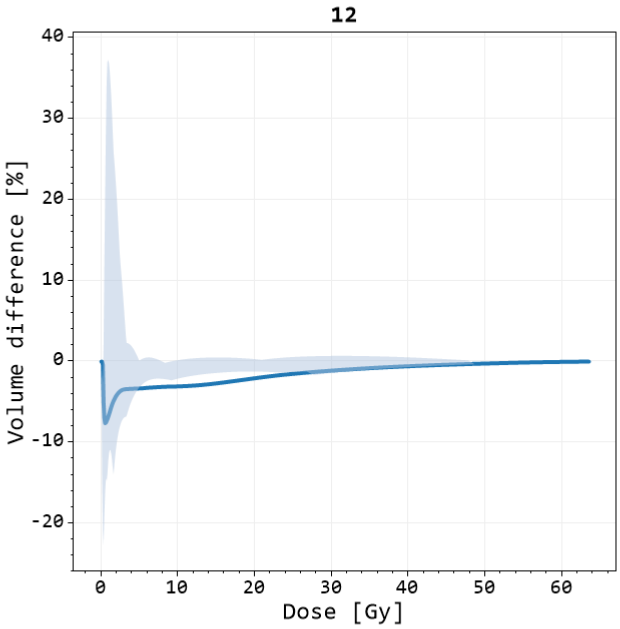
12
Volume difference [%]
Dose [Gy]

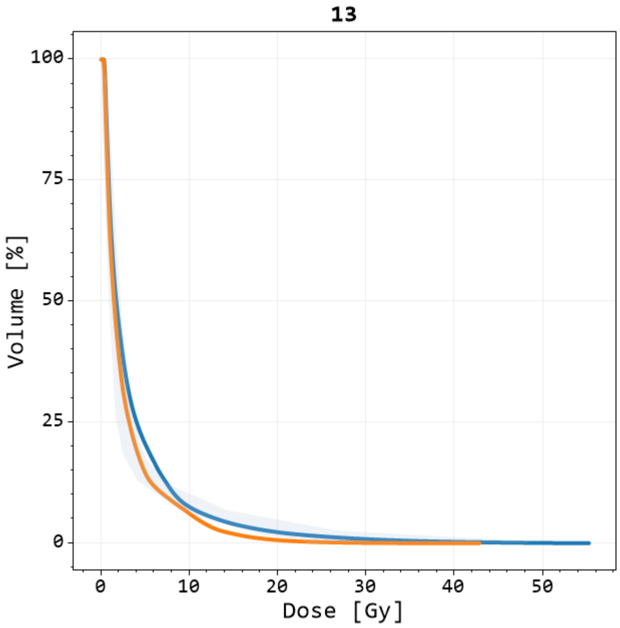
13
Volume [%]
Dose [Gy]

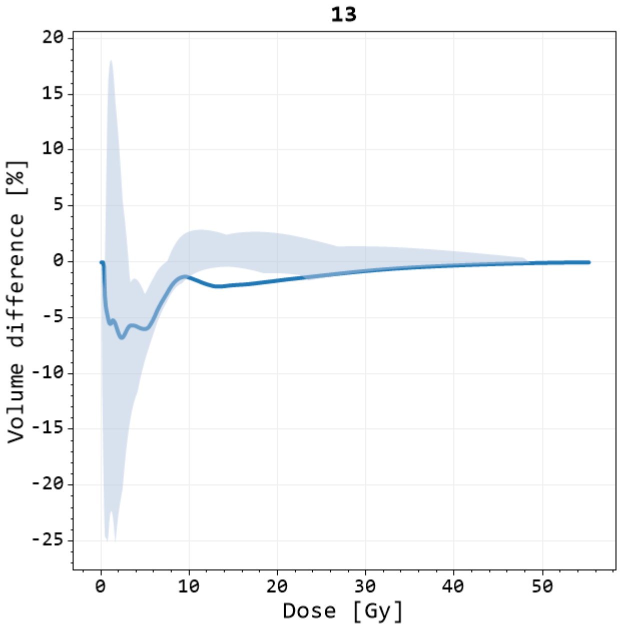
13
Volume difference [%]
Dose [Gy]

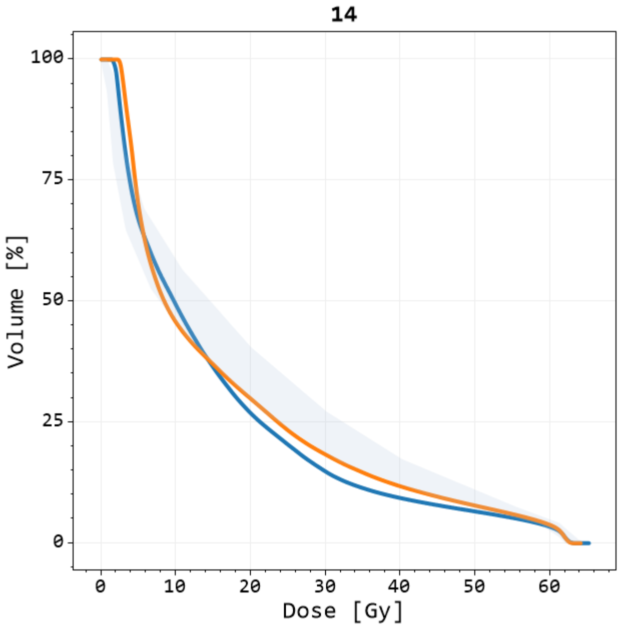
14
Volume [%]
Dose [Gy]

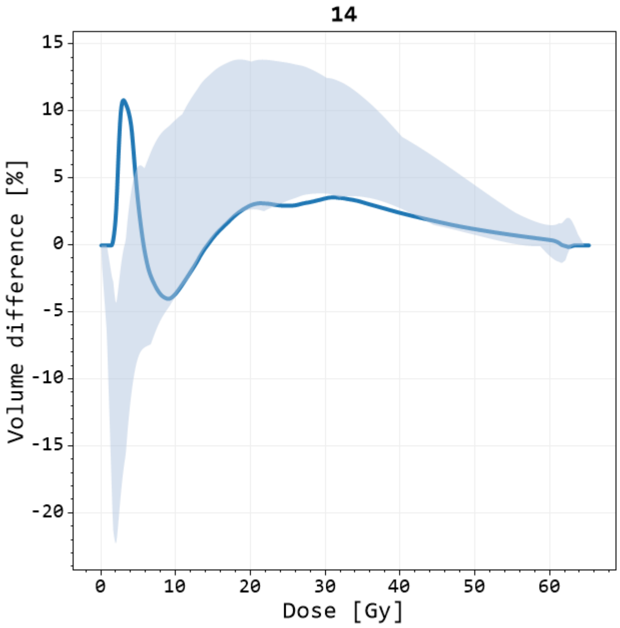
14
Volume difference [%]
Dose [Gy]

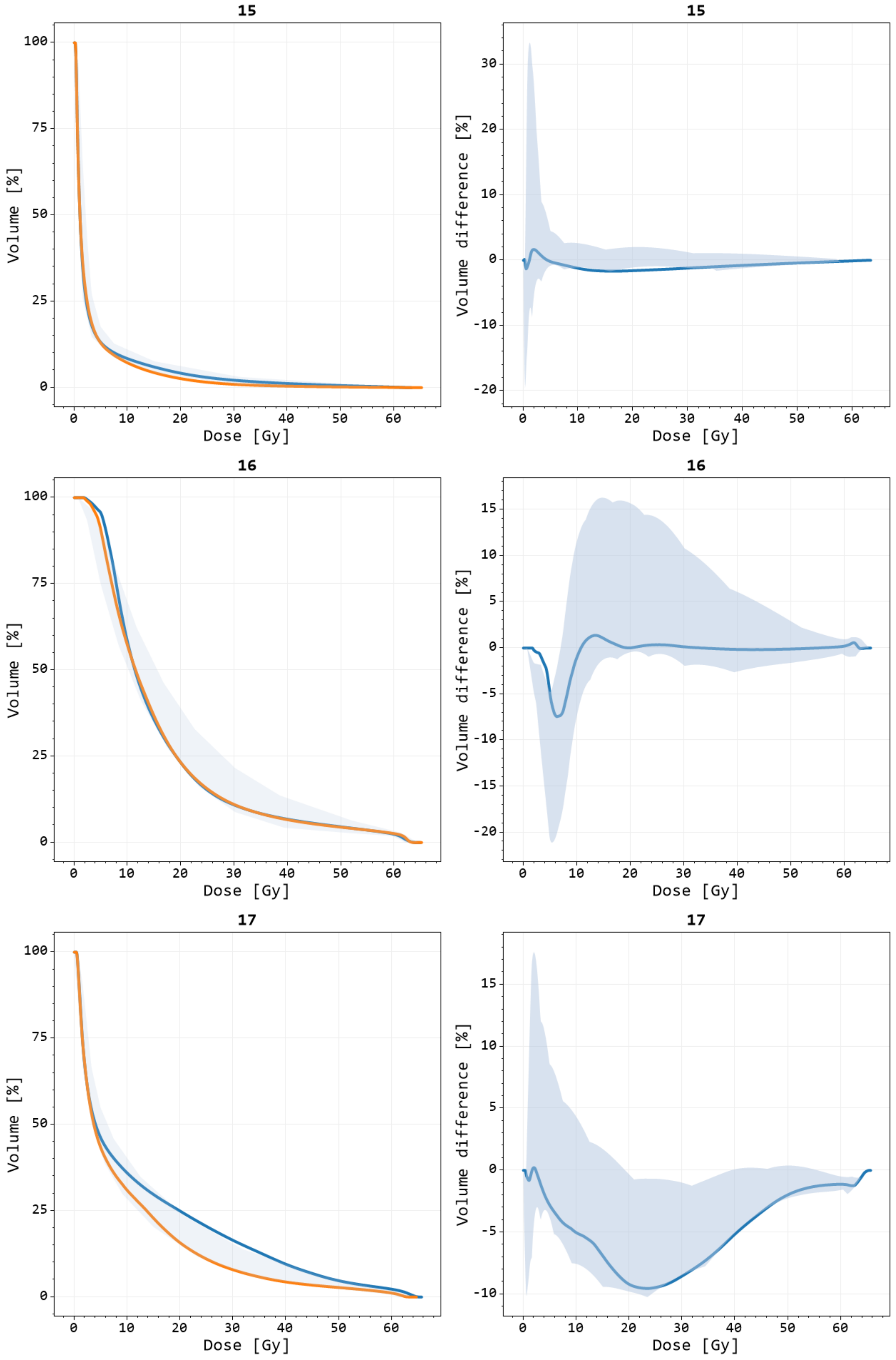
15
Volume [%]
Dose [Gy]
15
Volume difference [%]
Dose [Gy]
16
Volume [%]
Dose [Gy]
16
Volume difference [%]
Dose [Gy]
17
Volume [%]
Dose [Gy]
17
Volume difference [%]
Dose [Gy]

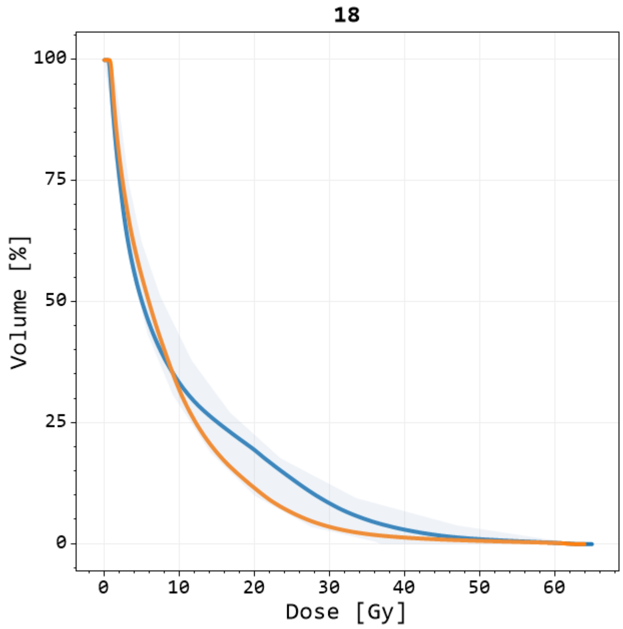
18
100
75
50
25
0
Volume [%]
0
10
20
30
40
50
60
Dose [Gy]

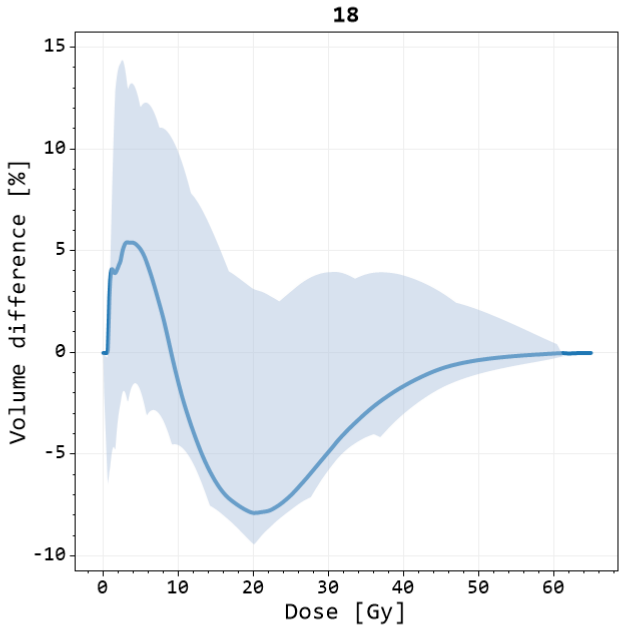
18
15
10
5
0
-5
-10
Volume difference [%]
0
10
20
30
40
50
60
Dose [Gy]

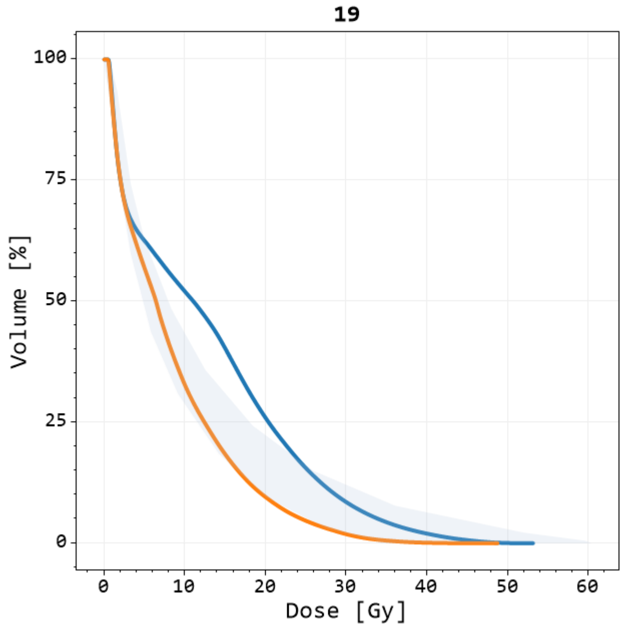
19
100
75
50
25
0
Volume [%]
0
10
20
30
40
50
60
Dose [Gy]

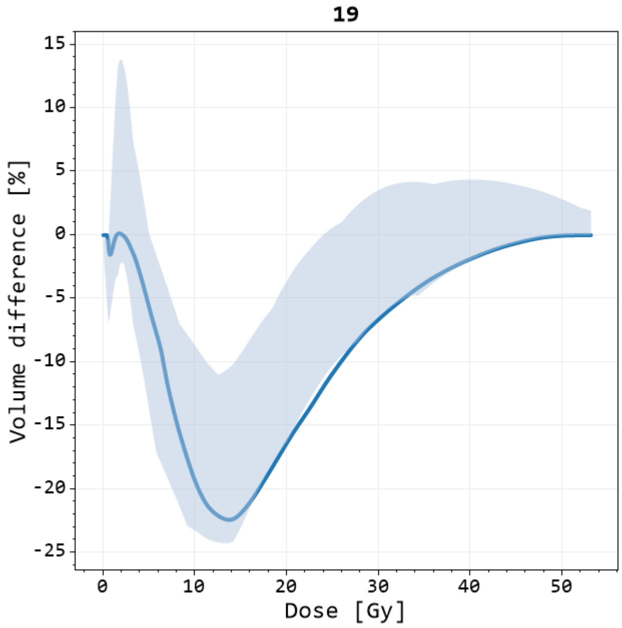
19
15
10
5
0
-5
-10
-15
-20
-25
Volume difference [%]
0
10
20
30
40
50
Dose [Gy]

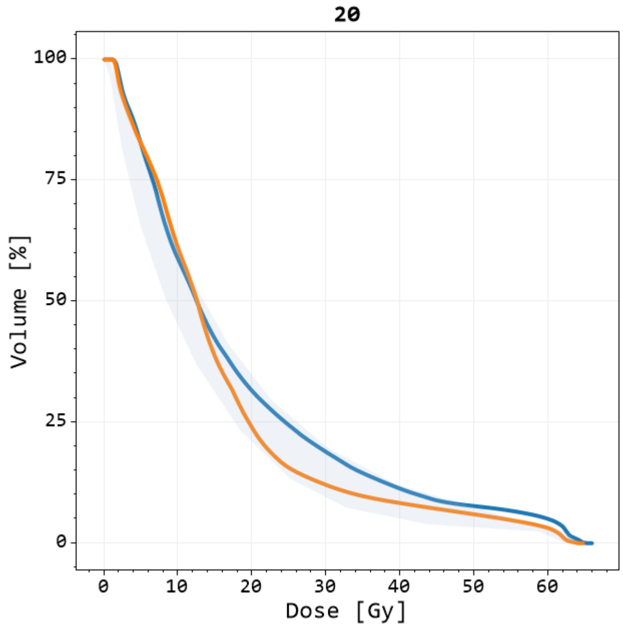
20
100
75
50
25
0
Volume [%]
0
10
20
30
40
50
60
Dose [Gy]

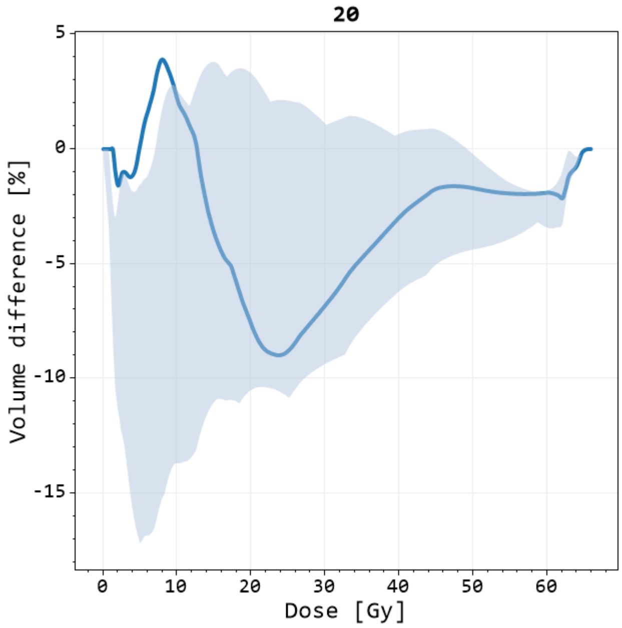
20
5
0
-5
-10
-15
Volume difference [%]
0
10
20
30
40
50
60
Dose [Gy]

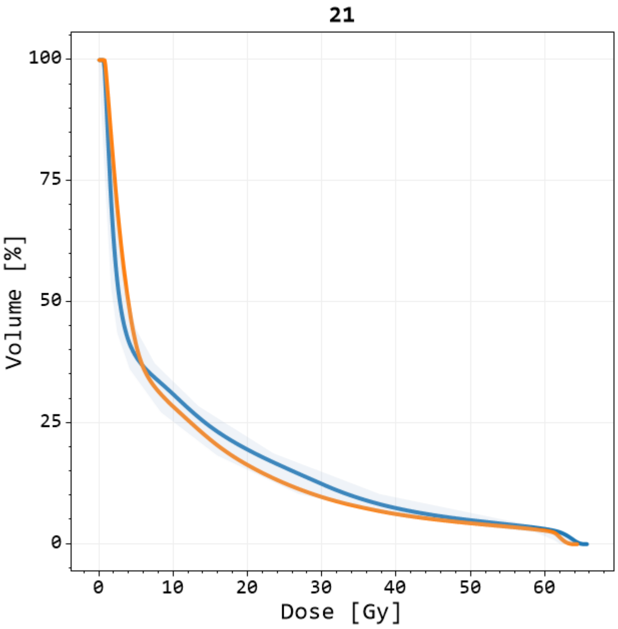
21
Volume [%]
Dose [Gy]
100
75
50
25
0
0 10 20 30 40 50 60

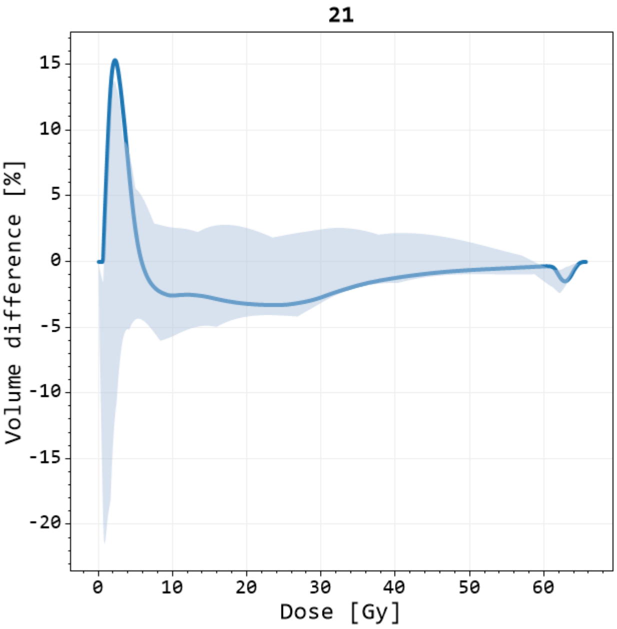
21
Volume difference [%]
Dose [Gy]
15
10
5
0
-5
-10
-15
-20
0 10 20 30 40 50 60

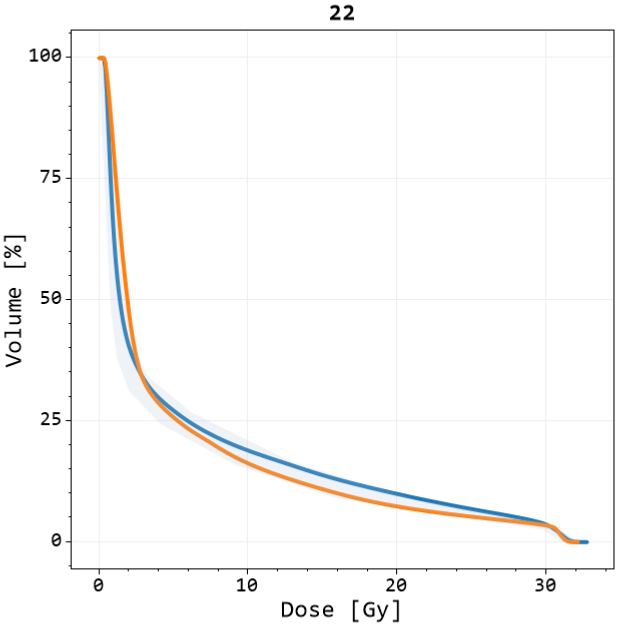
22
Volume [%]
Dose [Gy]
100
75
50
25
0
0 10 20 30

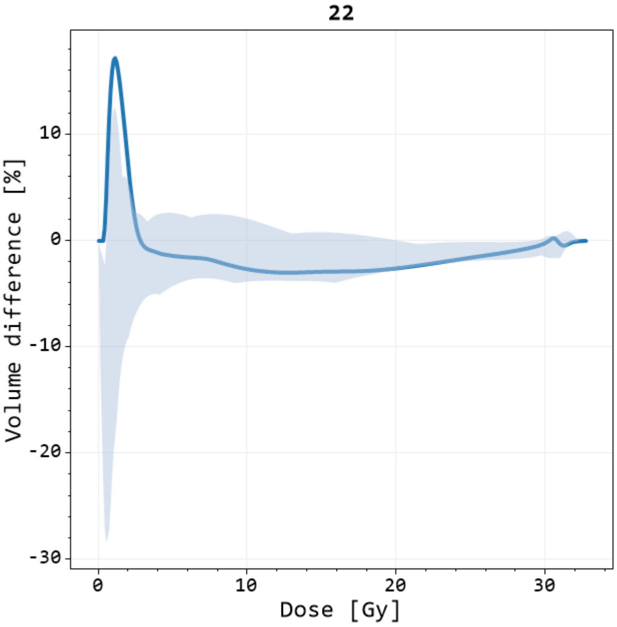
22
Volume difference [%]
Dose [Gy]
10
0
-10
-20
-30
0 10 20 30

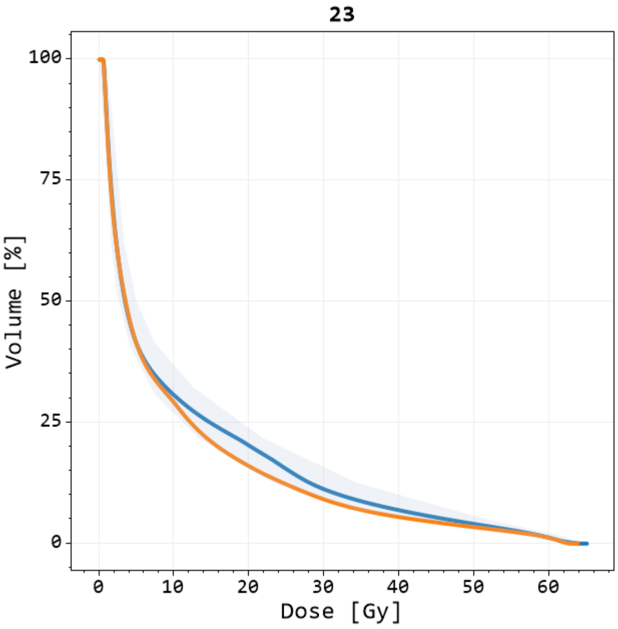
23
Volume [%]
Dose [Gy]
100
75
50
25
0
0 10 20 30 40 50 60

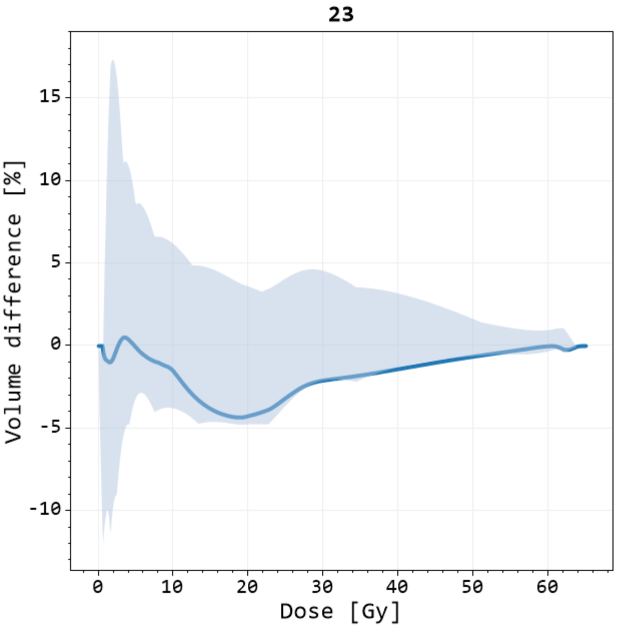
23
Volume difference [%]
Dose [Gy]
15
10
5
0
-5
-10
0 10 20 30 40 50 60

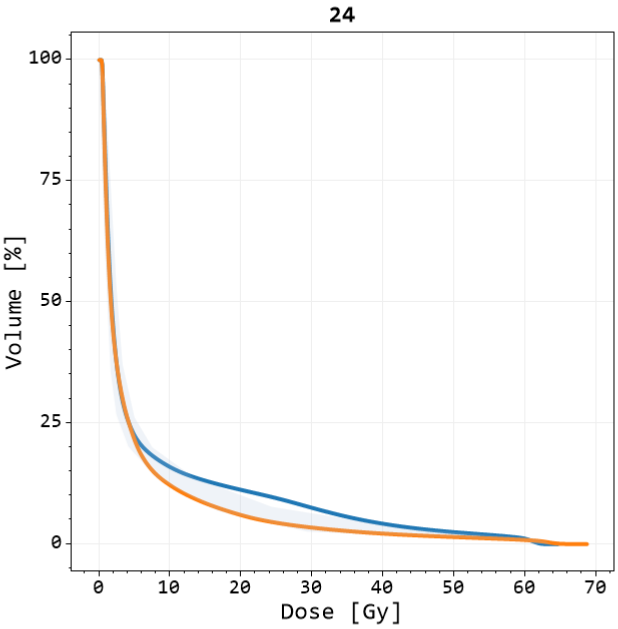
24
Volume [%]
Dose [Gy]
0
25
50
75
100
0 10 20 30 40 50 60 70

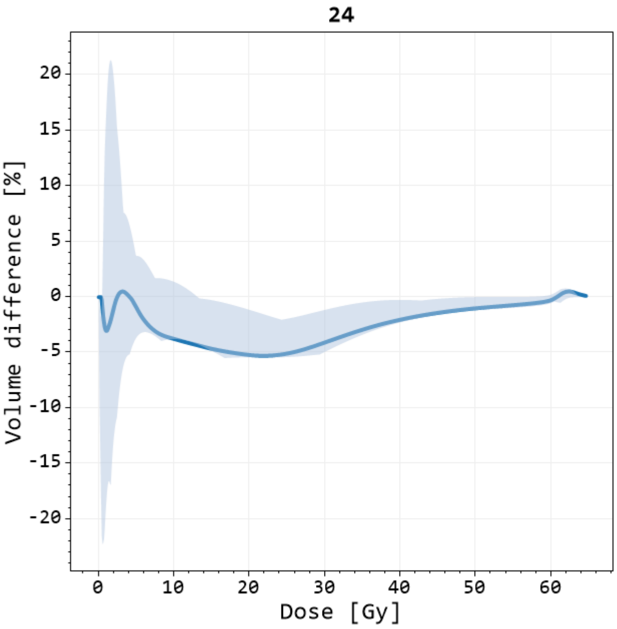
24
Volume difference [%]
Dose [Gy]
20
15
10
5
0
-5
-10
-15
-20
0 10 20 30 40 50 60

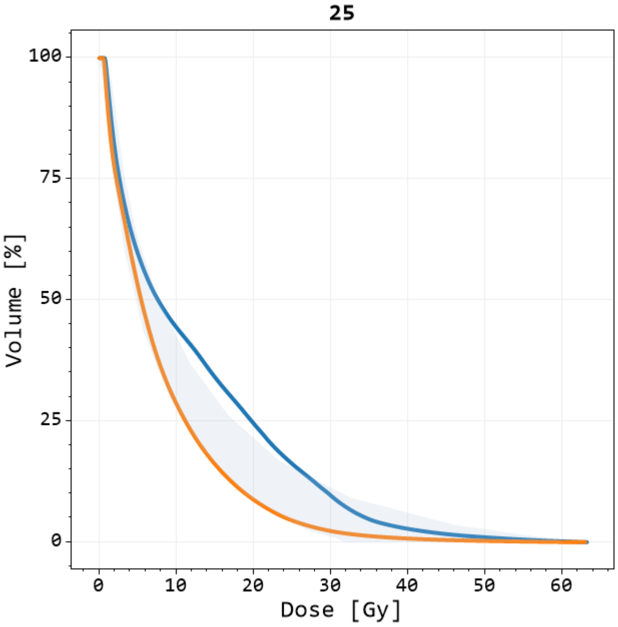
25
Volume [%]
Dose [Gy]
0
25
50
75
100
0 10 20 30 40 50 60

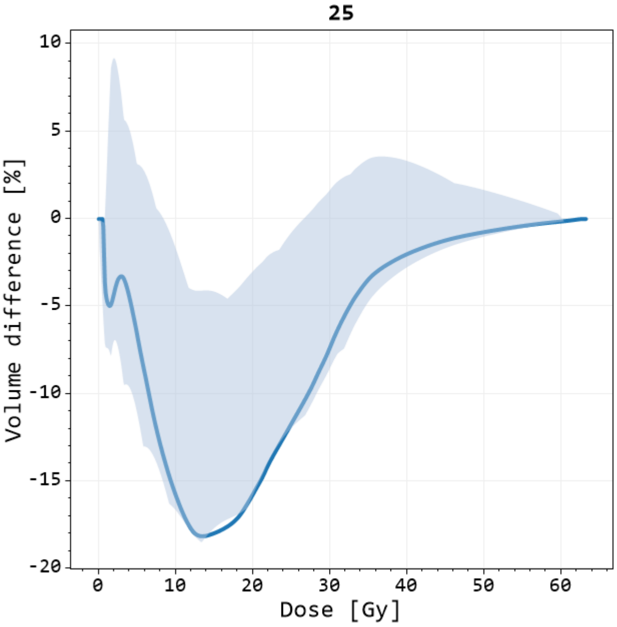
25
Volume difference [%]
Dose [Gy]
10
5
0
-5
-10
-15
-20
0 10 20 30 40 50 60

# Lung_Contra

## DVH Volume difference

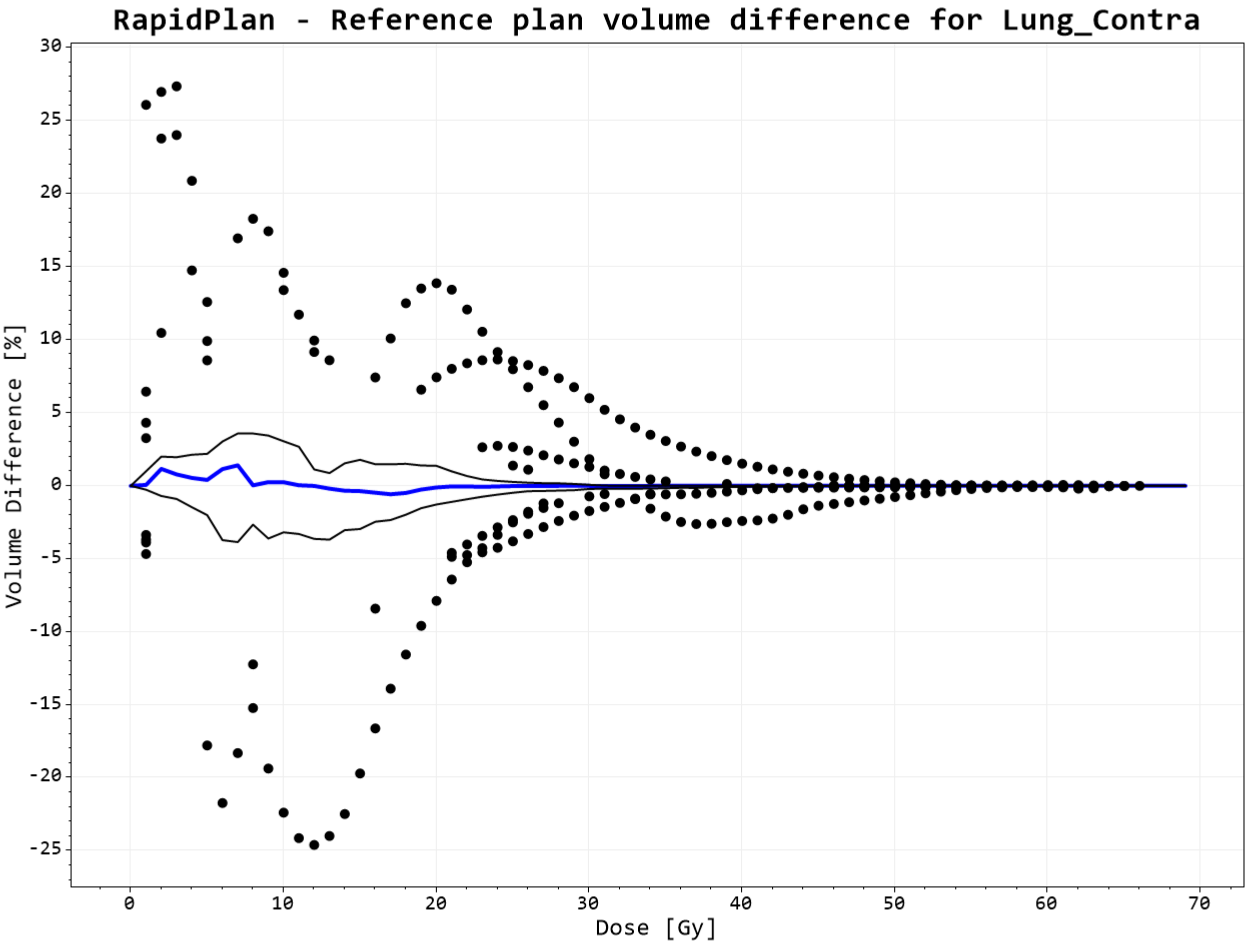


## Dose-volume metric summary table

| Metric | Reference Plan | RapidPlan | Difference |
|---|---|---|---|
| D30%[Gy] | 10.3 [7.2,13.9] | 10.3 [7.1,13.0] | 0.0 [-0.6,0.7] (p = 0.345) (1) |
| V20Gy[%] | 3.5 [1.9,12.7] | 3.2 [0.8,9.1] | -0.1 [-1.3,1.4] (p = 0.846) (2) |
| D60%[Gy] | 4.4 [2.6,6.7] | 4.5 [2.7,7.1] | 0.1 [-0.3,0.7] (p = 0.345) (0) |
| V5Gy[%] | 56.4 [48.5,72.1] | 57.6 [45.0,73.6] | 0.4 [-2.0,2.2] (p = 0.212) (3) |
| DMean[Gy] | 8.2 [5.5,9.8] | 7.5 [5.2,10.0] | 0.0 [-0.6,0.8] (p = 0.500) (1) |

### Dose-volume metric box whisker plots

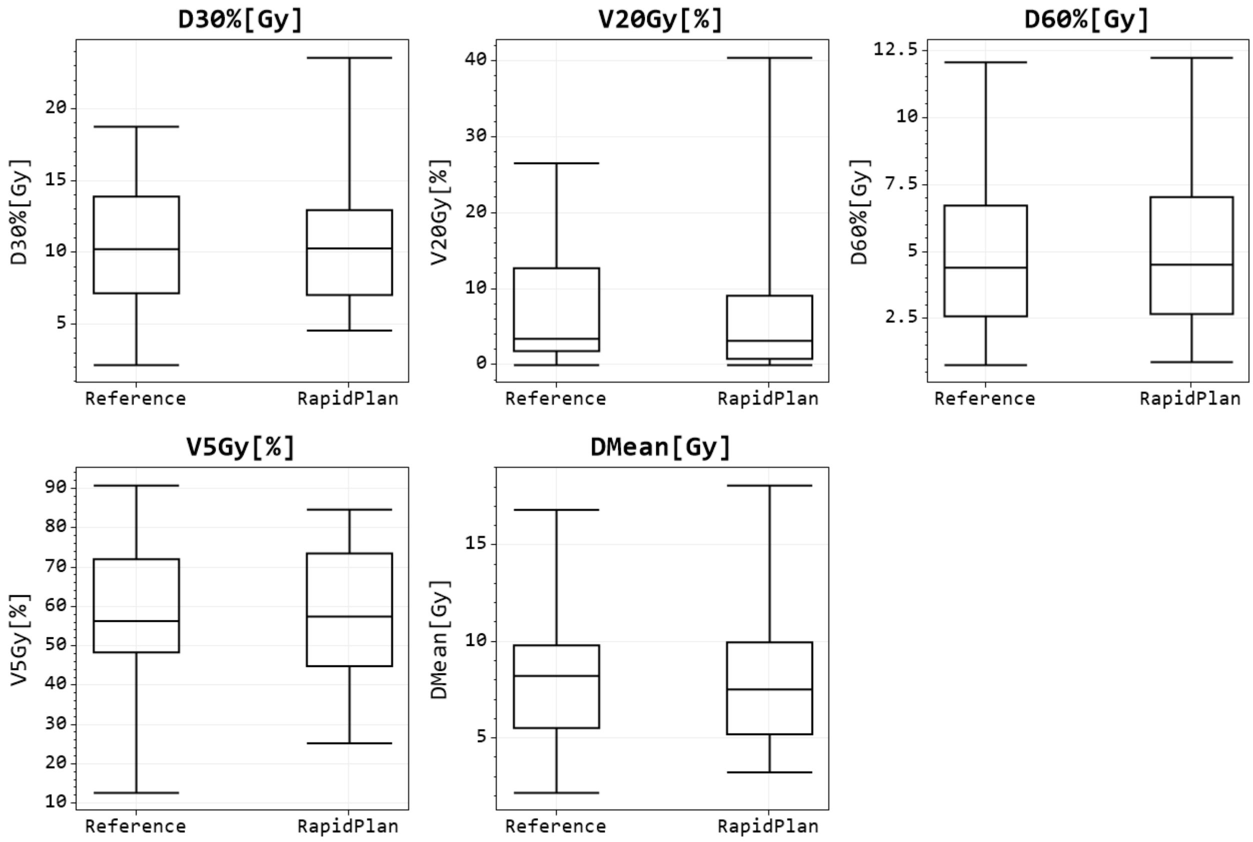

### Dose-volume metric differences by plan

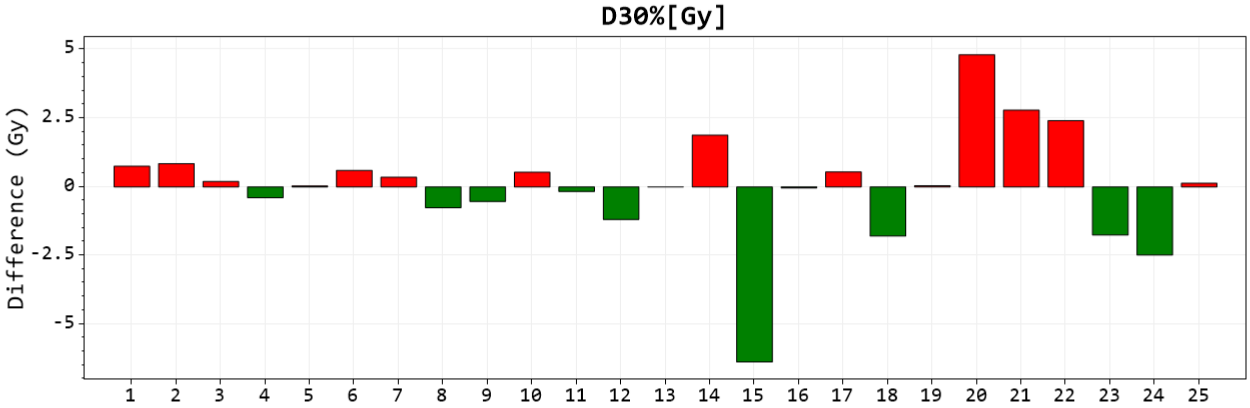


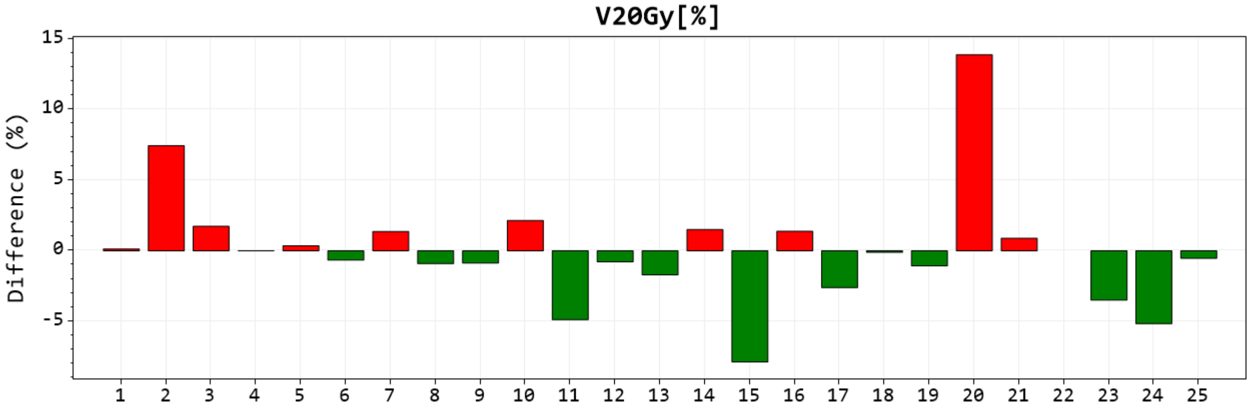


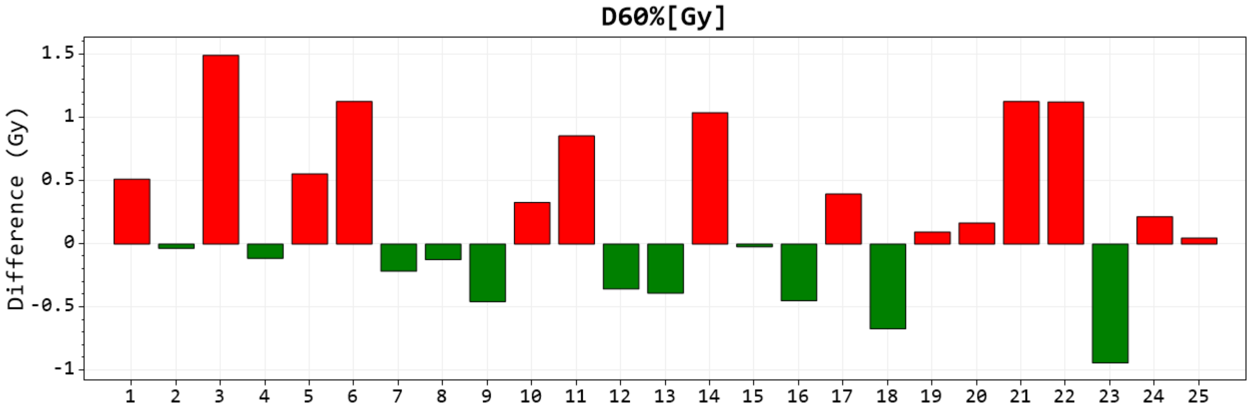

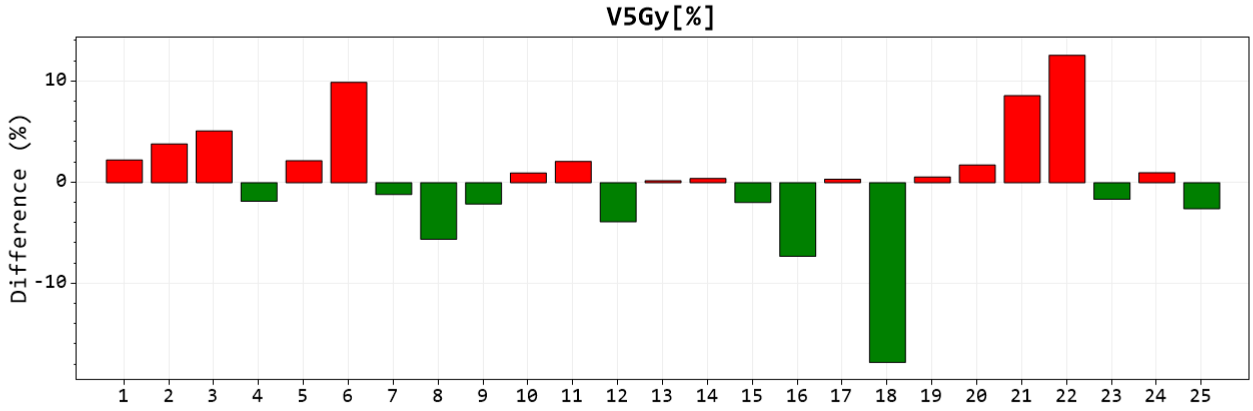
V5Gy[%]
Difference (%)
10
0
-10
1 2 3 4 5 6 7 8 9 10 11 12 13 14 15 16 17 18 19 20 21 22 23 24 25

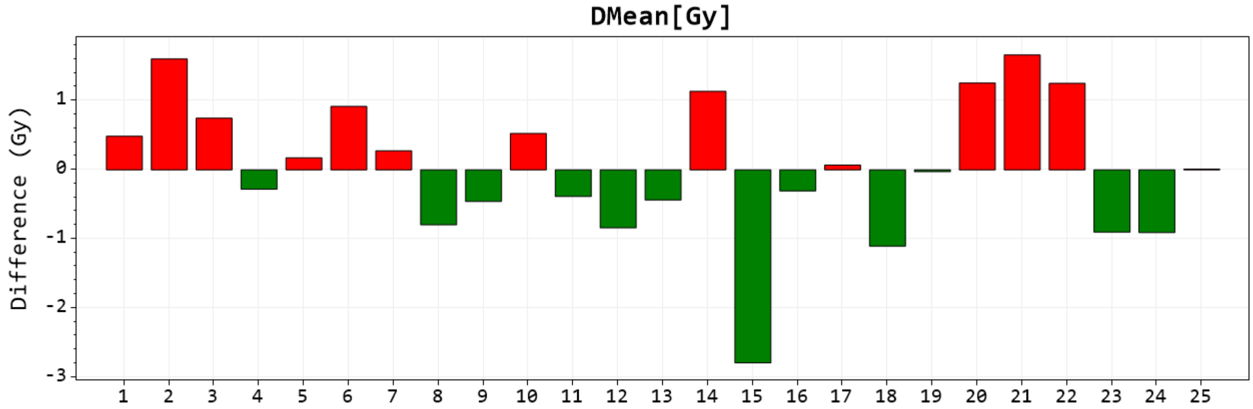
DMean[Gy]
Difference (Gy)
1
0
-1
-2
-3
1 2 3 4 5 6 7 8 9 10 11 12 13 14 15 16 17 18 19 20 21 22 23 24 25

## Dose-volume histograms

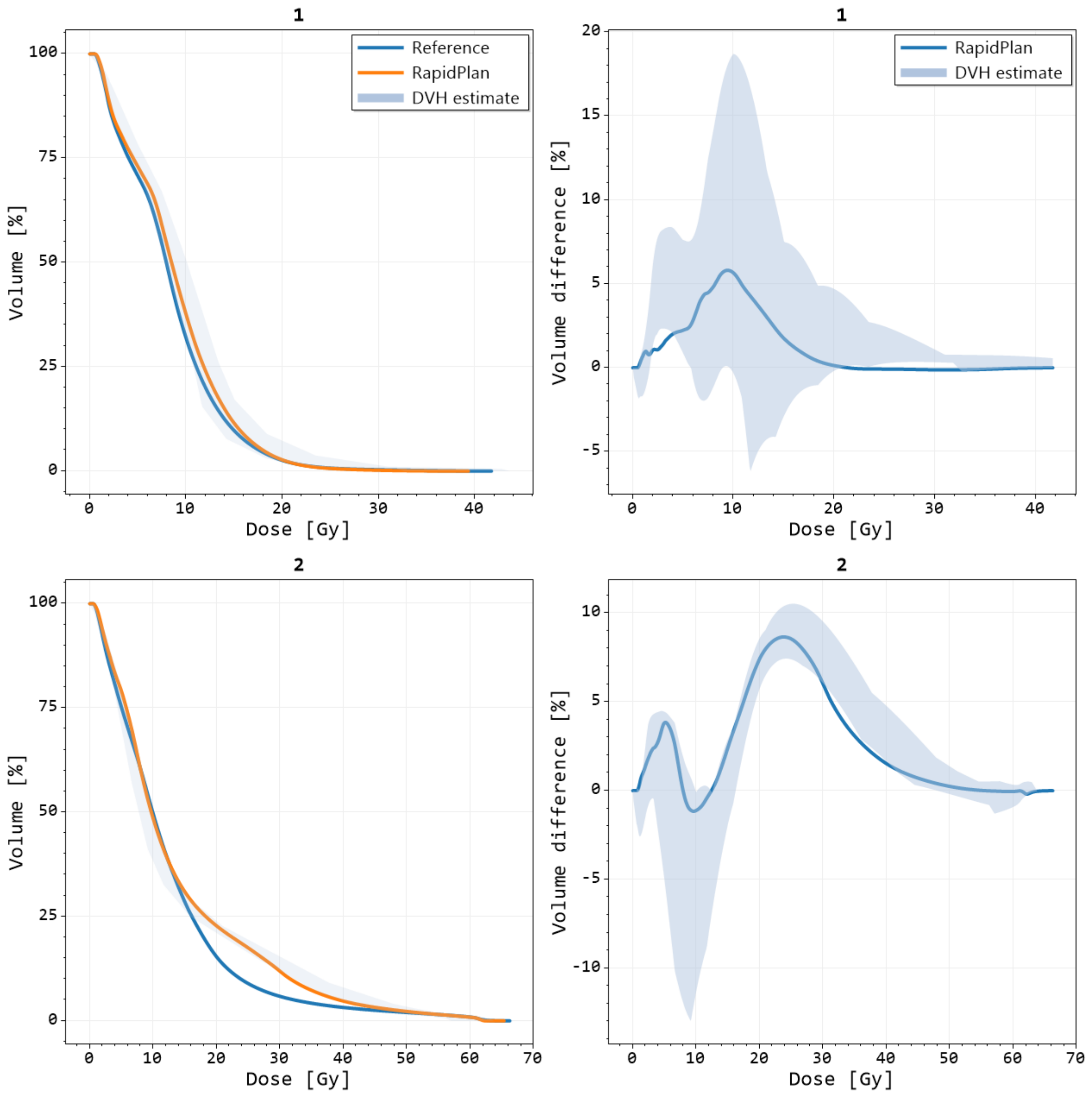

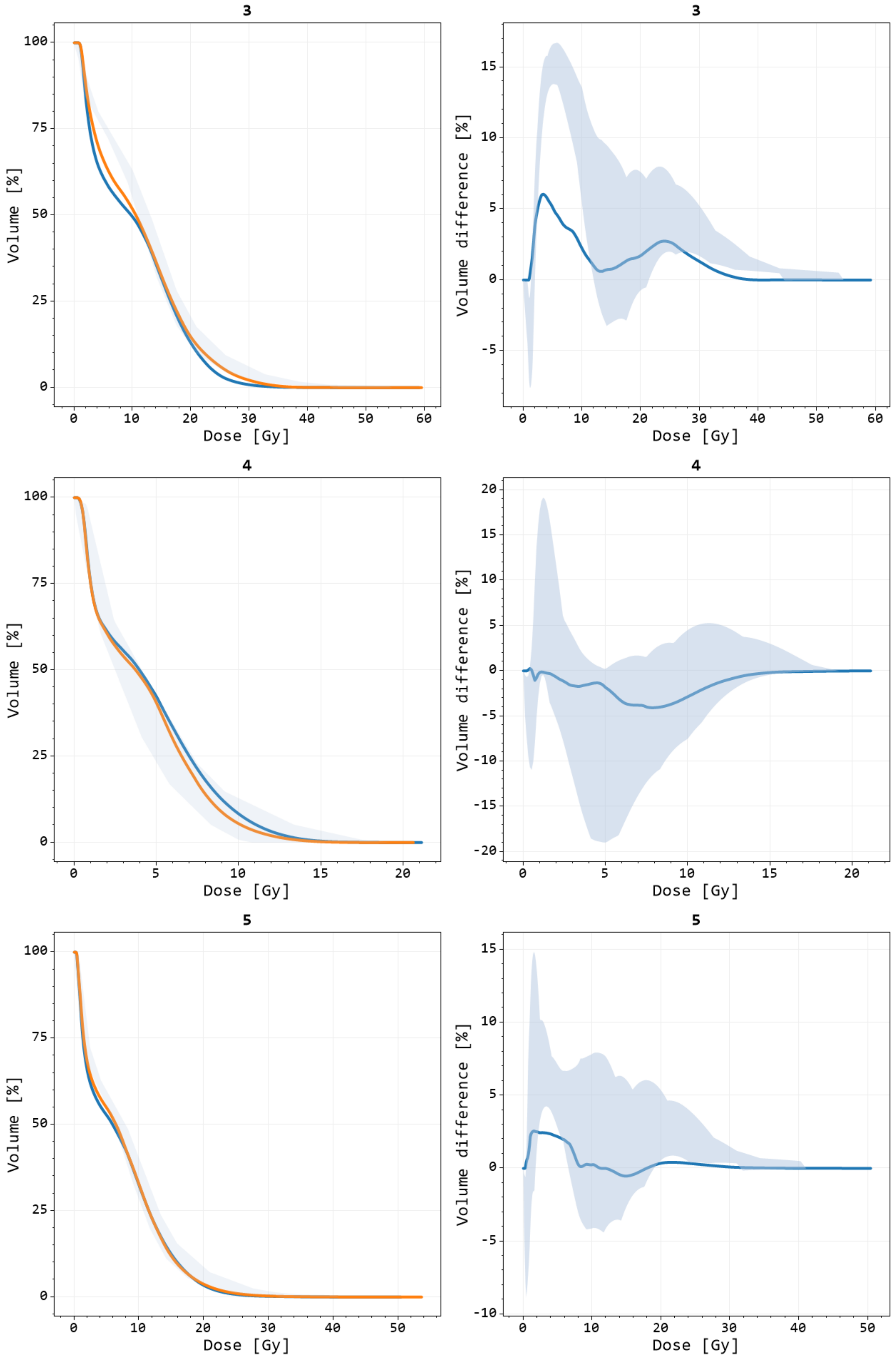
3
Volume [%]
Dose [Gy]
3
Volume difference [%]
Dose [Gy]
4
Volume [%]
Dose [Gy]
4
Volume difference [%]
Dose [Gy]
5
Volume [%]
Dose [Gy]
5
Volume difference [%]
Dose [Gy]

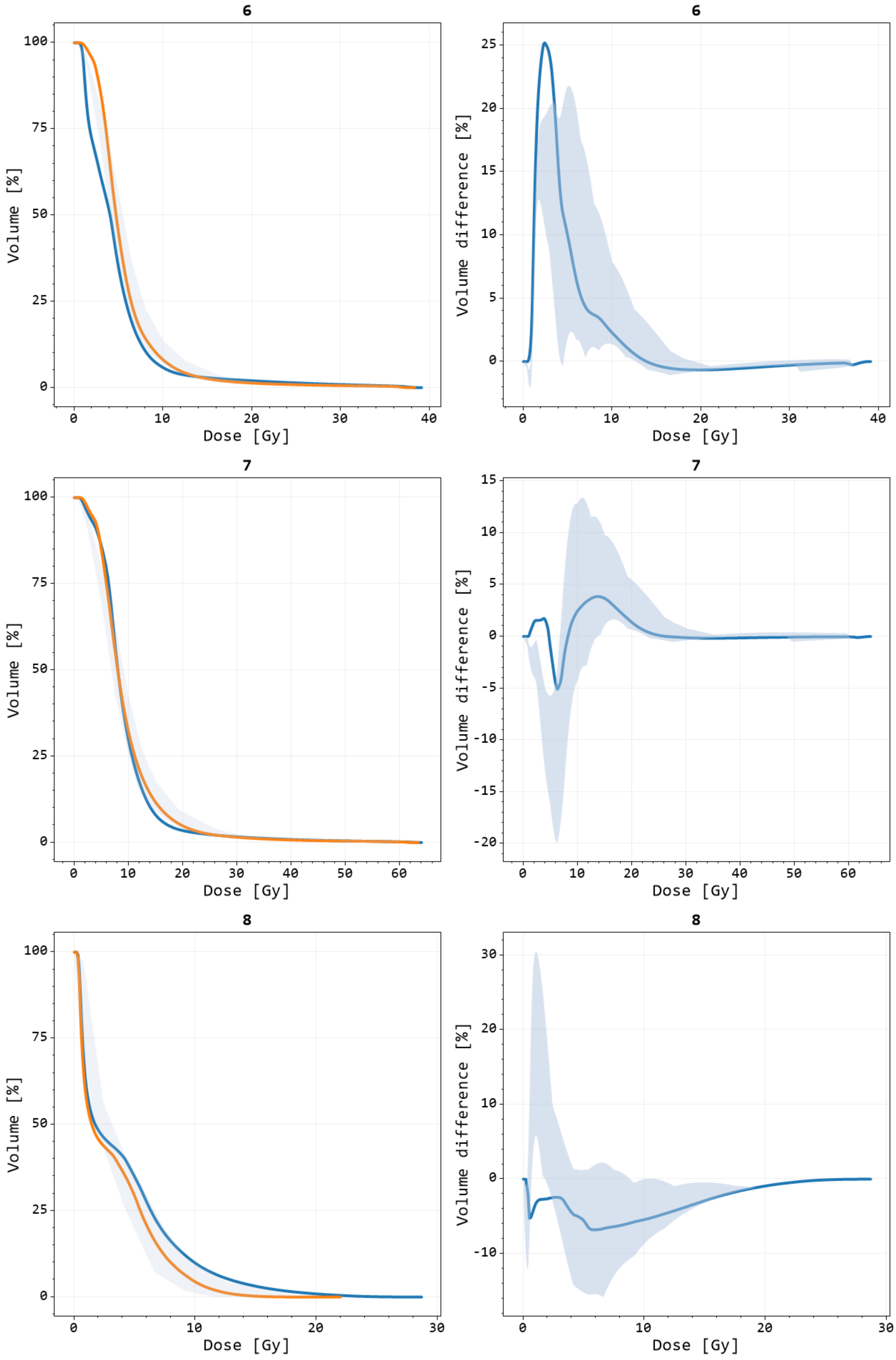
6
Volume [%]
Dose [Gy]
6
Volume difference [%]
Dose [Gy]
7
Volume [%]
Dose [Gy]
7
Volume difference [%]
Dose [Gy]
8
Volume [%]
Dose [Gy]
8
Volume difference [%]
Dose [Gy]

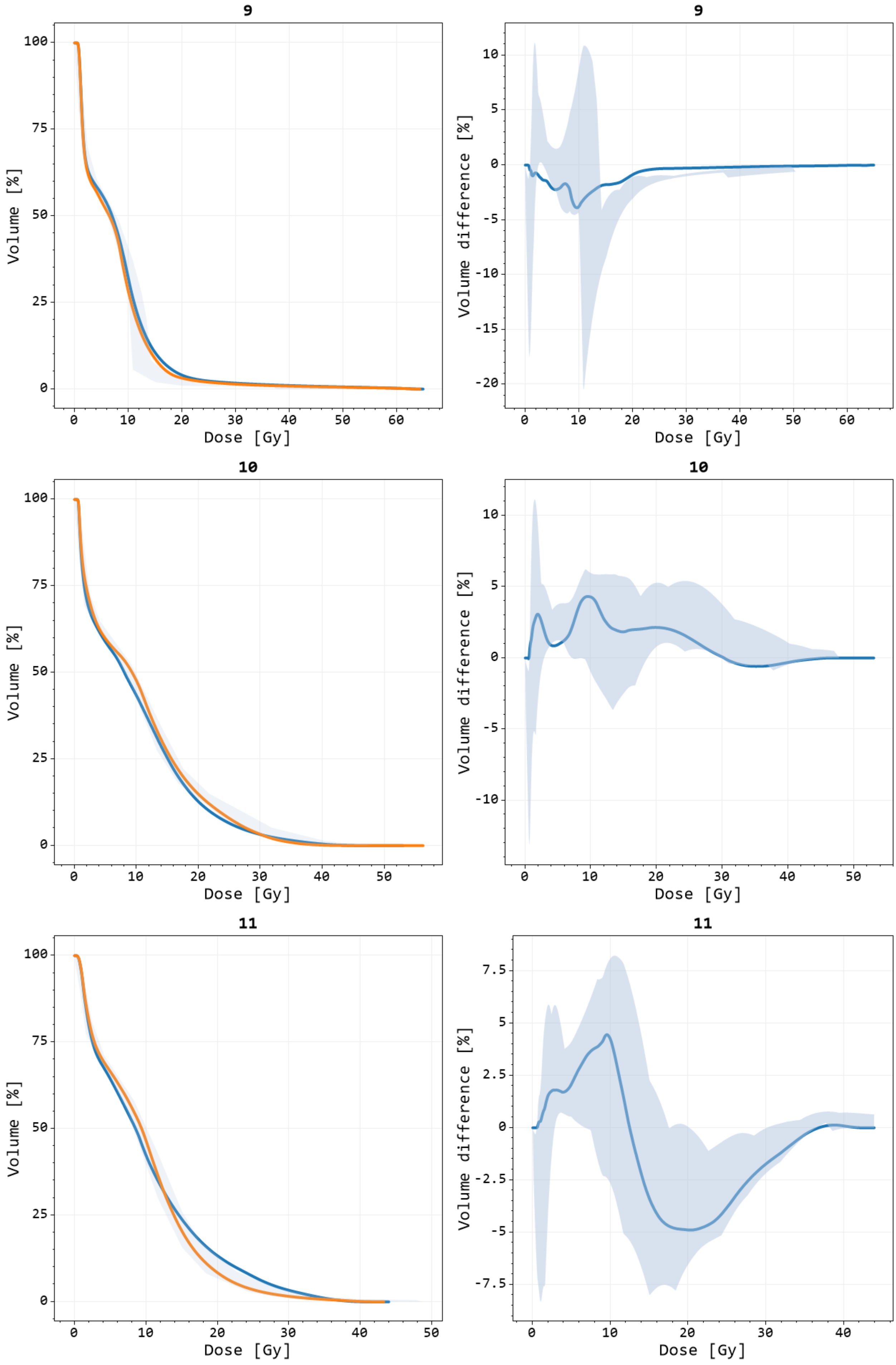
9
Volume [%]
Dose [Gy]
Volume difference [%]
10
11

**12**

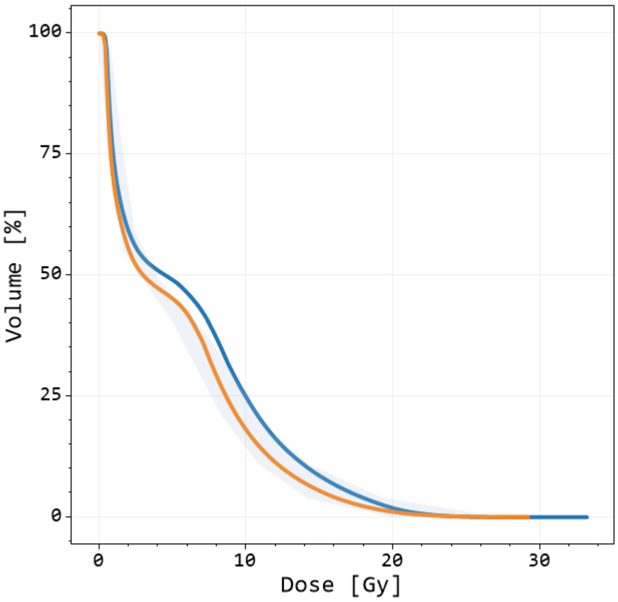


**12**

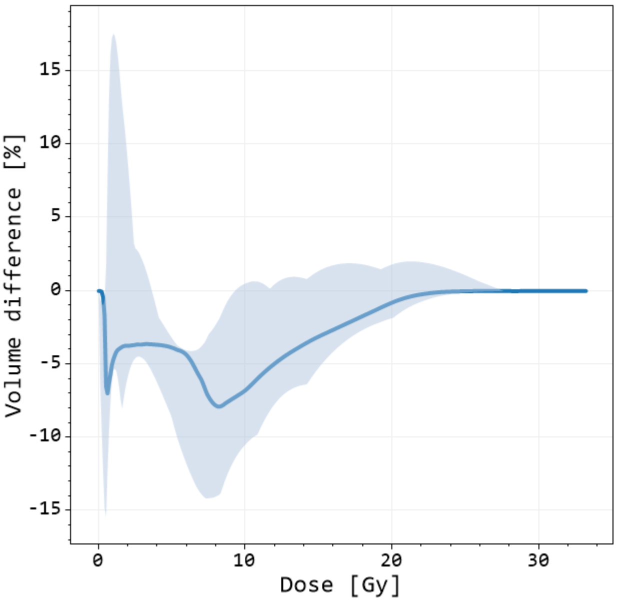


**13**

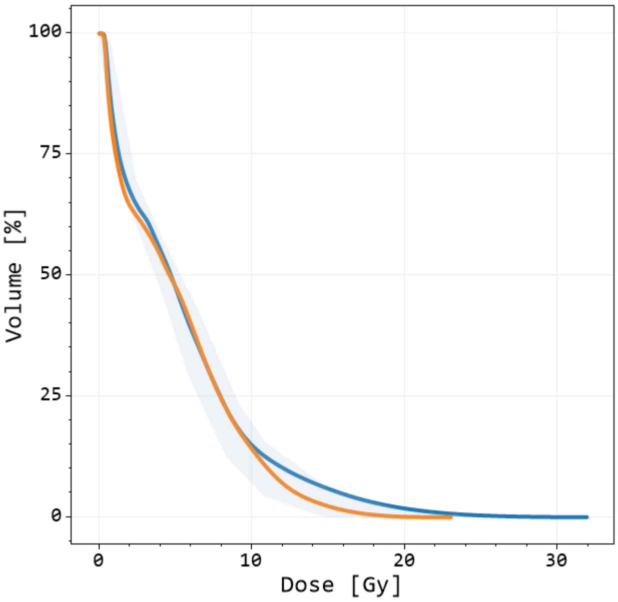


**13**

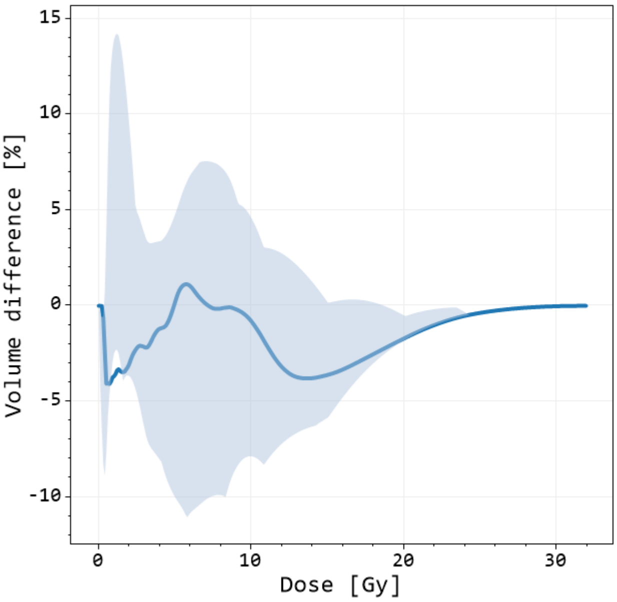


**14**

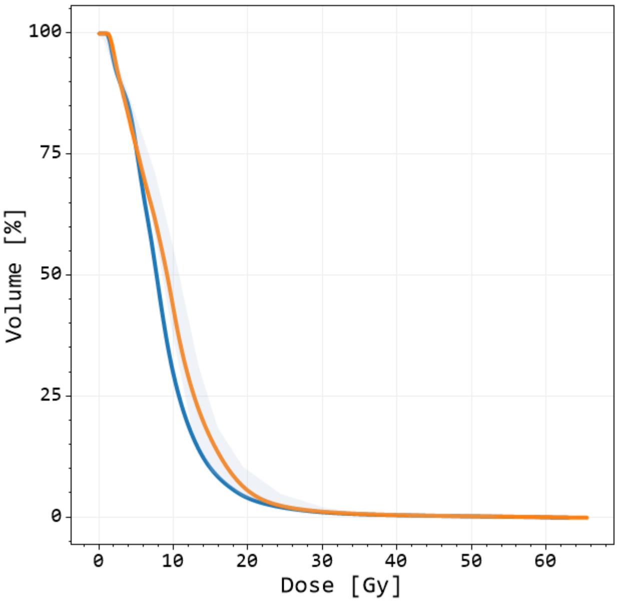


**14**

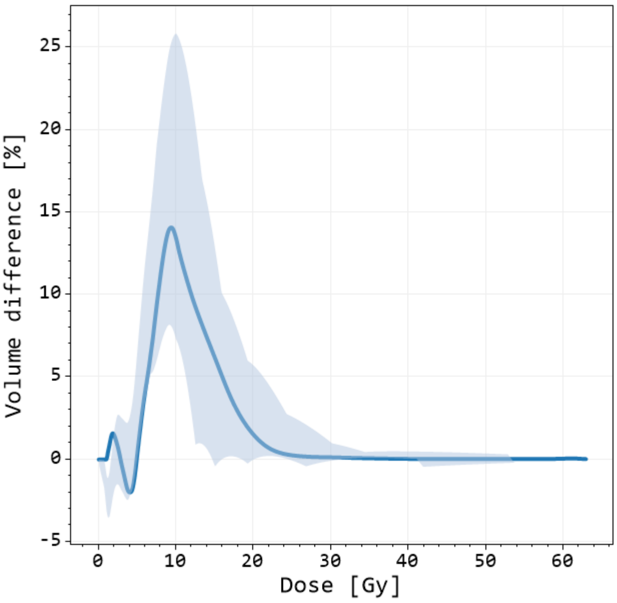

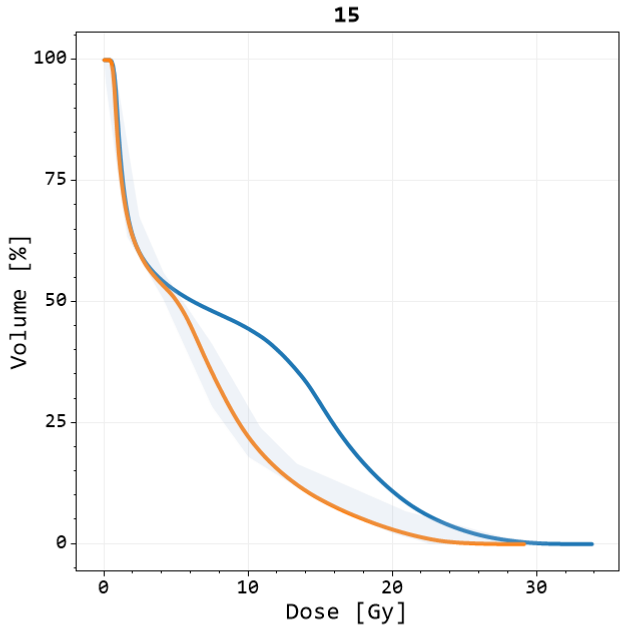
15
100
75
50
25
0
Volume [%]
0
10
20
30
Dose [Gy]

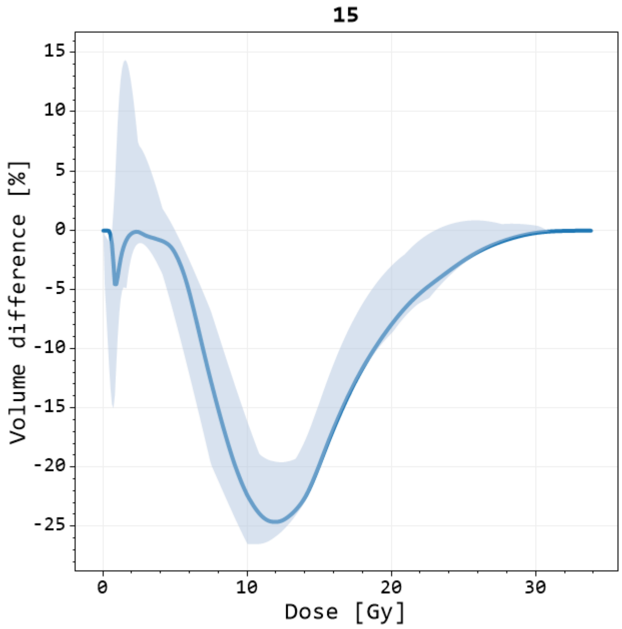
15
15
10
5
0
-5
-10
-15
-20
-25
Volume difference [%]
0
10
20
30
Dose [Gy]

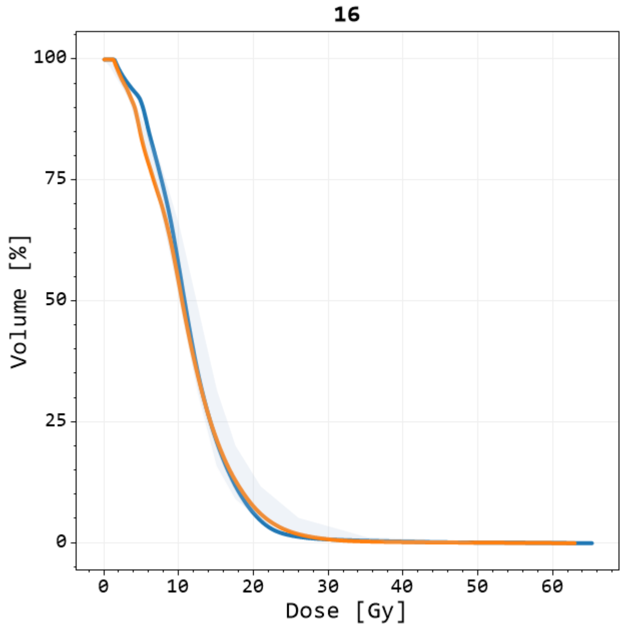
16
100
75
50
25
0
Volume [%]
0
10
20
30
40
50
60
Dose [Gy]

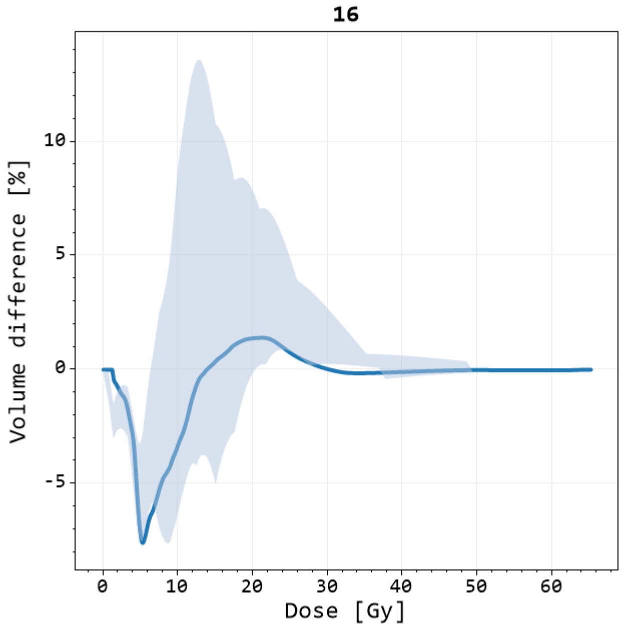
16
10
5
0
-5
Volume difference [%]
0
10
20
30
40
50
60
Dose [Gy]

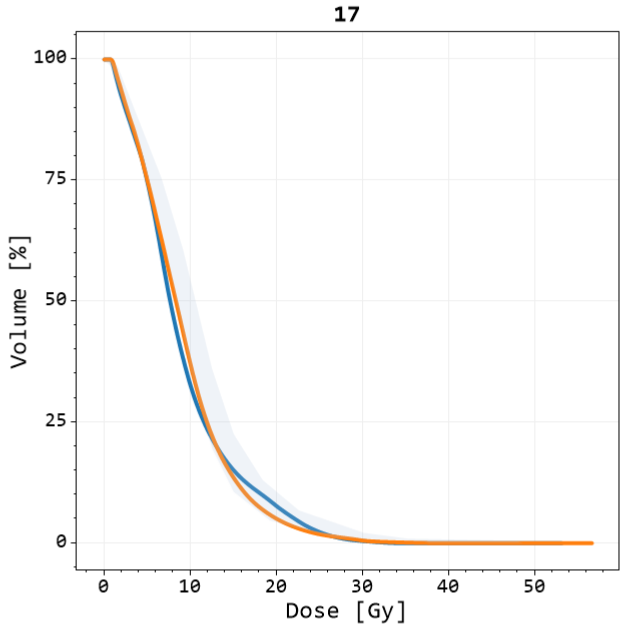
17
100
75
50
25
0
Volume [%]
0
10
20
30
40
50
Dose [Gy]

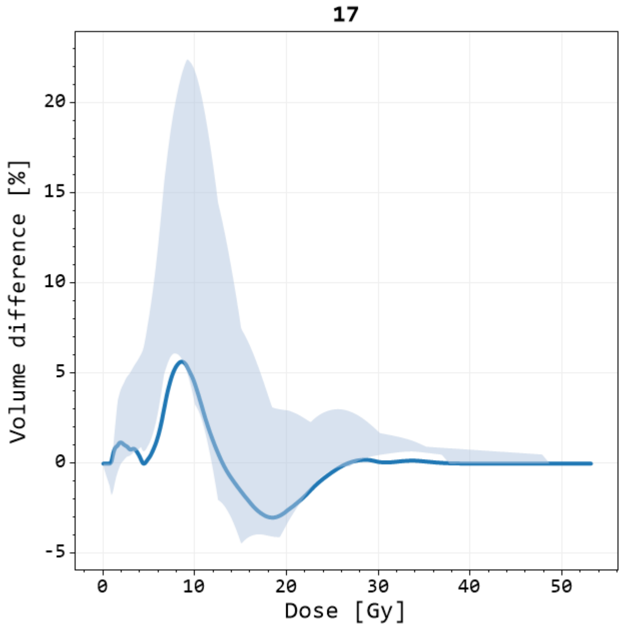
17
20
15
10
5
0
-5
Volume difference [%]
0
10
20
30
40
50
Dose [Gy]

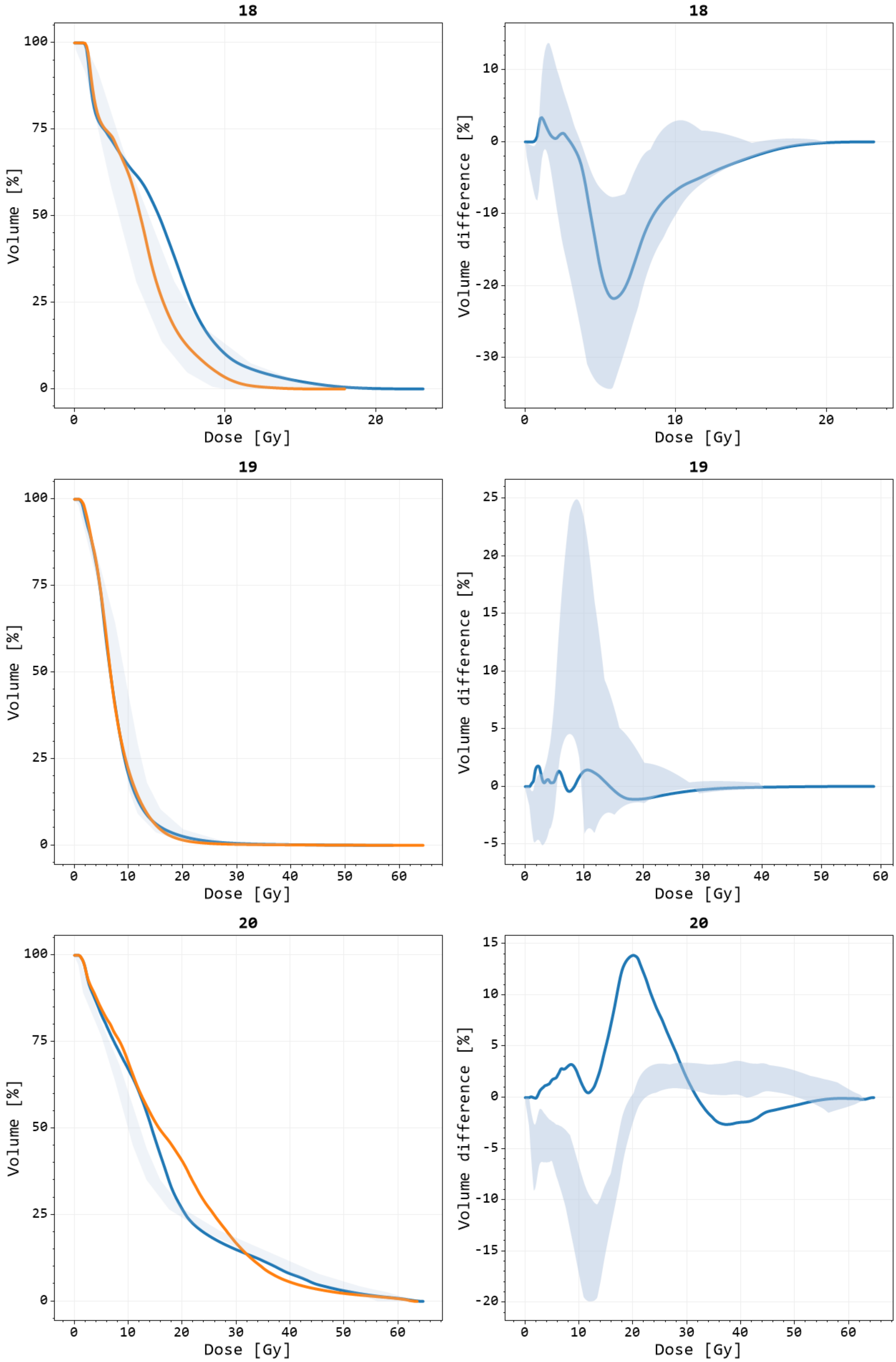
18
Volume [%]
Dose [Gy]
18
Volume difference [%]
Dose [Gy]
19
Volume [%]
Dose [Gy]
19
Volume difference [%]
Dose [Gy]
20
Volume [%]
Dose [Gy]
20
Volume difference [%]
Dose [Gy]

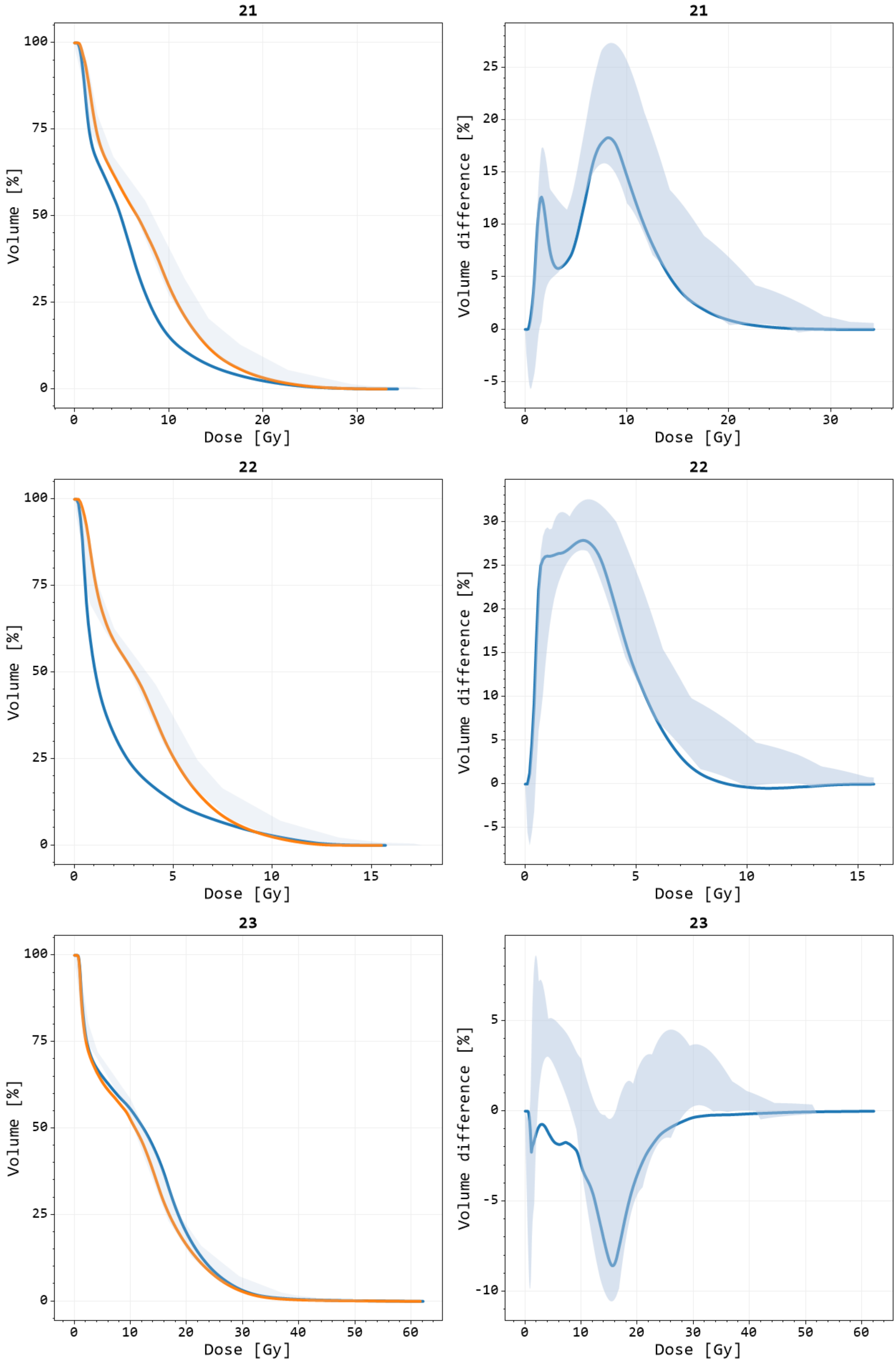
21
Volume [%]
Dose [Gy]
21
Volume difference [%]
Dose [Gy]
22
Volume [%]
Dose [Gy]
22
Volume difference [%]
Dose [Gy]
23
Volume [%]
Dose [Gy]
23
Volume difference [%]
Dose [Gy]

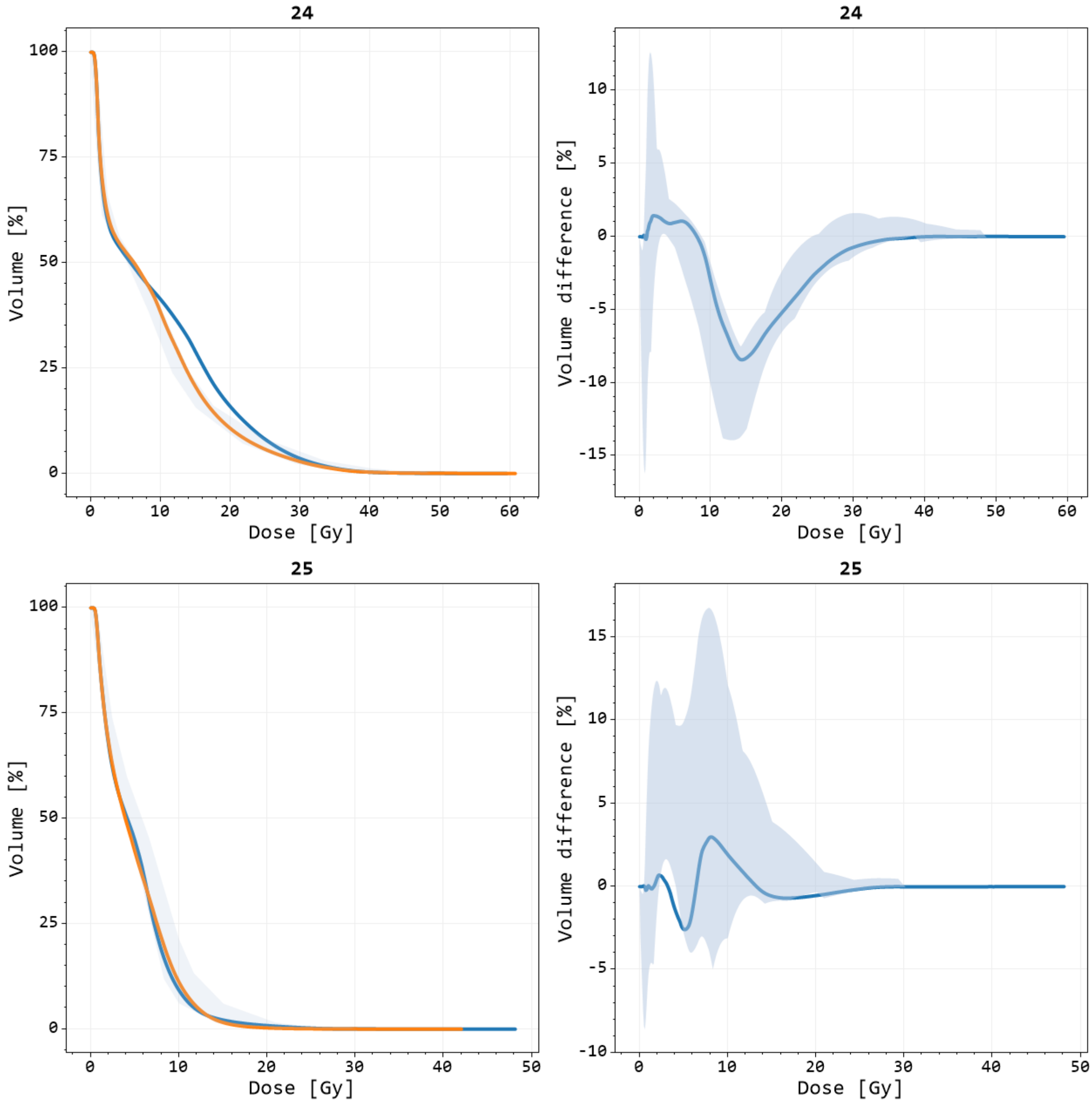
24
Volume [%]
100
75
50
25
0
0
10
20
30
40
50
60
Dose [Gy]
24
Volume difference [%]
10
5
0
-5
-10
-15
0
10
20
30
40
50
60
Dose [Gy]
25
Volume [%]
100
75
50
25
0
0
10
20
30
40
50
Dose [Gy]
25
Volume difference [%]
15
10
5
0
-5
-10
0
10
20
30
40
50
Dose [Gy]

# Lung_Ipsi

## DVH Volume difference

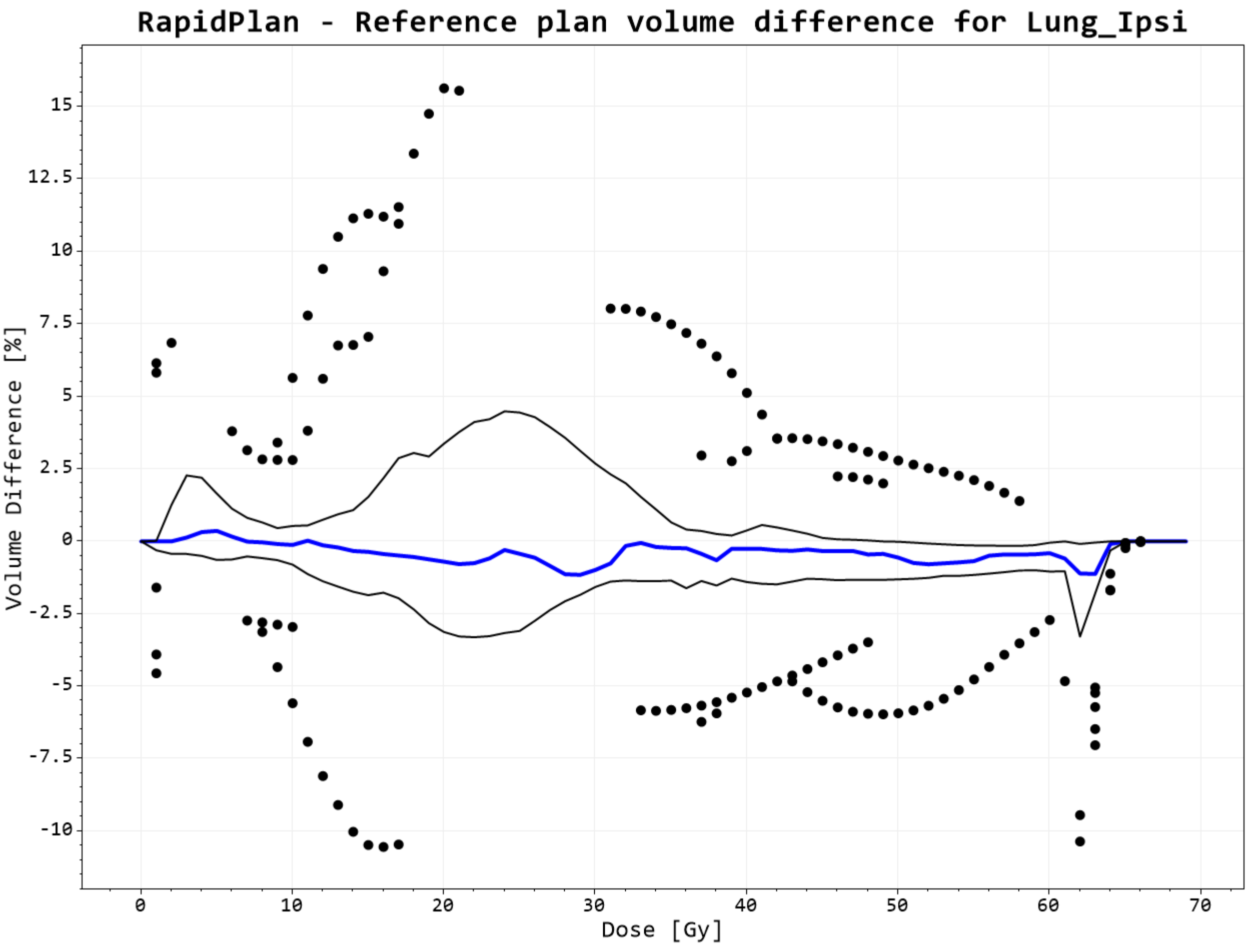


## Dose-volume metric summary table

| Metric | Reference Plan | RapidPlan | Difference |
|---|---|---|---|
| D30%[Gy] | 34.6 [27.2,47.8] | 34.5 [27.9,46.9] | -0.8 [-1.2,1.1] (p = 0.946) (2) |
| V20Gy[%] | 50.6 [40.1,55.9] | 48.6 [40.0,60.5] | -0.7 [-3.1,3.4] (p = 0.788) (1) |
| D60%[Gy] | 8.8 [4.6,17.9] | 9.1 [4.9,20.7] | -0.1 [-0.5,1.0] (p = 0.788) (7) |
| V5Gy[%] | 67.1 [59.3,83.2] | 68.3 [59.6,86.0] | 0.4 [-0.6,1.6] (p = 0.345) (0) |
| DMean[Gy] | 23.4 [19.3,29.9] | 23.0 [18.6,29.9] | -0.3 [-0.7,0.5] (p = 0.946) (0) |

### Dose-volume metric box whisker plots

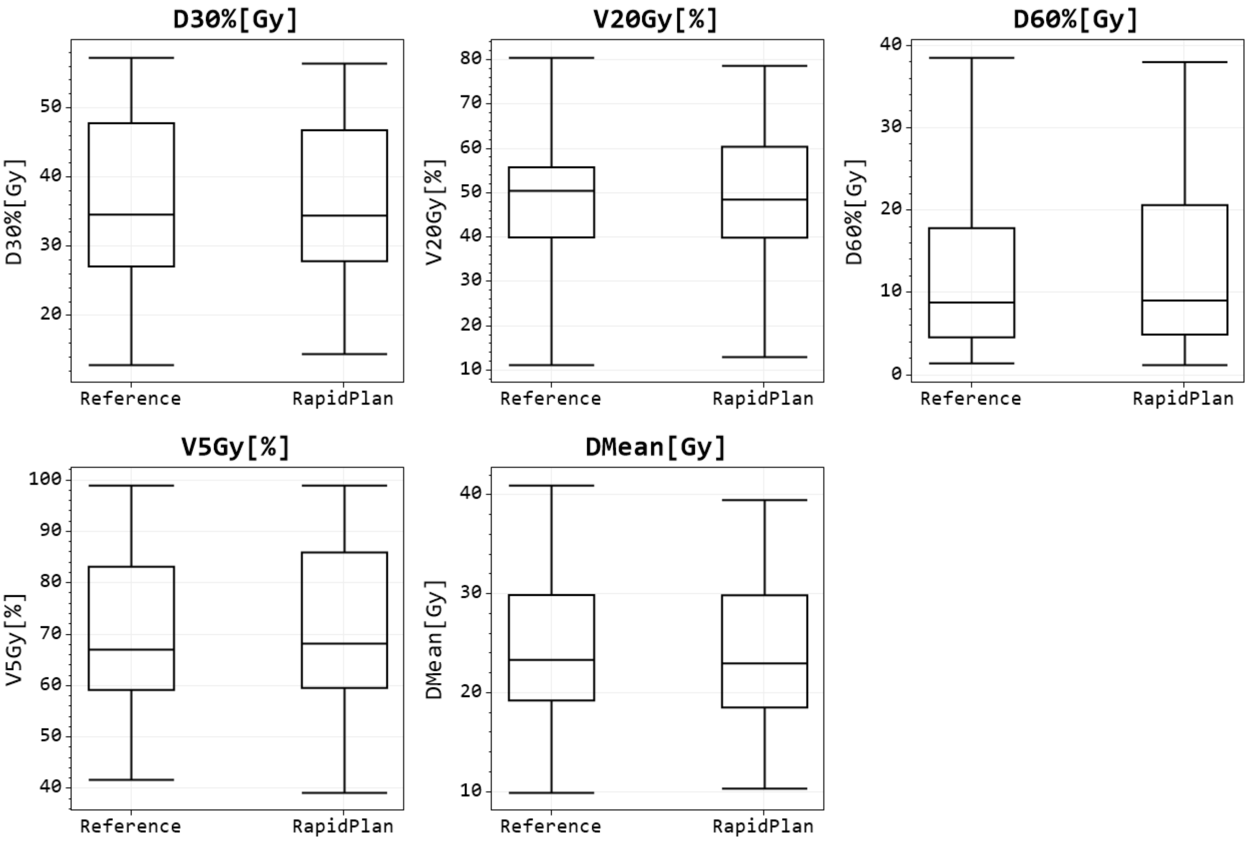

## Dose-volume metric differences by plan

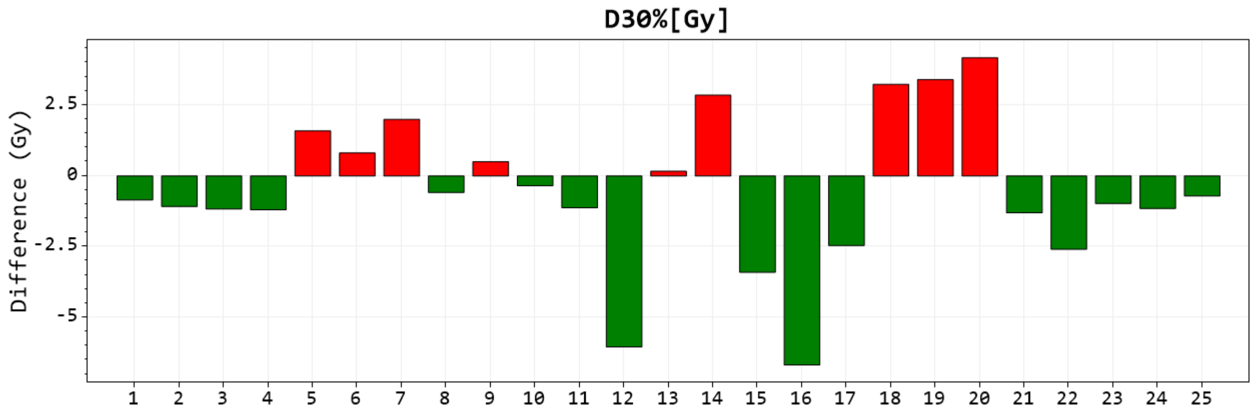


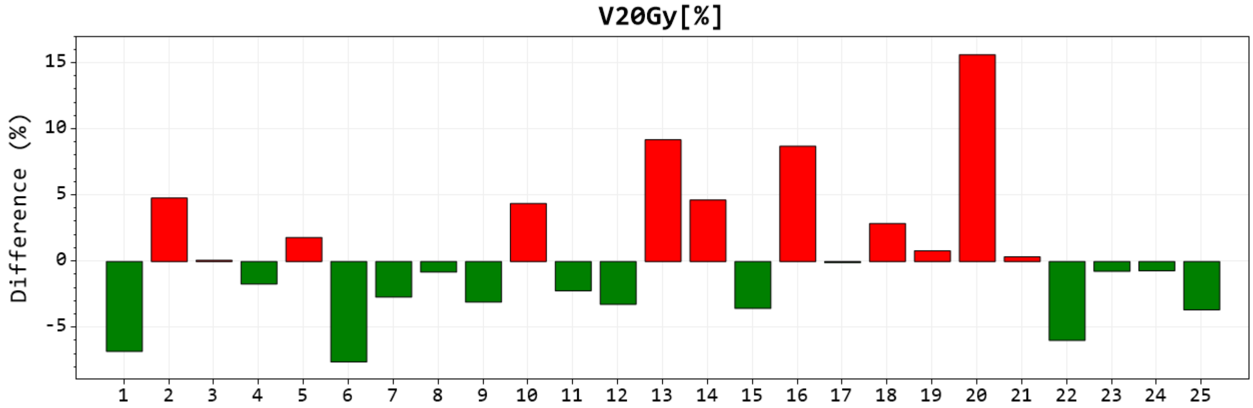


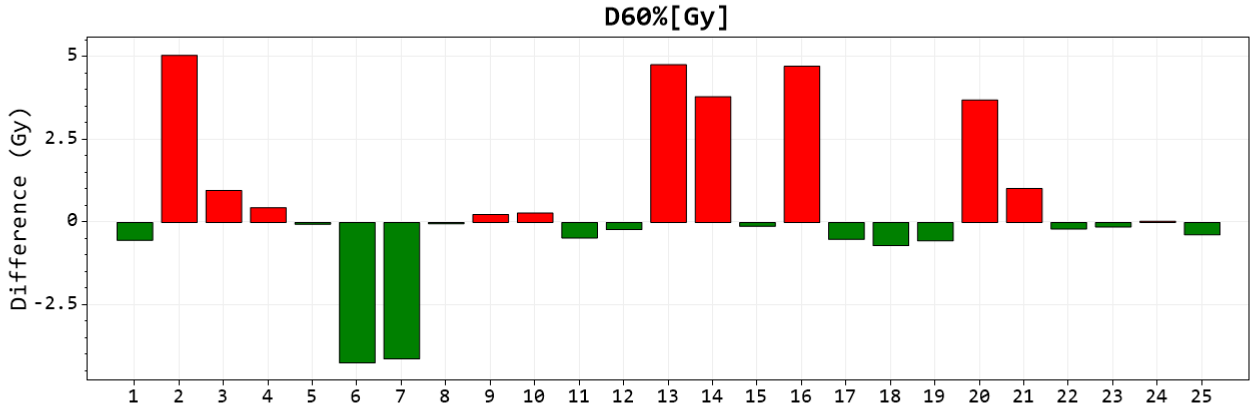

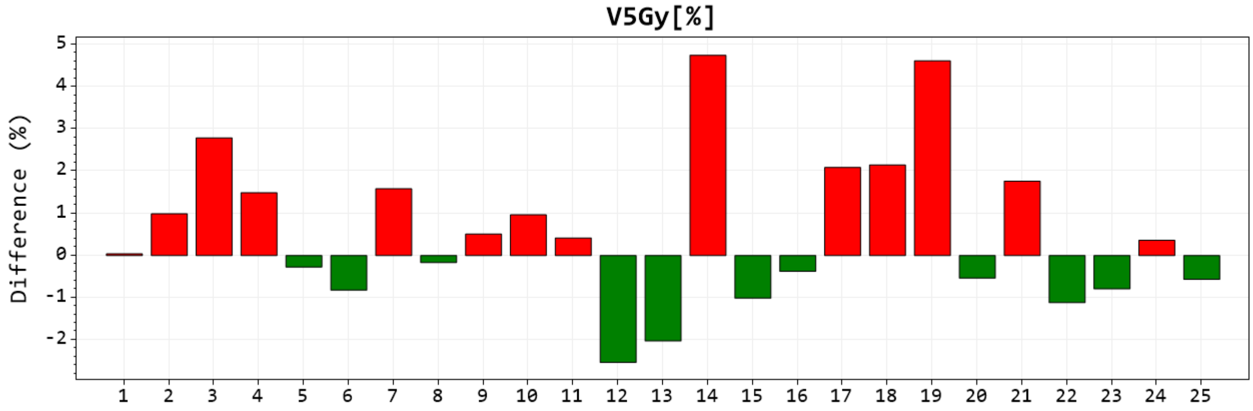
V5Gy[%]
Difference (%)
5
4
3
2
1
0
-1
-2
1 2 3 4 5 6 7 8 9 10 11 12 13 14 15 16 17 18 19 20 21 22 23 24 25

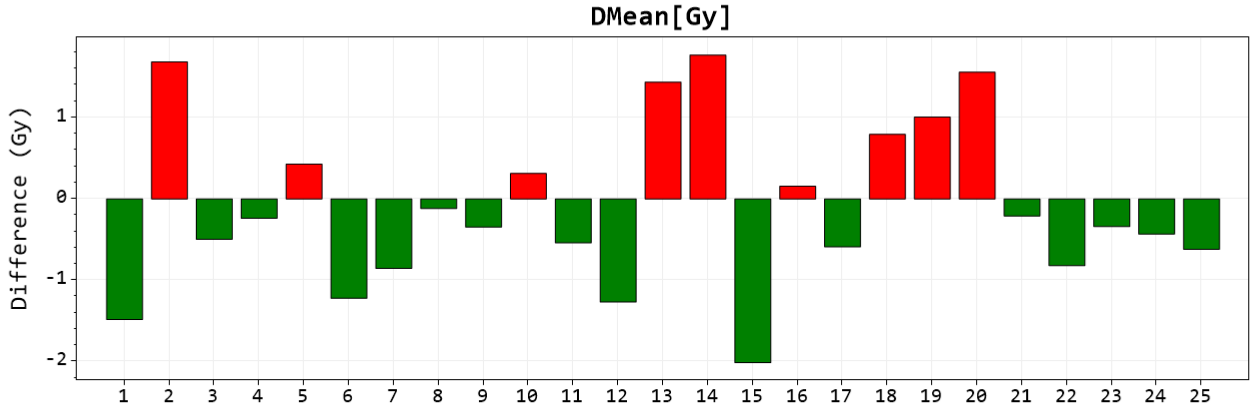
DMean[Gy]
Difference (Gy)
1
0
-1
-2
1 2 3 4 5 6 7 8 9 10 11 12 13 14 15 16 17 18 19 20 21 22 23 24 25

## Dose-volume histograms

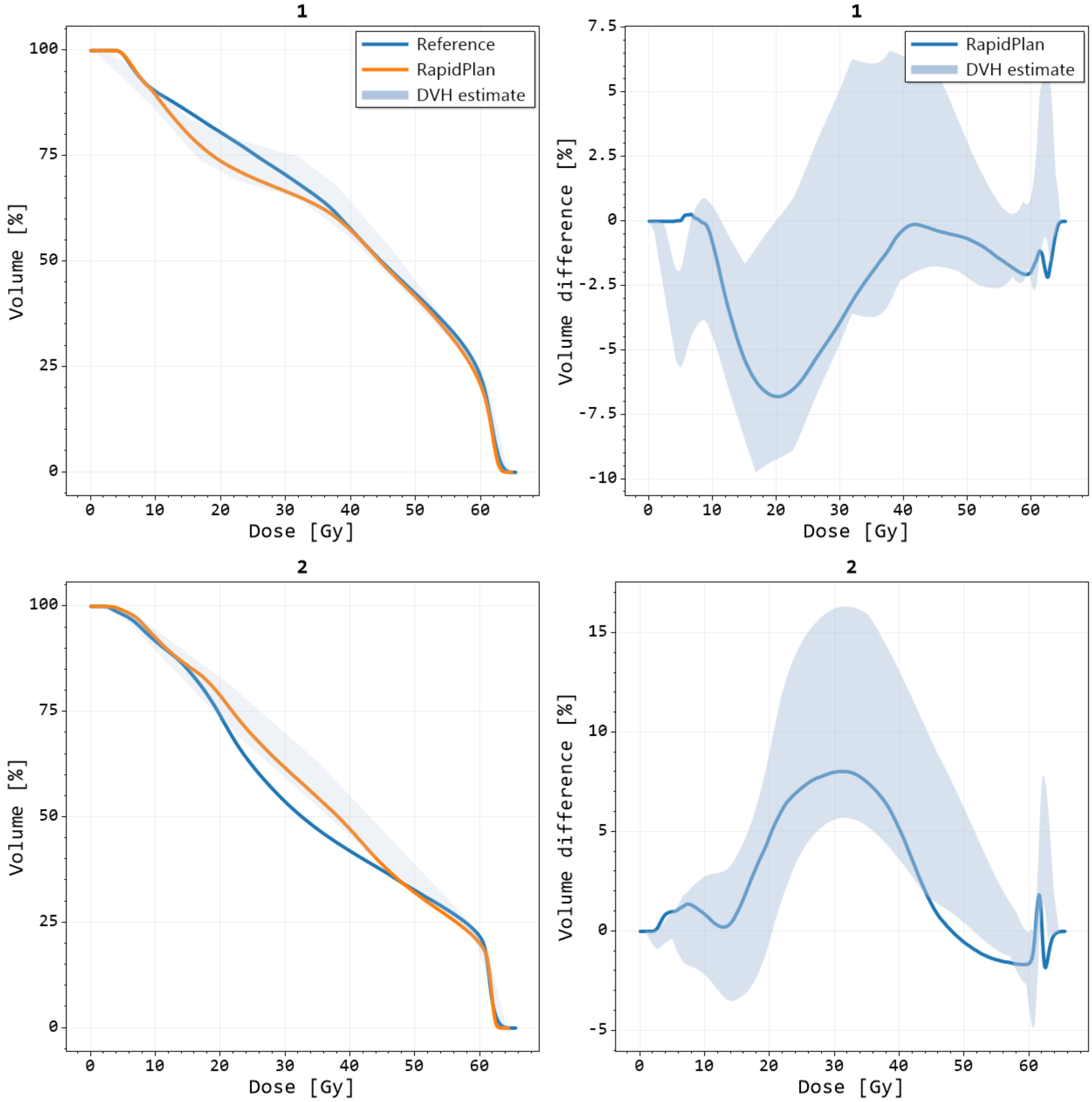

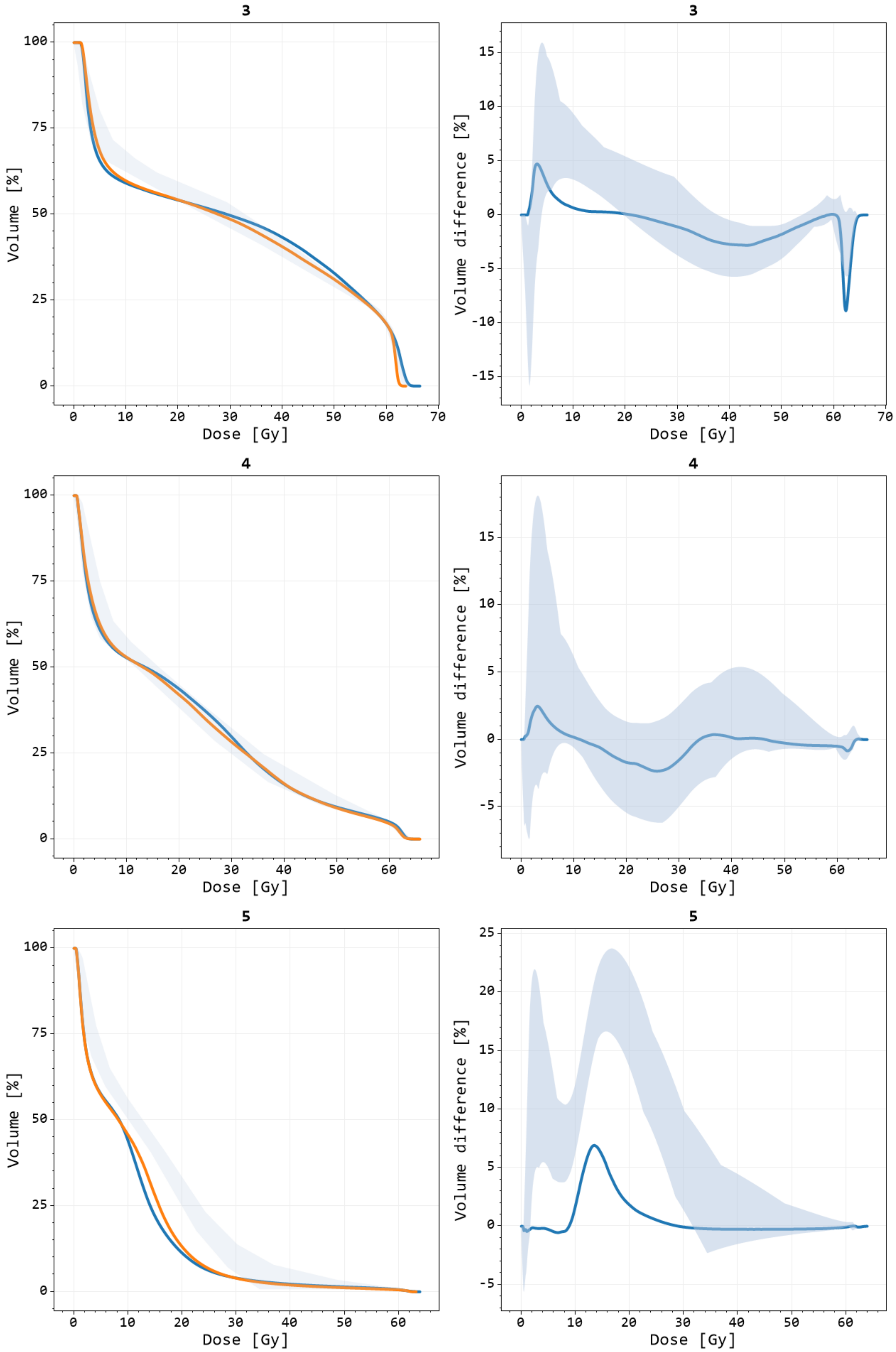
3
Volume [%]
Dose [Gy]
3
Volume difference [%]
Dose [Gy]
4
Volume [%]
Dose [Gy]
4
Volume difference [%]
Dose [Gy]
5
Volume [%]
Dose [Gy]
5
Volume difference [%]
Dose [Gy]

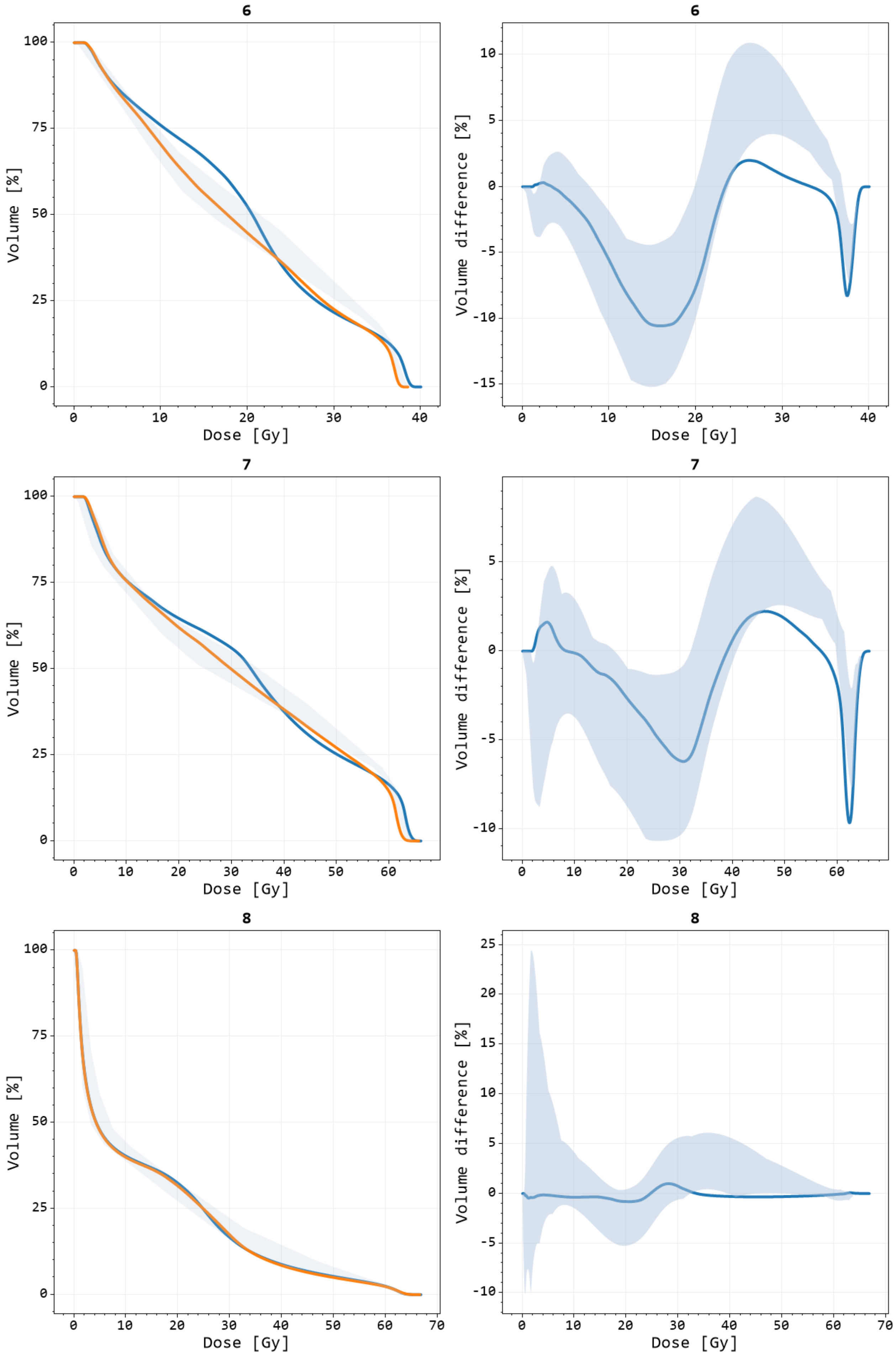
6
Volume [%]
Dose [Gy]
6
Volume difference [%]
Dose [Gy]
7
Volume [%]
Dose [Gy]
7
Volume difference [%]
Dose [Gy]
8
Volume [%]
Dose [Gy]
8
Volume difference [%]
Dose [Gy]

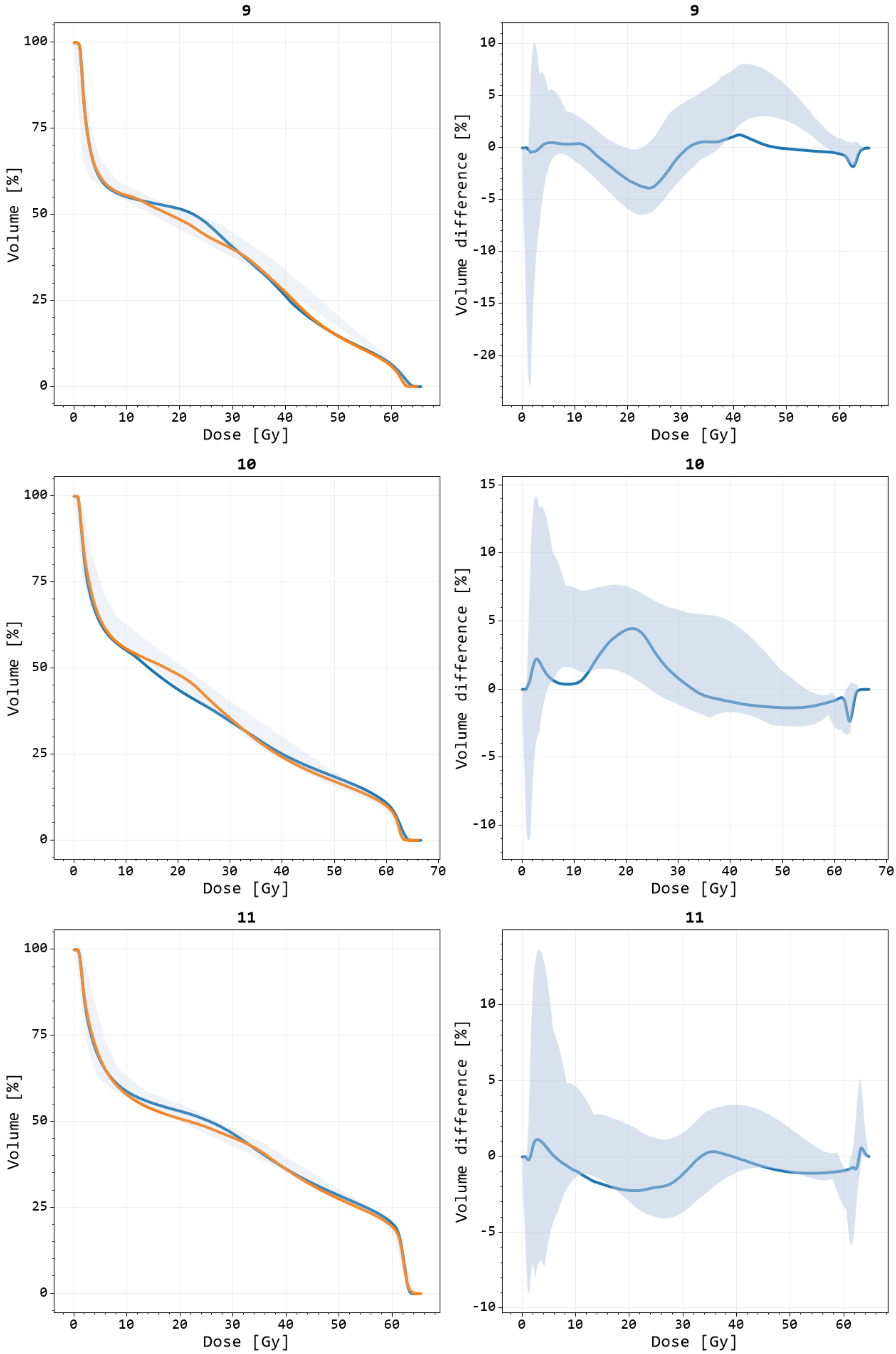
9
Volume [%]
Dose [Gy]
100
75
50
25
0
0
10
20
30
40
50
60
9
Volume difference [%]
Dose [Gy]
10
5
0
-5
-10
-15
-20
10
Volume [%]
Dose [Gy]
70
10
Volume difference [%]
Dose [Gy]
15
11
Volume [%]
Dose [Gy]
11
Volume difference [%]
Dose [Gy]

12

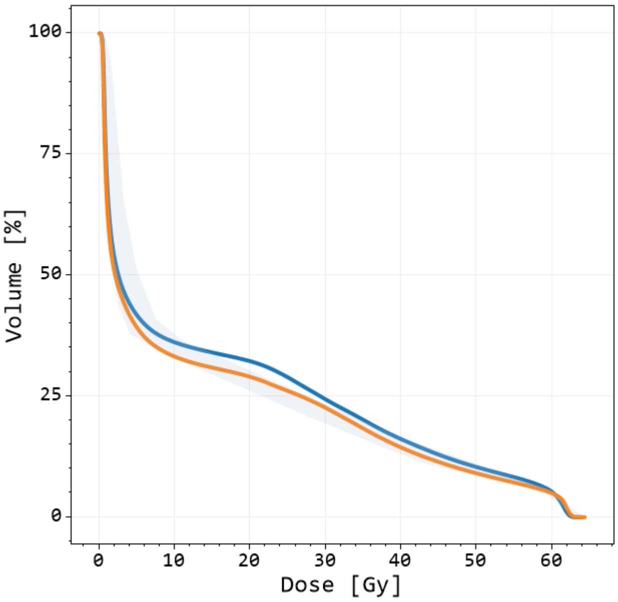


12

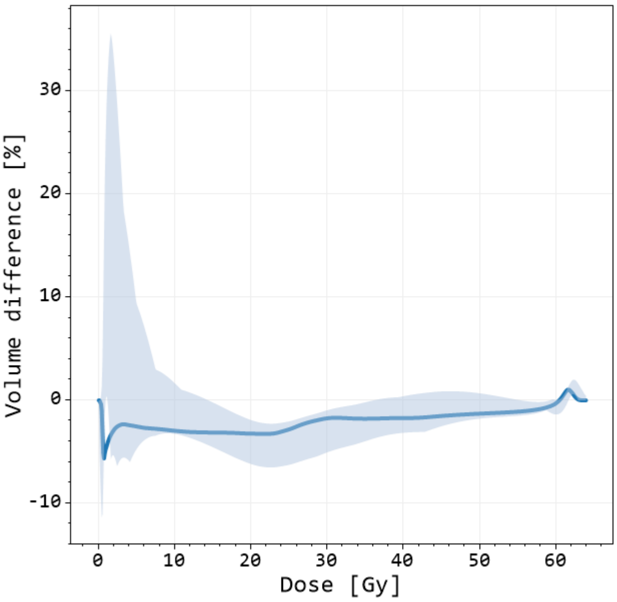


13

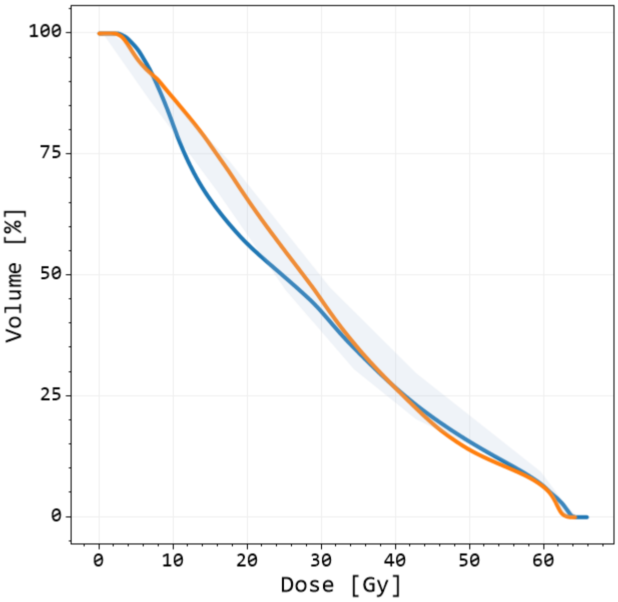


13

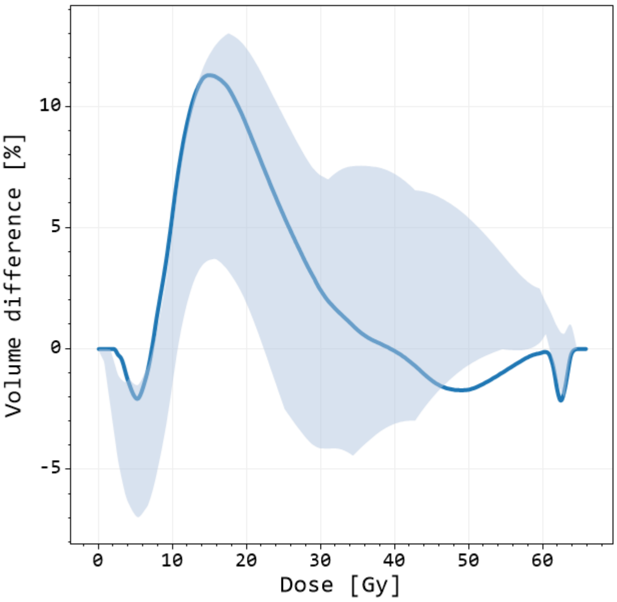


14

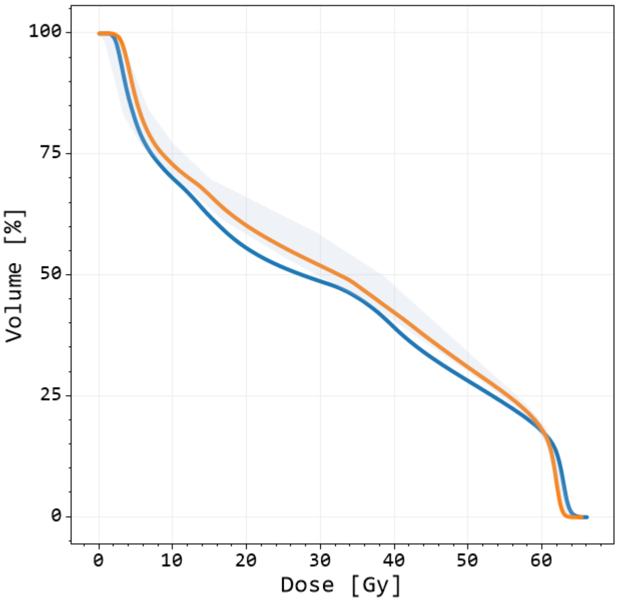


14

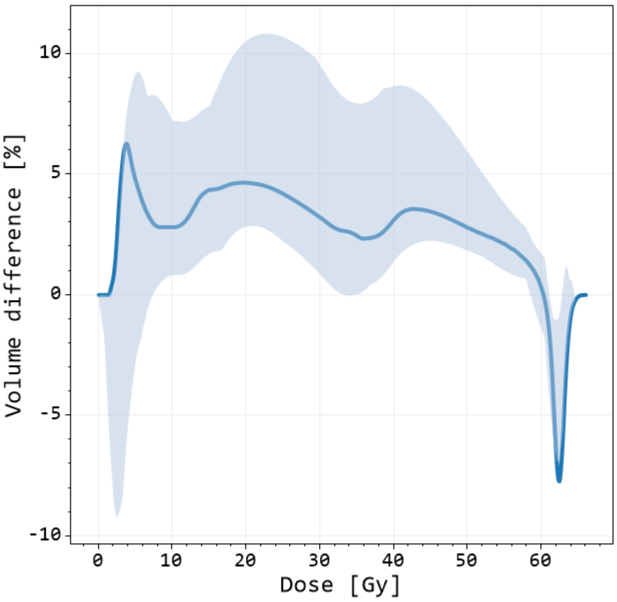

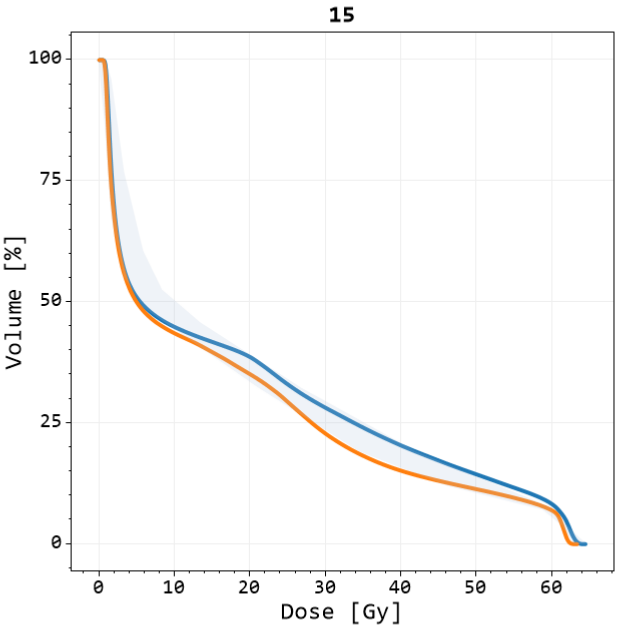
15
Volume [%]
Dose [Gy]

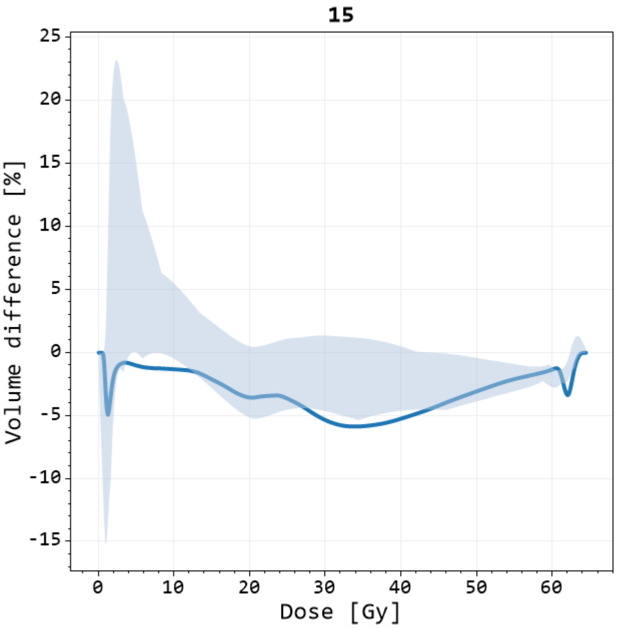
15
Volume difference [%]
Dose [Gy]

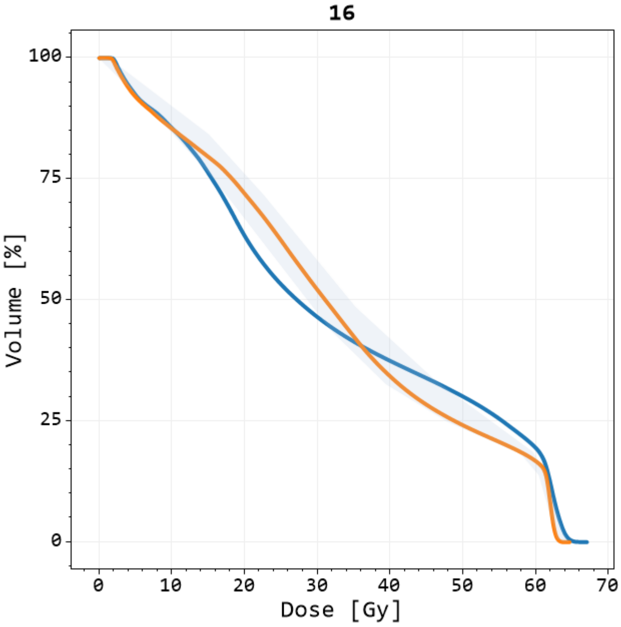
16
Volume [%]
Dose [Gy]

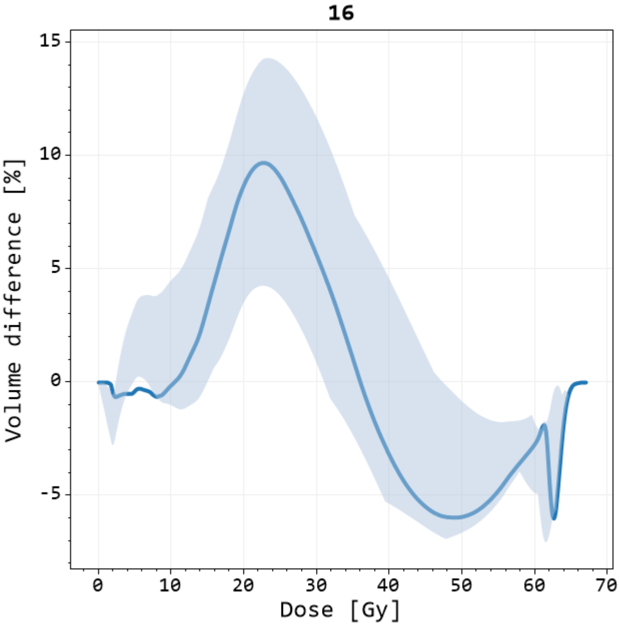
16
Volume difference [%]
Dose [Gy]

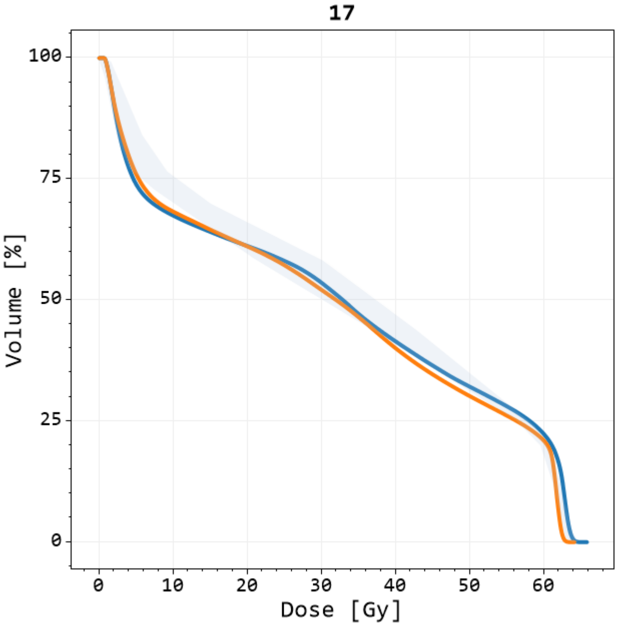
17
Volume [%]
Dose [Gy]

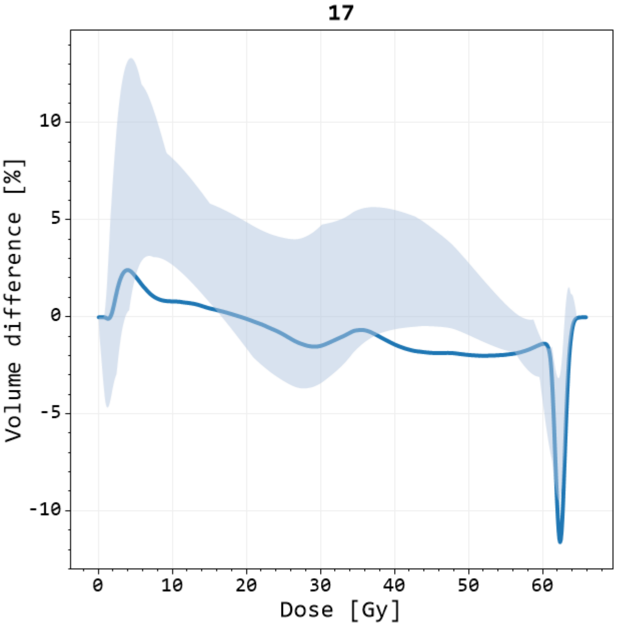
17
Volume difference [%]
Dose [Gy]

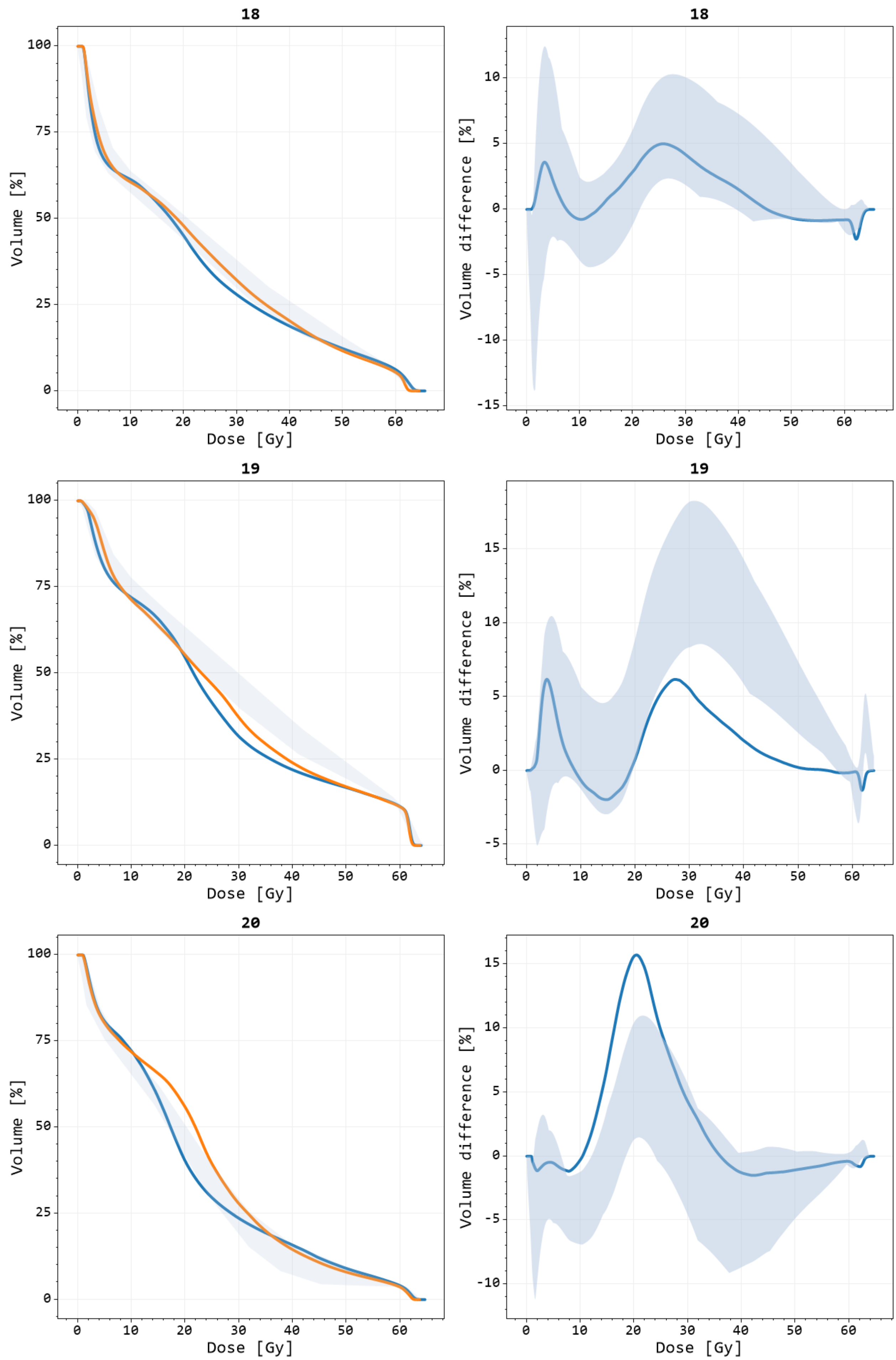
18
Volume [%]
Dose [Gy]
18
Volume difference [%]
Dose [Gy]
19
Volume [%]
Dose [Gy]
19
Volume difference [%]
Dose [Gy]
20
Volume [%]
Dose [Gy]
20
Volume difference [%]
Dose [Gy]

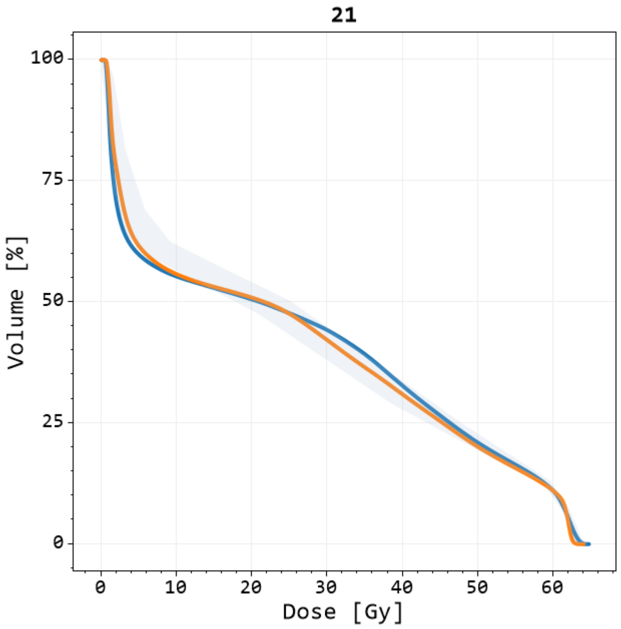
21
Volume [%]
Dose [Gy]
100
75
50
25
0
0
10
20
30
40
50
60

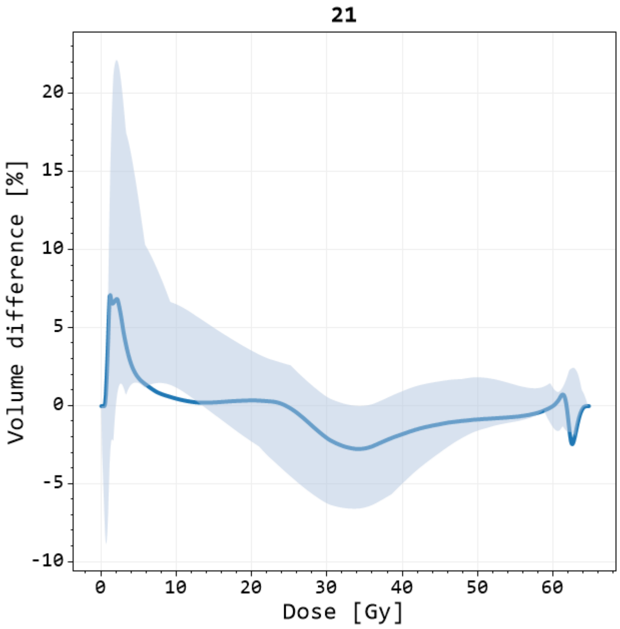
21
Volume difference [%]
Dose [Gy]
20
15
10
5
0
-5
-10
0
10
20
30
40
50
60

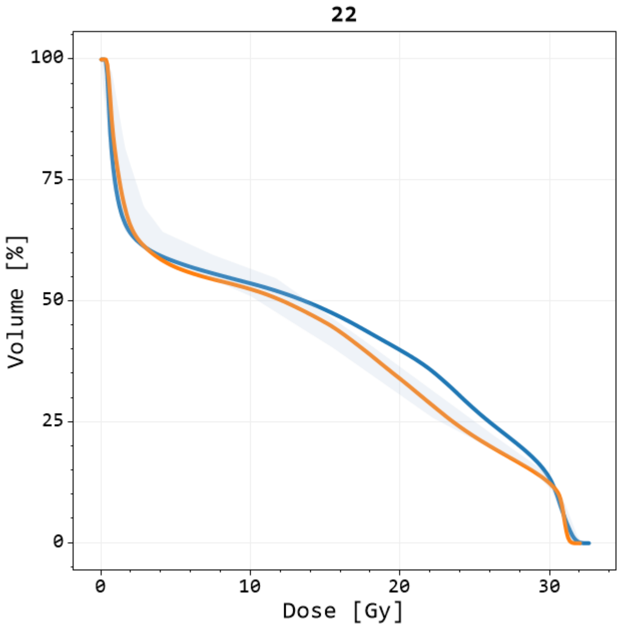
22
Volume [%]
Dose [Gy]
100
75
50
25
0
0
10
20
30

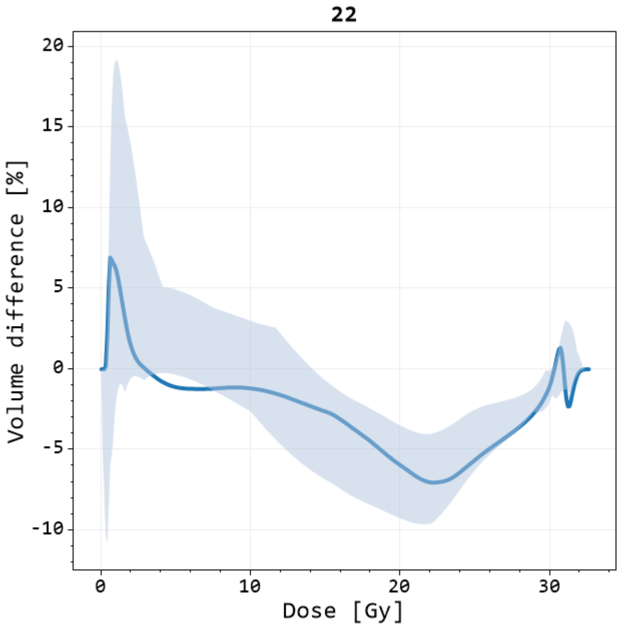
22
Volume difference [%]
Dose [Gy]
20
15
10
5
0
-5
-10
0
10
20
30

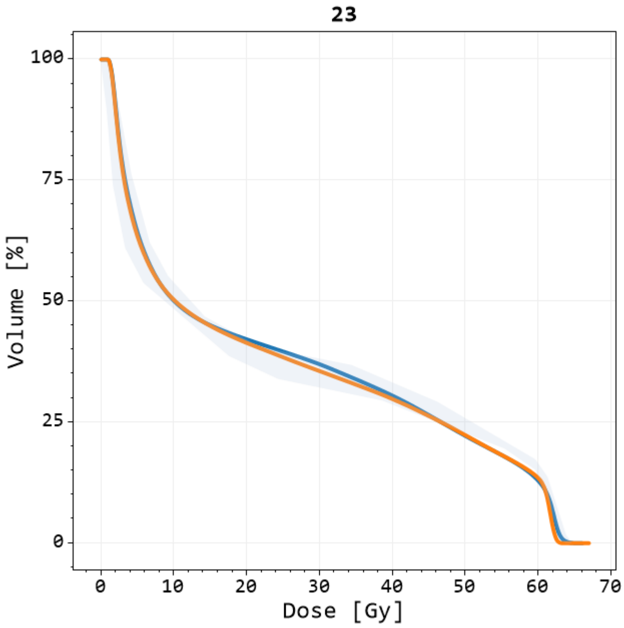
23
Volume [%]
Dose [Gy]
100
75
50
25
0
0
10
20
30
40
50
60
70

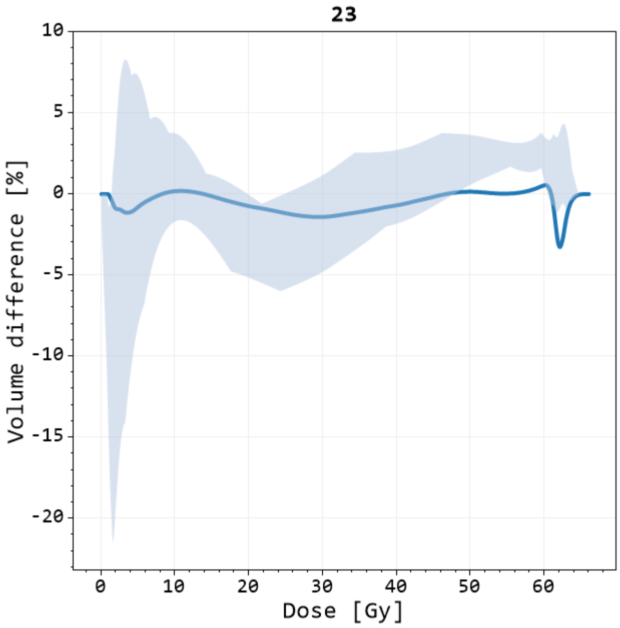
23
Volume difference [%]
Dose [Gy]
10
5
0
-5
-10
-15
-20
0
10
20
30
40
50
60

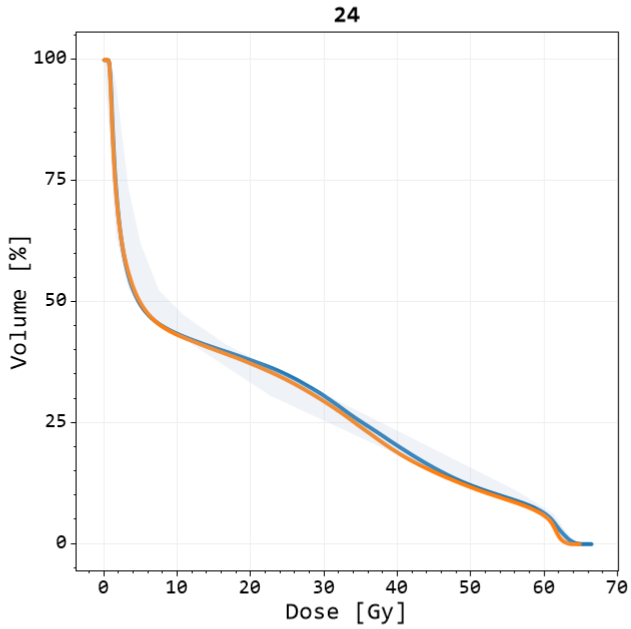
24
Volume [%]
100
75
50
25
0
0
10
20
30
40
50
60
70
Dose [Gy]

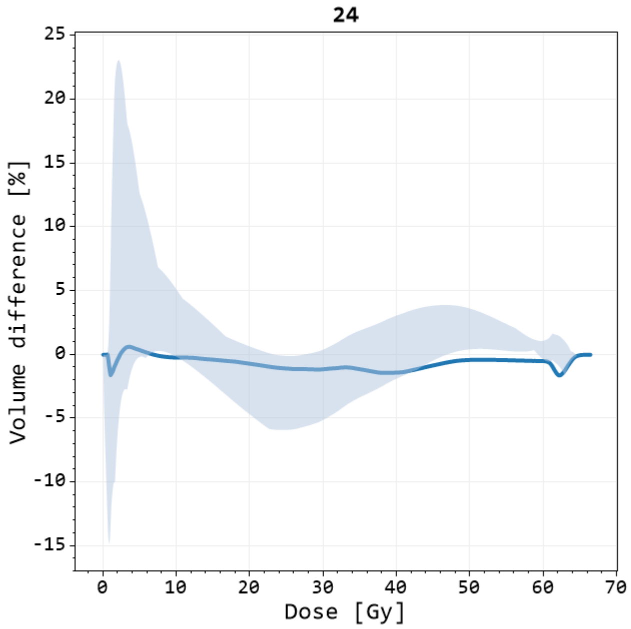
24
Volume difference [%]
25
20
15
10
5
0
-5
-10
-15
0
10
20
30
40
50
60
70
Dose [Gy]

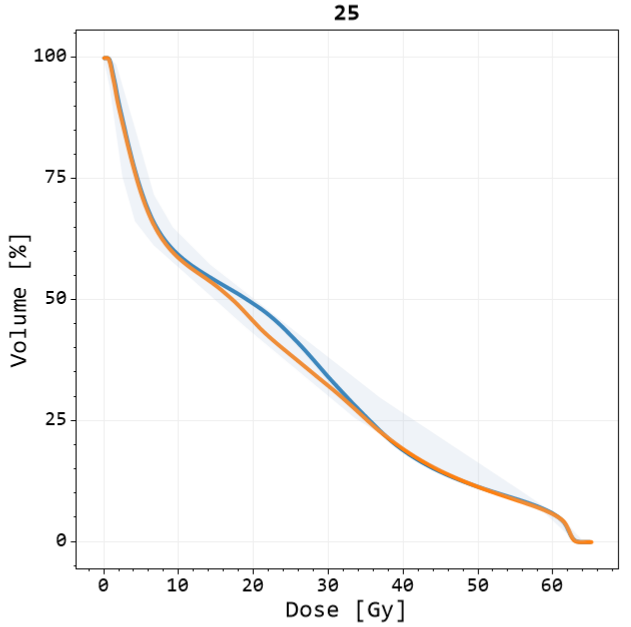
25
Volume [%]
100
75
50
25
0
0
10
20
30
40
50
60
Dose [Gy]

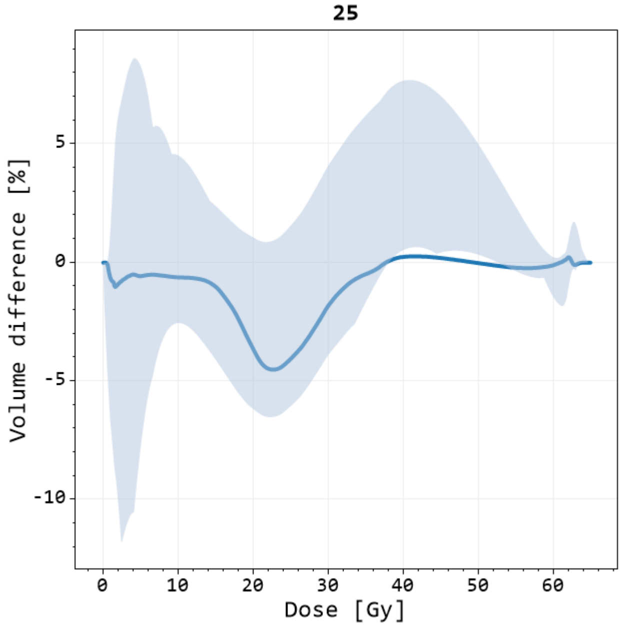
25
Volume difference [%]
5
0
-5
-10
0
10
20
30
40
50
60
Dose [Gy]

# SpinalCord

## DVH Volume difference

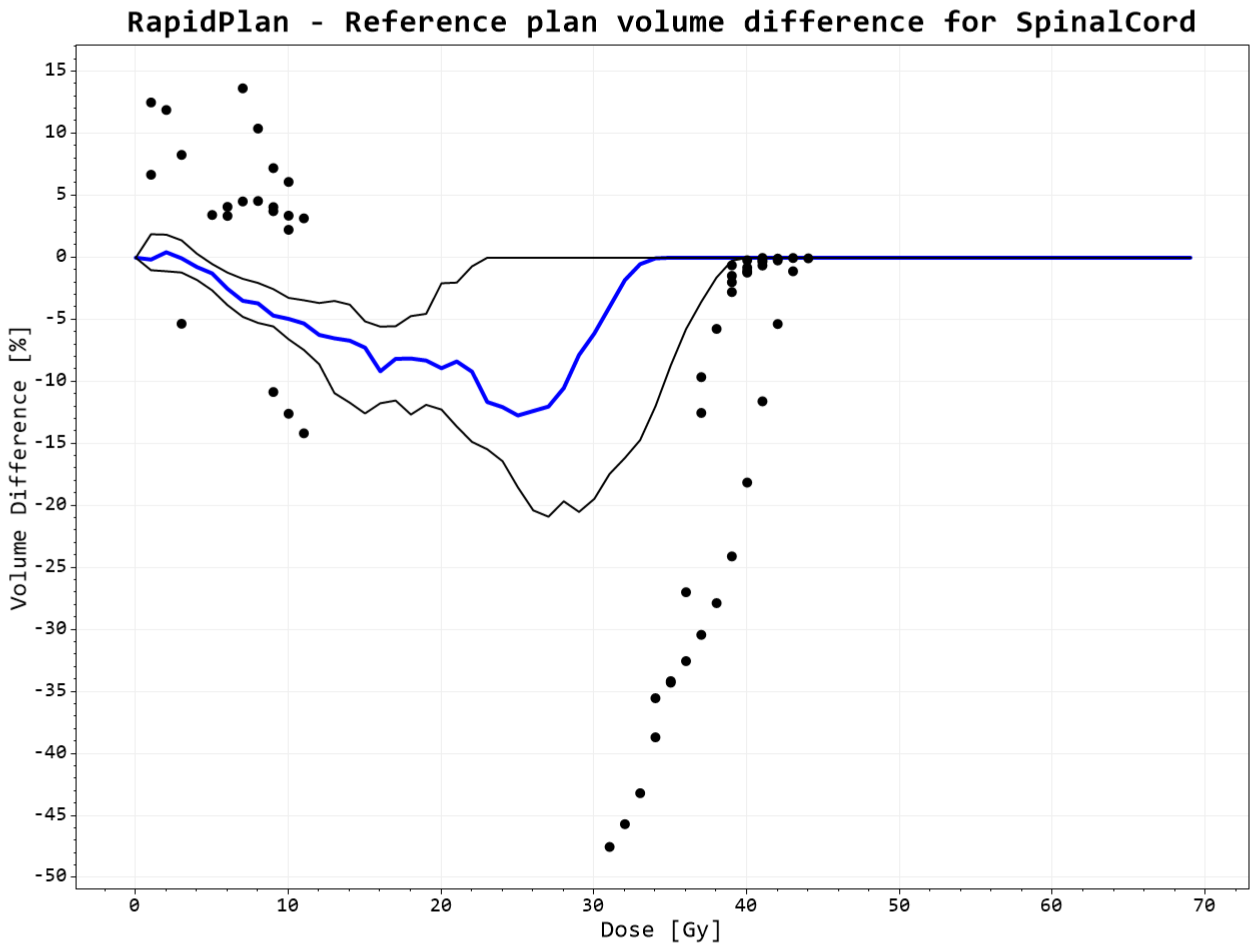


## Dose-volume metric summary table

| Metric | Reference Plan | RapidPlan | Difference |
|---|---|---|---|
| DMax[Gy] | 34.7 [24.8,40.1] | 30.1 [20.5,33.3] | -4.6 [-8.4,-0.7] (p = 0.998) (0) |
| V50Gy[%] | 0.0 [0.0,0.0] | 0.0 [0.0,0.0] | 0.0 [0.0,0.0] (p = 1.000) (0) |

## Dose-volume metric box whisker plots

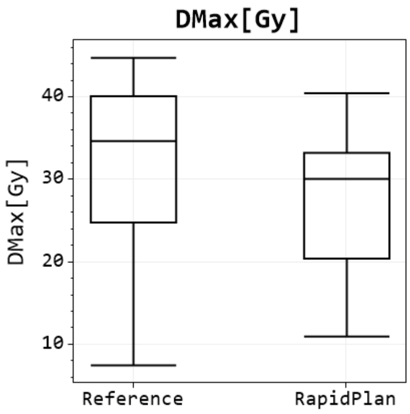


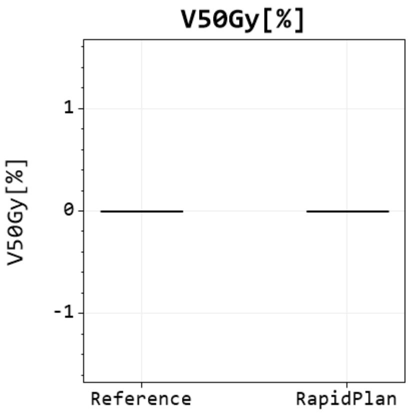

## Dose-volume metric differences by plan

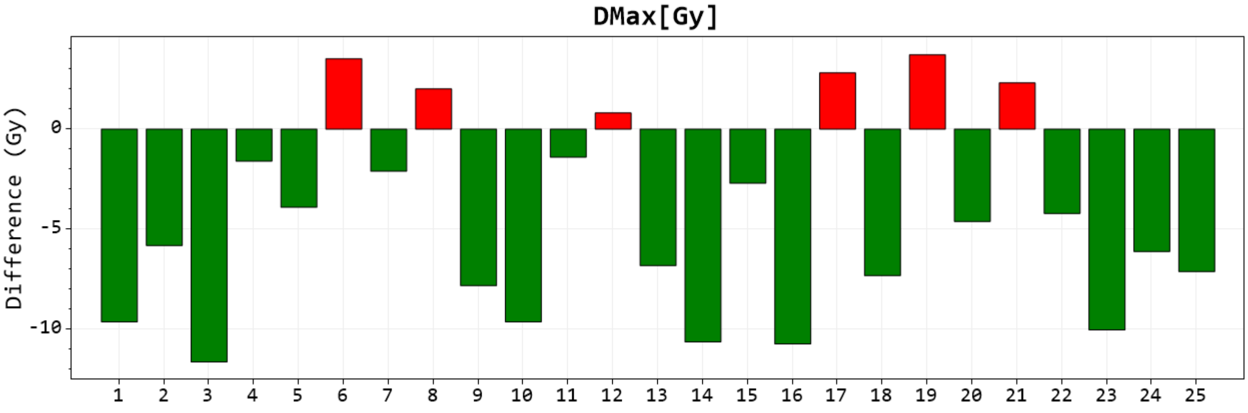


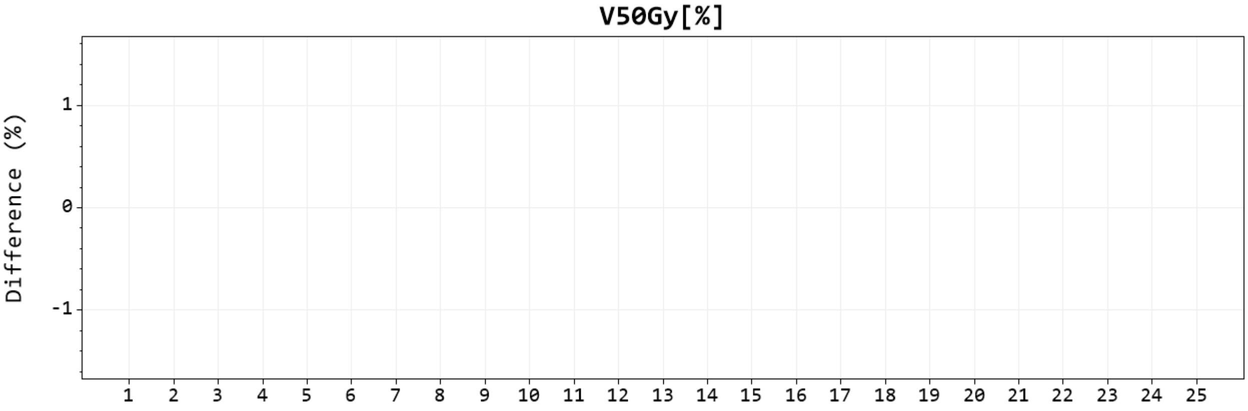

## Dose-volume histograms

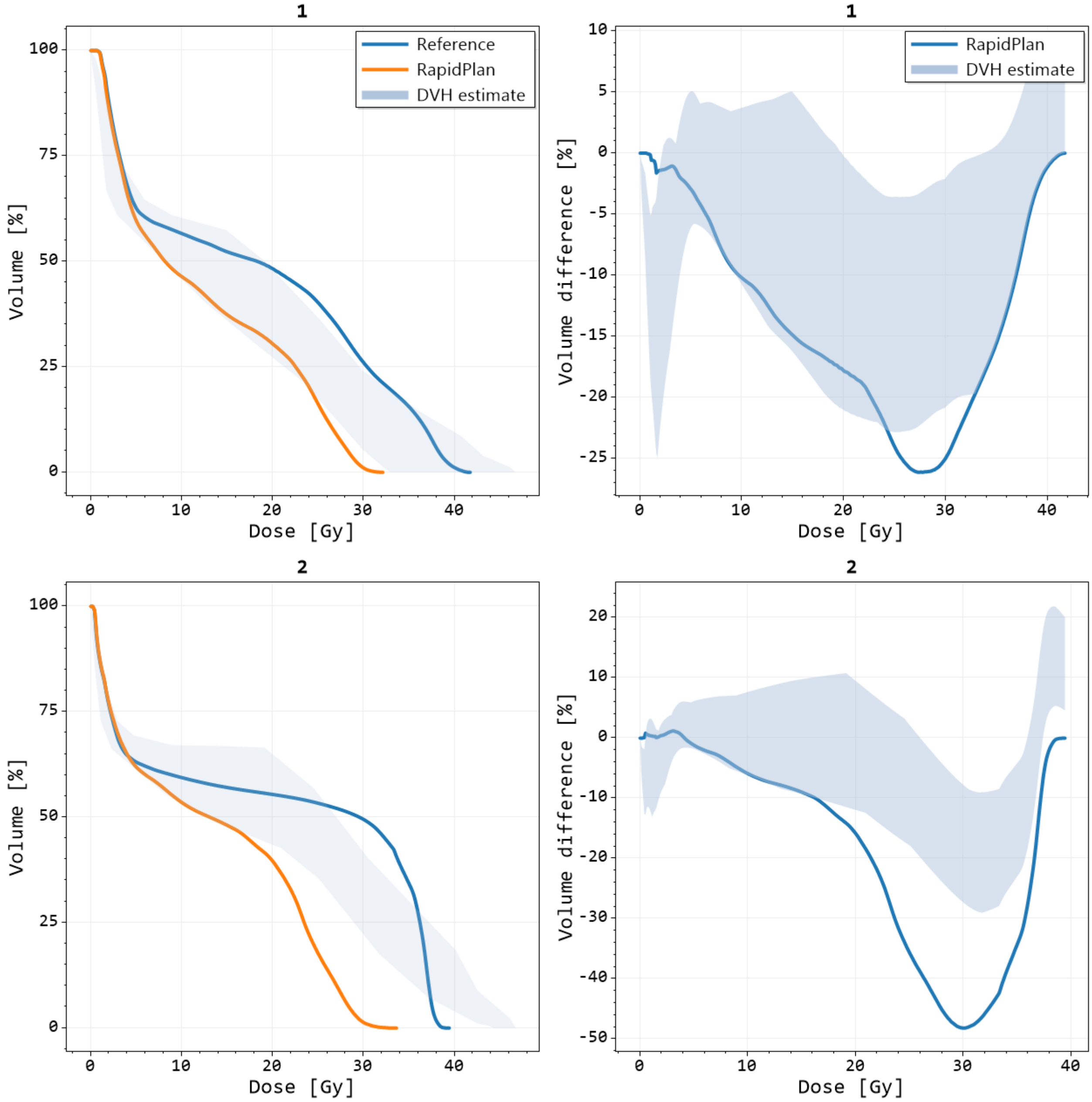

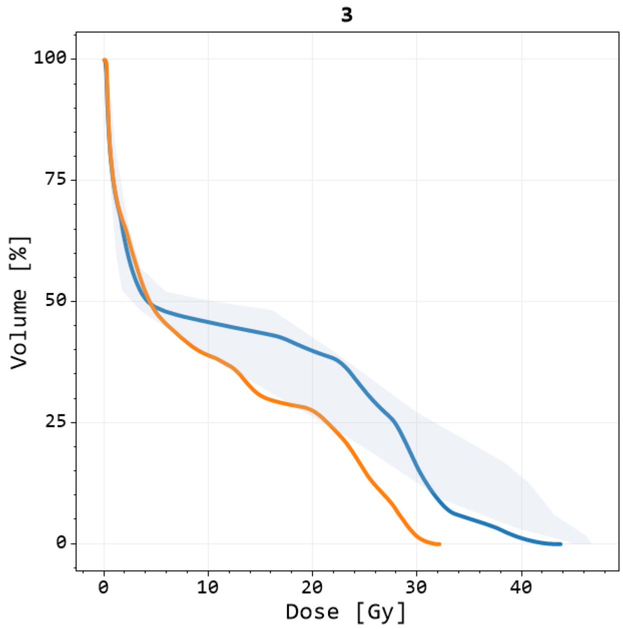
3
100
75
50
25
0
Volume [%]
0
10
20
30
40
Dose [Gy]

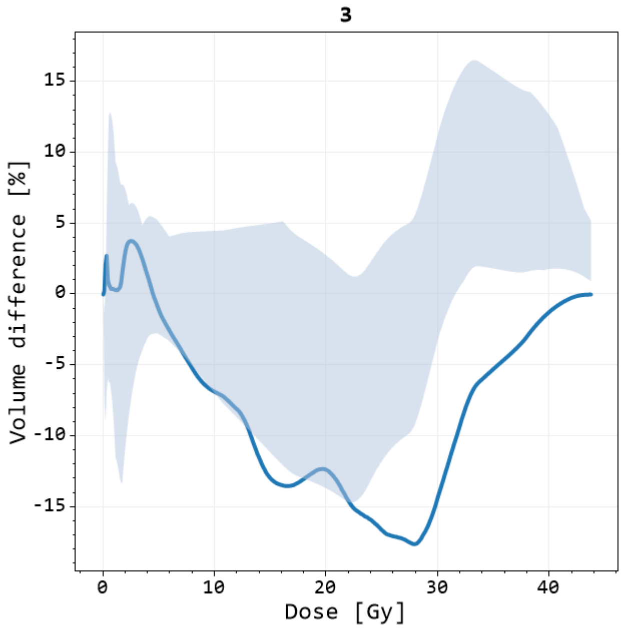
3
15
10
5
0
-5
-10
-15
Volume difference [%]
0
10
20
30
40
Dose [Gy]

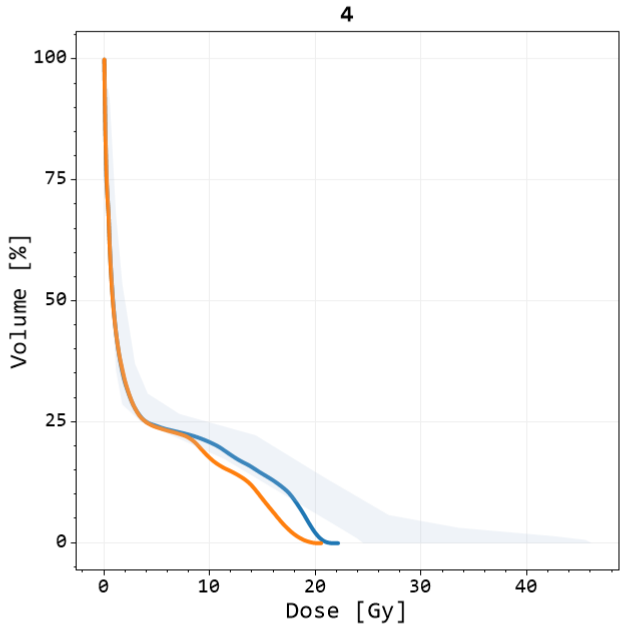
4
100
75
50
25
0
Volume [%]
0
10
20
30
40
Dose [Gy]

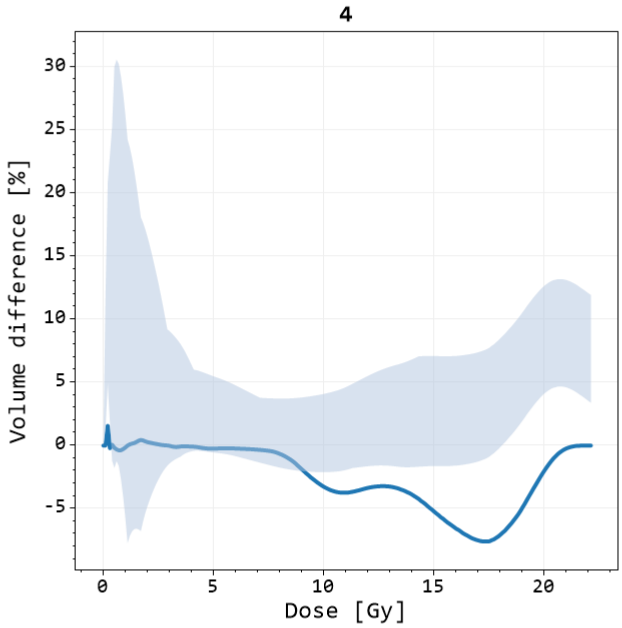
4
30
25
20
15
10
5
0
-5
Volume difference [%]
0
5
10
15
20
Dose [Gy]

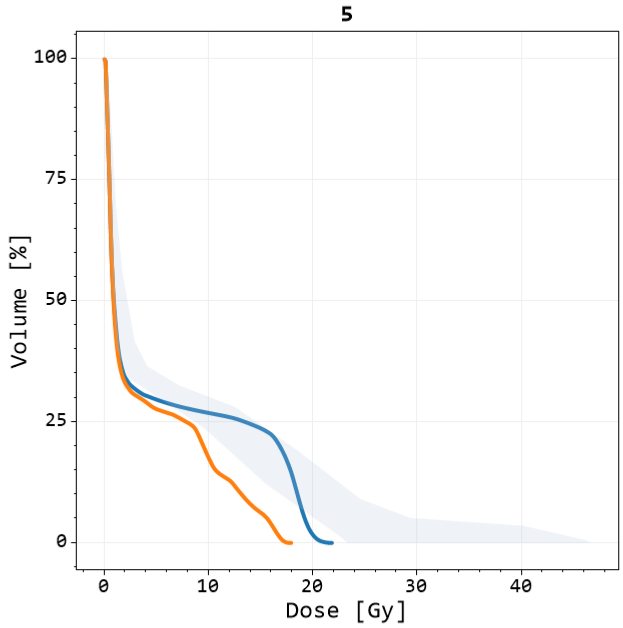
5
100
75
50
25
0
Volume [%]
0
10
20
30
40
Dose [Gy]

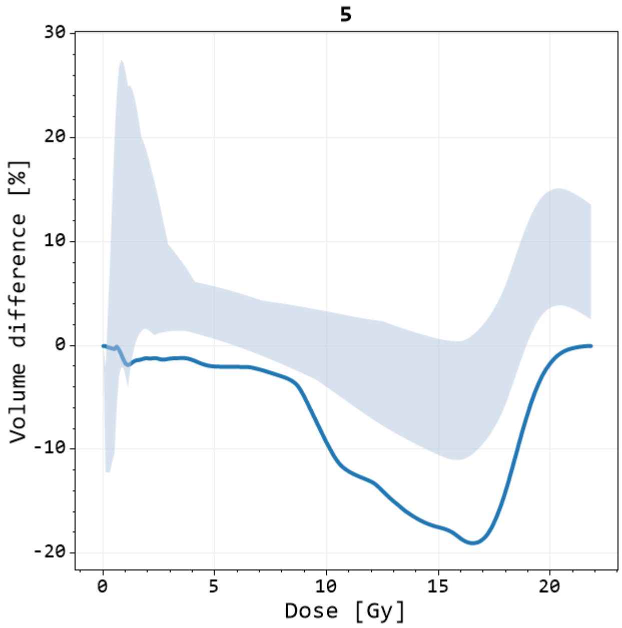
5
30
20
10
0
-10
-20
Volume difference [%]
0
5
10
15
20
Dose [Gy]

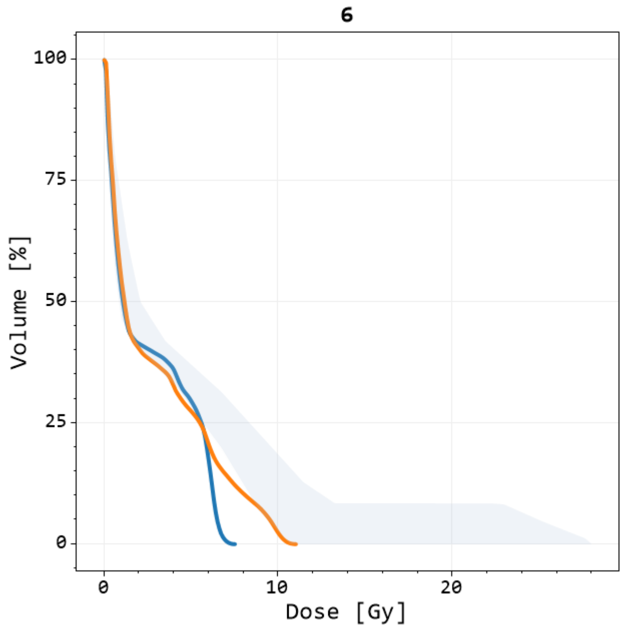
6
100
75
50
25
0
Volume [%]
0
10
20
Dose [Gy]

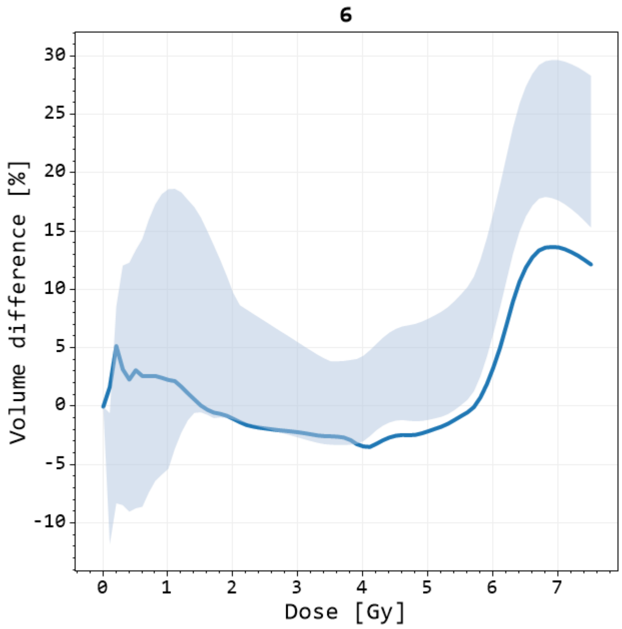
6
30
25
20
15
10
5
0
-5
-10
Volume difference [%]
0
1
2
3
4
5
6
7
Dose [Gy]

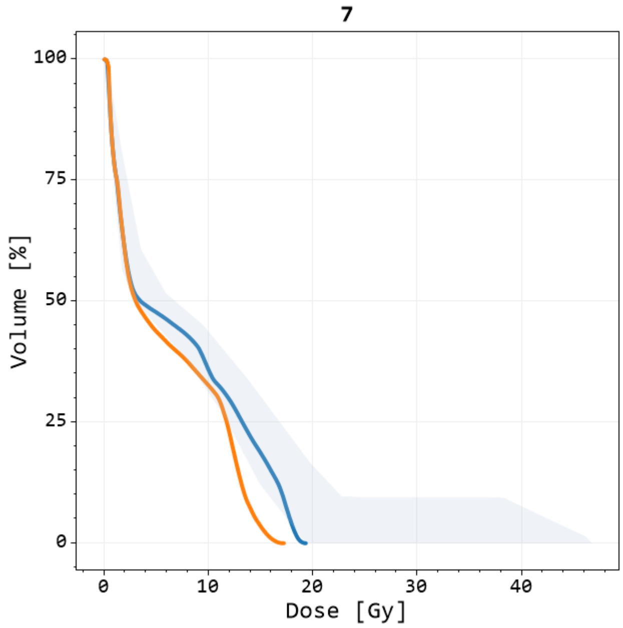
7
100
75
50
25
0
Volume [%]
0
10
20
30
40
Dose [Gy]

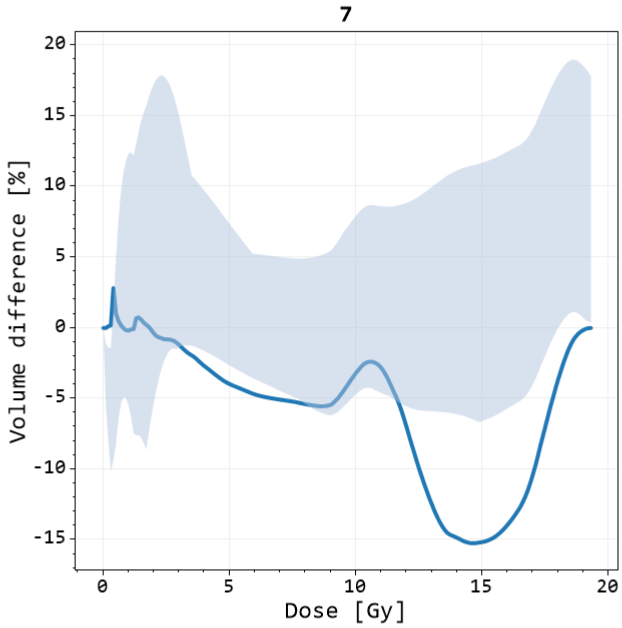
7
20
15
10
5
0
-5
-10
-15
Volume difference [%]
0
5
10
15
20
Dose [Gy]

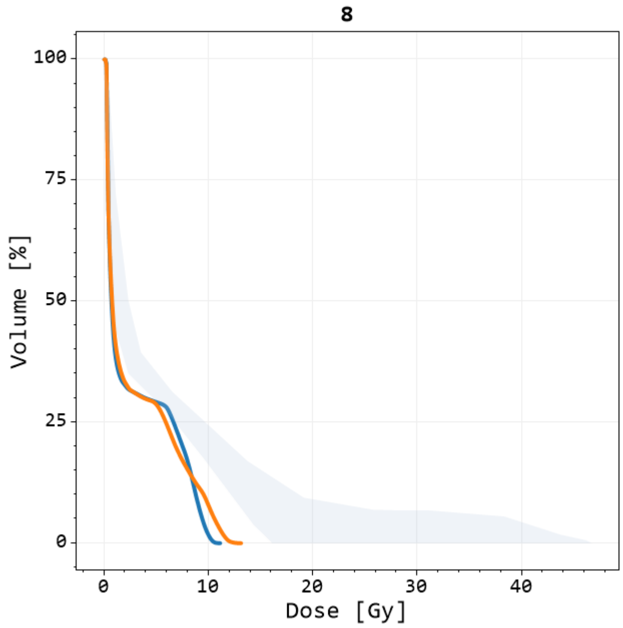
8
100
75
50
25
0
Volume [%]
0
10
20
30
40
Dose [Gy]

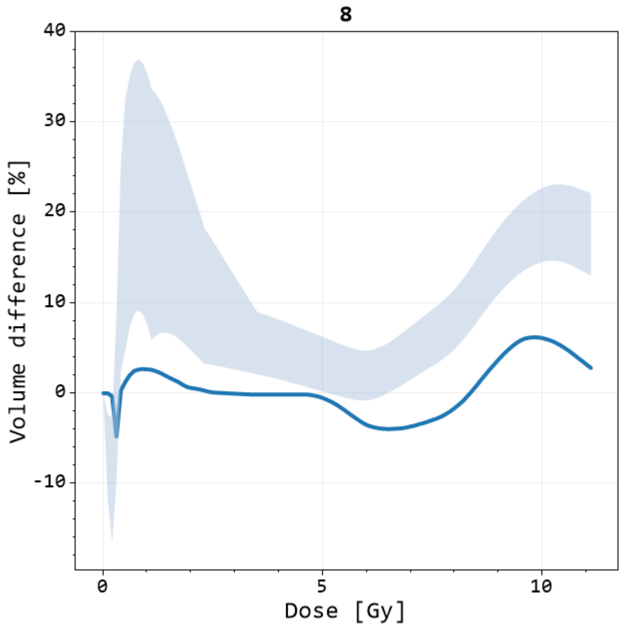
8
40
30
20
10
0
-10
Volume difference [%]
0
5
10
Dose [Gy]

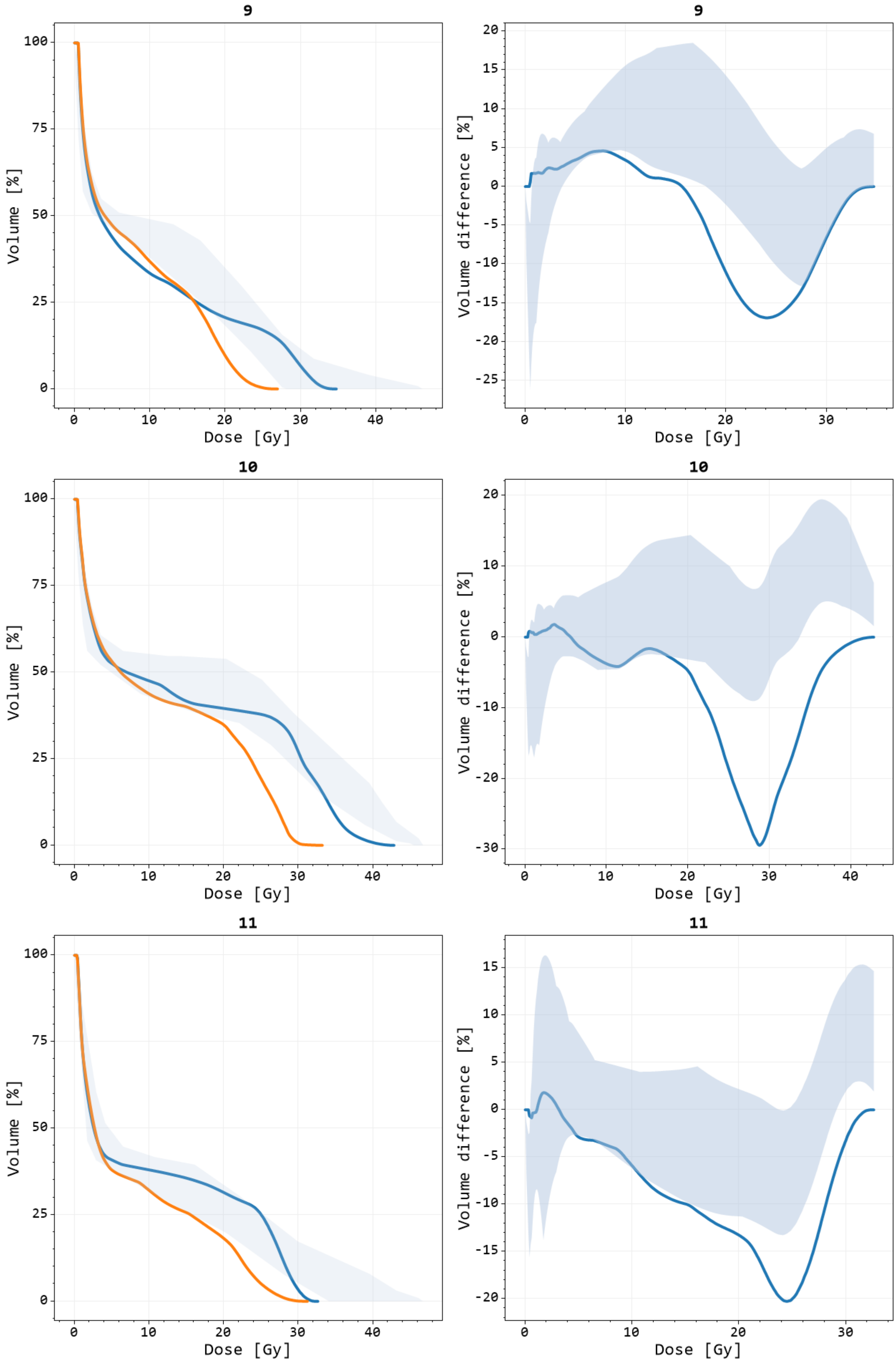
9
Volume [%]
Dose [Gy]
9
Volume difference [%]
Dose [Gy]
10
Volume [%]
Dose [Gy]
10
Volume difference [%]
Dose [Gy]
11
Volume [%]
Dose [Gy]
11
Volume difference [%]
Dose [Gy]

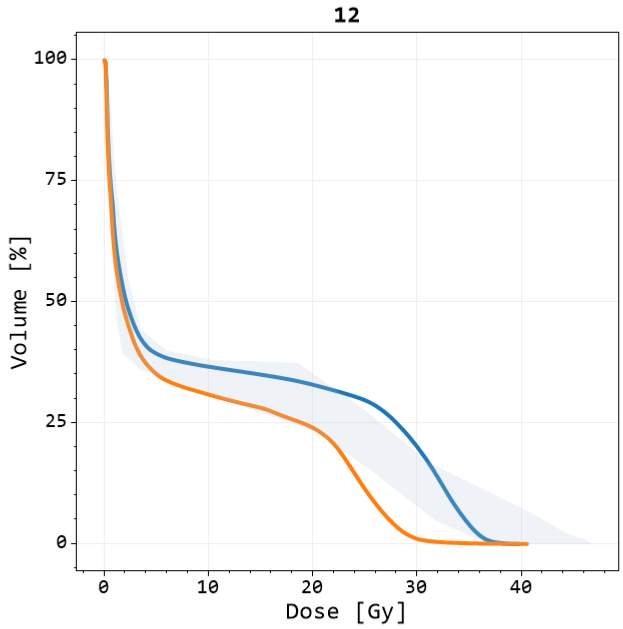
12
Volume [%]
Dose [Gy]

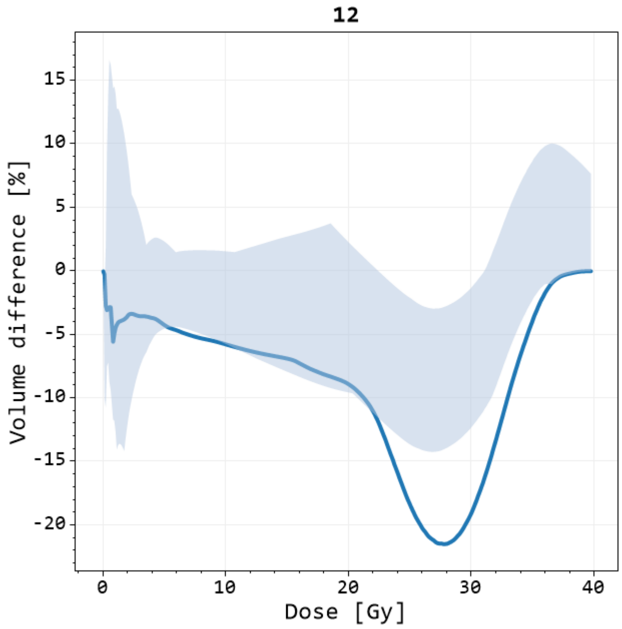
12
Volume difference [%]
Dose [Gy]

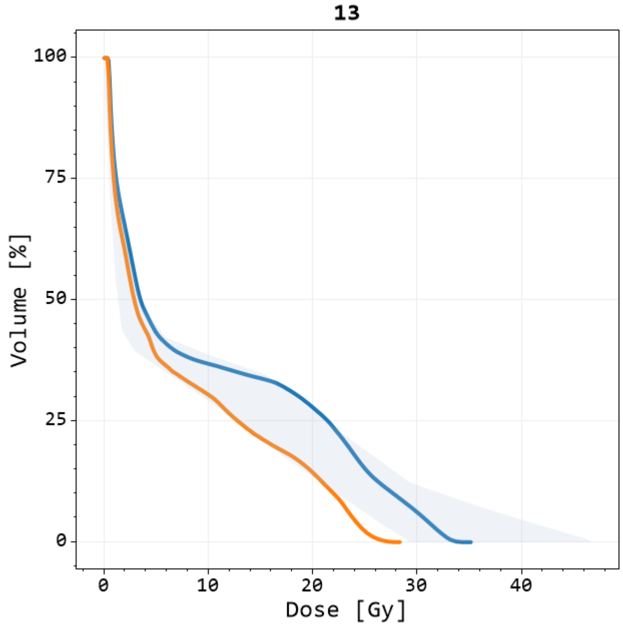
13
Volume [%]
Dose [Gy]

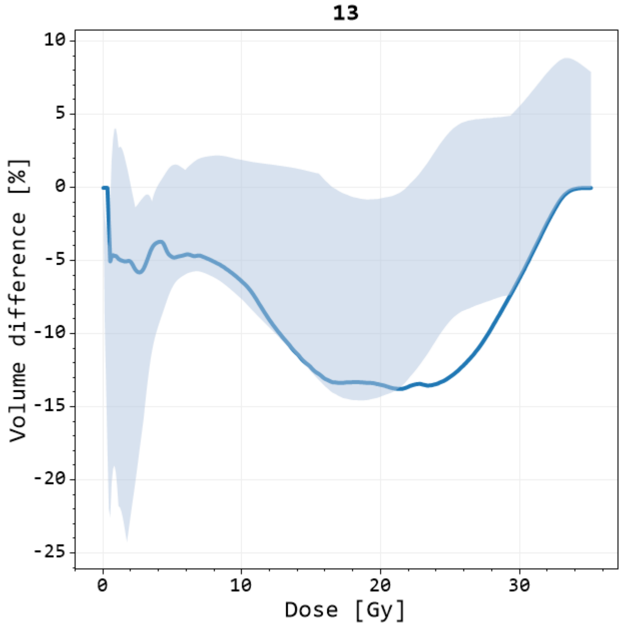
13
Volume difference [%]
Dose [Gy]

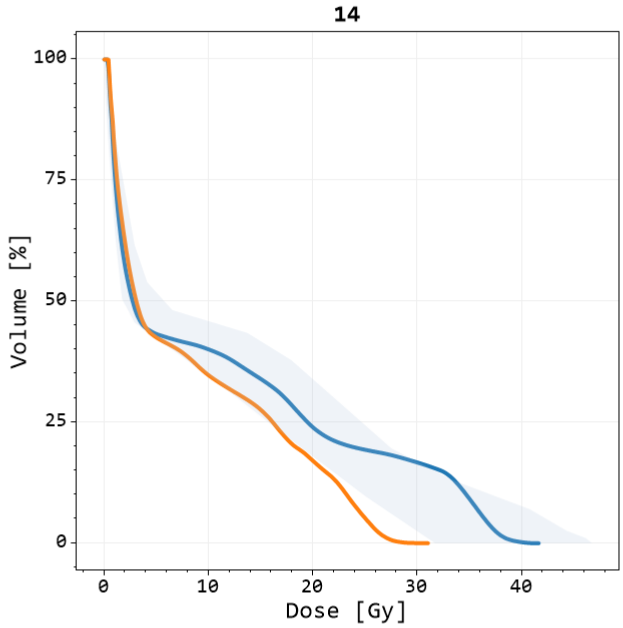
14
Volume [%]
Dose [Gy]

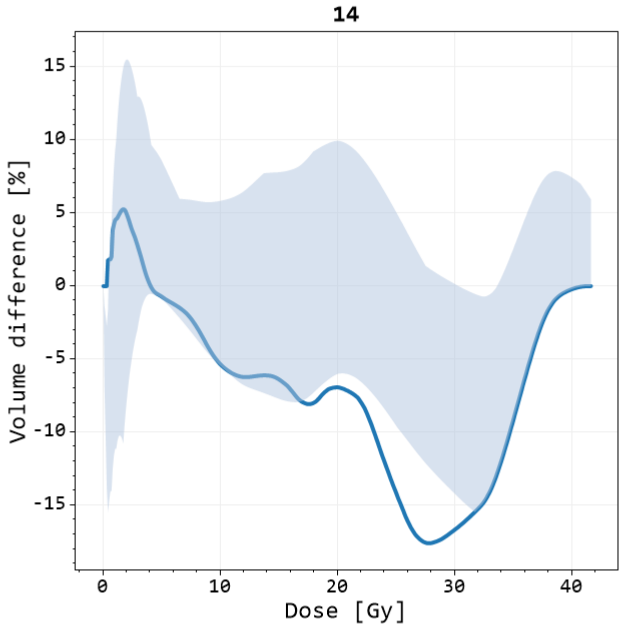
14
Volume difference [%]
Dose [Gy]

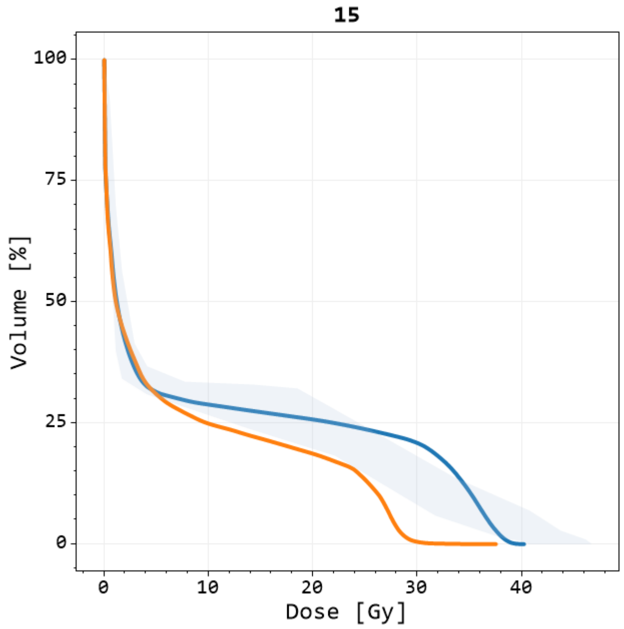
15
Volume [%]
Dose [Gy]
0
25
50
75
100
0
10
20
30
40

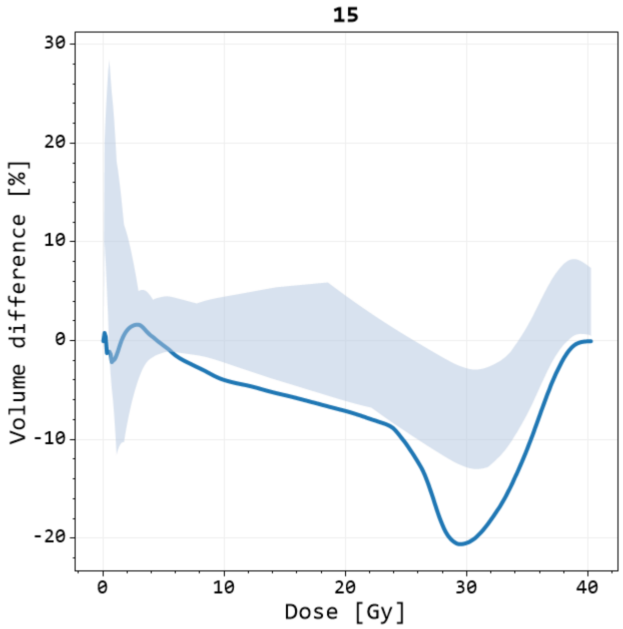
15
Volume difference [%]
Dose [Gy]
-20
-10
0
10
20
30
0
10
20
30
40

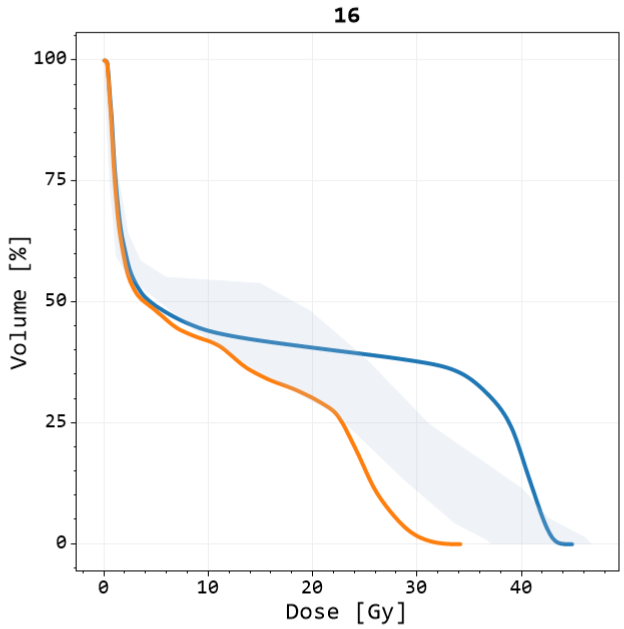
16
Volume [%]
Dose [Gy]
0
25
50
75
100
0
10
20
30
40

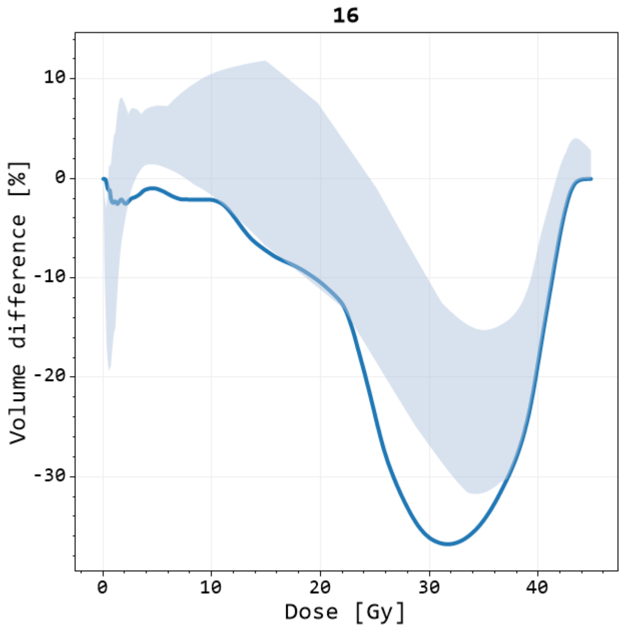
16
Volume difference [%]
Dose [Gy]
-30
-20
-10
0
10
0
10
20
30
40

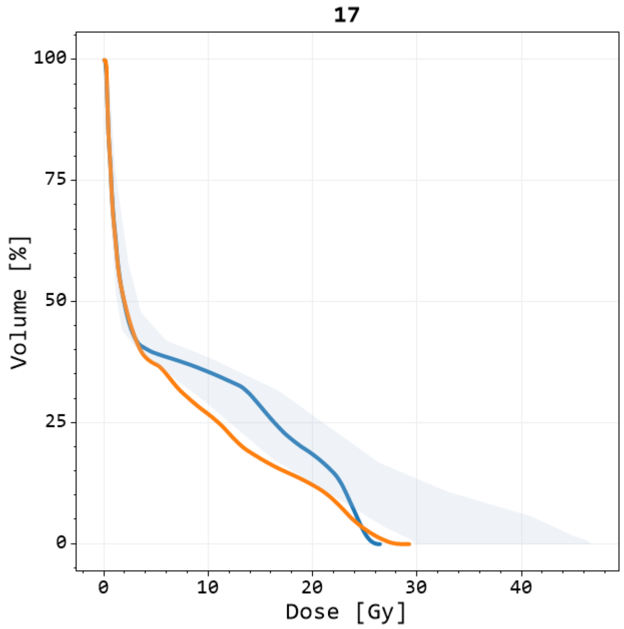
17
Volume [%]
Dose [Gy]
0
25
50
75
100
0
10
20
30
40

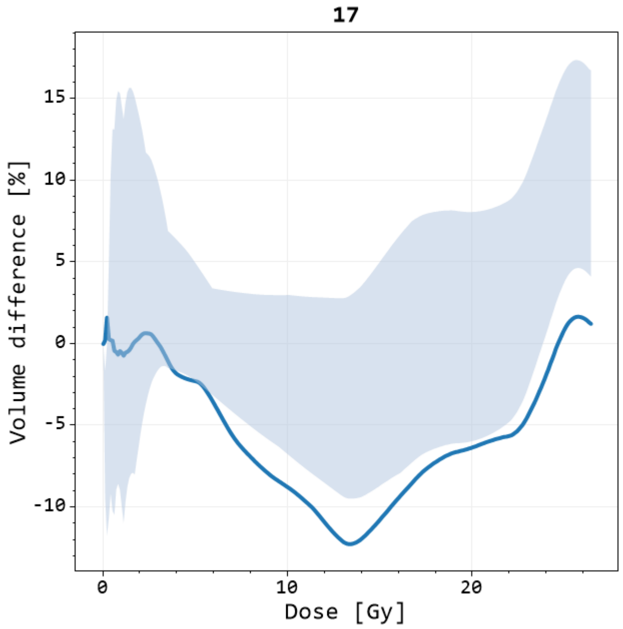
17
Volume difference [%]
Dose [Gy]
-10
-5
0
5
10
15
0
10
20

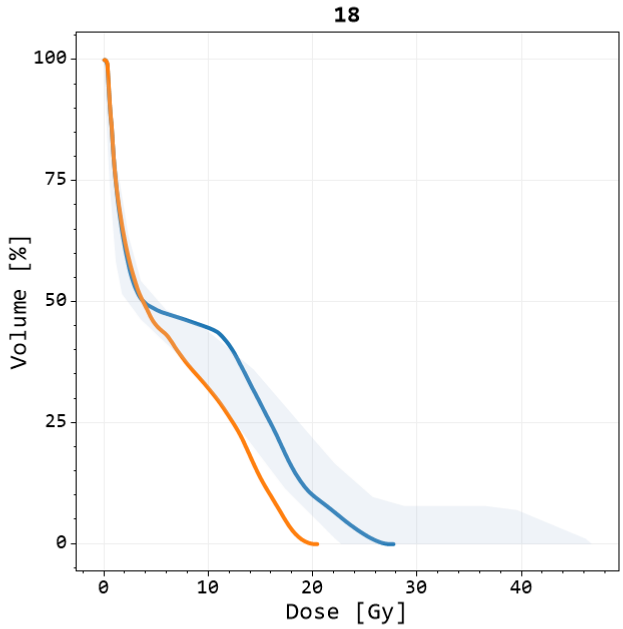
18
100
75
50
25
0
Volume [%]
0
10
20
30
40
Dose [Gy]

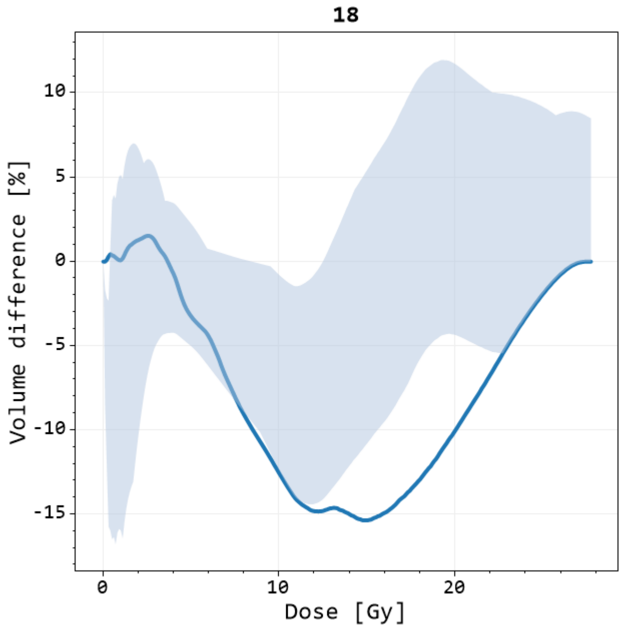
18
10
5
0
-5
-10
-15
Volume difference [%]
0
10
20
Dose [Gy]

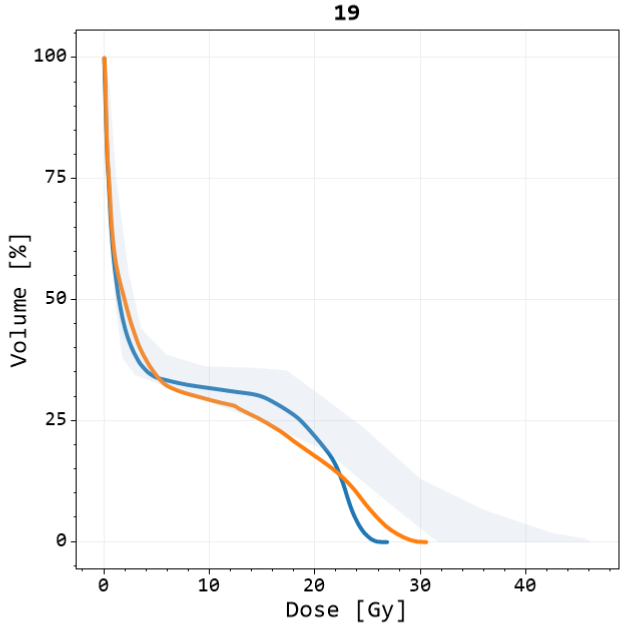
19
100
75
50
25
0
Volume [%]
0
10
20
30
40
Dose [Gy]

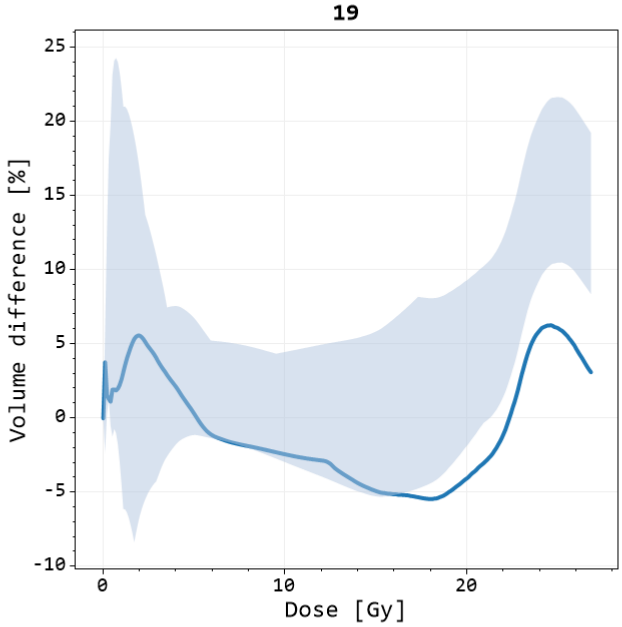
19
25
20
15
10
5
0
-5
-10
Volume difference [%]
0
10
20
Dose [Gy]

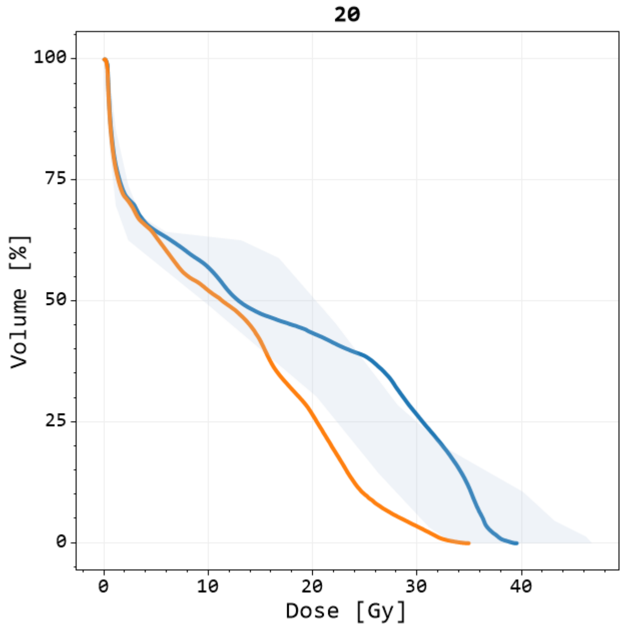
20
100
75
50
25
0
Volume [%]
0
10
20
30
40
Dose [Gy]

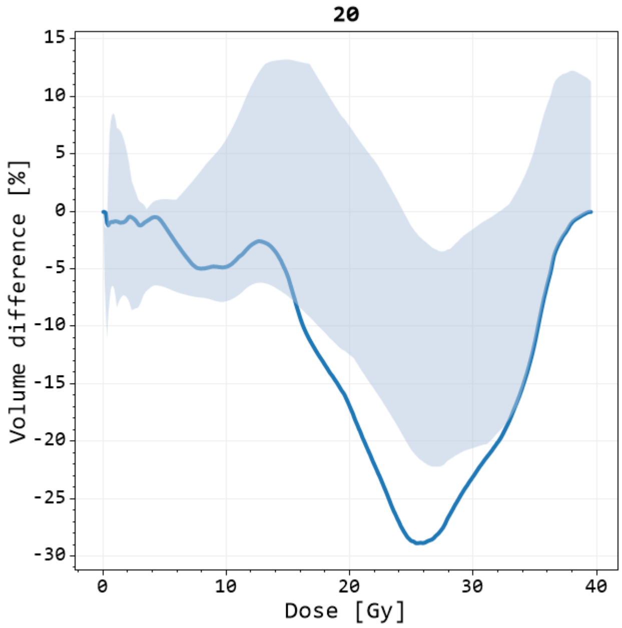
20
15
10
5
0
-5
-10
-15
-20
-25
-30
Volume difference [%]
0
10
20
30
40
Dose [Gy]

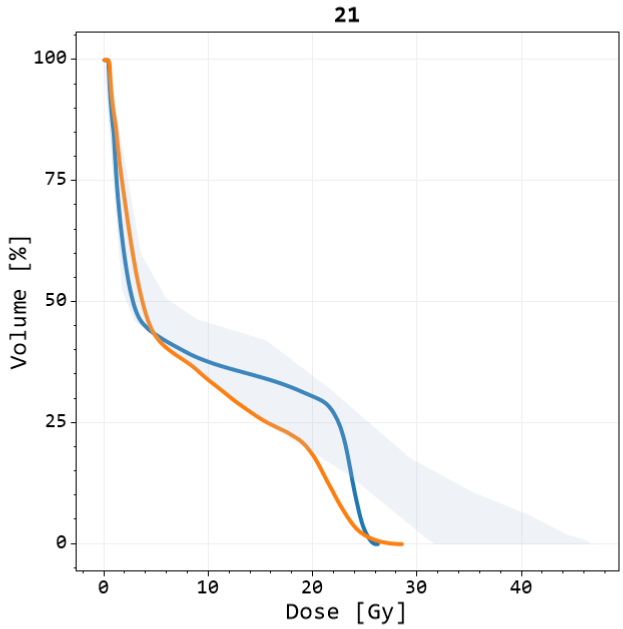
21
Volume [%]
Dose [Gy]
100
75
50
25
0
0
10
20
30
40

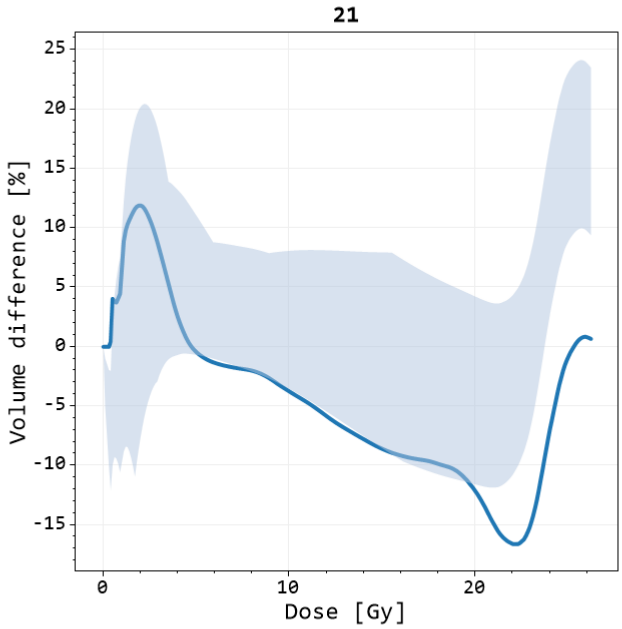
21
Volume difference [%]
Dose [Gy]
25
20
15
10
5
0
-5
-10
-15
0
10
20

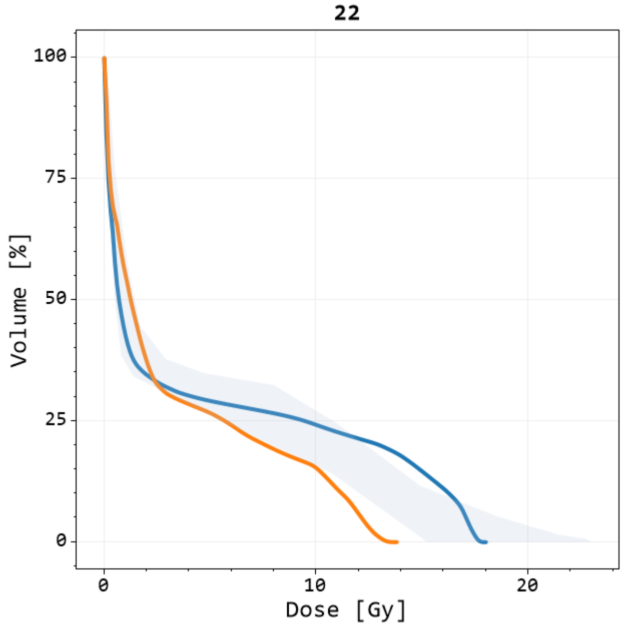
22
Volume [%]
Dose [Gy]
100
75
50
25
0
0
10
20

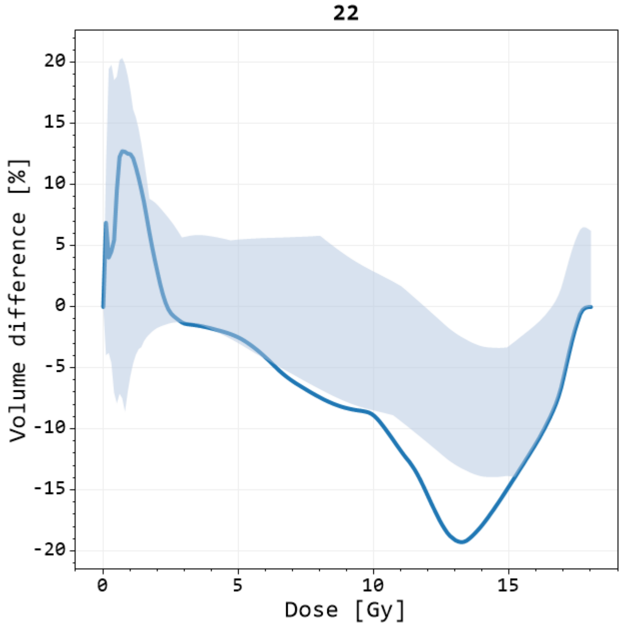
22
Volume difference [%]
Dose [Gy]
20
15
10
5
0
-5
-10
-15
-20
0
5
10
15

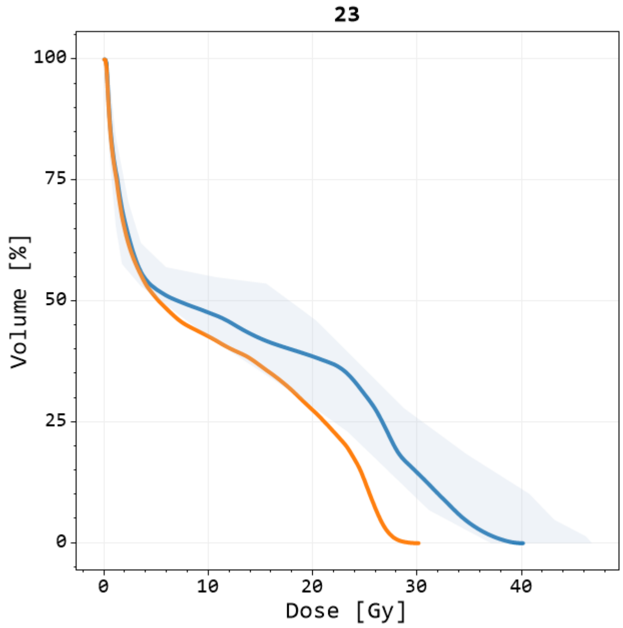
23
Volume [%]
Dose [Gy]
100
75
50
25
0
0
10
20
30
40

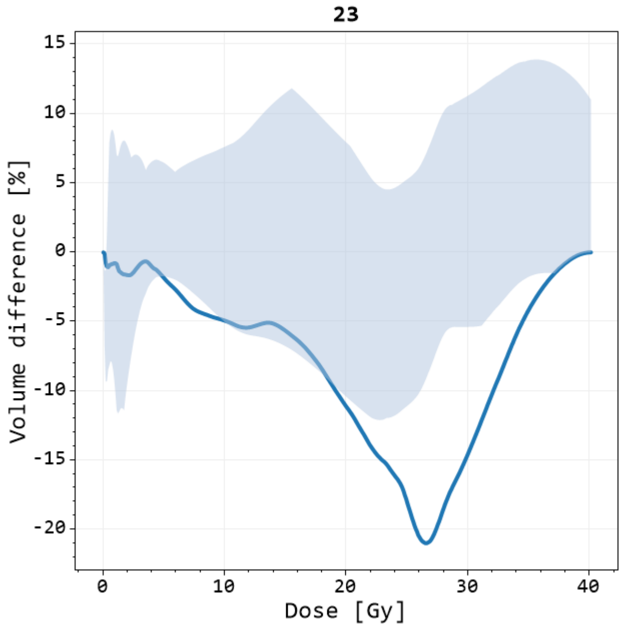
23
Volume difference [%]
Dose [Gy]
15
10
5
0
-5
-10
-15
-20
0
10
20
30
40

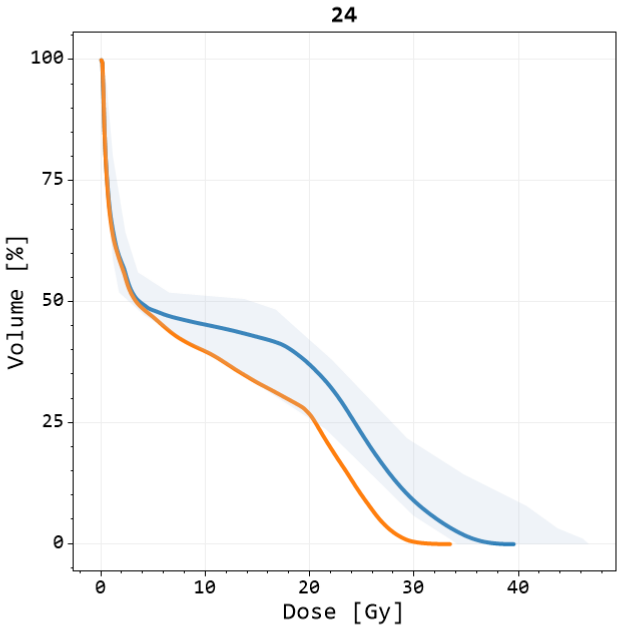
24
Volume [%]
Dose [Gy]

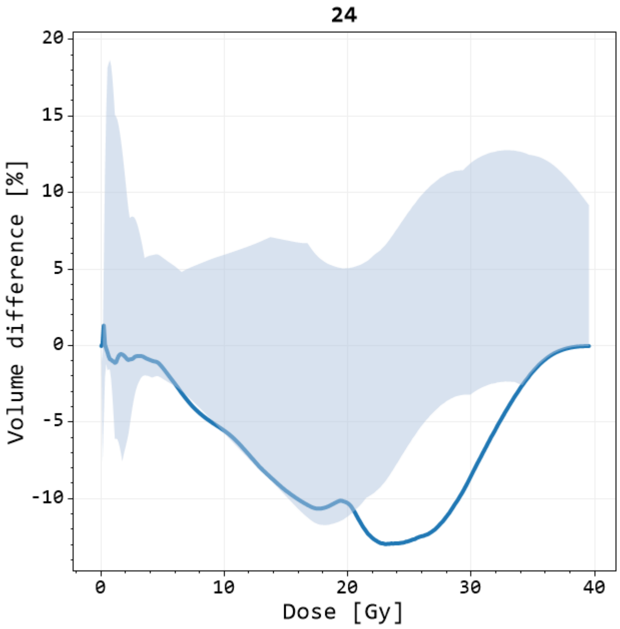
24
Volume difference [%]
Dose [Gy]

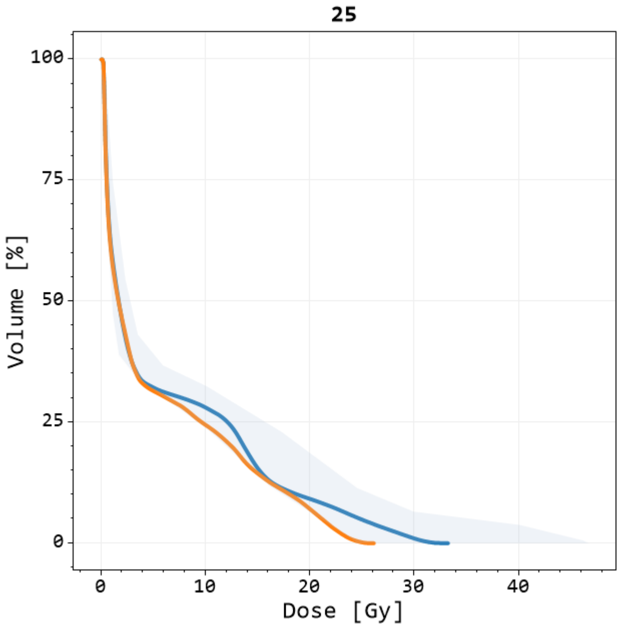
25
Volume [%]
Dose [Gy]

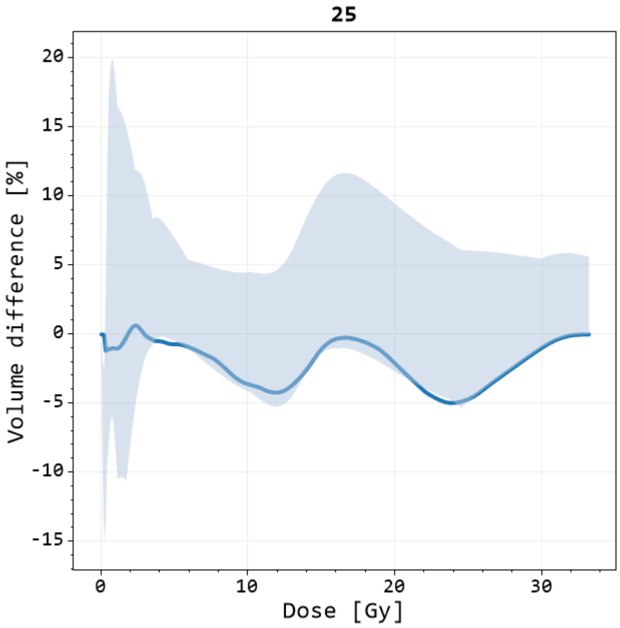
25
Volume difference [%]
Dose [Gy]

# SpinalCord_PRV05

## DVH Volume difference

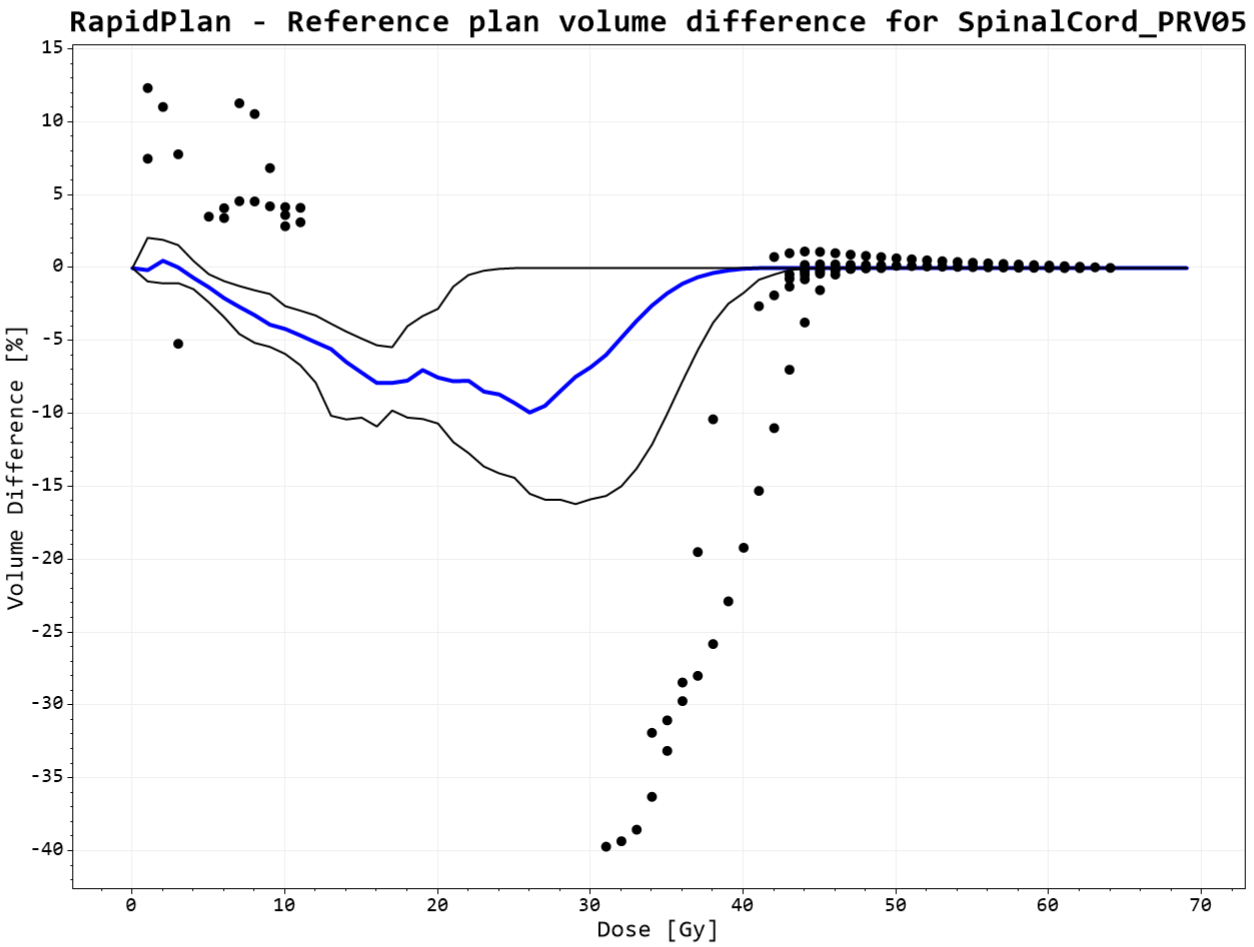


## Dose-volume metric summary table

| Metric | Reference Plan | RapidPlan | Difference |
|---|---|---|---|

**Dose-volume metric box whisker plots**

## Dose-volume metric differences by plan

## Dose-volume histograms

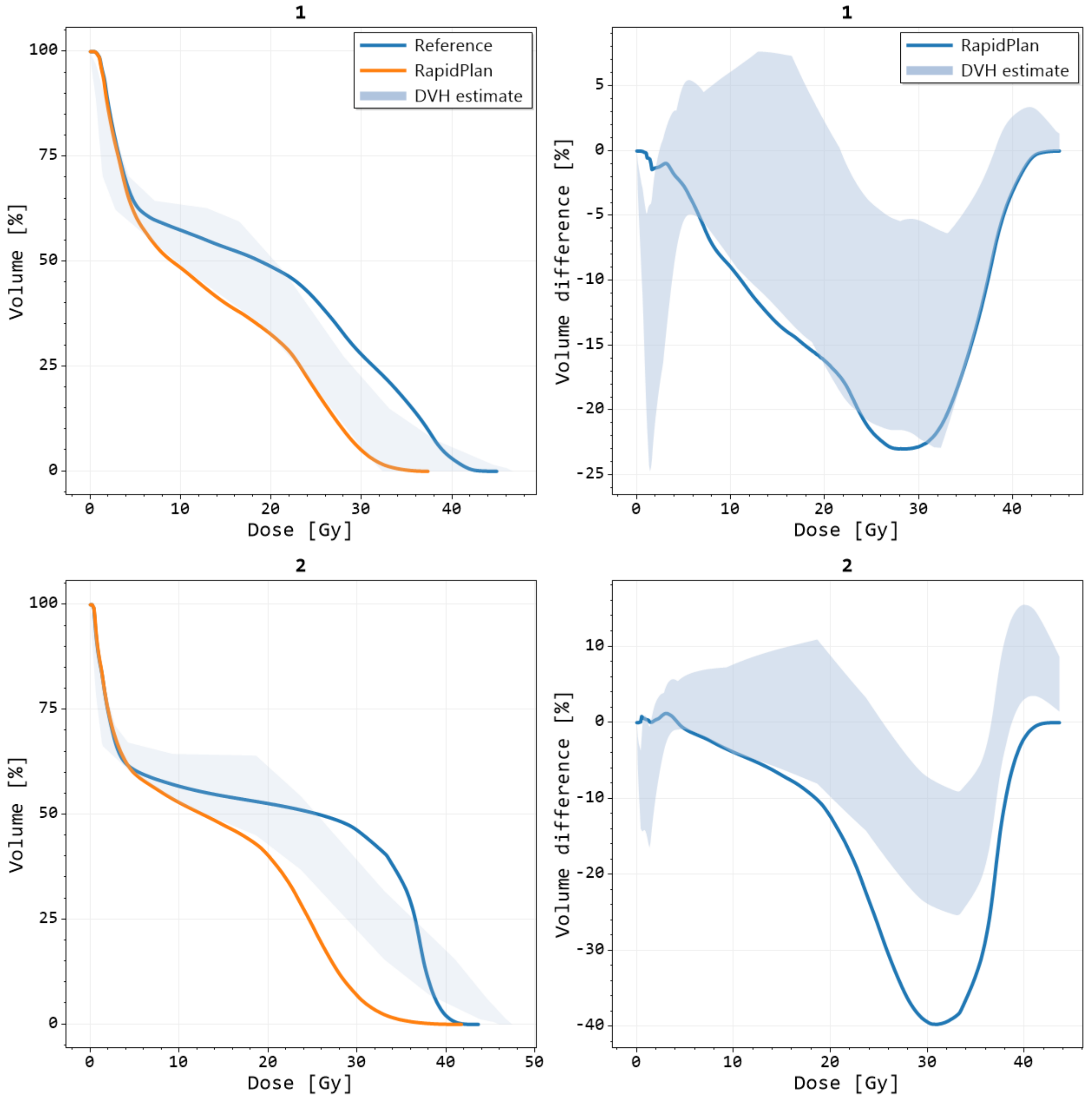

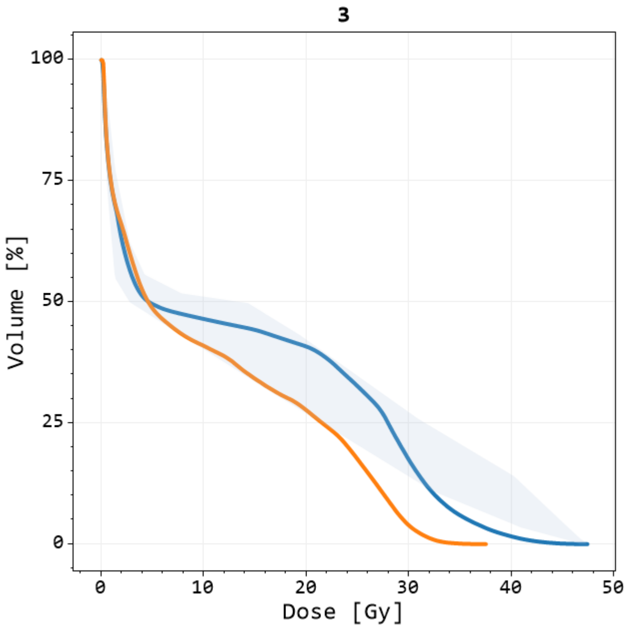
3
100
75
50
25
0
Volume [%]
0
10
20
30
40
50
Dose [Gy]

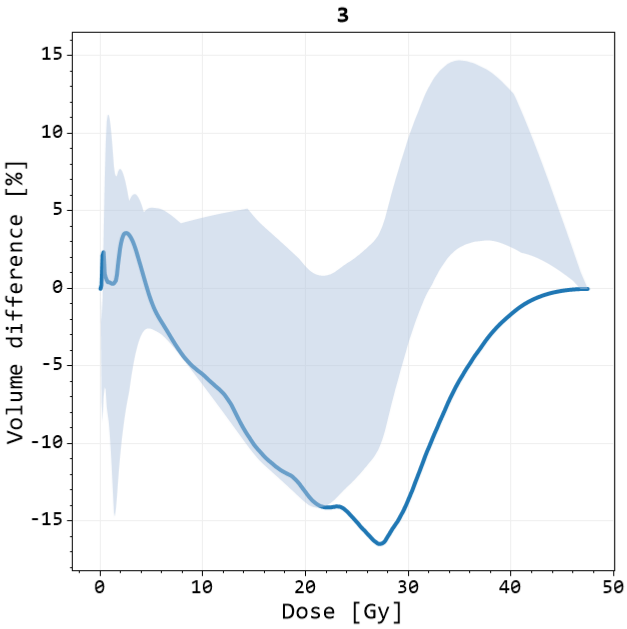
3
15
10
5
0
-5
-10
-15
Volume difference [%]
0
10
20
30
40
50
Dose [Gy]

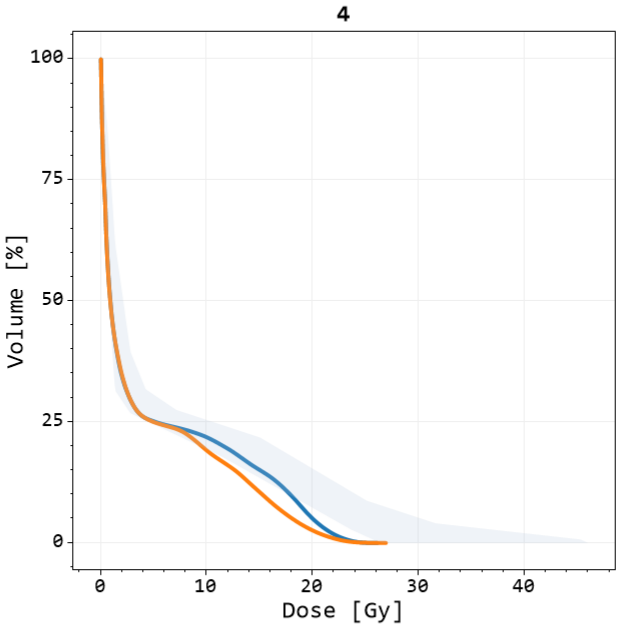
4
100
75
50
25
0
Volume [%]
0
10
20
30
40
Dose [Gy]

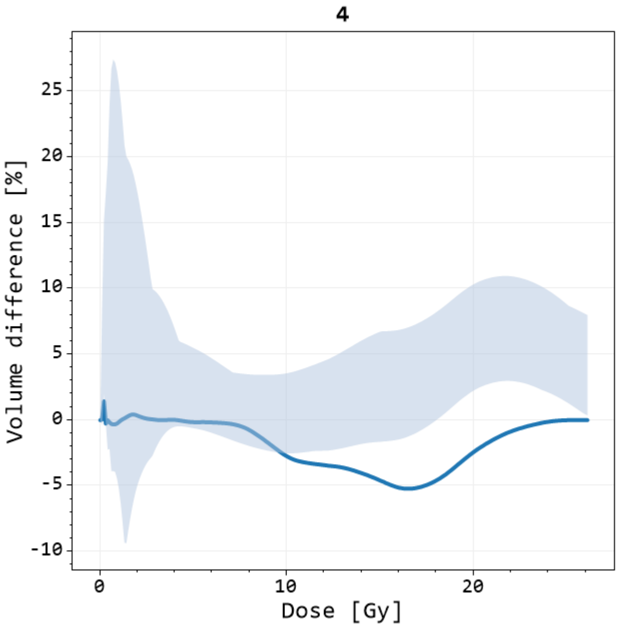
4
25
20
15
10
5
0
-5
-10
Volume difference [%]
0
10
20
Dose [Gy]

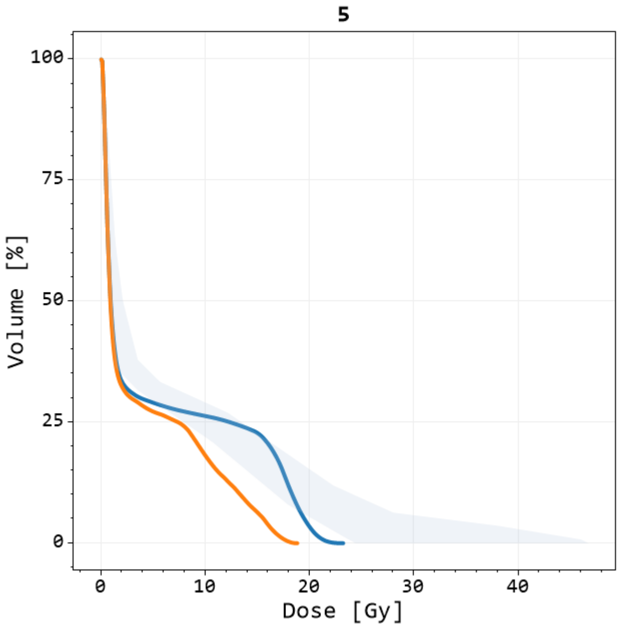
5
100
75
50
25
0
Volume [%]
0
10
20
30
40
Dose [Gy]

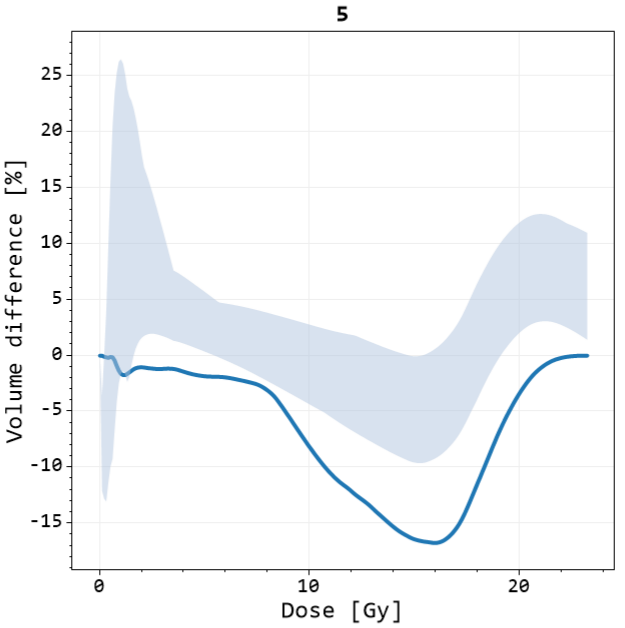
5
25
20
15
10
5
0
-5
-10
-15
Volume difference [%]
0
10
20
Dose [Gy]

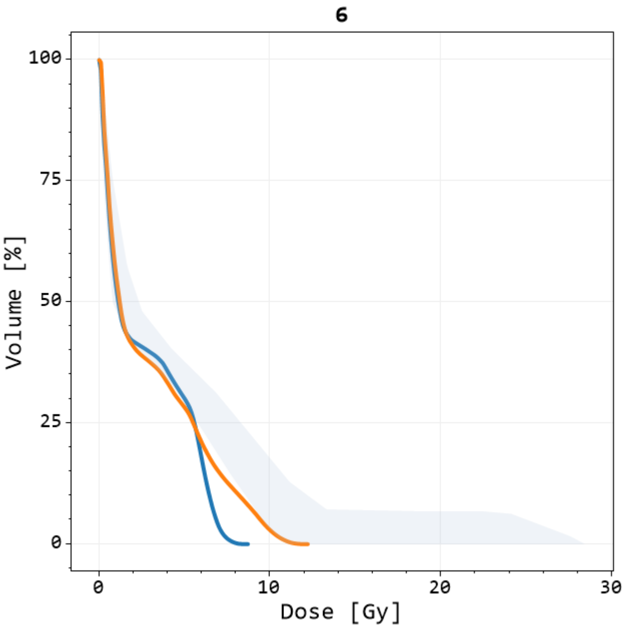
6
Volume [%]
100
75
50
25
0
0
10
20
30
Dose [Gy]

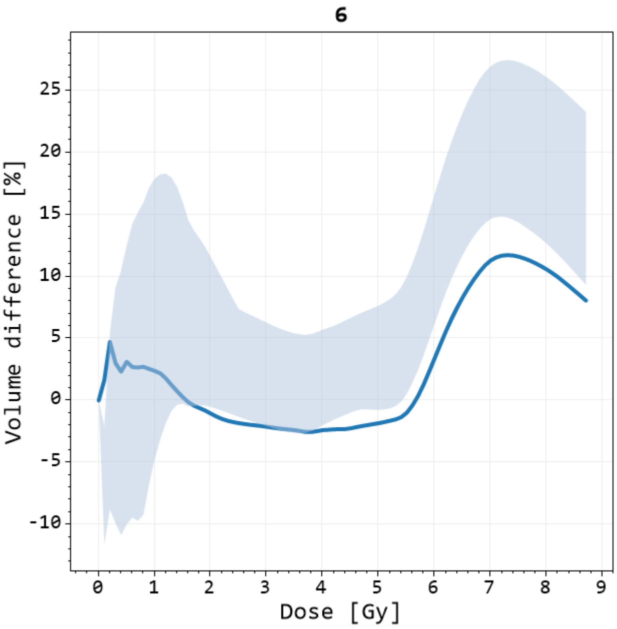
6
Volume difference [%]
25
20
15
10
5
0
-5
-10
0
1
2
3
4
5
6
7
8
9
Dose [Gy]

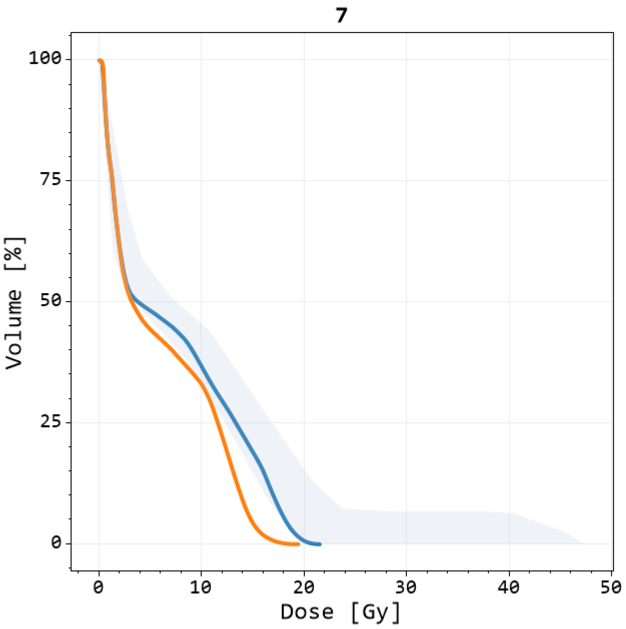
7
Volume [%]
100
75
50
25
0
0
10
20
30
40
50
Dose [Gy]

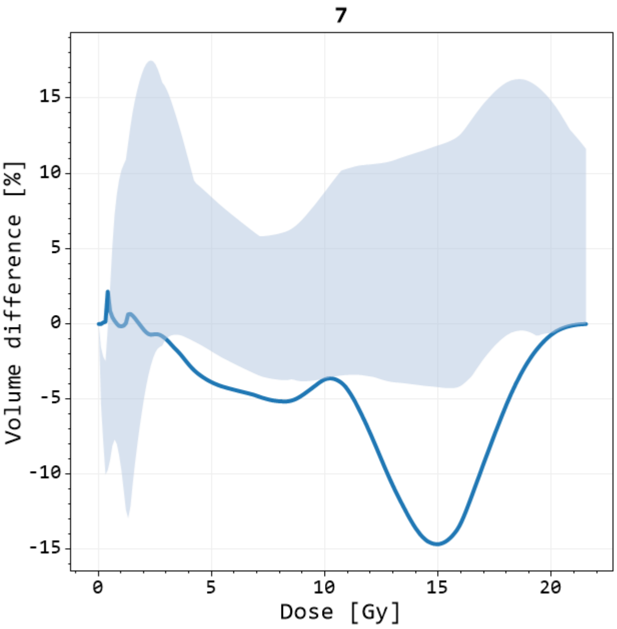
7
Volume difference [%]
15
10
5
0
-5
-10
-15
0
5
10
15
20
Dose [Gy]

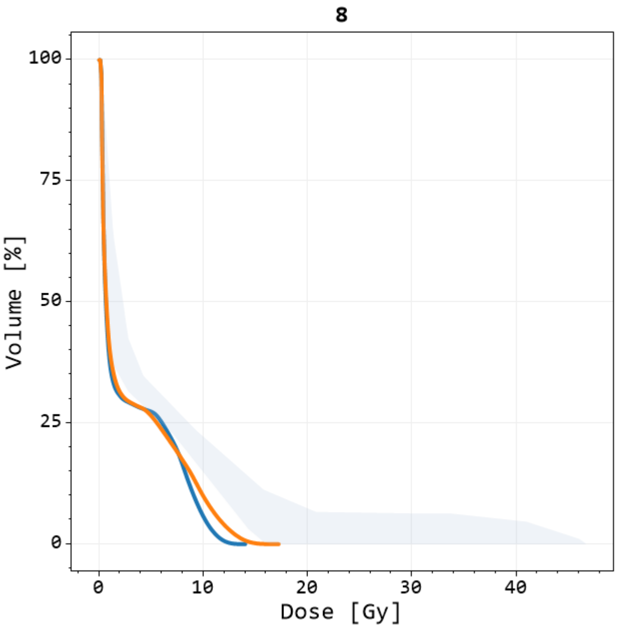
8
Volume [%]
100
75
50
25
0
0
10
20
30
40
Dose [Gy]

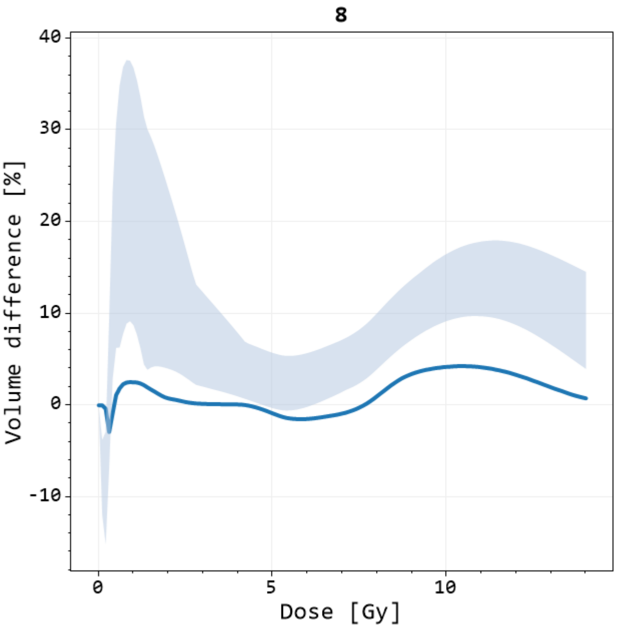
8
Volume difference [%]
40
30
20
10
0
-10
0
5
10
Dose [Gy]

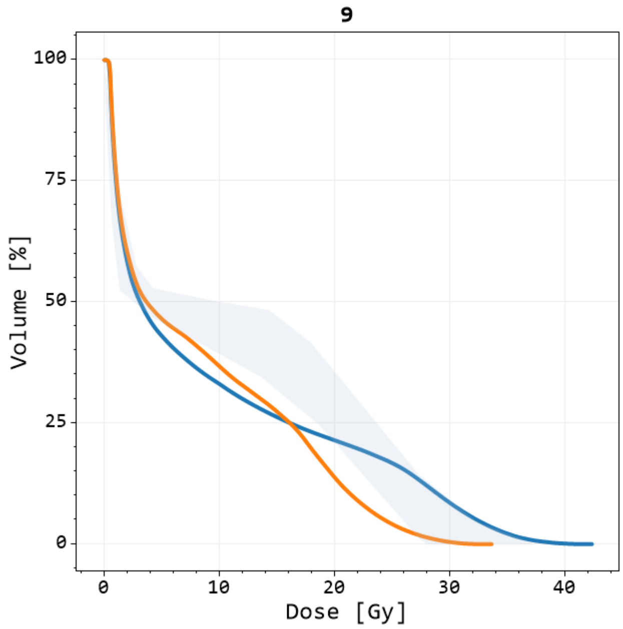
9
Volume [%]
Dose [Gy]

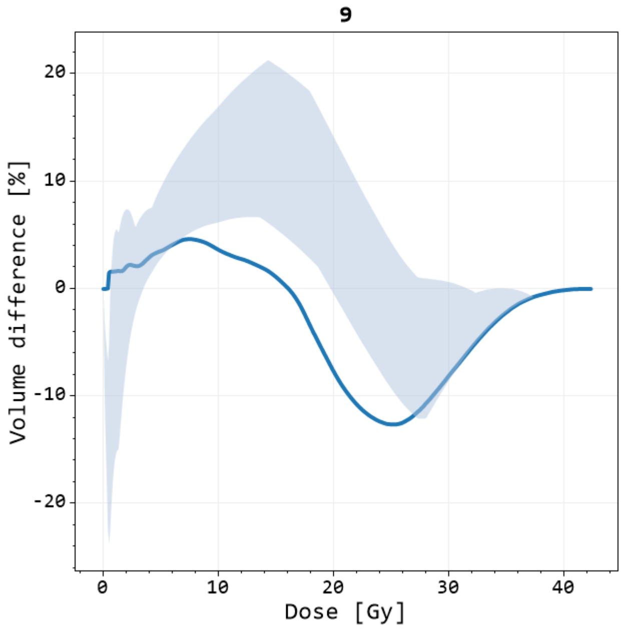
9
Volume difference [%]
Dose [Gy]

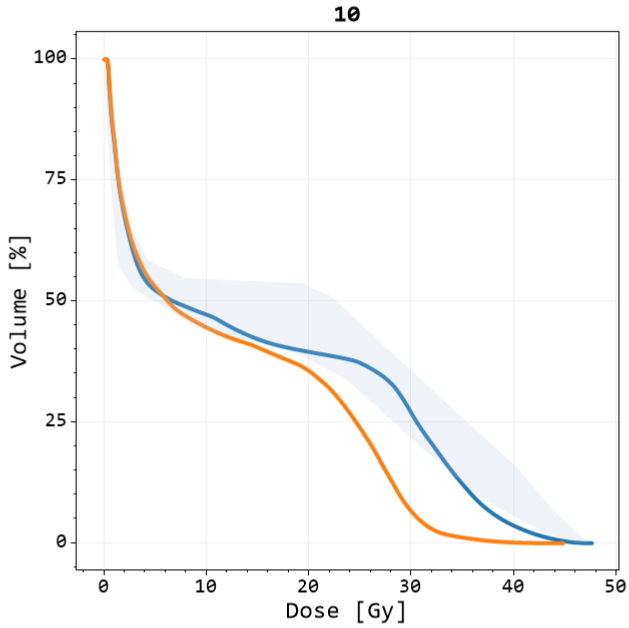
10
Volume [%]
Dose [Gy]

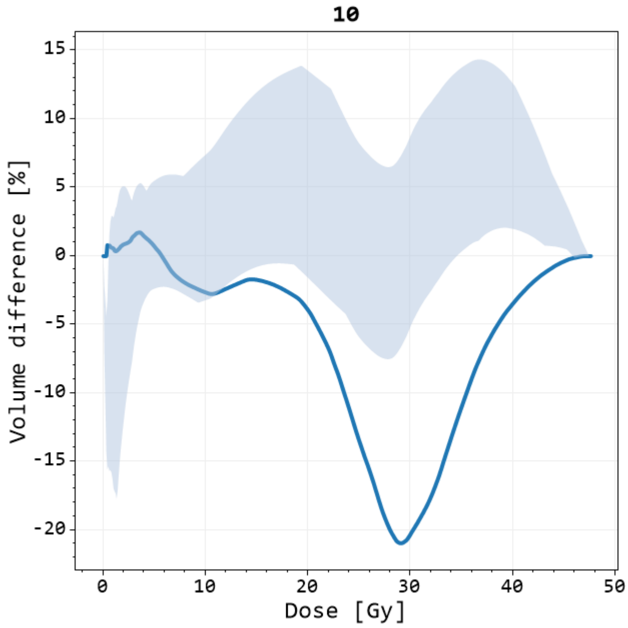
10
Volume difference [%]
Dose [Gy]

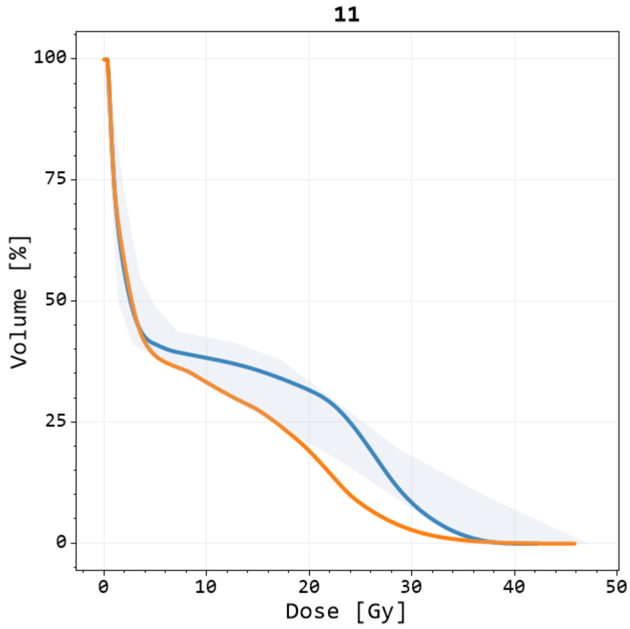
11
Volume [%]
Dose [Gy]

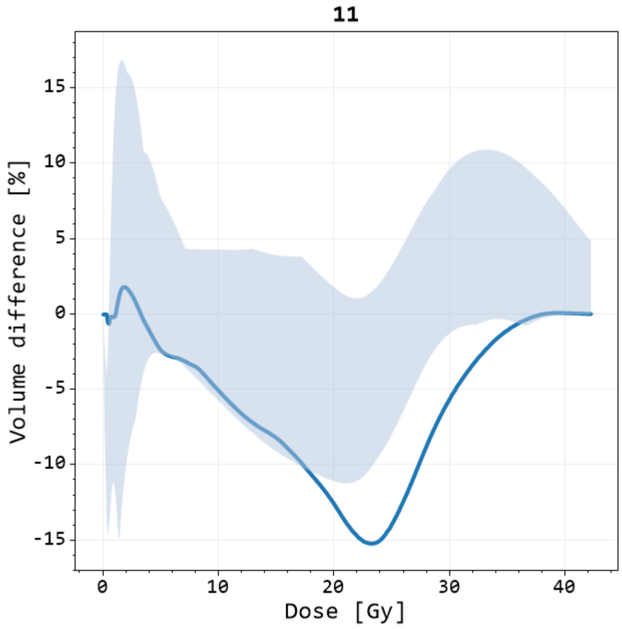
11
Volume difference [%]
Dose [Gy]

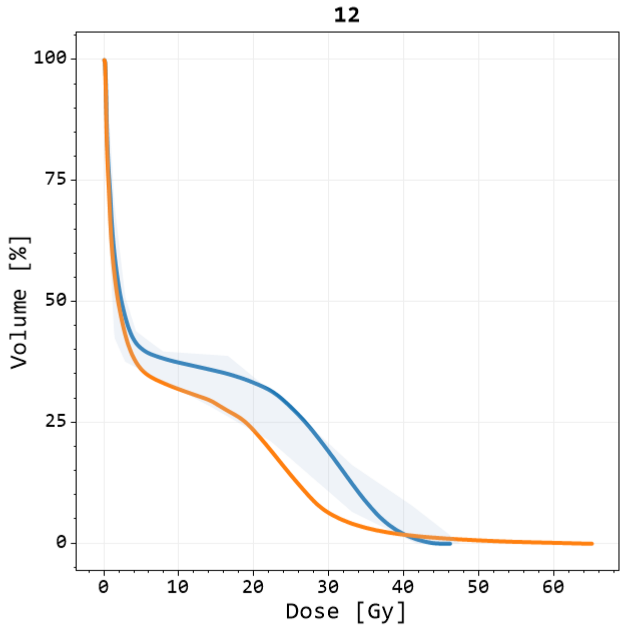
12
Volume [%]
Dose [Gy]
0
25
50
75
100
0 10 20 30 40 50 60

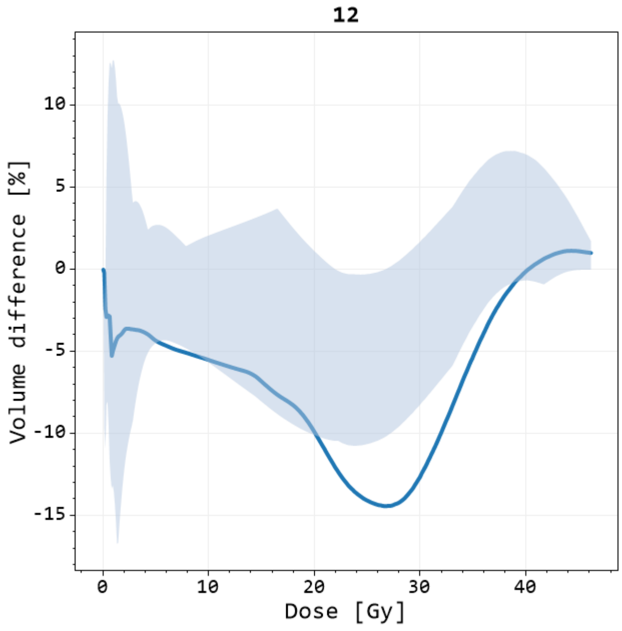
12
Volume difference [%]
Dose [Gy]
10
5
0
-5
-10
-15
0 10 20 30 40

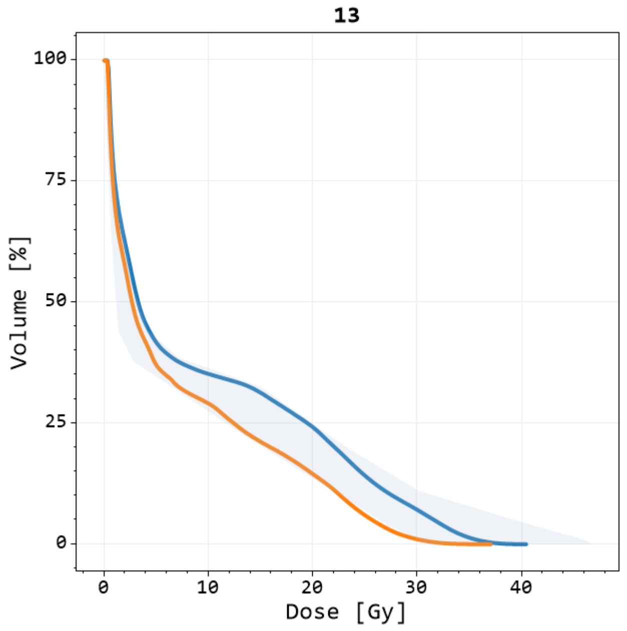
13
Volume [%]
Dose [Gy]
0
25
50
75
100
0 10 20 30 40

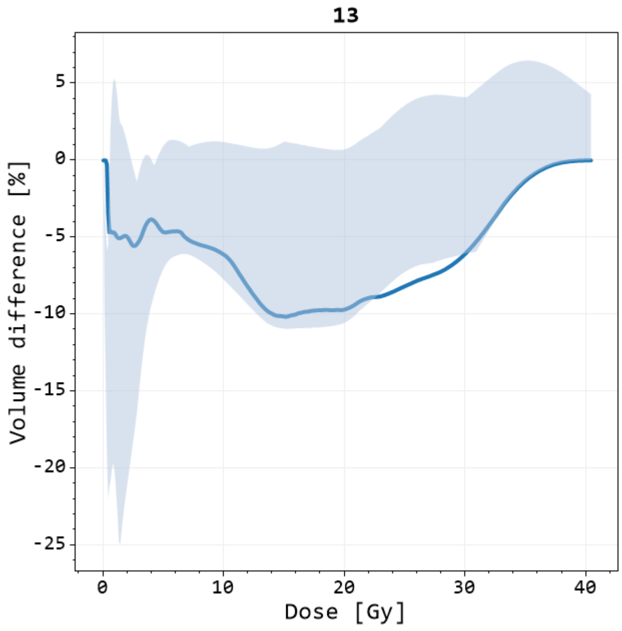
13
Volume difference [%]
Dose [Gy]
5
0
-5
-10
-15
-20
-25
0 10 20 30 40

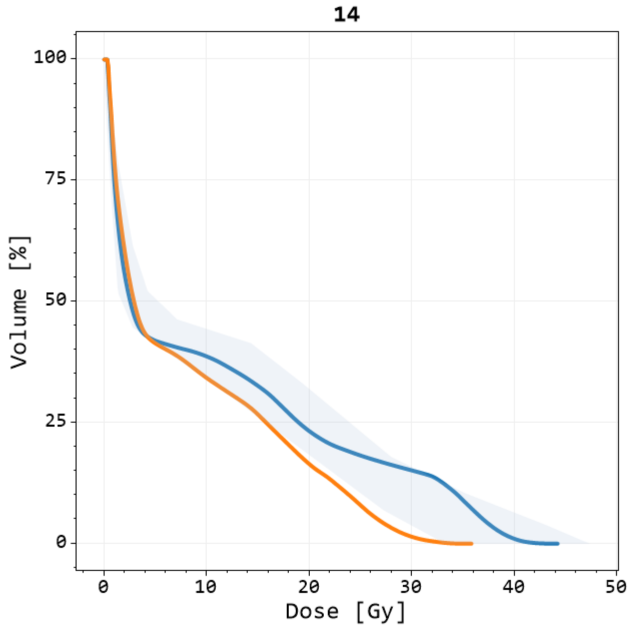
14
Volume [%]
Dose [Gy]
0
25
50
75
100
0 10 20 30 40 50

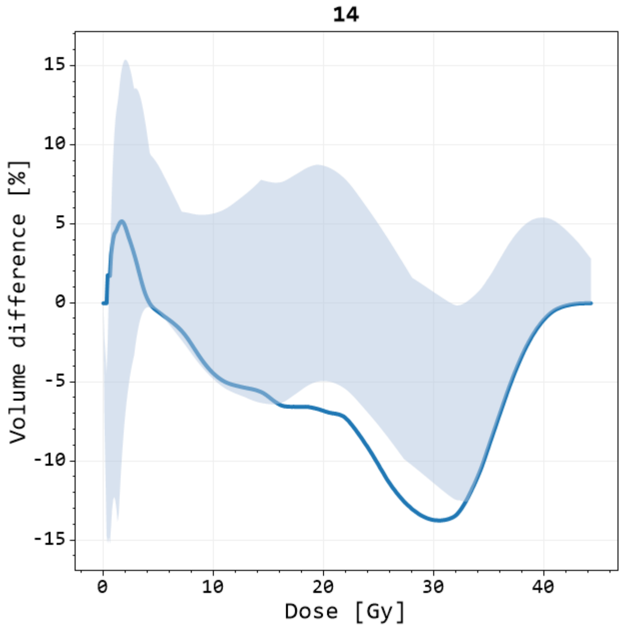
14
Volume difference [%]
Dose [Gy]
15
10
5
0
-5
-10
-15
0 10 20 30 40

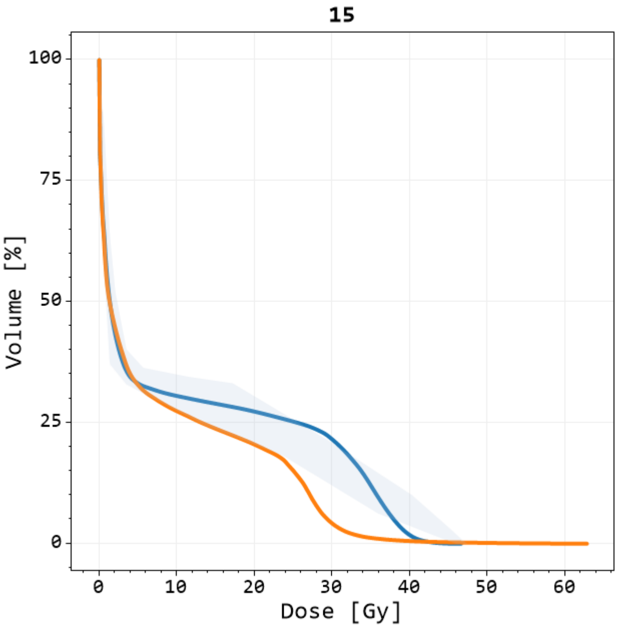
15
Volume [%]
Dose [Gy]
100
75
50
25
0
0
10
20
30
40
50
60

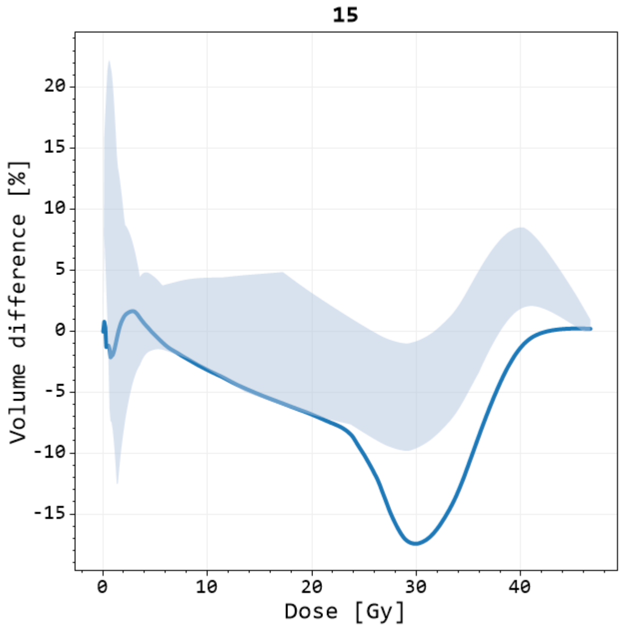
15
Volume difference [%]
Dose [Gy]
20
15
10
5
0
-5
-10
-15
0
10
20
30
40

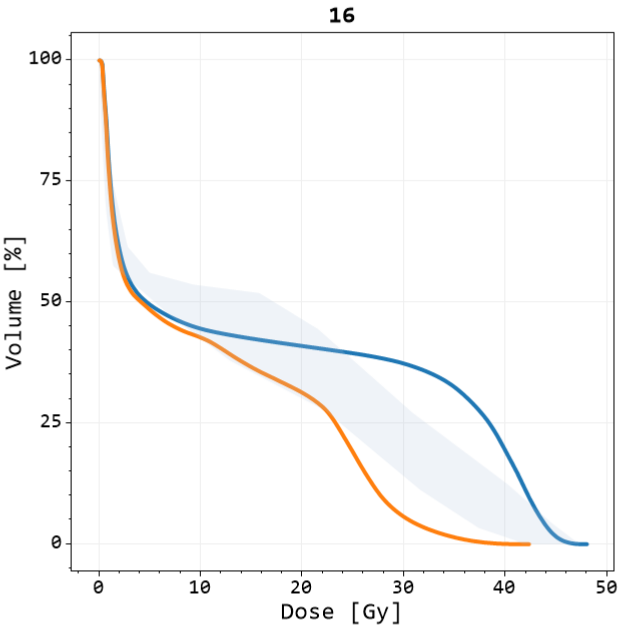
16
Volume [%]
Dose [Gy]
100
75
50
25
0
0
10
20
30
40
50

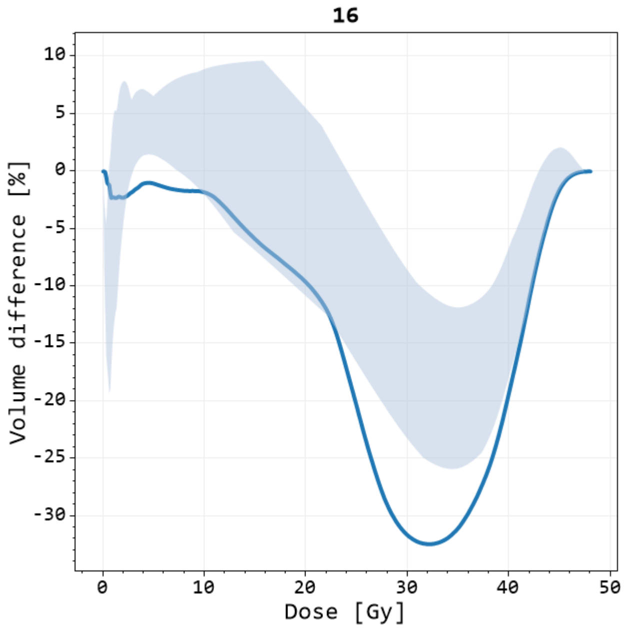
16
Volume difference [%]
Dose [Gy]
10
5
0
-5
-10
-15
-20
-25
-30
0
10
20
30
40
50

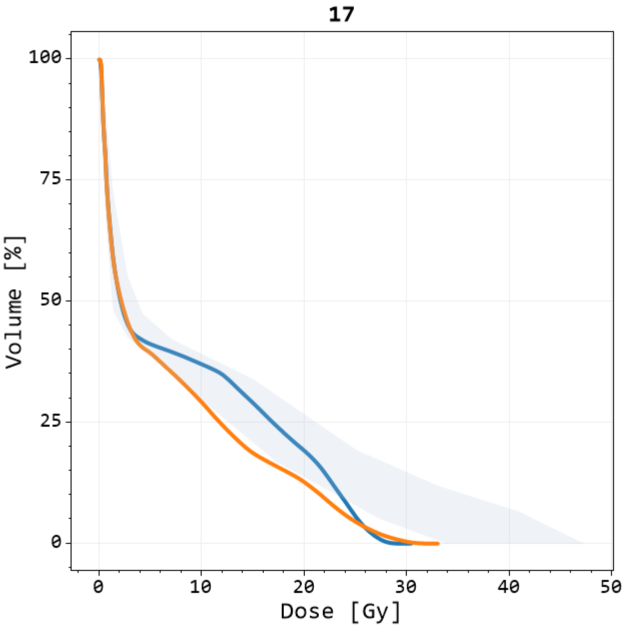
17
Volume [%]
Dose [Gy]
100
75
50
25
0
0
10
20
30
40
50

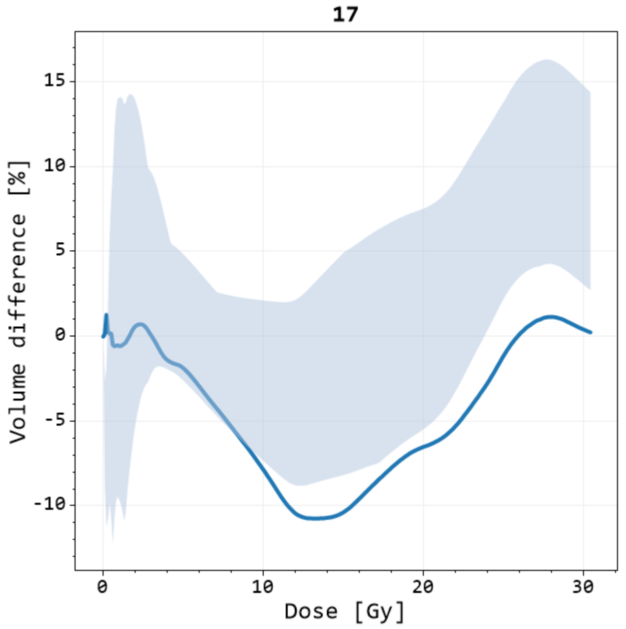
17
Volume difference [%]
Dose [Gy]
15
10
5
0
-5
-10
0
10
20
30

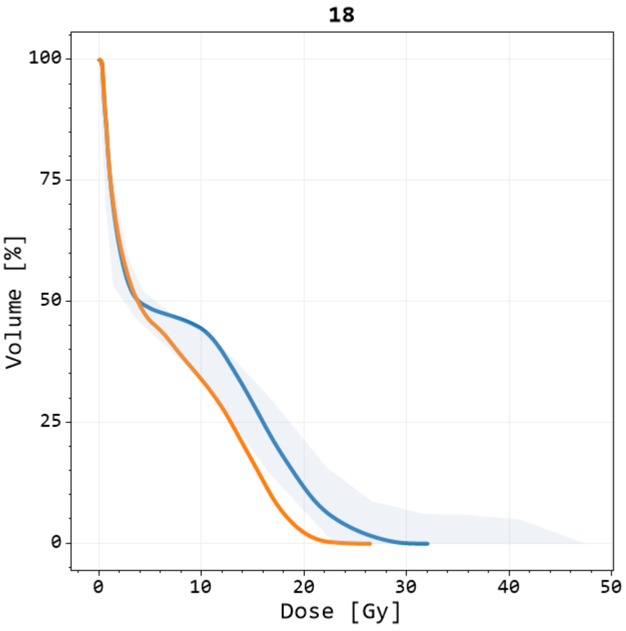
18
Volume [%]
100
75
50
25
0
0
10
20
30
40
50
Dose [Gy]

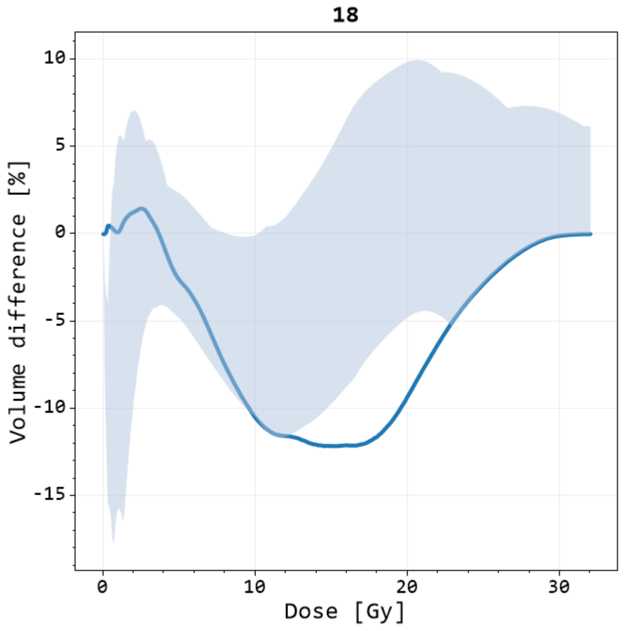
18
Volume difference [%]
10
5
0
-5
-10
-15
0
10
20
30
Dose [Gy]

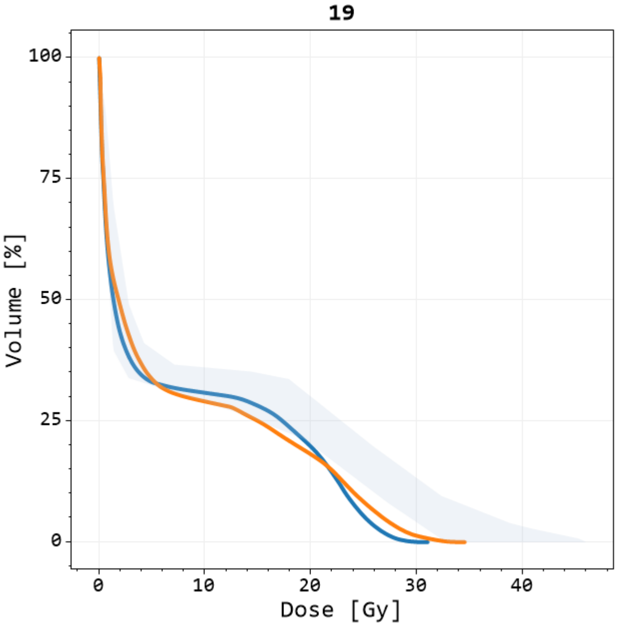
19
Volume [%]
100
75
50
25
0
0
10
20
30
40
Dose [Gy]

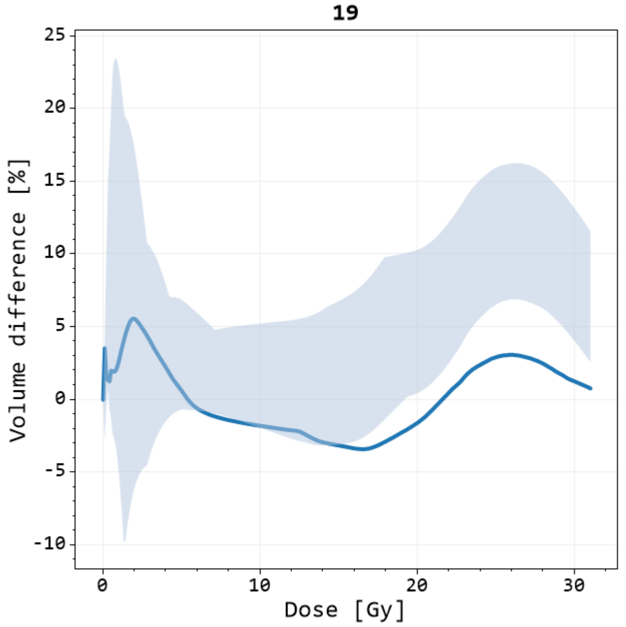
19
Volume difference [%]
25
20
15
10
5
0
-5
-10
0
10
20
30
Dose [Gy]

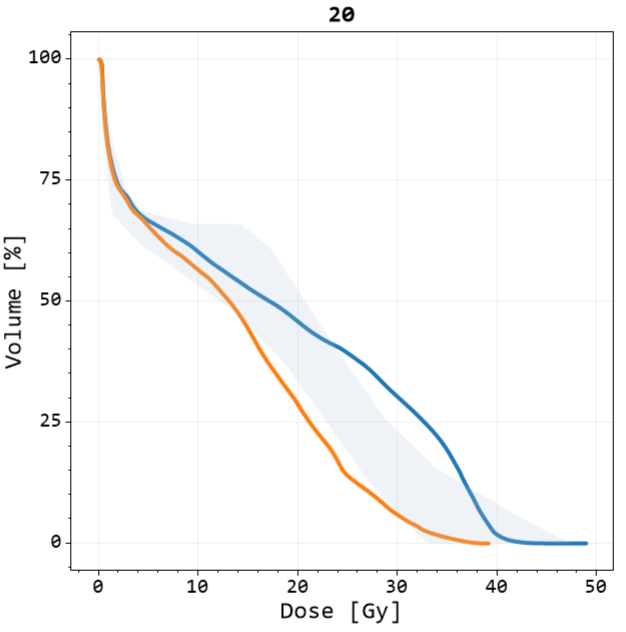
20
Volume [%]
100
75
50
25
0
0
10
20
30
40
50
Dose [Gy]

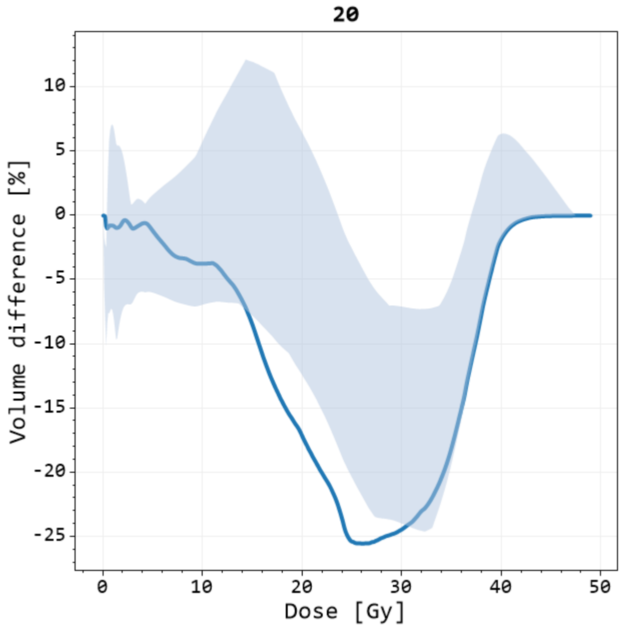
20
Volume difference [%]
10
5
0
-5
-10
-15
-20
-25
0
10
20
30
40
50
Dose [Gy]

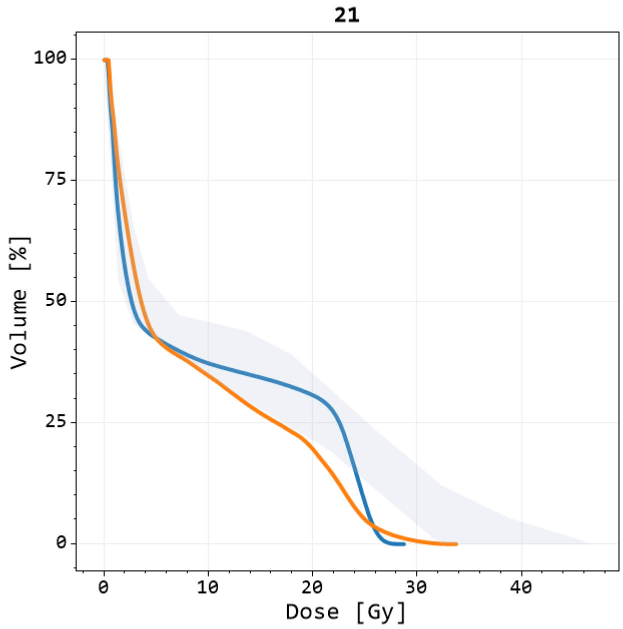
21
Volume [%]
100
75
50
25
0
0
10
20
30
40
Dose [Gy]

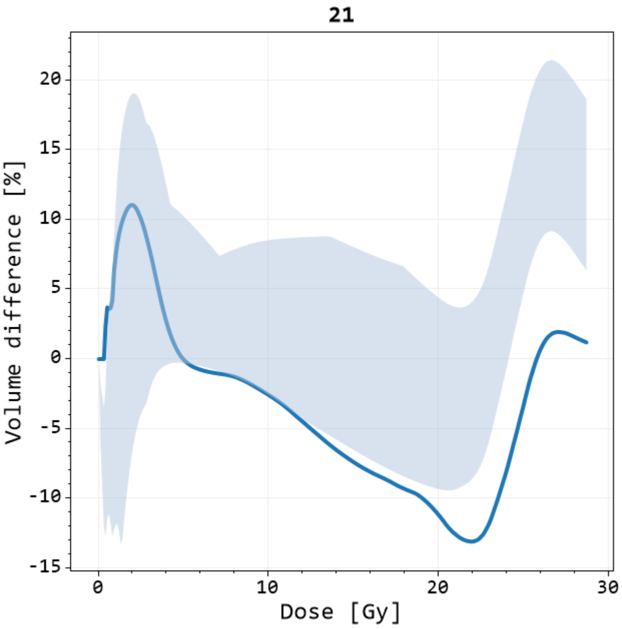
21
Volume difference [%]
20
15
10
5
0
-5
-10
-15
0
10
20
30
Dose [Gy]

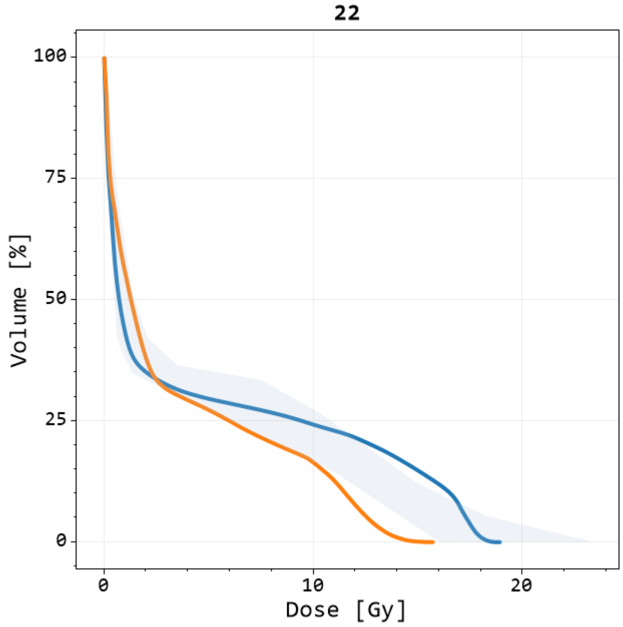
22
Volume [%]
100
75
50
25
0
0
10
20
Dose [Gy]

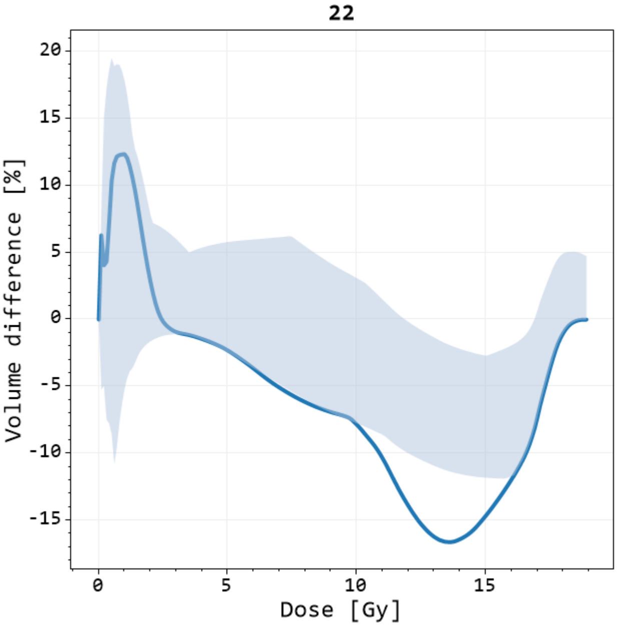
22
Volume difference [%]
20
15
10
5
0
-5
-10
-15
0
5
10
15
Dose [Gy]

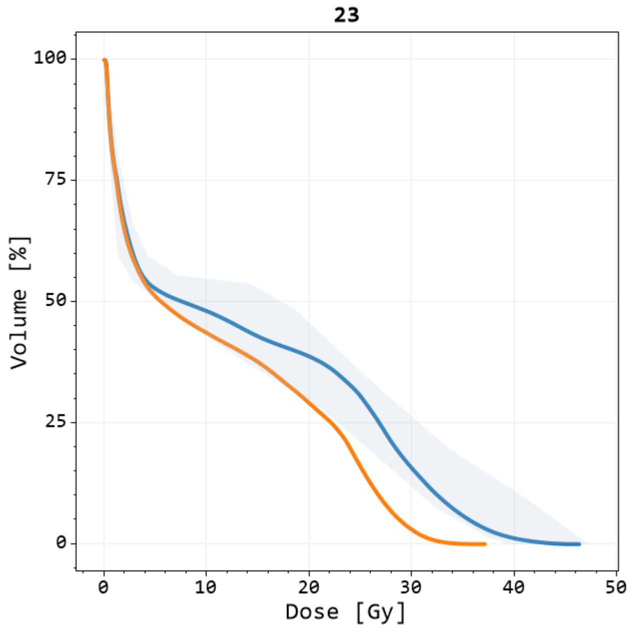
23
100
75
50
25
0
Volume [%]
0
10
20
30
40
50
Dose [Gy]

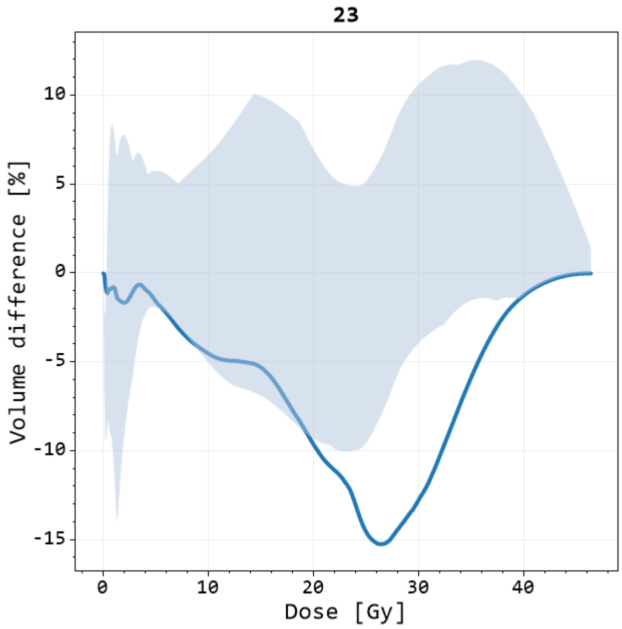
23
10
5
0
-5
-10
-15
Volume difference [%]
0
10
20
30
40
Dose [Gy]

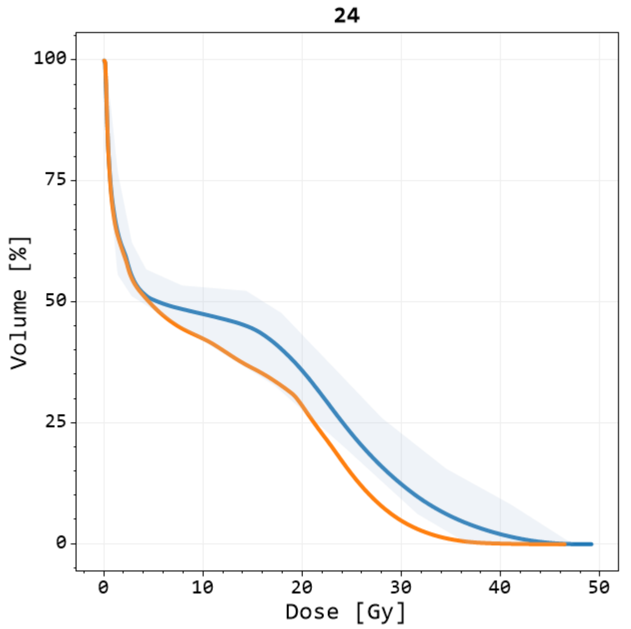
24
100
75
50
25
0
Volume [%]
0
10
20
30
40
50
Dose [Gy]

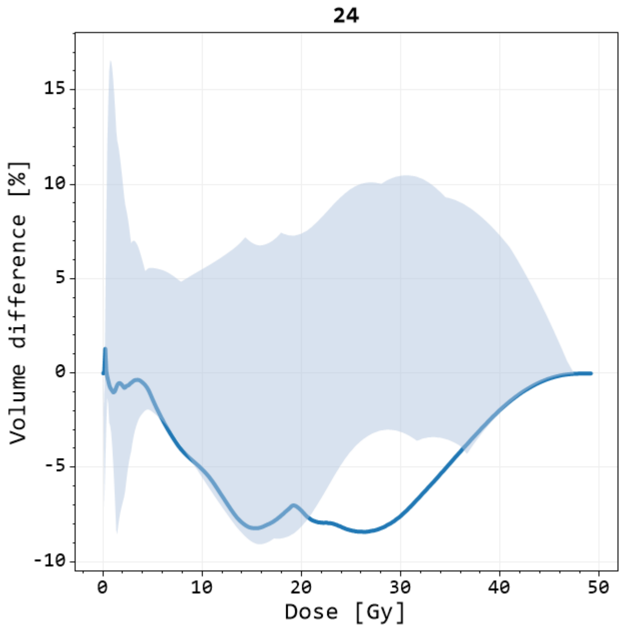
24
15
10
5
0
-5
-10
Volume difference [%]
0
10
20
30
40
50
Dose [Gy]

# PTV

## DVH Volume difference

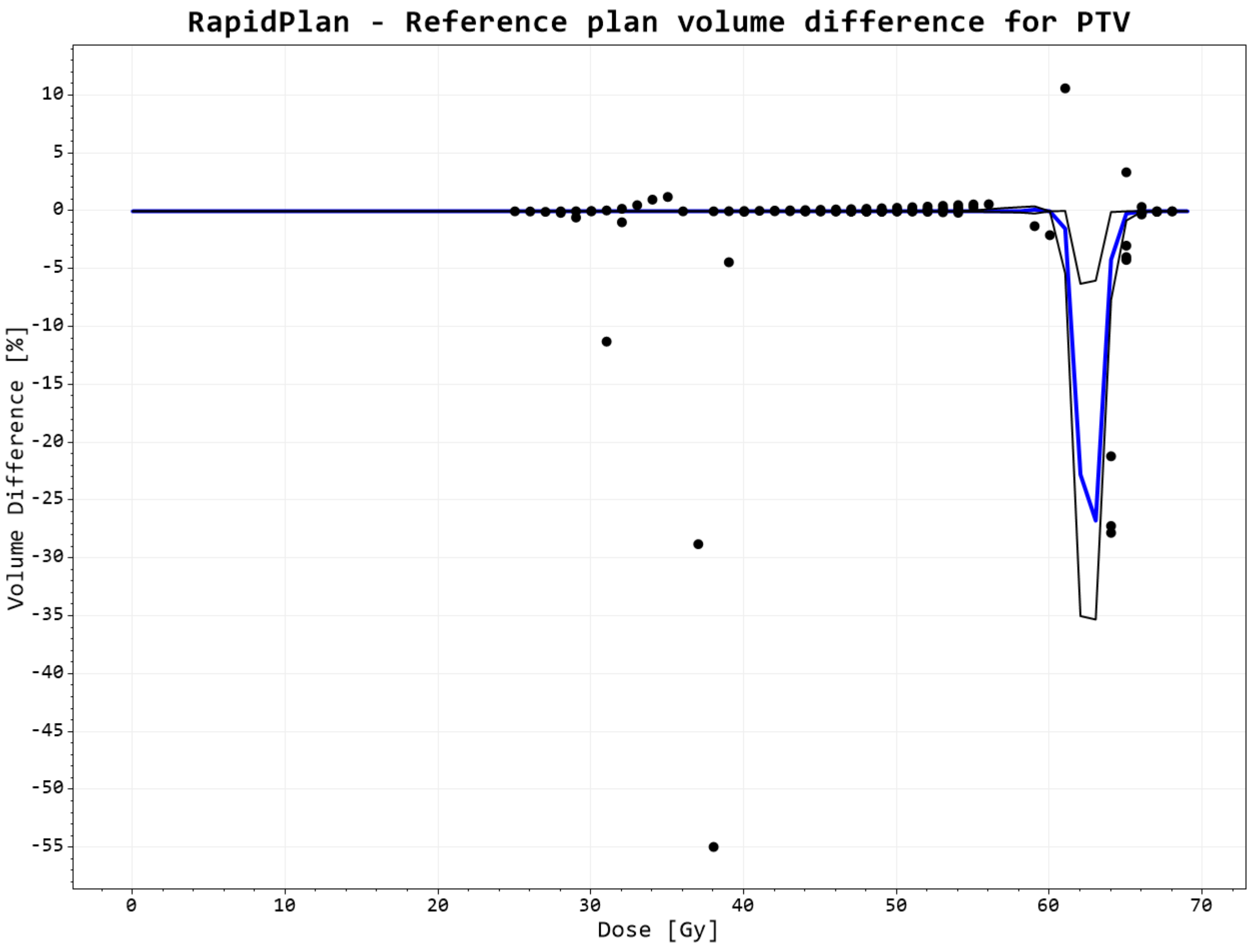


## Dose-volume metric summary table

| Metric | Reference Plan | RapidPlan | Difference |
|---|---|---|---|
| DMax[Percent] | 111.0 [109.9,112.2] | 109.7 [109.0,110.5] | -1.2 [-2.3,0.3] (p = 0.993) (0) |
| V102Percent[%] | 84.9 [80.5,87.8] | 81.0 [76.1,83.8] | -3.2 [-9.5,-0.1] (p = 1.000) (1) |
| D2%[Percent] | 107.4 [106.3,108.0] | 105.4 [104.8,105.9] | -2.0 [-2.5,-1.1] (p = 1.000) (0) |
| V105Percent[%] | 33.5 [14.5,52.3] | 4.2 [1.2,7.8] | -26.9 [-38.1,-12.2] (p = 1.000) (0) |
| DMin[Percent] | 83.8 [80.3,88.2] | 83.0 [81.1,86.2] | -0.8 [-5.3,3.8] (p = 0.655) (2) |
| V101Percent[%] | 91.5 [90.5,92.2] | 90.9 [90.2,91.3] | -0.4 [-1.7,0.4] (p = 0.978) (2) |
| D98%[Percent] | 98.2 [98.1,98.7] | 98.4 [98.3,98.6] | 0.1 [-0.2,0.4] (p = 0.212) (1) |
| V98Percent[%] | 98.2 [98.1,98.7] | 98.5 [98.4,98.7] | 0.2 [-0.3,0.5] (p = 0.212) (0) |

## Dose-volume metric box whisker plots

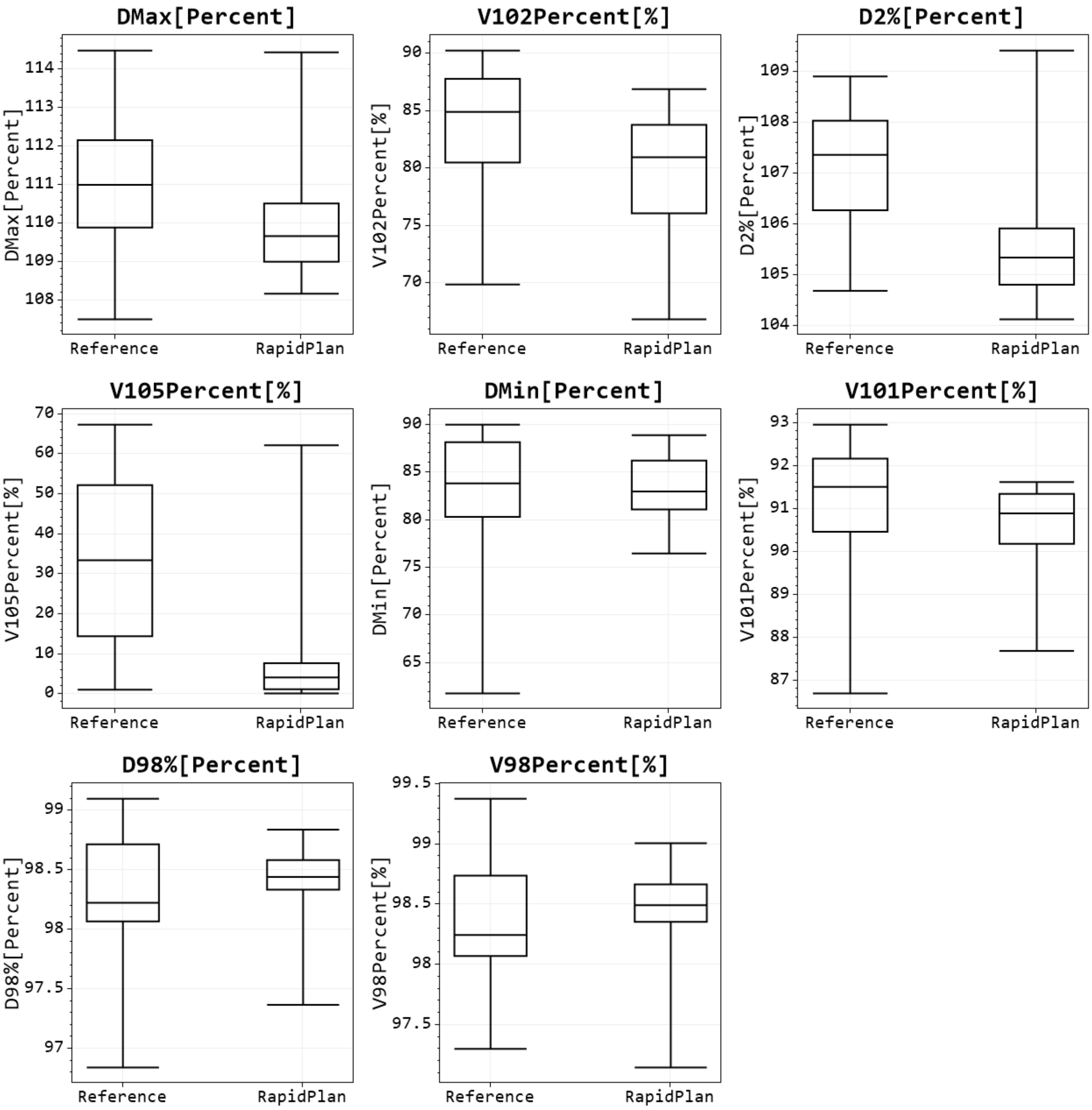

## Dose-volume metric differences by plan

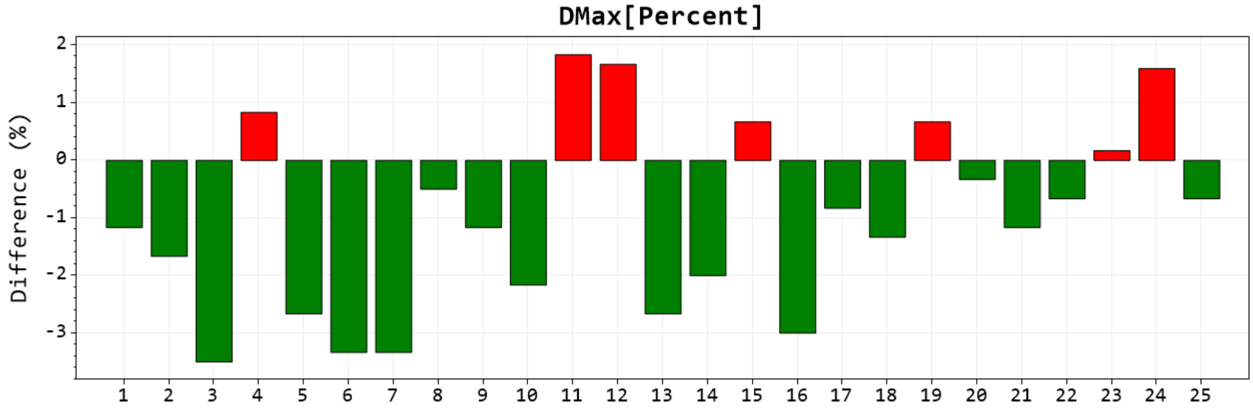


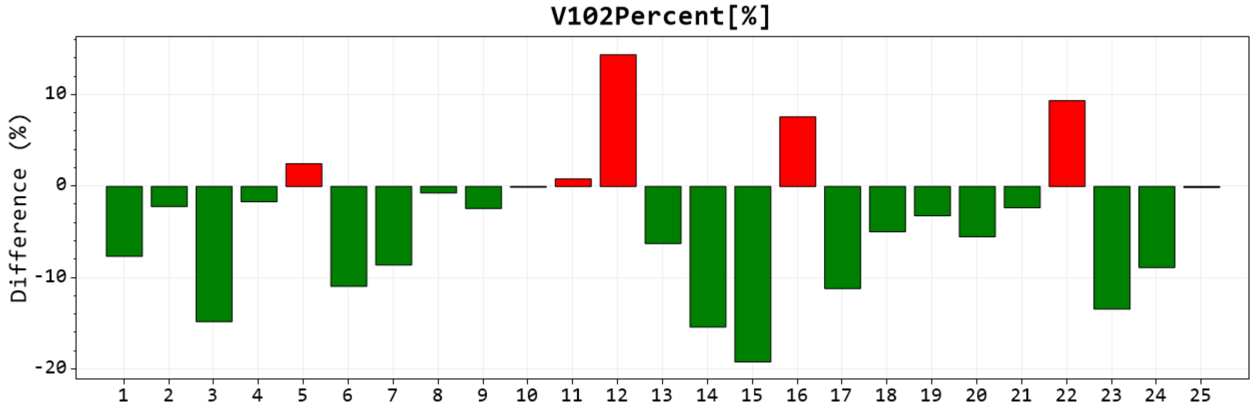


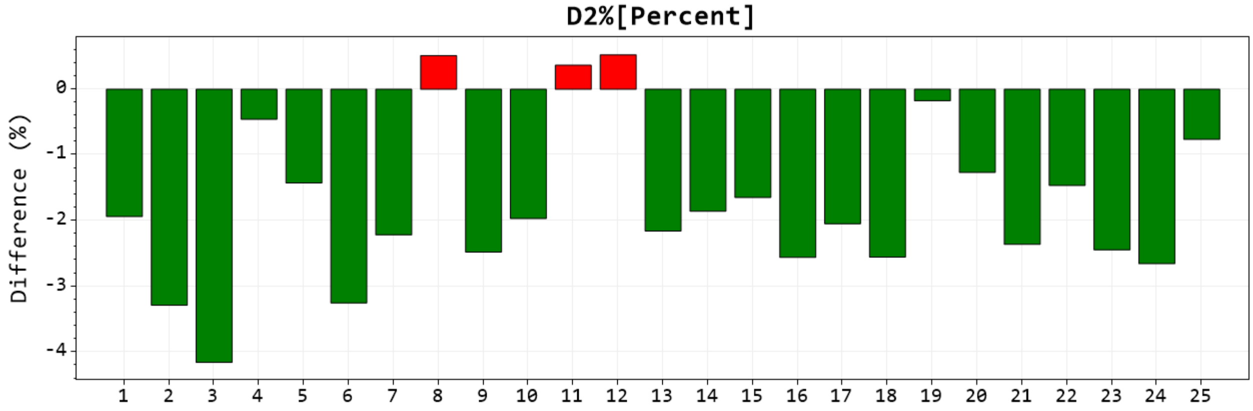


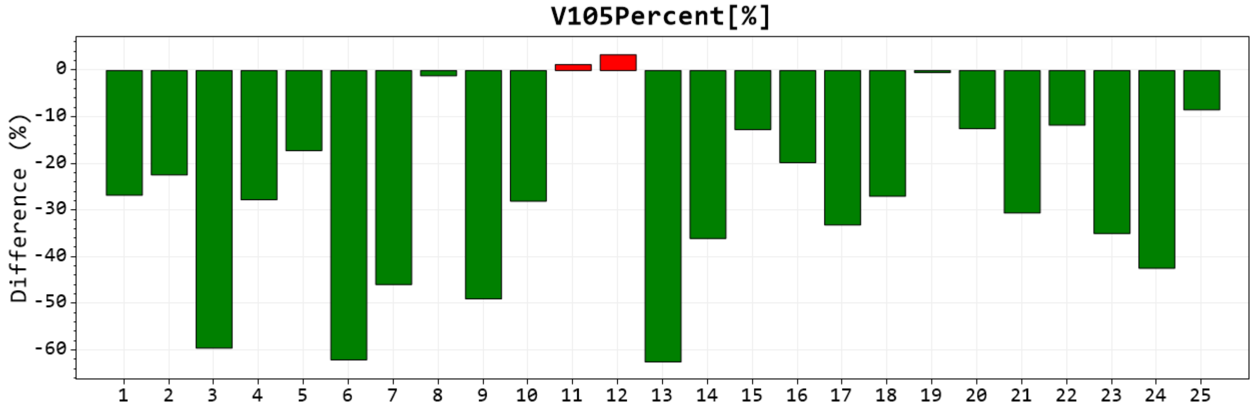

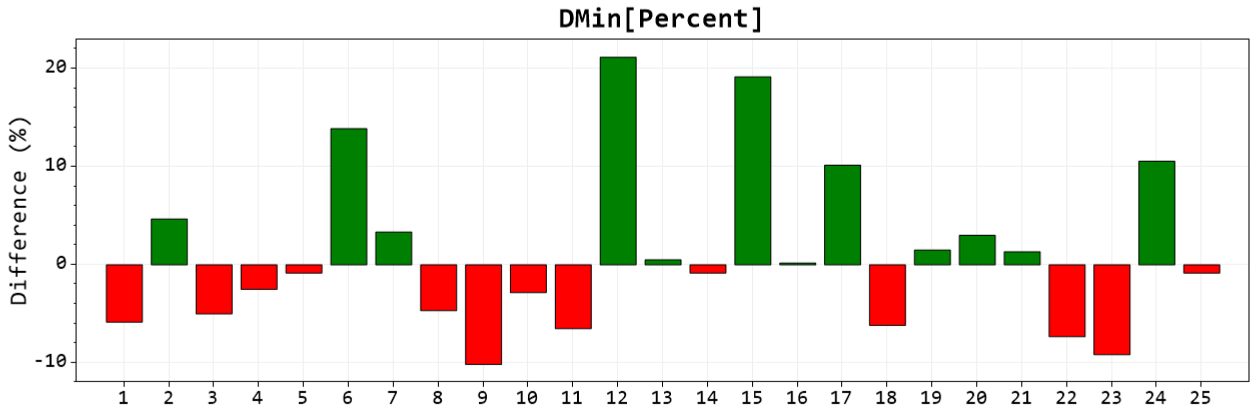
DMin[Percent]
Difference (%)
20
10
0
-10
1 2 3 4 5 6 7 8 9 10 11 12 13 14 15 16 17 18 19 20 21 22 23 24 25

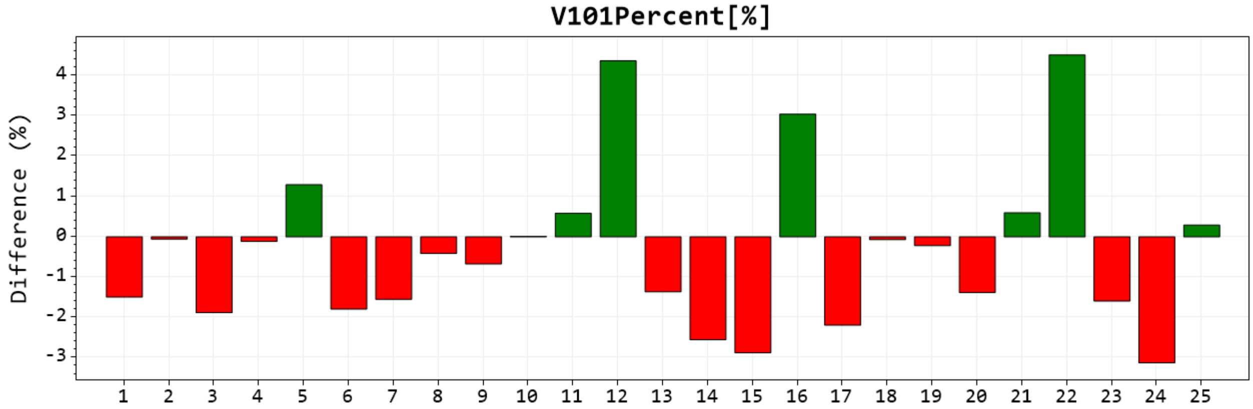
V101Percent[%]
Difference (%)
4
3
2
1
0
-1
-2
-3
1 2 3 4 5 6 7 8 9 10 11 12 13 14 15 16 17 18 19 20 21 22 23 24 25

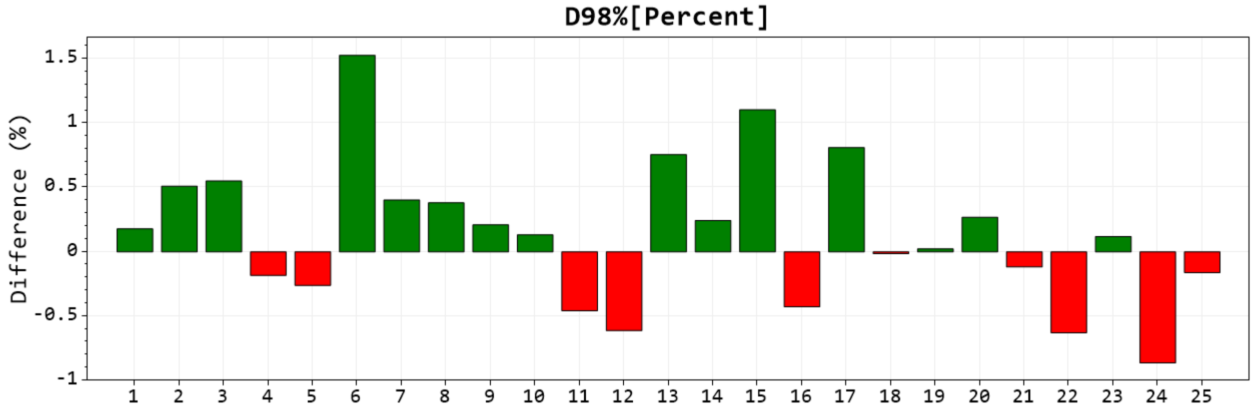
D98%[Percent]
Difference (%)
1.5
1
0.5
0
-0.5
-1
1 2 3 4 5 6 7 8 9 10 11 12 13 14 15 16 17 18 19 20 21 22 23 24 25

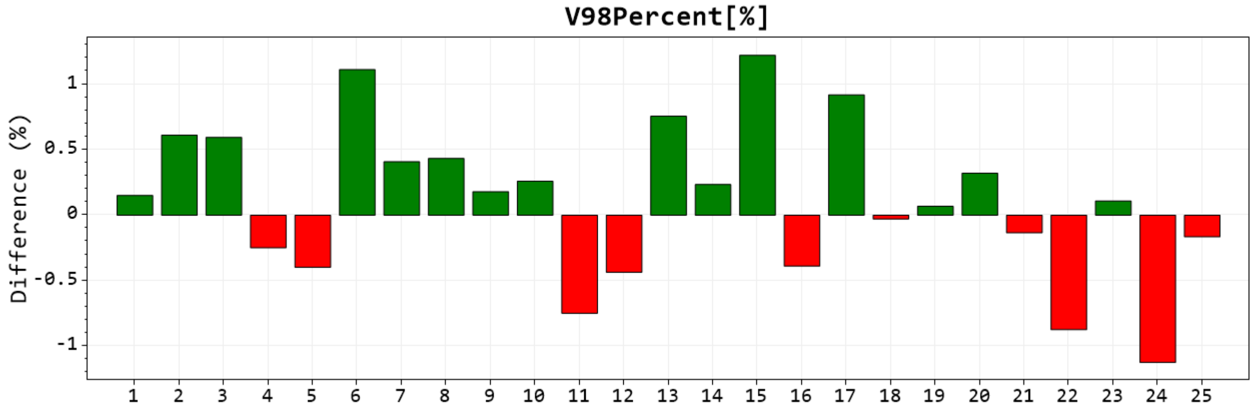
V98Percent[%]
Difference (%)
1
0.5
0
-0.5
-1
1 2 3 4 5 6 7 8 9 10 11 12 13 14 15 16 17 18 19 20 21 22 23 24 25

## Dose-volume histograms

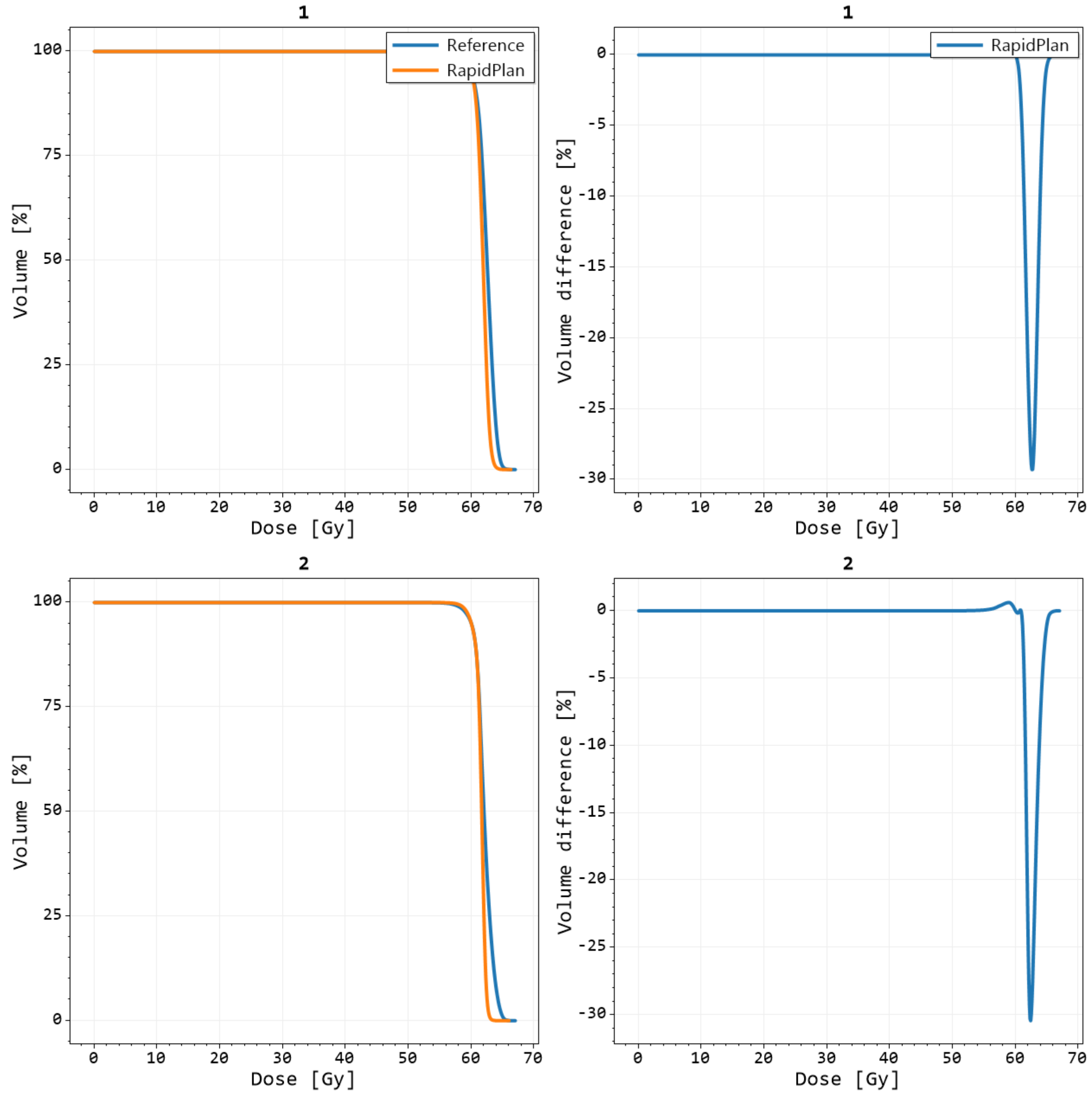

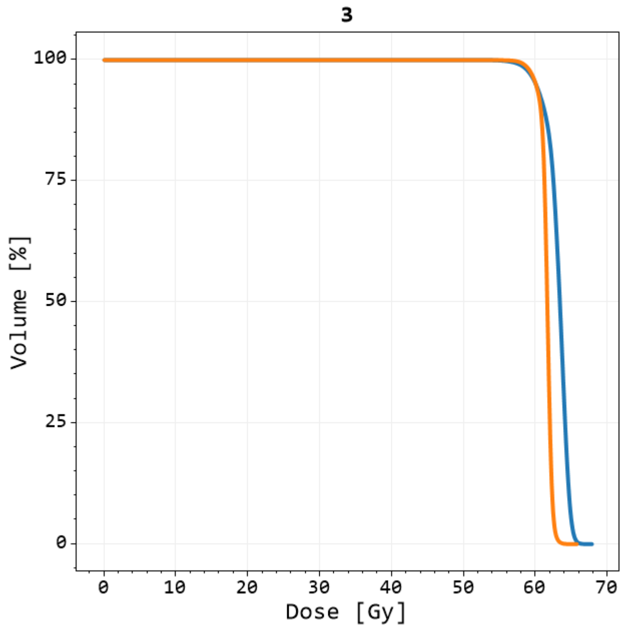
3
100
75
50
25
0
Volume [%]
0 10 20 30 40 50 60 70
Dose [Gy]

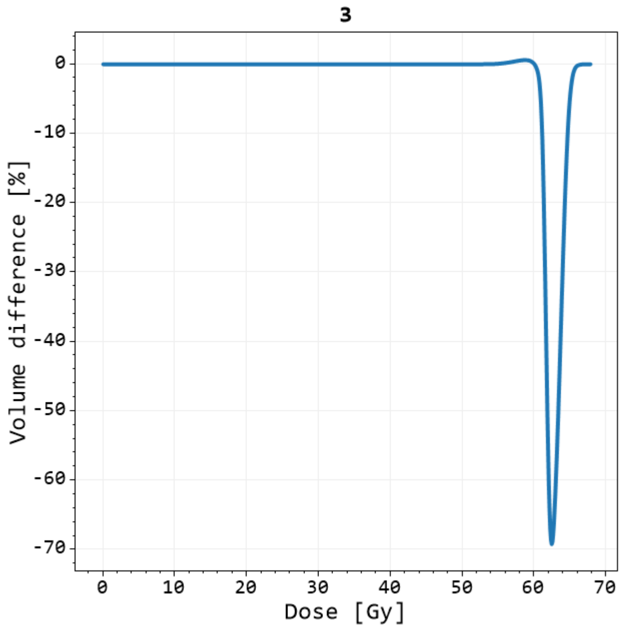
3
0
-10
-20
-30
-40
-50
-60
-70
Volume difference [%]
0 10 20 30 40 50 60 70
Dose [Gy]

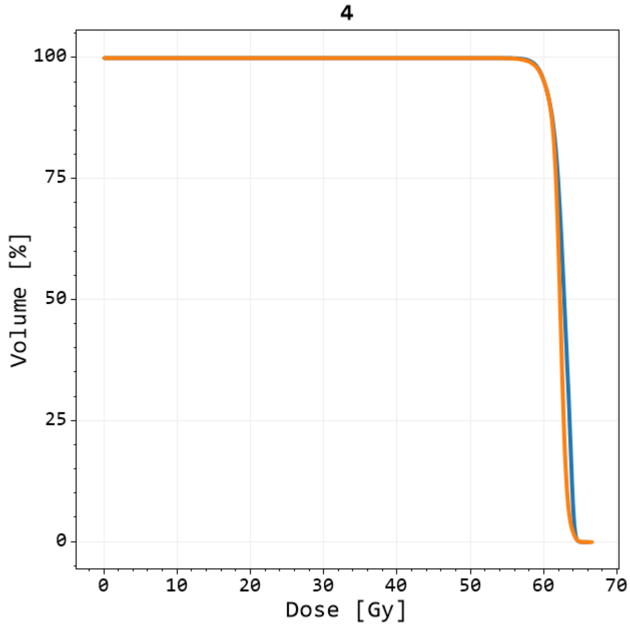
4
100
75
50
25
0
Volume [%]
0 10 20 30 40 50 60 70
Dose [Gy]

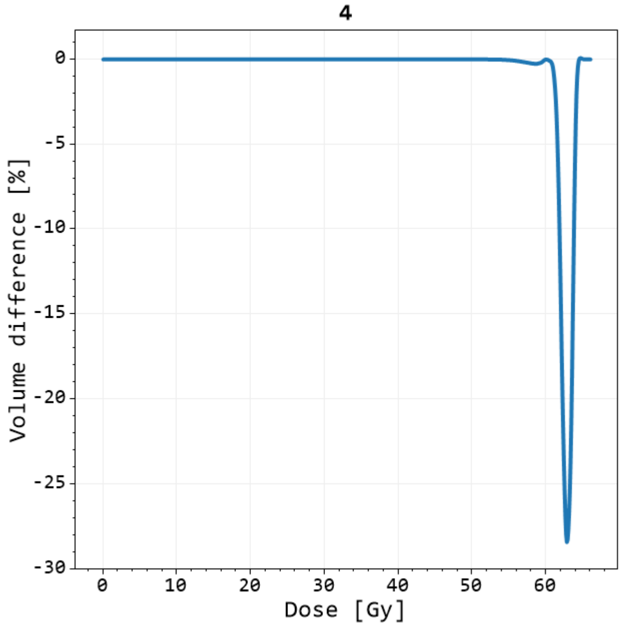
4
0
-5
-10
-15
-20
-25
-30
Volume difference [%]
0 10 20 30 40 50 60
Dose [Gy]

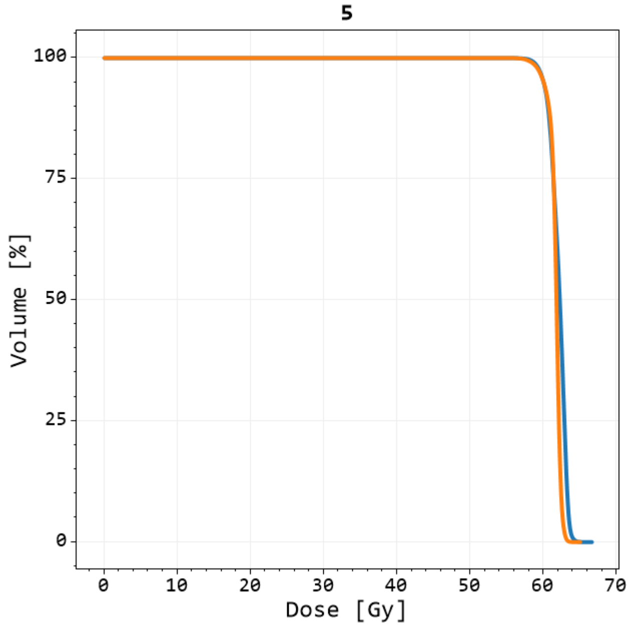
5
100
75
50
25
0
Volume [%]
0 10 20 30 40 50 60 70
Dose [Gy]

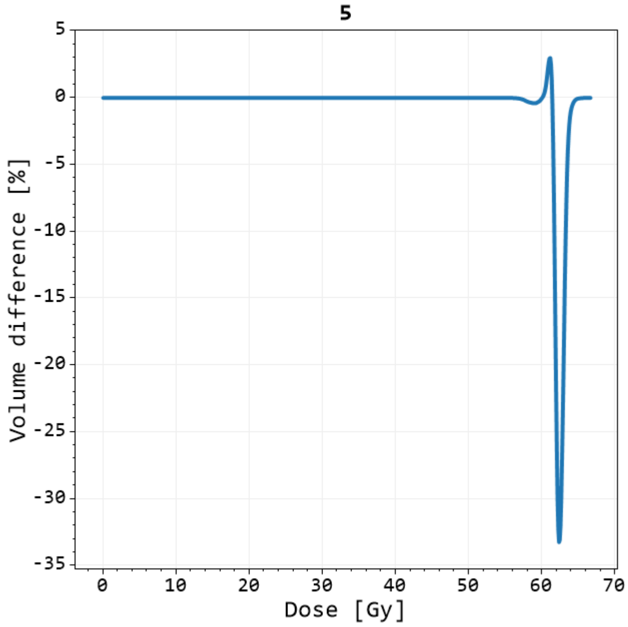
5
5
0
-5
-10
-15
-20
-25
-30
-35
Volume difference [%]
0 10 20 30 40 50 60 70
Dose [Gy]

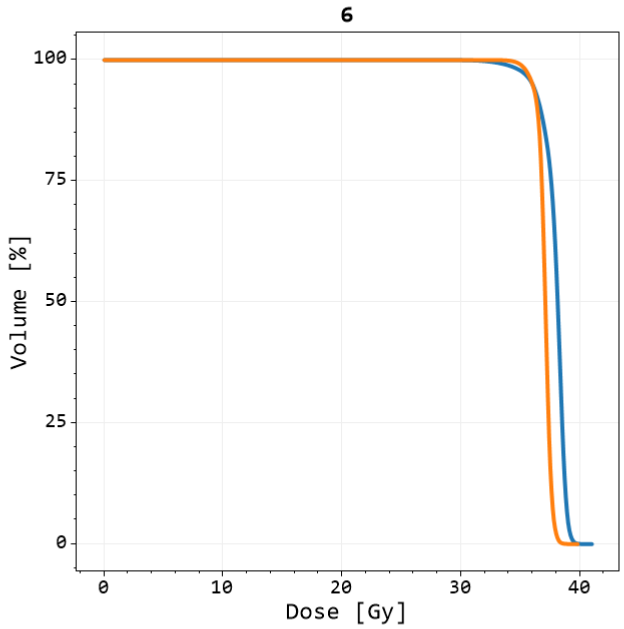
6
Volume [%]
Dose [Gy]
100
75
50
25
0
0
10
20
30
40

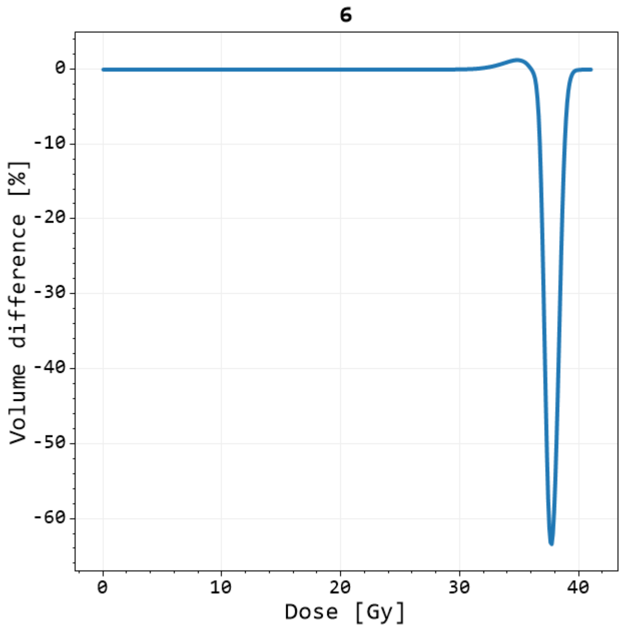
6
Volume difference [%]
Dose [Gy]
0
-10
-20
-30
-40
-50
-60
0
10
20
30
40

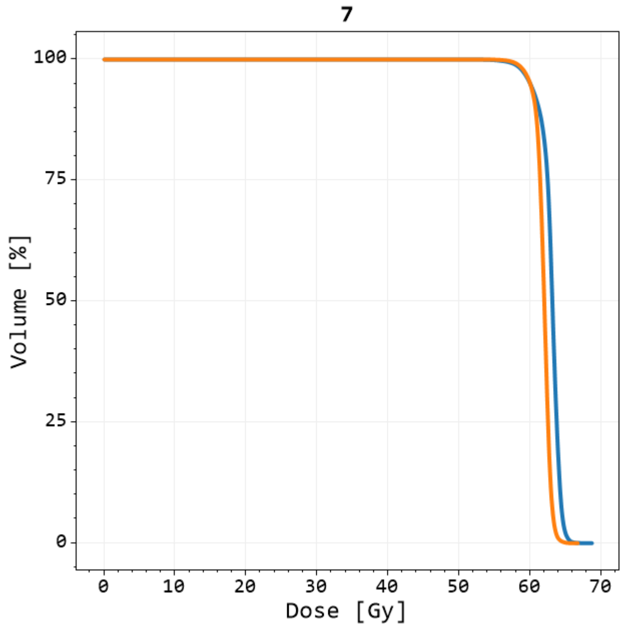
7
Volume [%]
Dose [Gy]
100
75
50
25
0
0
10
20
30
40
50
60
70

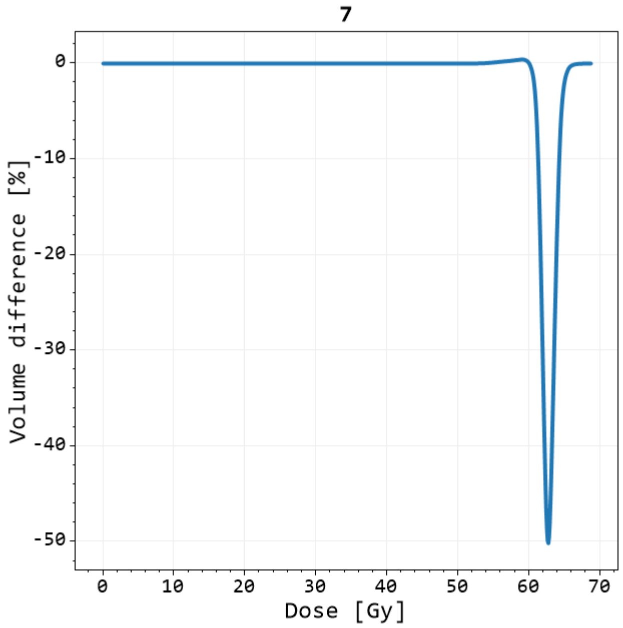
7
Volume difference [%]
Dose [Gy]
0
-10
-20
-30
-40
-50
0
10
20
30
40
50
60
70

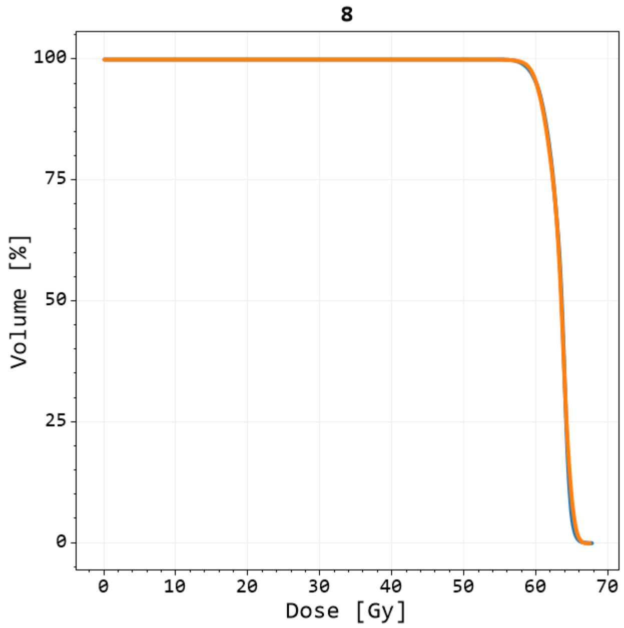
8
Volume [%]
Dose [Gy]
100
75
50
25
0
0
10
20
30
40
50
60
70

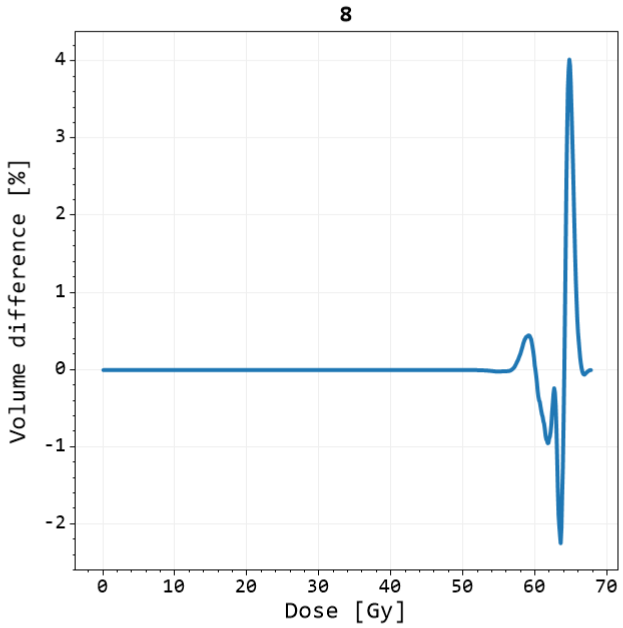
8
Volume difference [%]
Dose [Gy]
4
3
2
1
0
-1
-2
0
10
20
30
40
50
60
70

9

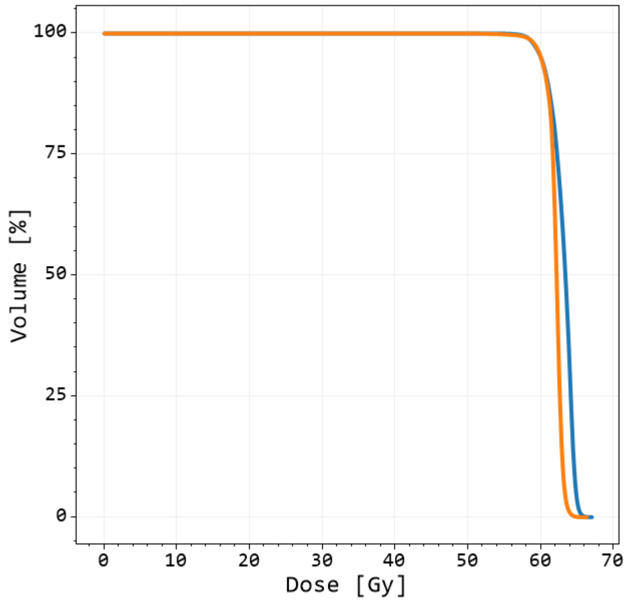


9

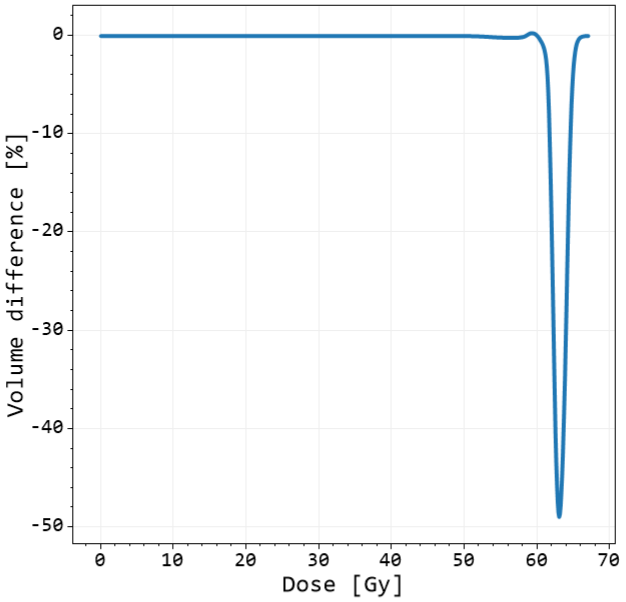


10

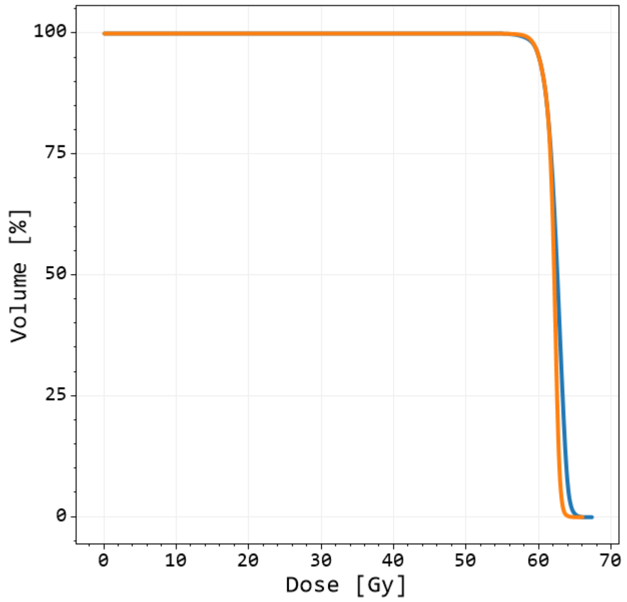


10

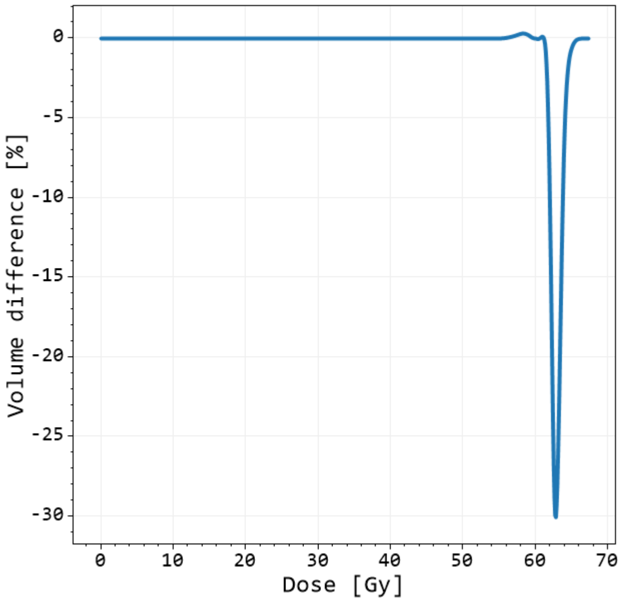


11

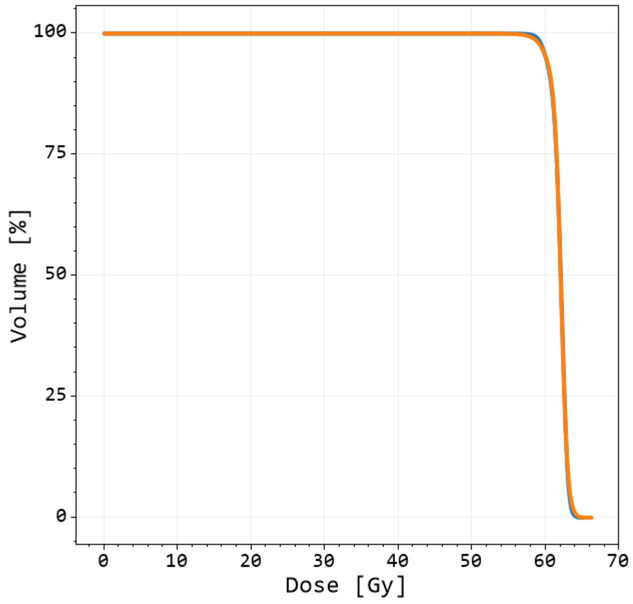


11

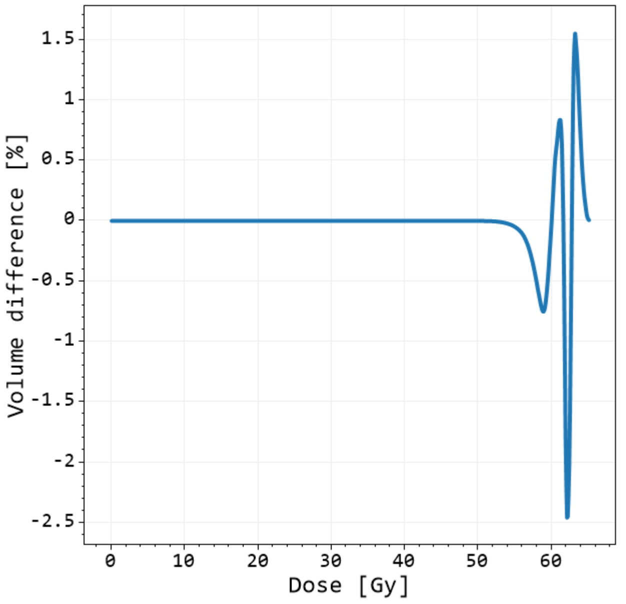


Image not found.

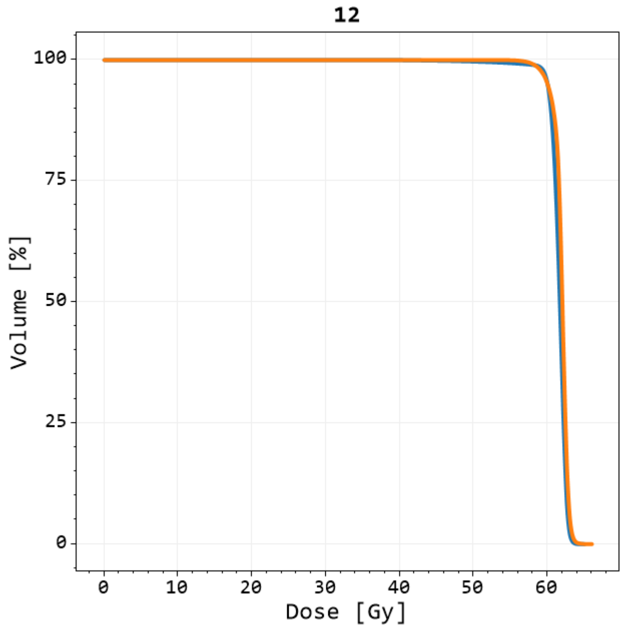
12
100
75
50
25
0
Volume [%]
0 10 20 30 40 50 60
Dose [Gy]

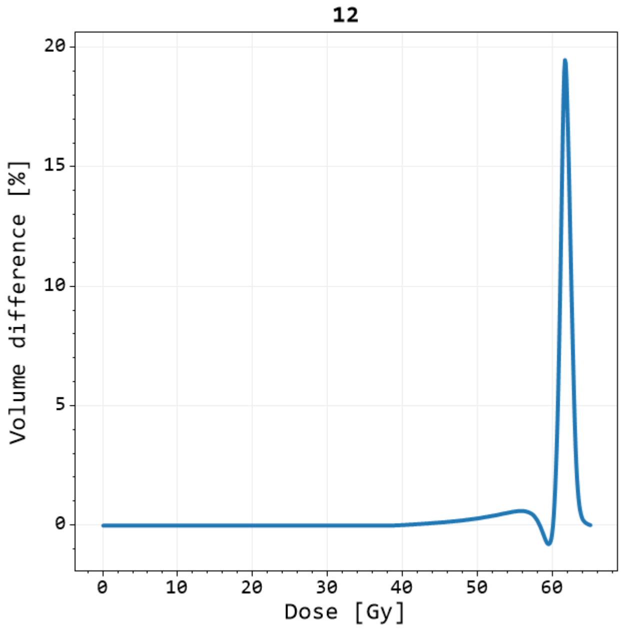
12
20
15
10
5
0
Volume difference [%]
0 10 20 30 40 50 60
Dose [Gy]

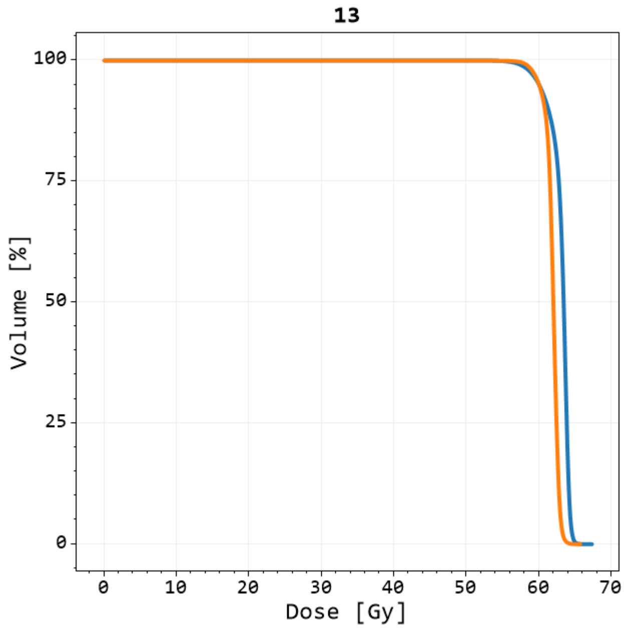
13
100
75
50
25
0
Volume [%]
0 10 20 30 40 50 60 70
Dose [Gy]

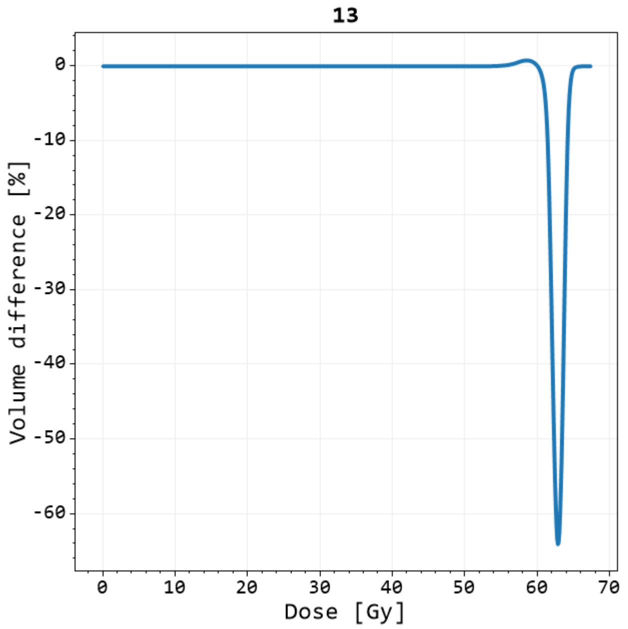
13
0
-10
-20
-30
-40
-50
-60
Volume difference [%]
0 10 20 30 40 50 60 70
Dose [Gy]

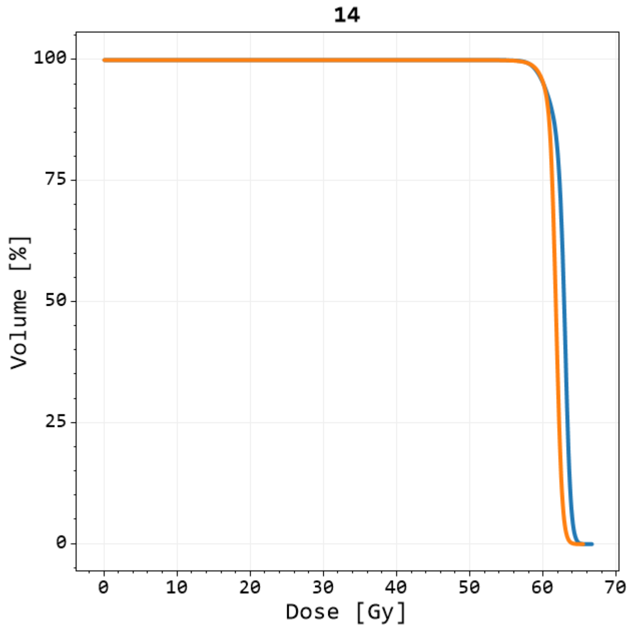
14
100
75
50
25
0
Volume [%]
0 10 20 30 40 50 60 70
Dose [Gy]

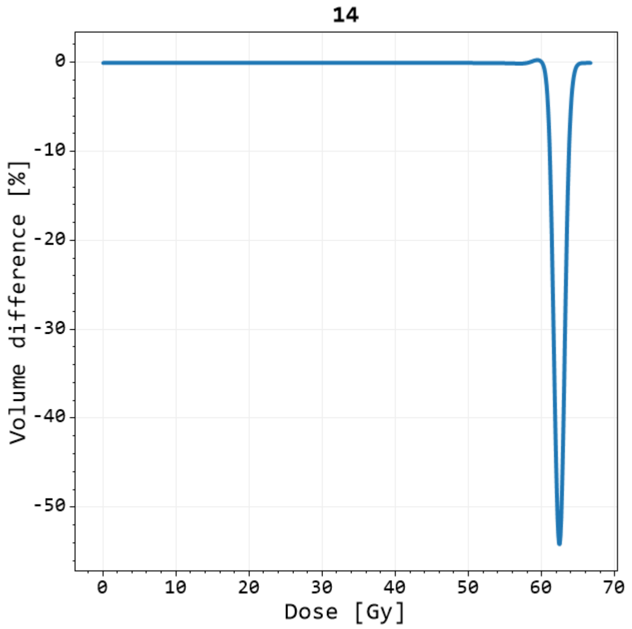
14
0
-10
-20
-30
-40
-50
Volume difference [%]
0 10 20 30 40 50 60 70
Dose [Gy]

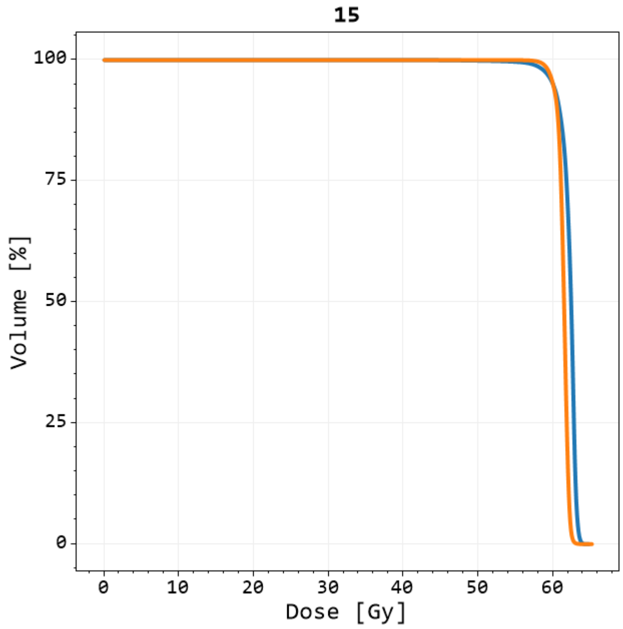
15
Volume [%]
Dose [Gy]

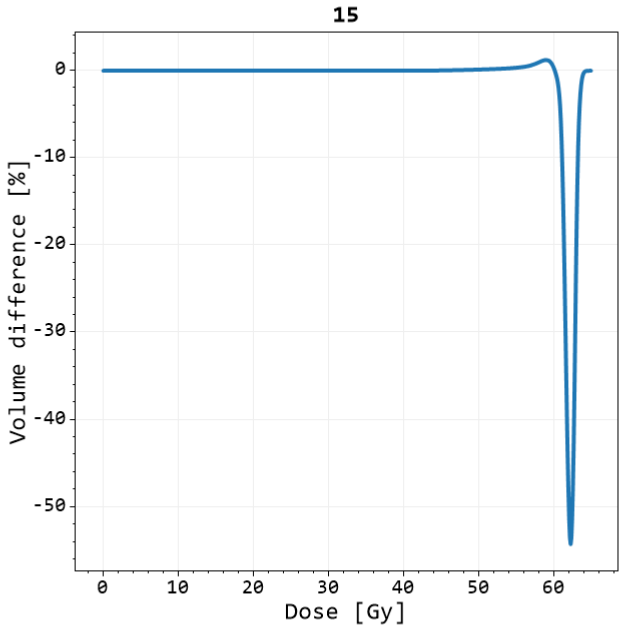
15
Volume difference [%]
Dose [Gy]

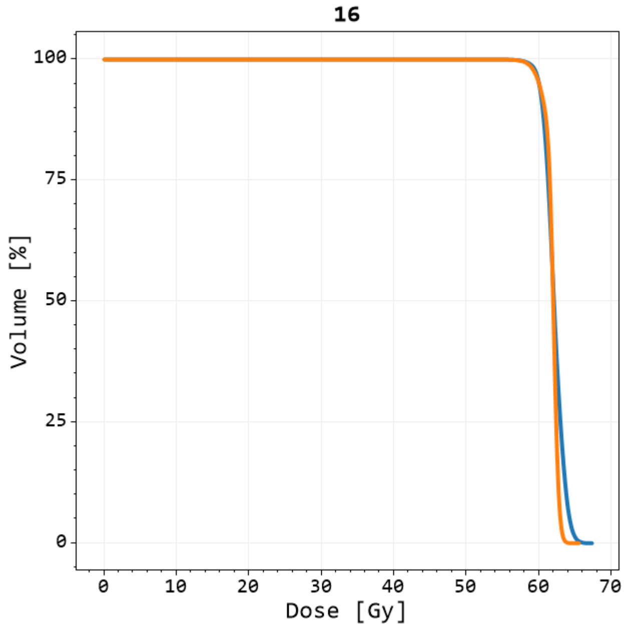
16
Volume [%]
Dose [Gy]

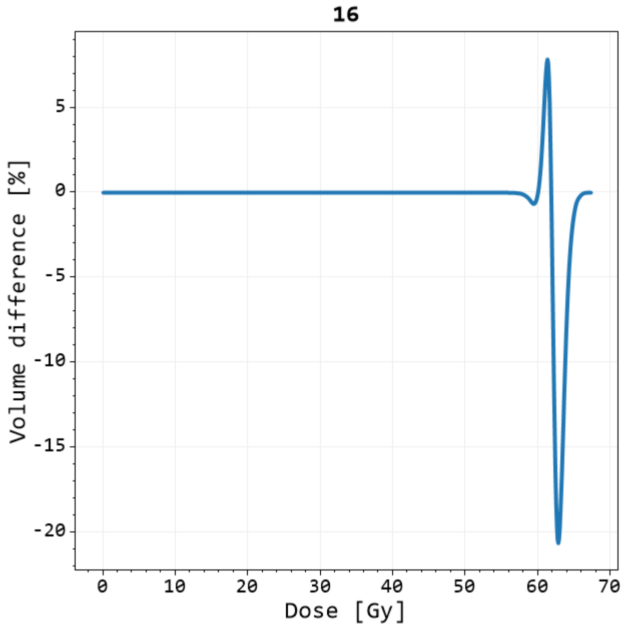
16
Volume difference [%]
Dose [Gy]

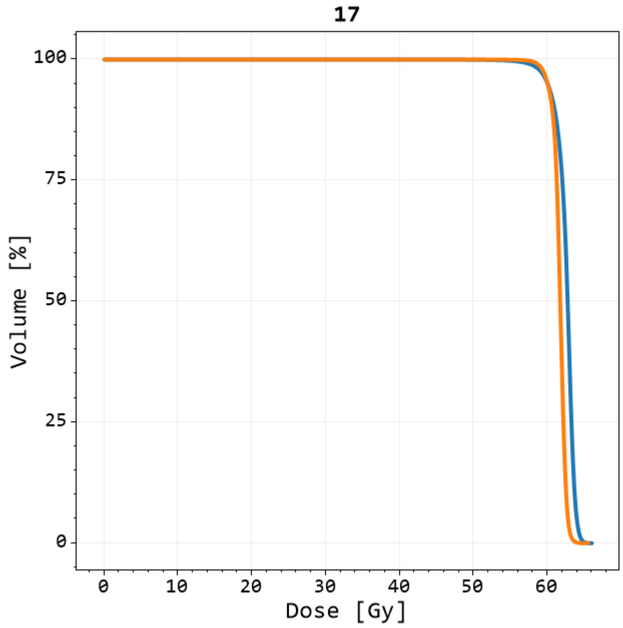
17
Volume [%]
Dose [Gy]

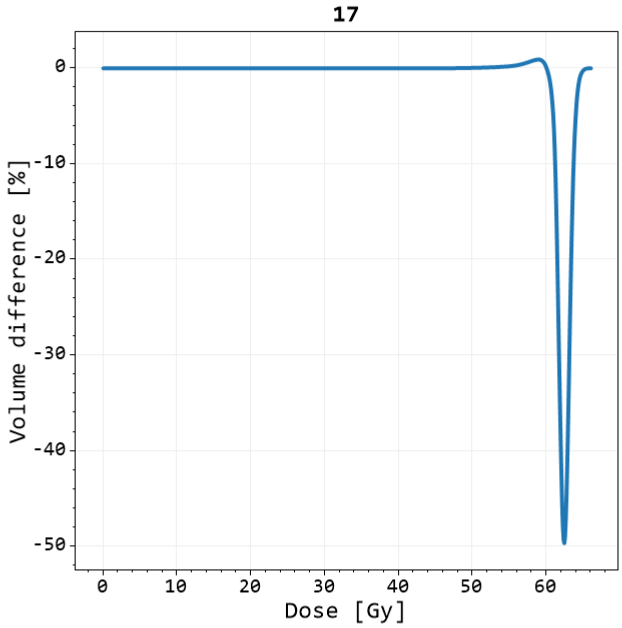
17
Volume difference [%]
Dose [Gy]

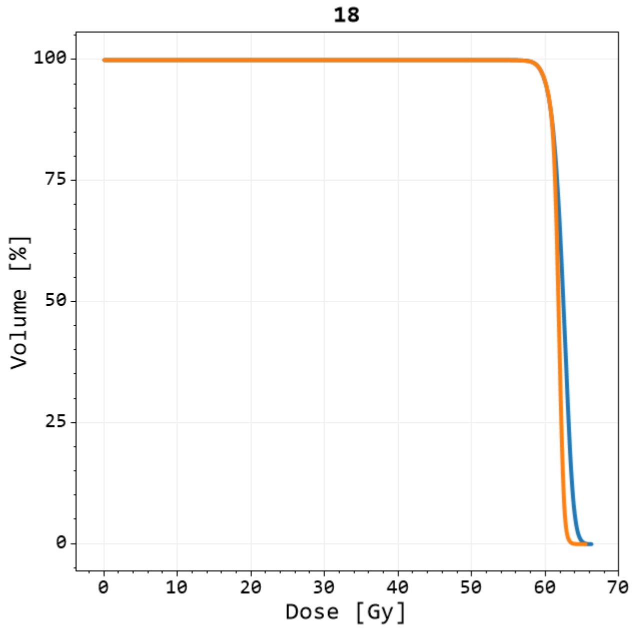
18
Volume [%]
100
75
50
25
0
0 10 20 30 40 50 60 70
Dose [Gy]

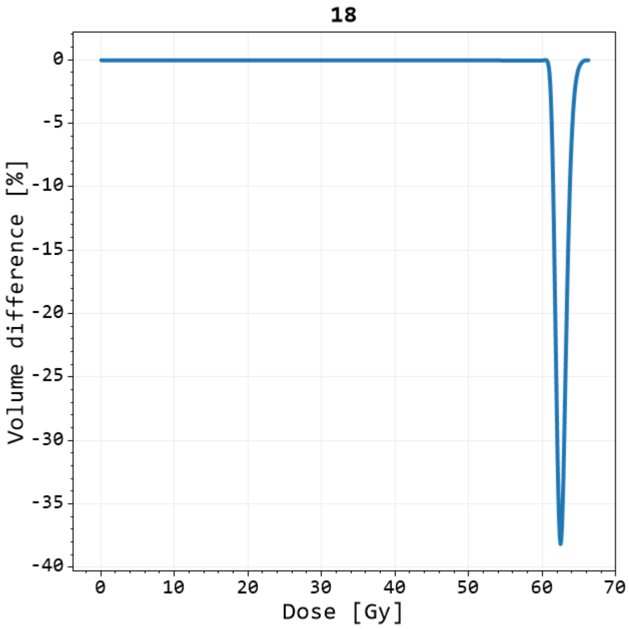
18
Volume difference [%]
0
-5
-10
-15
-20
-25
-30
-35
-40
0 10 20 30 40 50 60 70
Dose [Gy]

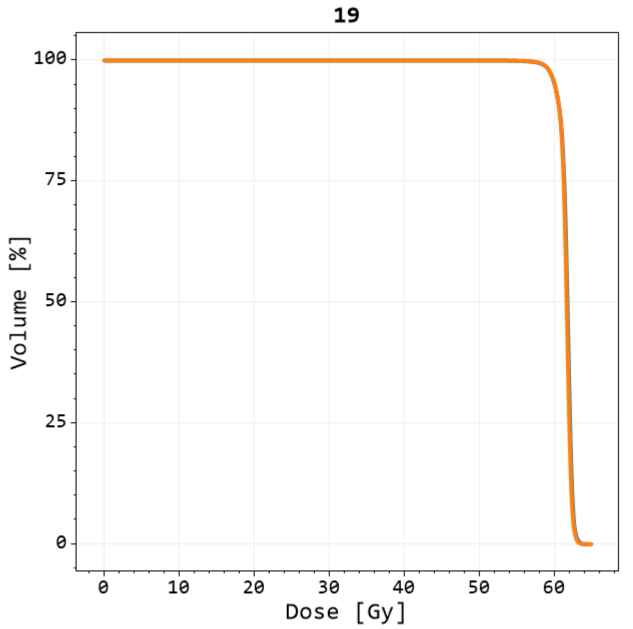
19
Volume [%]
100
75
50
25
0
0 10 20 30 40 50 60
Dose [Gy]

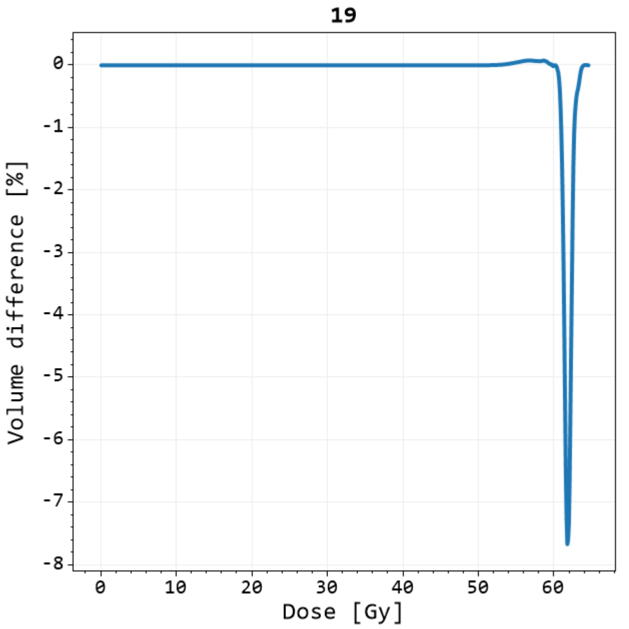
19
Volume difference [%]
0
-1
-2
-3
-4
-5
-6
-7
-8
0 10 20 30 40 50 60
Dose [Gy]

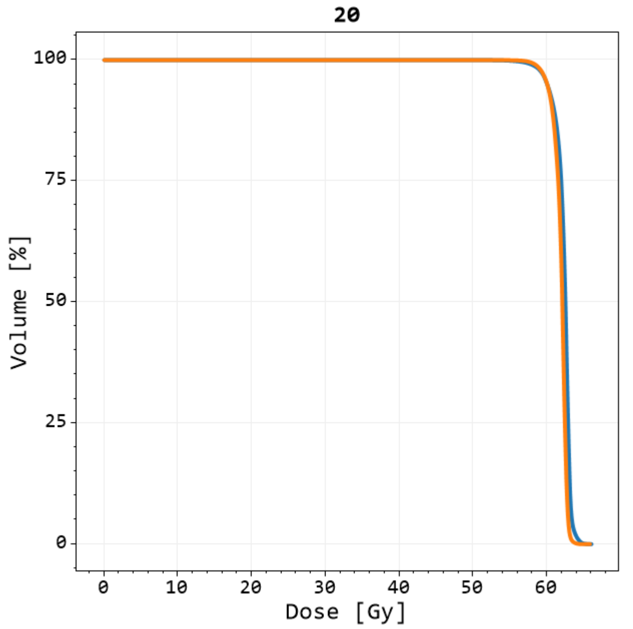
20
Volume [%]
100
75
50
25
0
0 10 20 30 40 50 60
Dose [Gy]

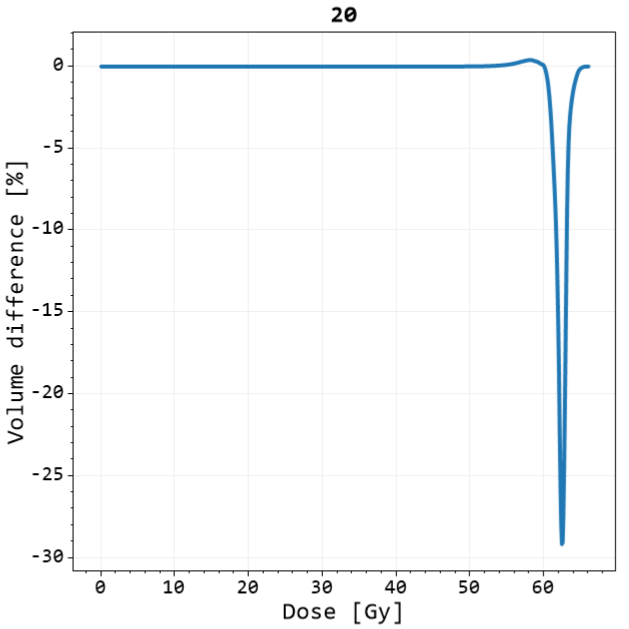
20
Volume difference [%]
0
-5
-10
-15
-20
-25
-30
0 10 20 30 40 50 60
Dose [Gy]

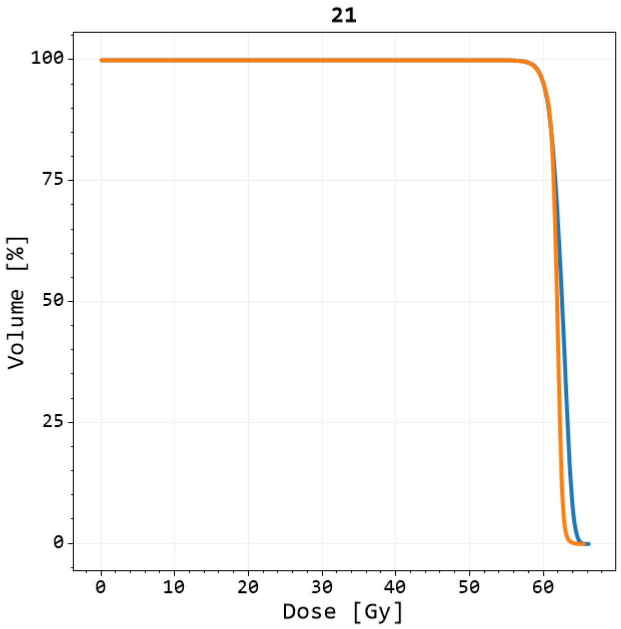
21
Volume [%]
Dose [Gy]

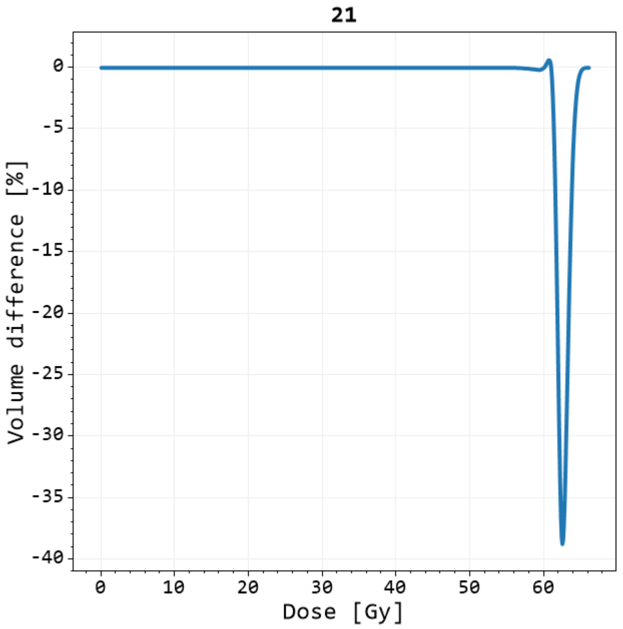
21
Volume difference [%]
Dose [Gy]

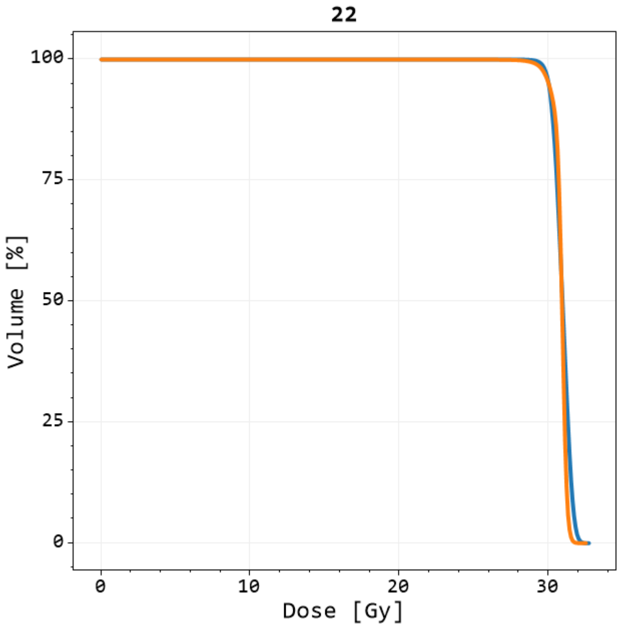
22
Volume [%]
Dose [Gy]

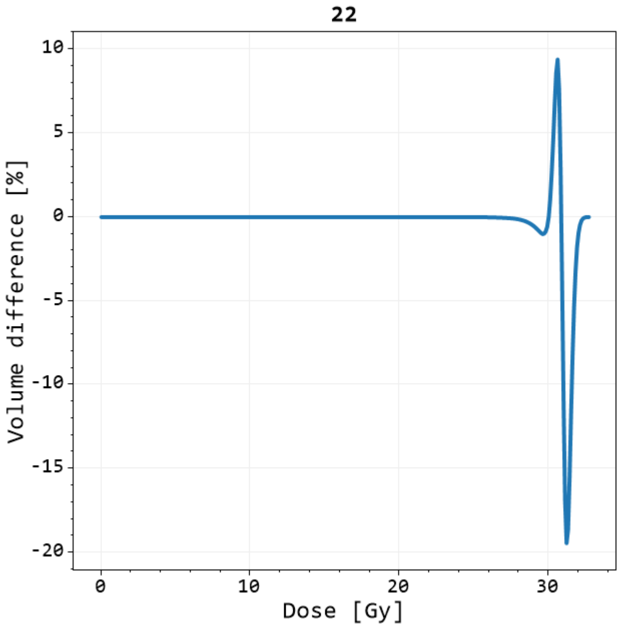
22
Volume difference [%]
Dose [Gy]

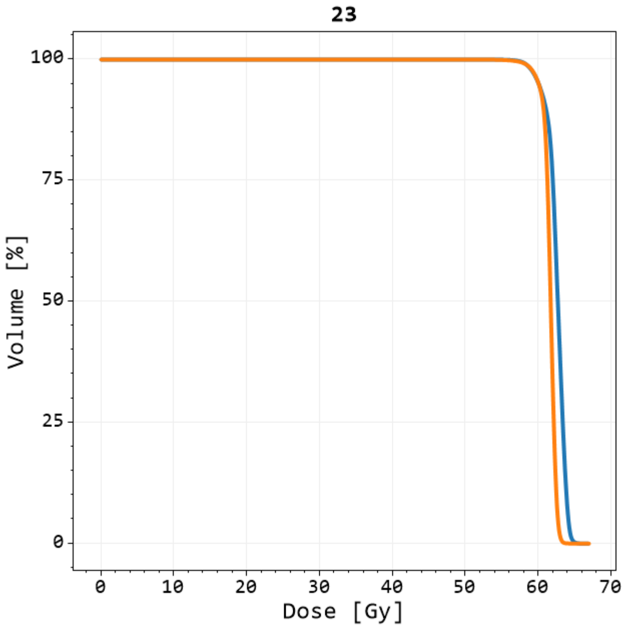
23
Volume [%]
Dose [Gy]

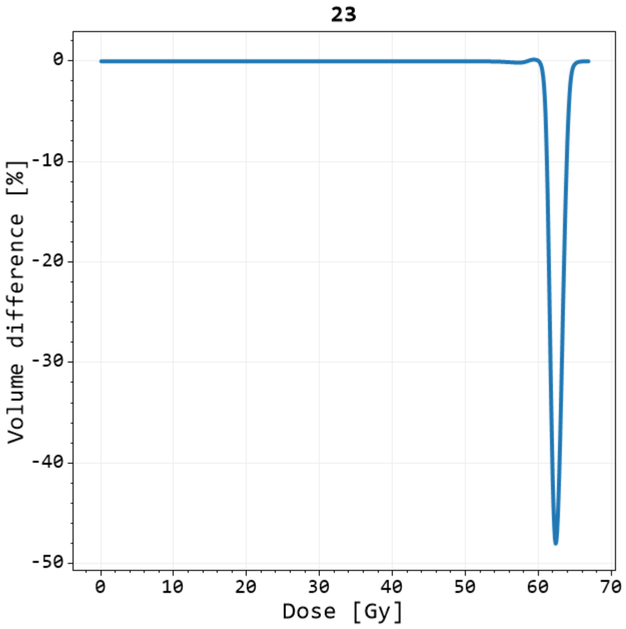
23
Volume difference [%]
Dose [Gy]

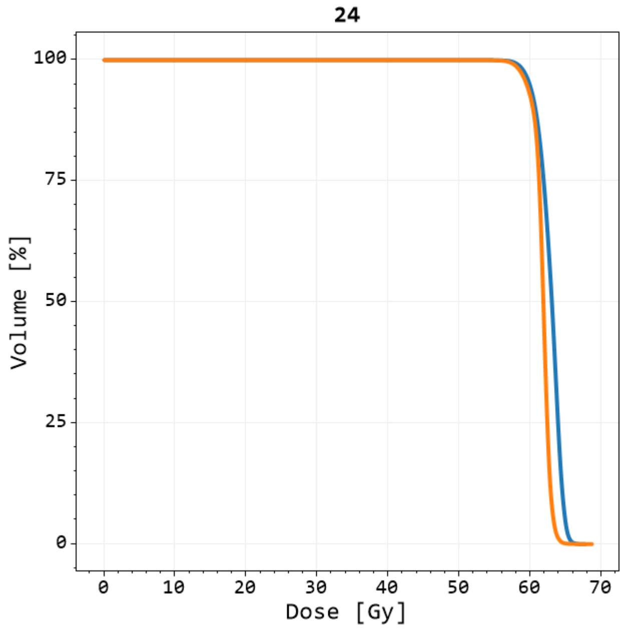
24
Volume [%]
Dose [Gy]
100
75
50
25
0
0 10 20 30 40 50 60 70

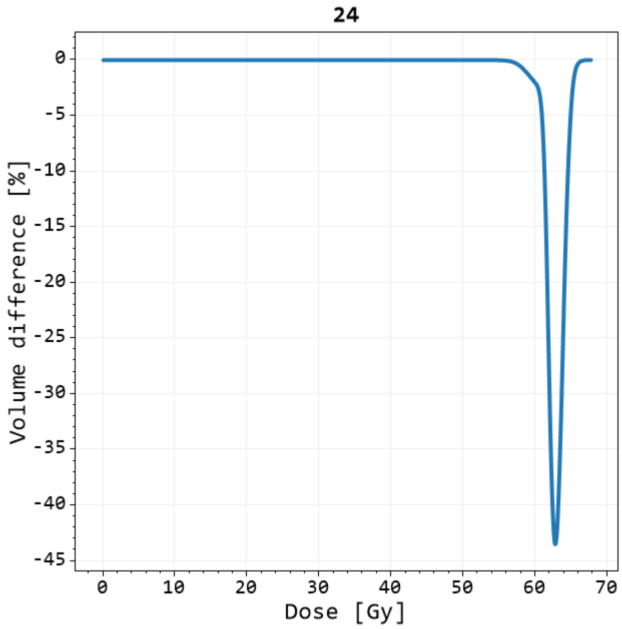
24
Volume difference [%]
Dose [Gy]
0
-5
-10
-15
-20
-25
-30
-35
-40
-45
0 10 20 30 40 50 60 70

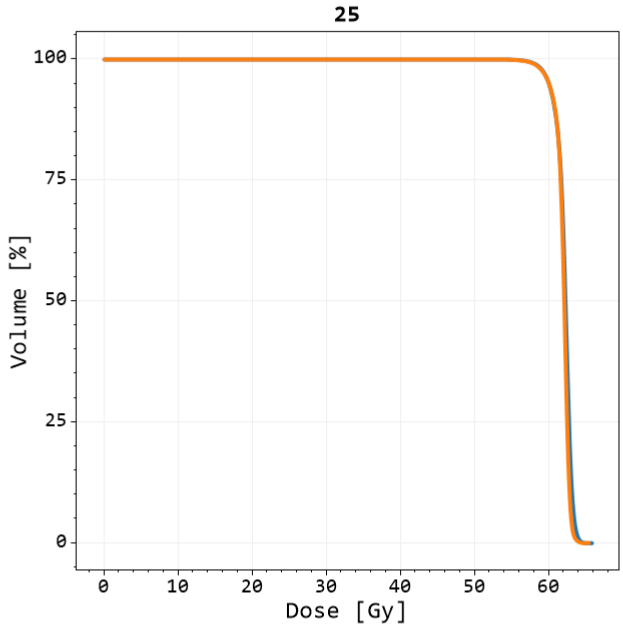
25
Volume [%]
Dose [Gy]
100
75
50
25
0
0 10 20 30 40 50 60

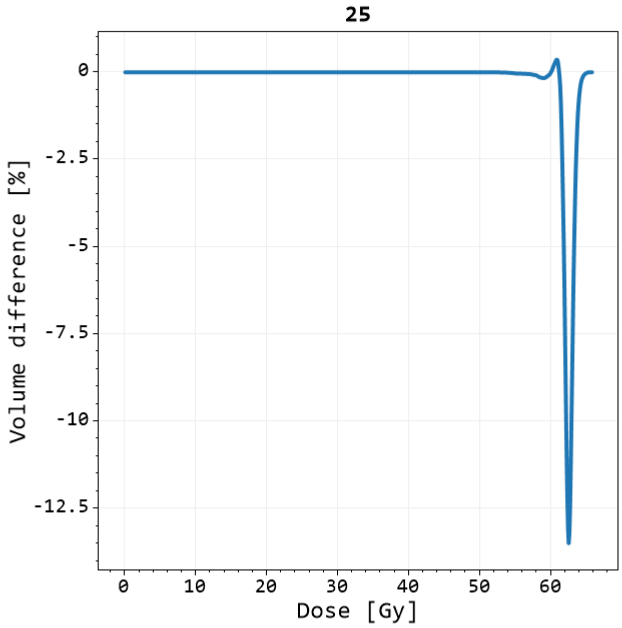
25
Volume difference [%]
Dose [Gy]
0
-2.5
-5
-7.5
-10
-12.5
0 10 20 30 40 50 60

# BrachPlex_Ipsi

## DVH Volume difference

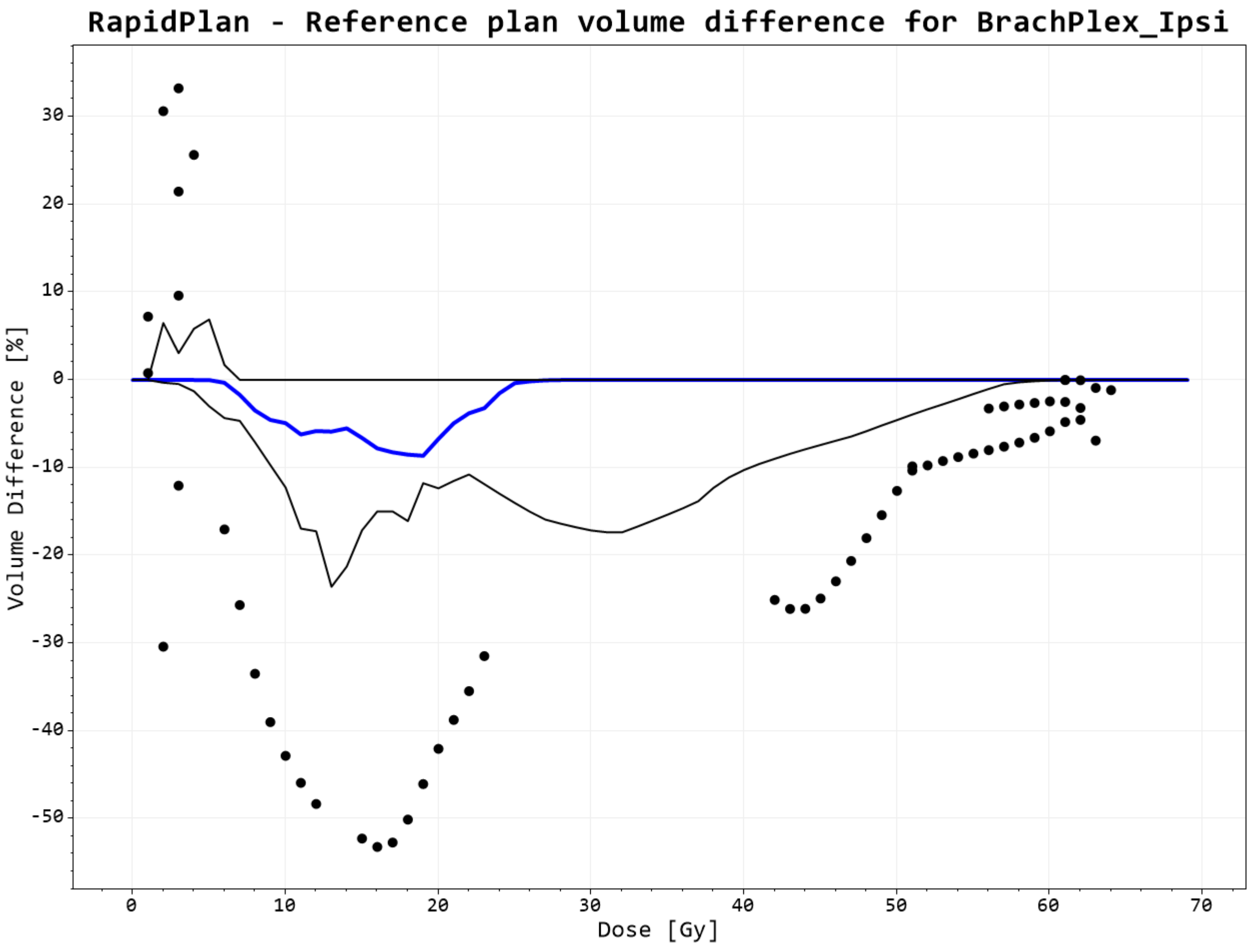


## Dose-volume metric summary table

| Metric | Reference Plan | RapidPlan | Difference |
|---|---|---|---|
| DMax[Gy] | 30.8 [6.9,61.5] | 31.5 [7.7,59.8] | -1.0 [-1.8,0.8] (p = 0.910) (1) |
| V66Gy[%] | 0.0 [0.0,0.0] | 0.0 [0.0,0.0] | 0.0 [0.0,0.0] (p = 1.000) (0) |

## Dose-volume metric box whisker plots

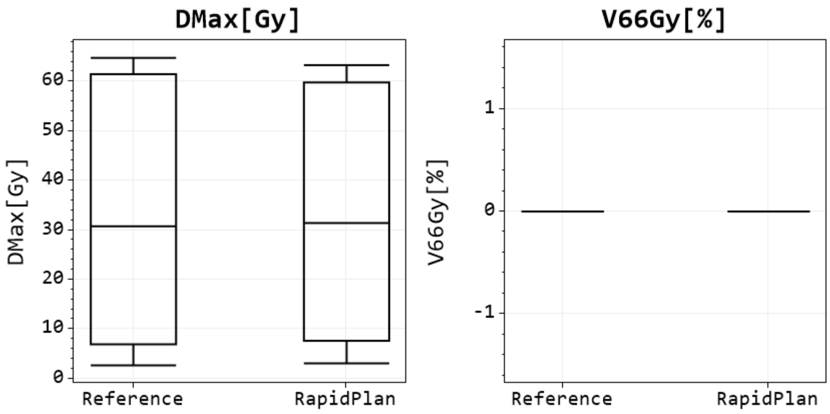

## Dose-volume metric differences by plan

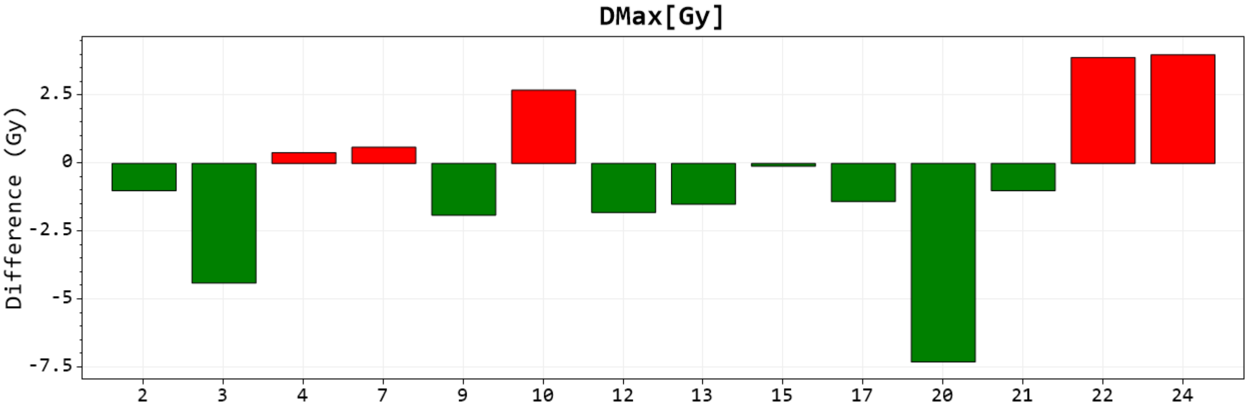


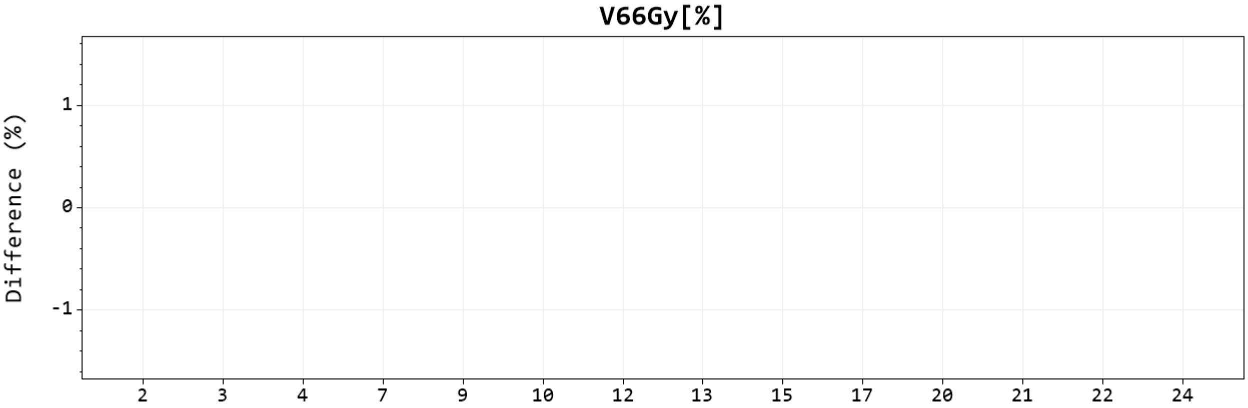

## Dose-volume histograms

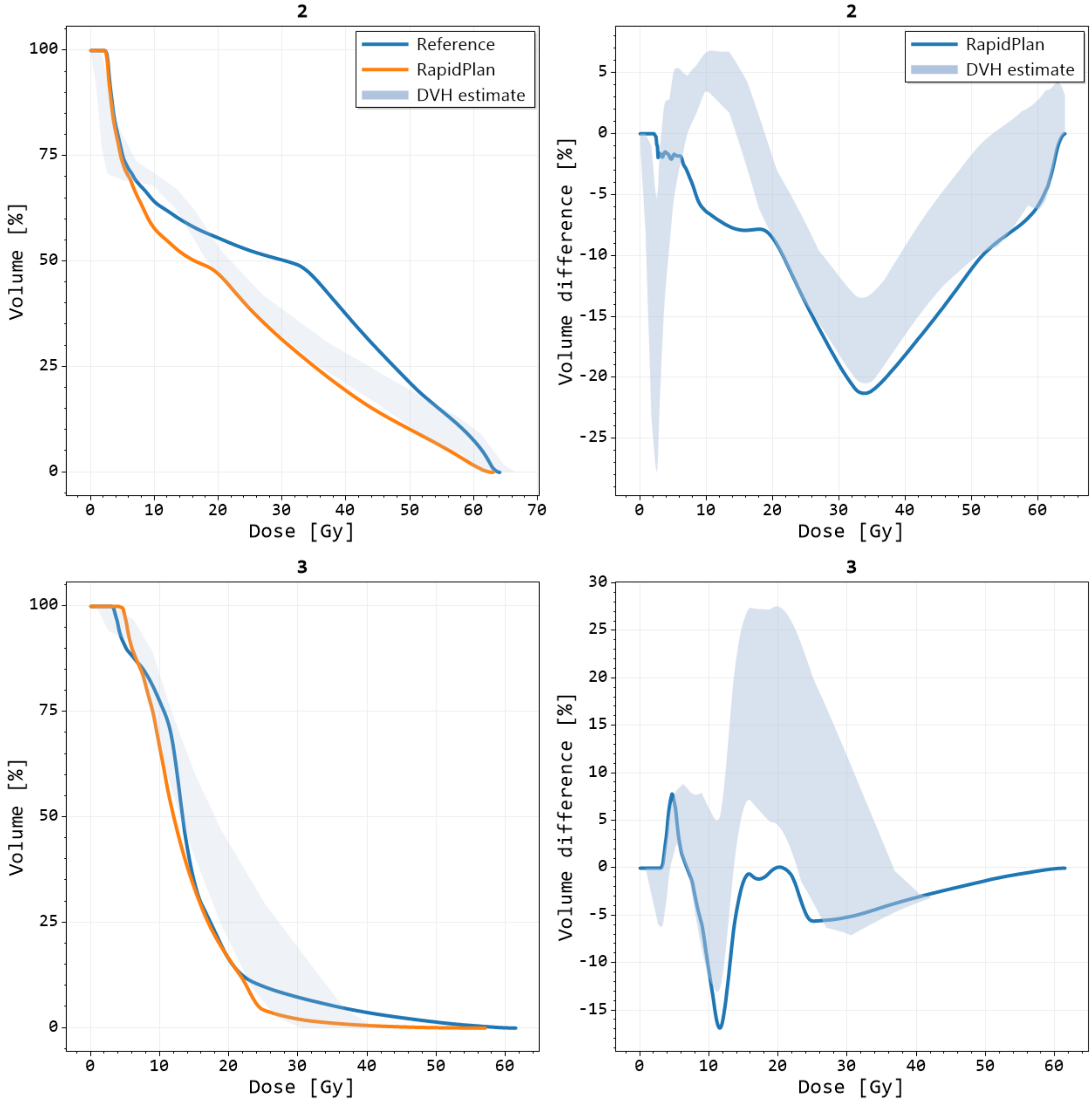

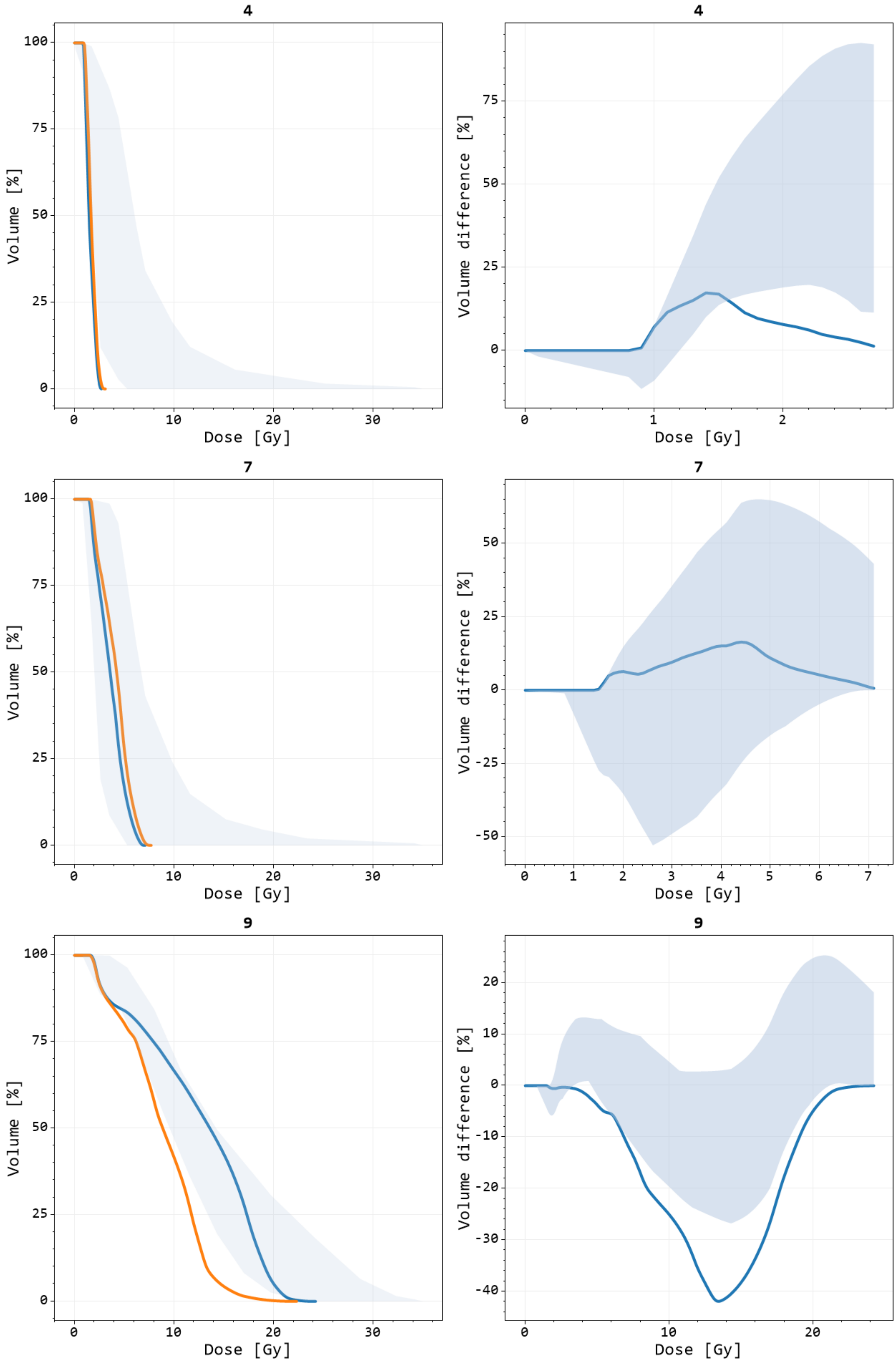
4
Volume [%]
Dose [Gy]
4
Volume difference [%]
Dose [Gy]
7
Volume [%]
Dose [Gy]
7
Volume difference [%]
Dose [Gy]
9
Volume [%]
Dose [Gy]
9
Volume difference [%]
Dose [Gy]

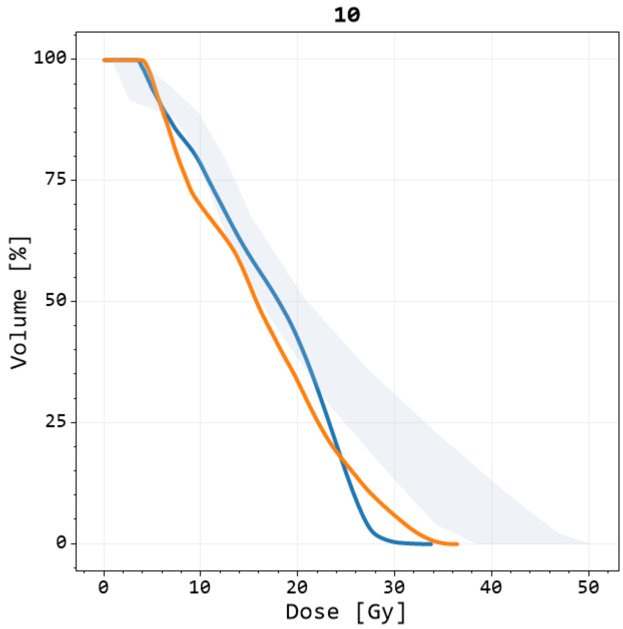
10
Volume [%]
100
75
50
25
0
0 10 20 30 40 50
Dose [Gy]

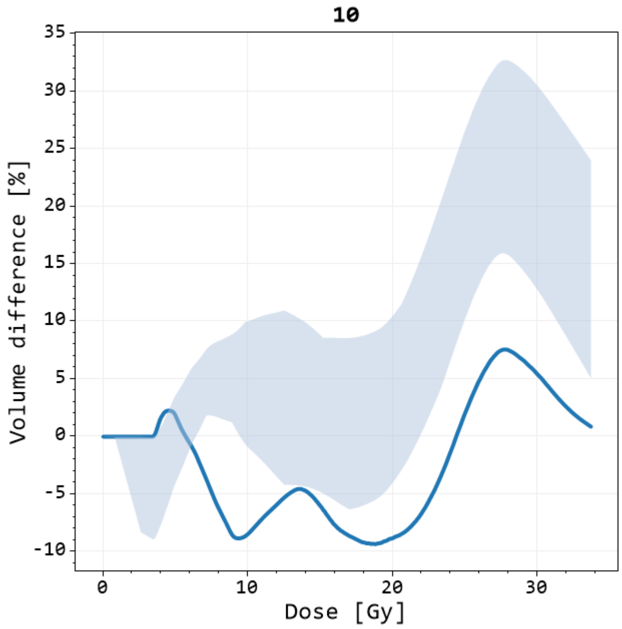
10
Volume difference [%]
35 30 25 20 15 10 5 0 -5 -10
0 10 20 30
Dose [Gy]

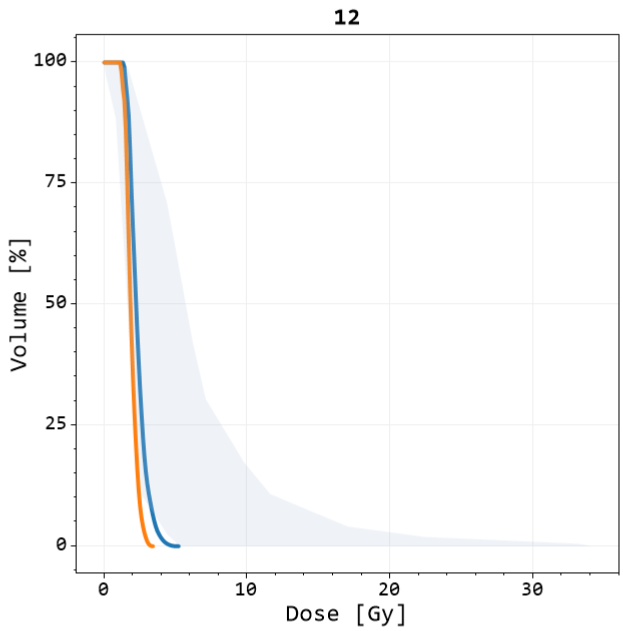
12
Volume [%]
100
75
50
25
0
0 10 20 30
Dose [Gy]

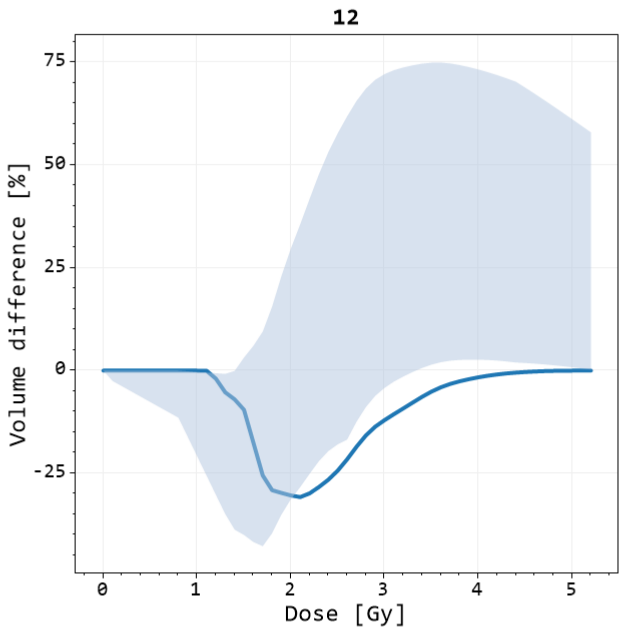
12
Volume difference [%]
75 50 25 0 -25
0 1 2 3 4 5
Dose [Gy]

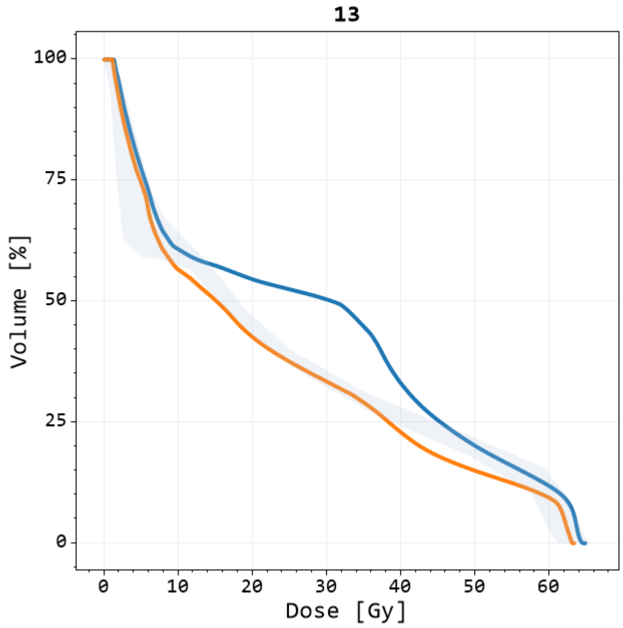
13
Volume [%]
100
75
50
25
0
0 10 20 30 40 50 60
Dose [Gy]

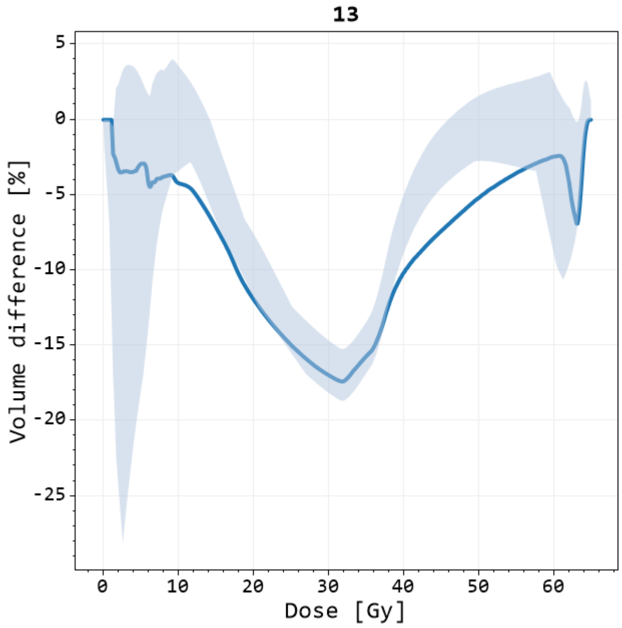
13
Volume difference [%]
5 0 -5 -10 -15 -20 -25
0 10 20 30 40 50 60
Dose [Gy]

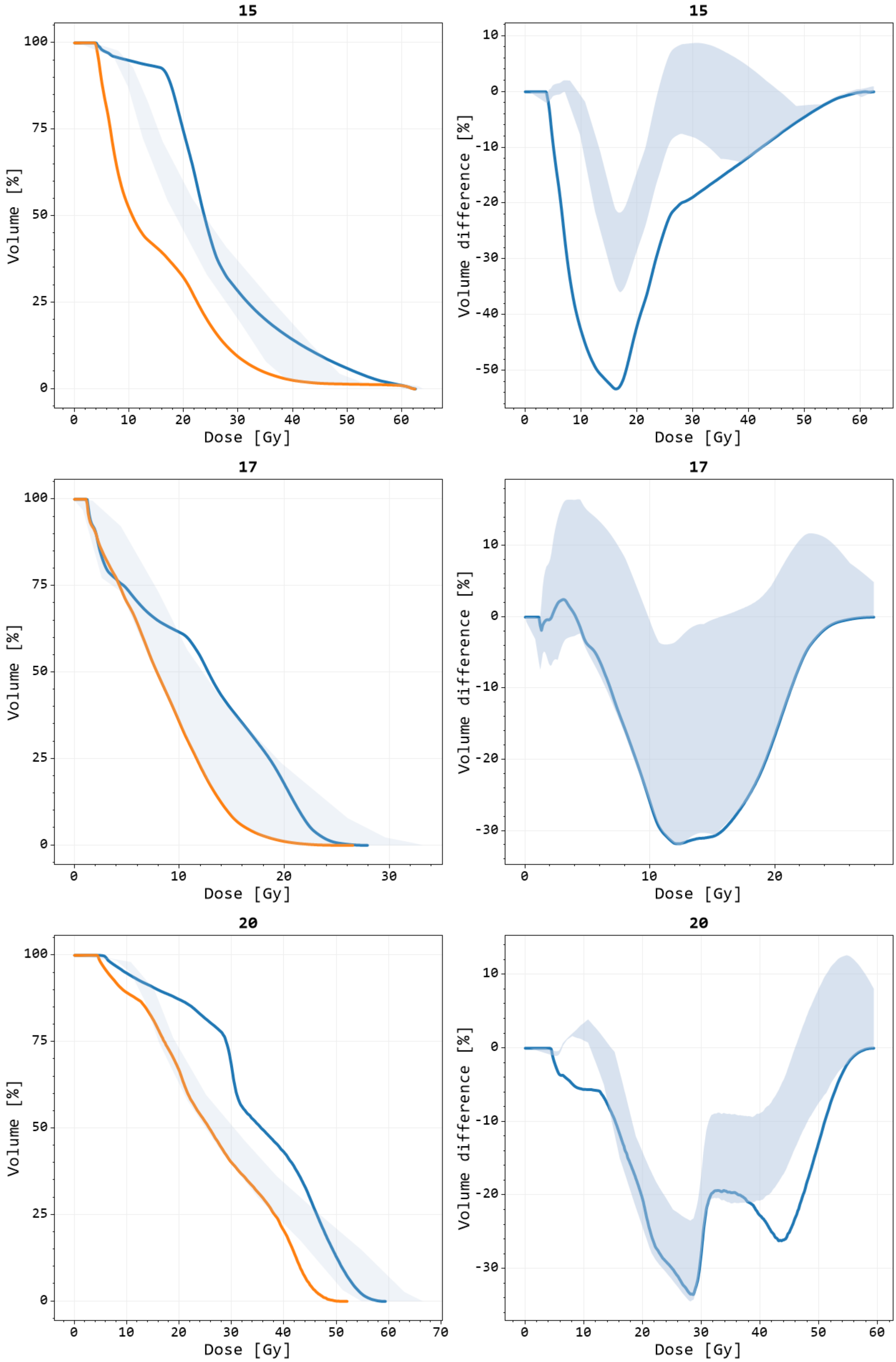
15
Volume [%]
Dose [Gy]
0
25
50
75
100
0 10 20 30 40 50 60
15
Volume difference [%]
Dose [Gy]
10
0
-10
-20
-30
-40
-50
0 10 20 30 40 50 60
17
Volume [%]
Dose [Gy]
0
25
50
75
100
0 10 20 30
17
Volume difference [%]
Dose [Gy]
10
0
-10
-20
-30
0 10 20
20
Volume [%]
Dose [Gy]
0
25
50
75
100
0 10 20 30 40 50 60 70
20
Volume difference [%]
Dose [Gy]
10
0
-10
-20
-30
0 10 20 30 40 50 60

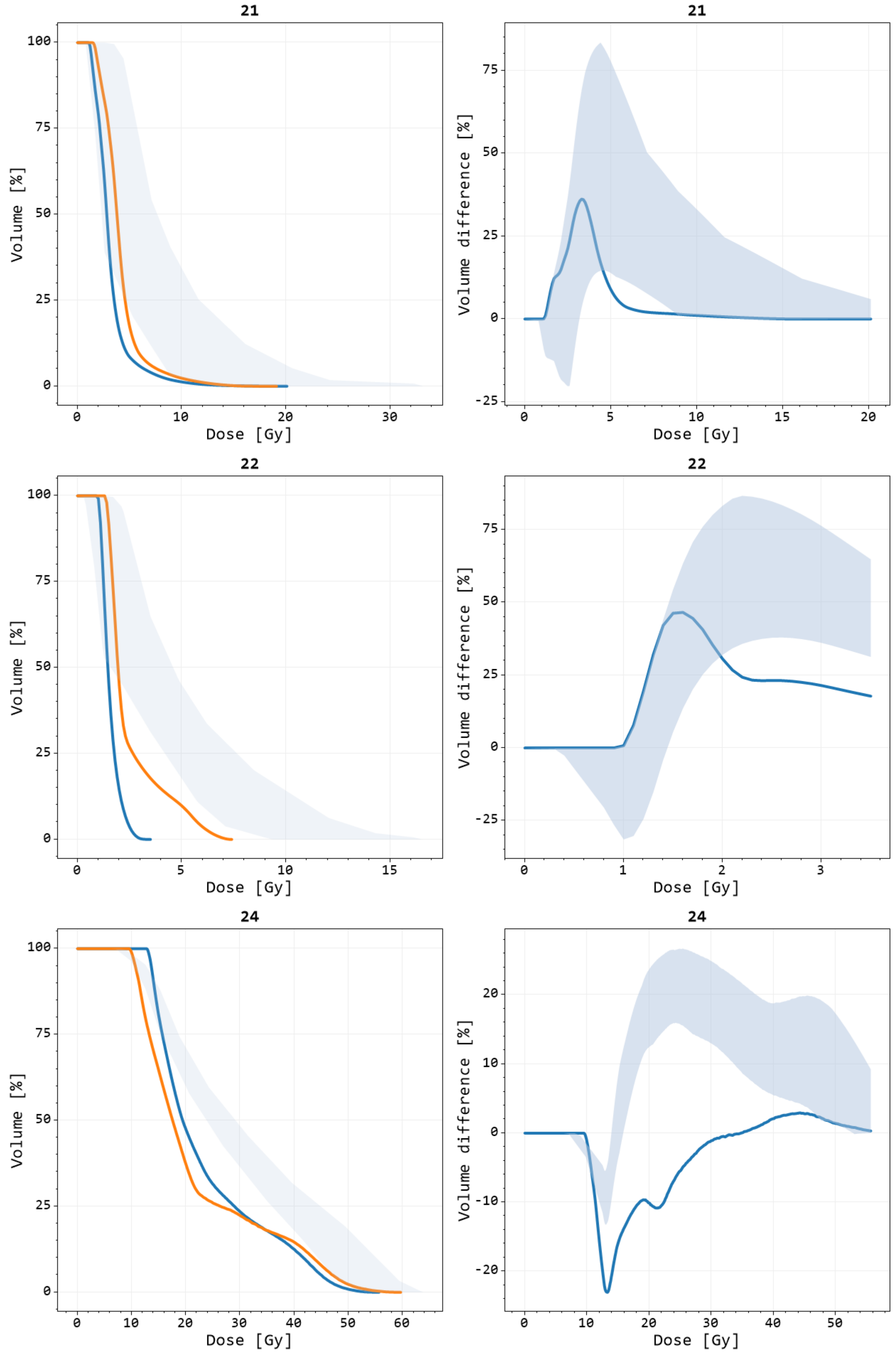
21
Volume [%]
Dose [Gy]
21
Volume difference [%]
Dose [Gy]
22
Volume [%]
Dose [Gy]
22
Volume difference [%]
Dose [Gy]
24
Volume [%]
Dose [Gy]
24
Volume difference [%]
Dose [Gy]

# SpinalCord_PRV03

## DVH Volume difference

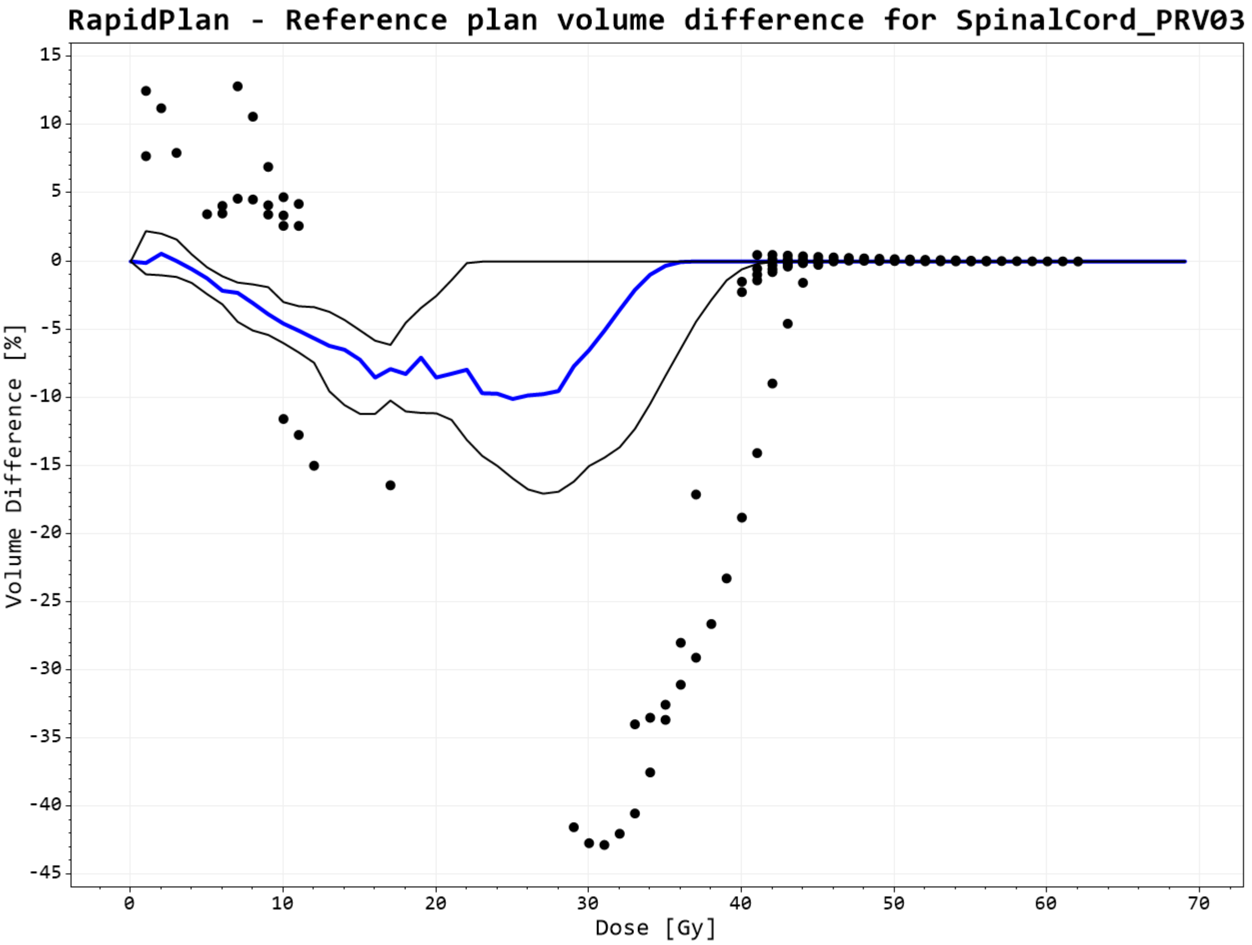


## Dose-volume metric summary table

| Metric | Reference Plan | RapidPlan | Difference |
| --- | --- | --- | --- |

## Dose-volume metric box whisker plots

## Dose-volume metric differences by plan

## Dose-volume histograms

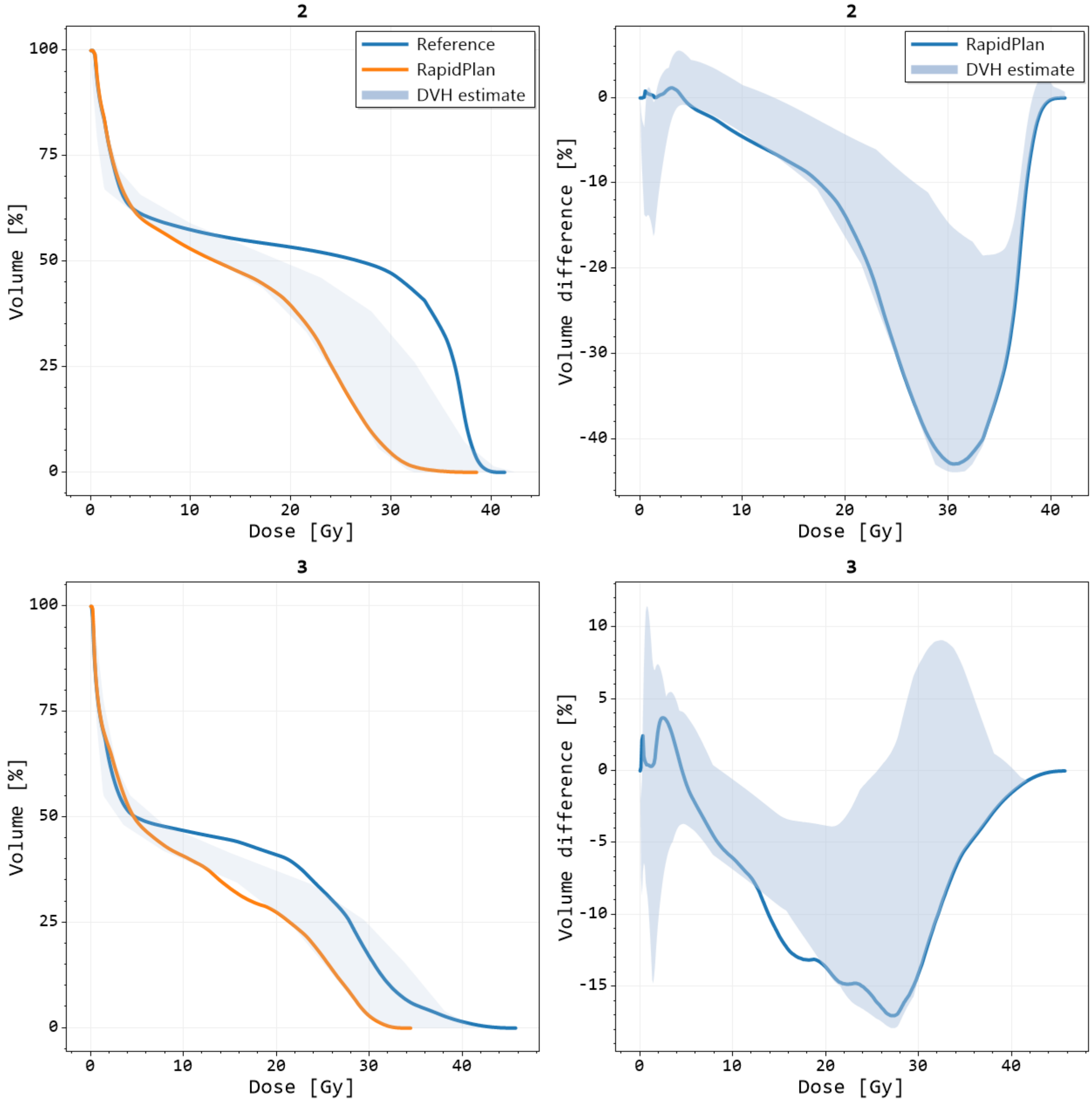

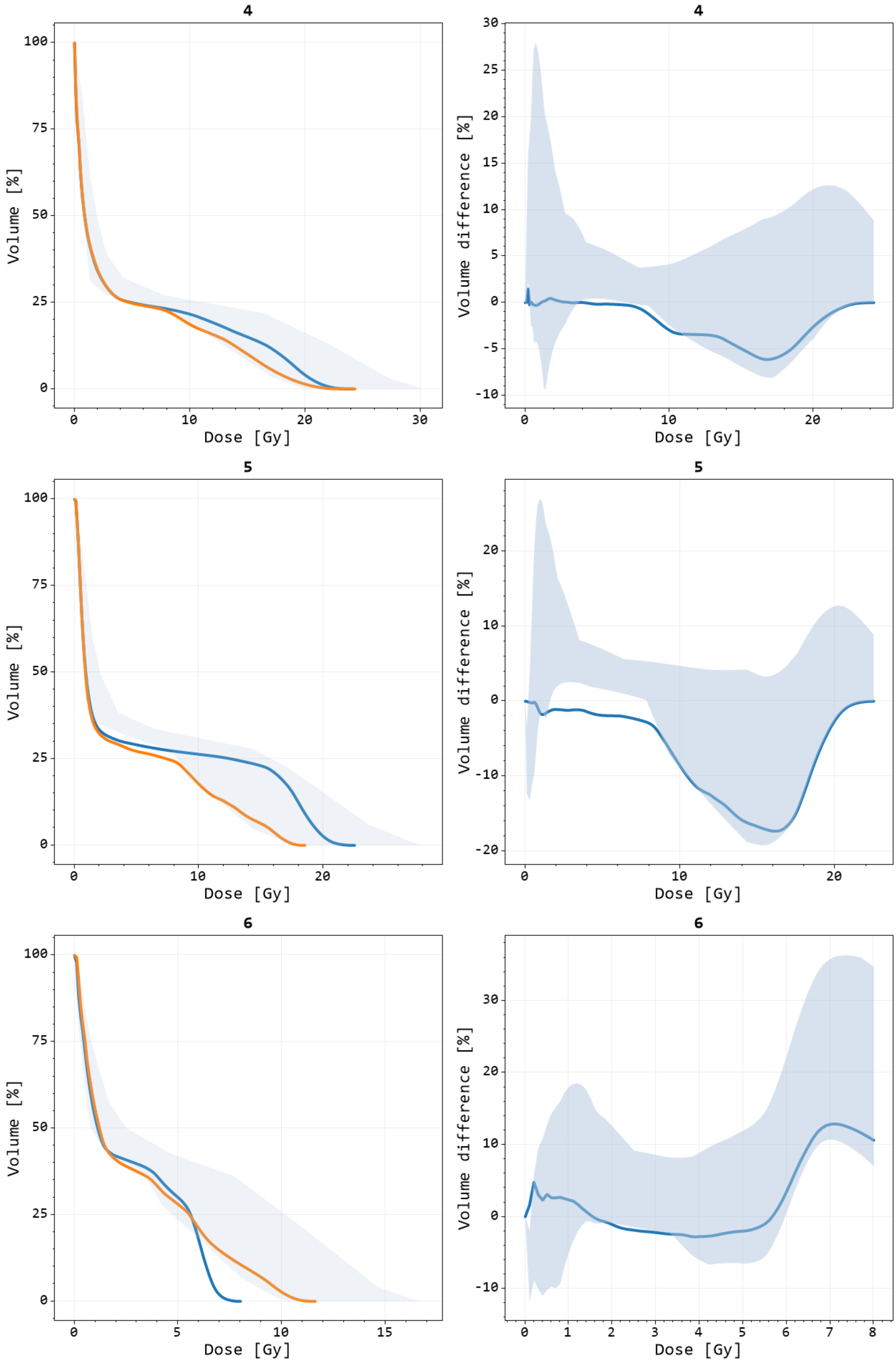
4
Volume [%]
Dose [Gy]
4
Volume difference [%]
Dose [Gy]
5
Volume [%]
Dose [Gy]
5
Volume difference [%]
Dose [Gy]
6
Volume [%]
Dose [Gy]
6
Volume difference [%]
Dose [Gy]

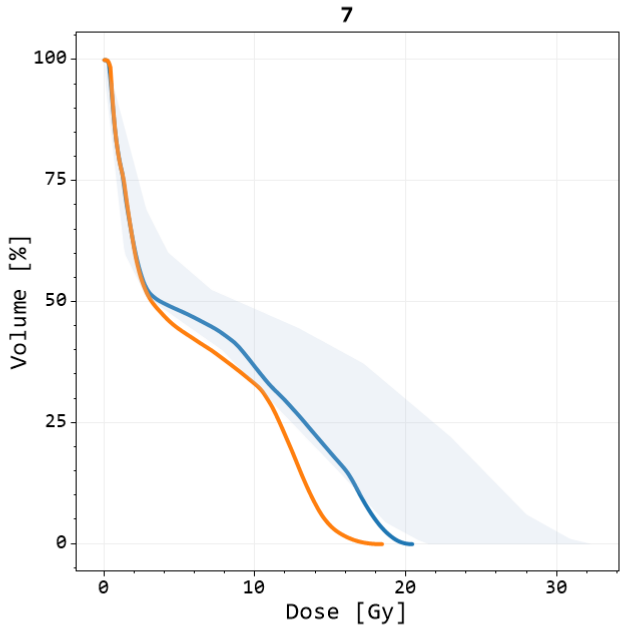
7
100
75
50
25
0
Volume [%]
0
10
20
30
Dose [Gy]

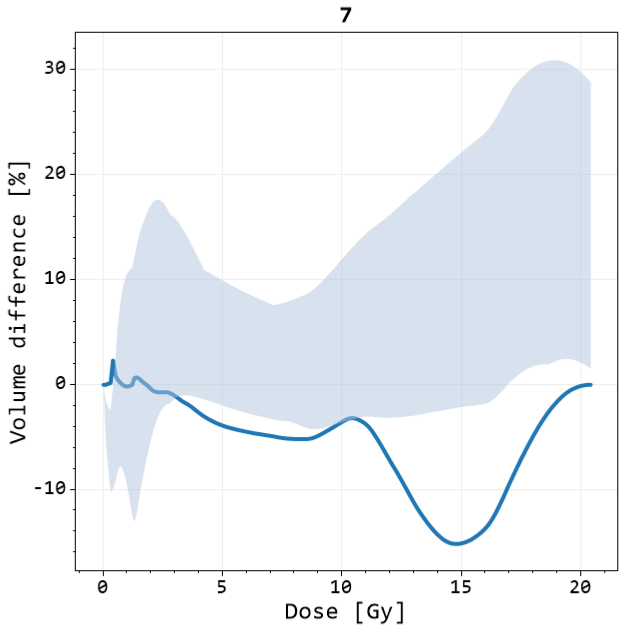
7
30
20
10
0
-10
Volume difference [%]
0
5
10
15
20
Dose [Gy]

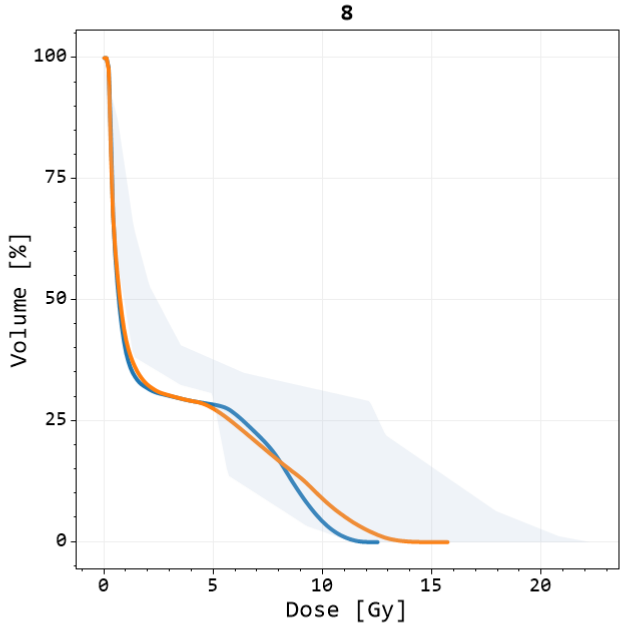
8
100
75
50
25
0
Volume [%]
0
5
10
15
20
Dose [Gy]

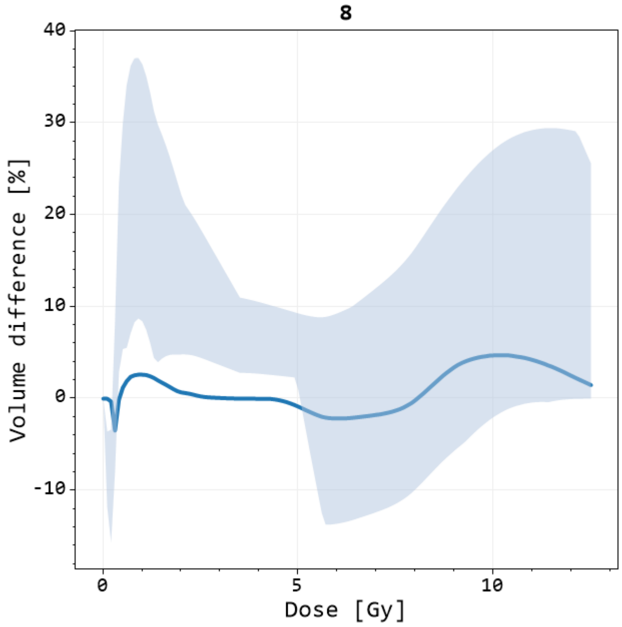
8
40
30
20
10
0
-10
Volume difference [%]
0
5
10
Dose [Gy]

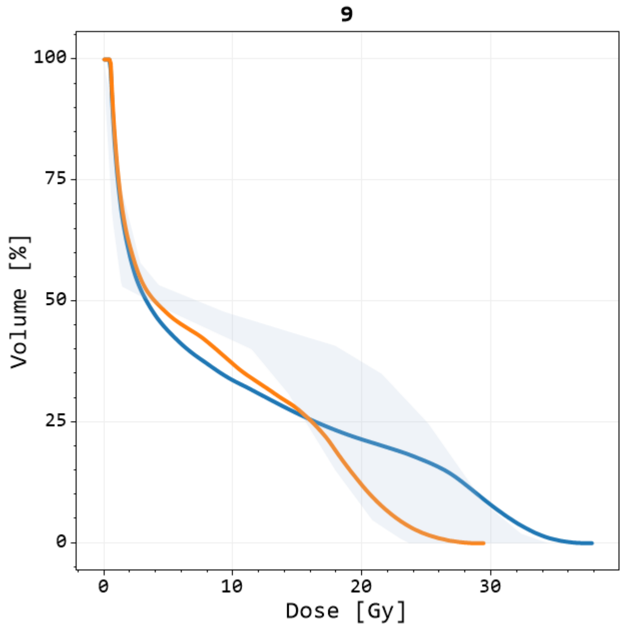
9
100
75
50
25
0
Volume [%]
0
10
20
30
Dose [Gy]

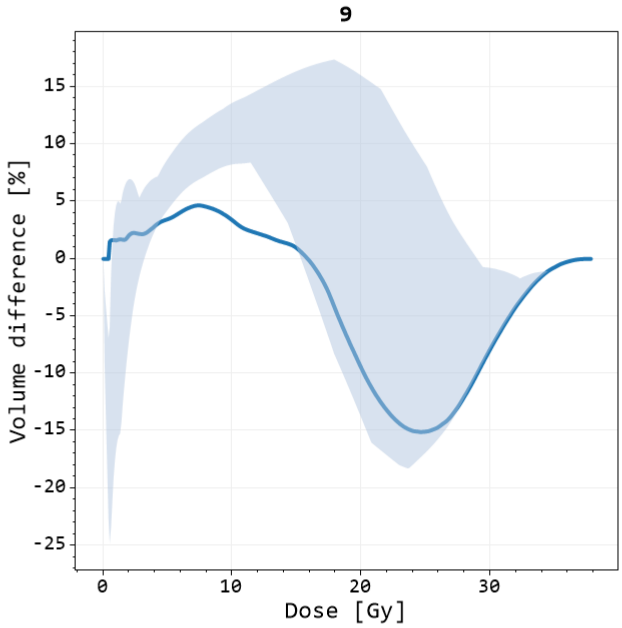
9
15
10
5
0
-5
-10
-15
-20
-25
Volume difference [%]
0
10
20
30
Dose [Gy]

10

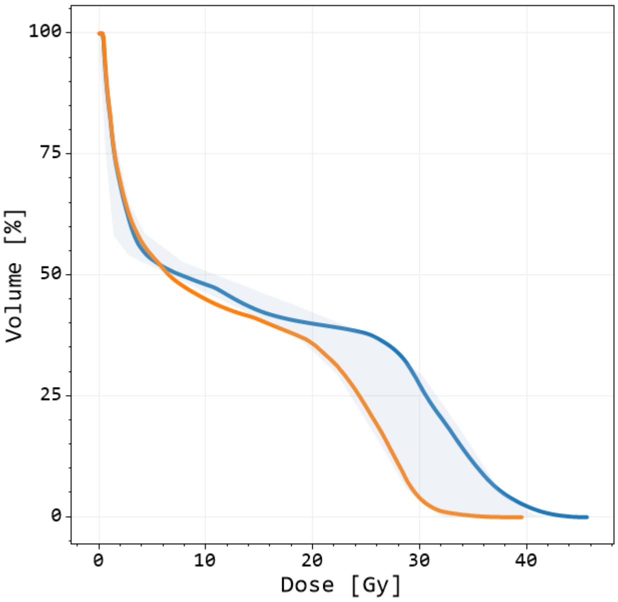


10

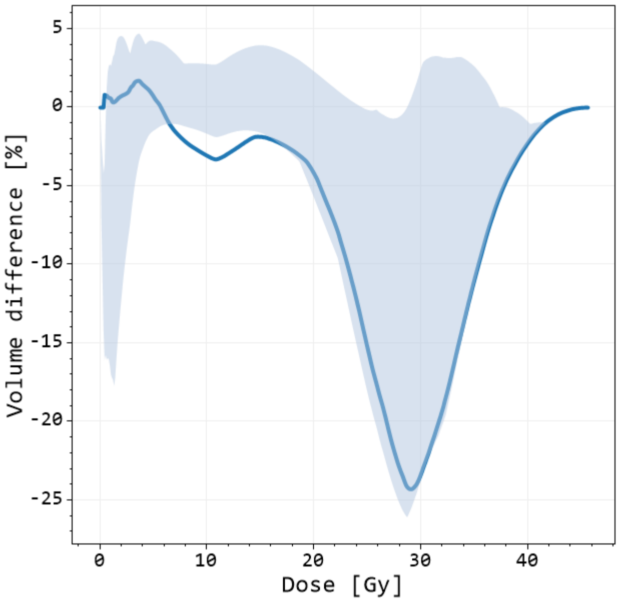


11

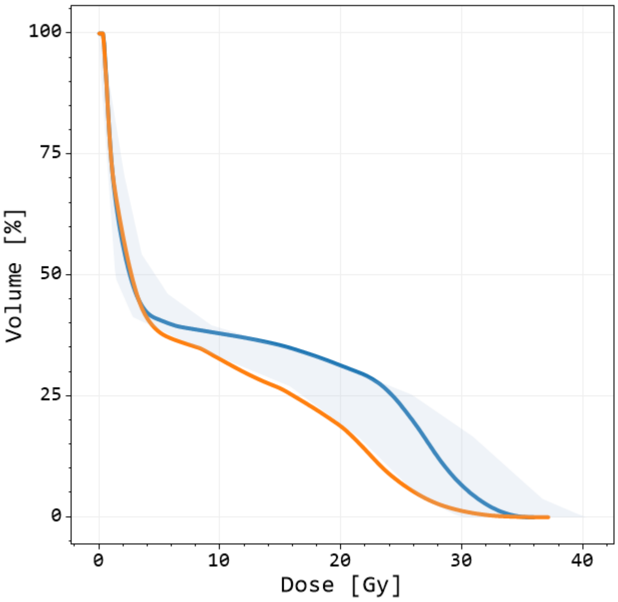


11

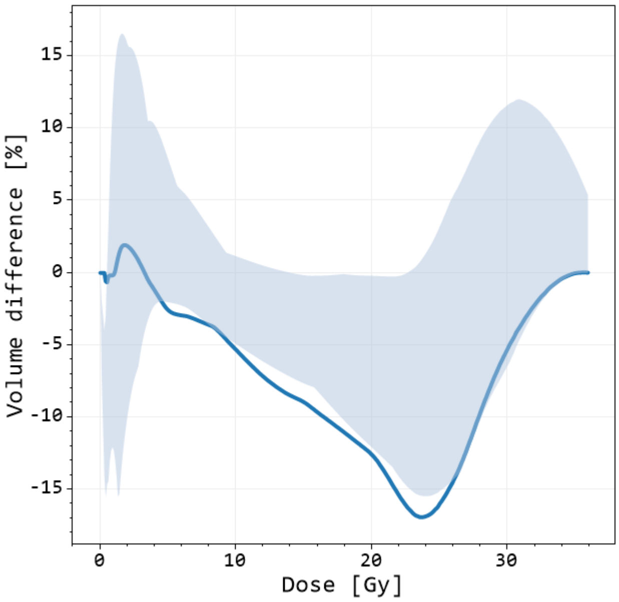


12

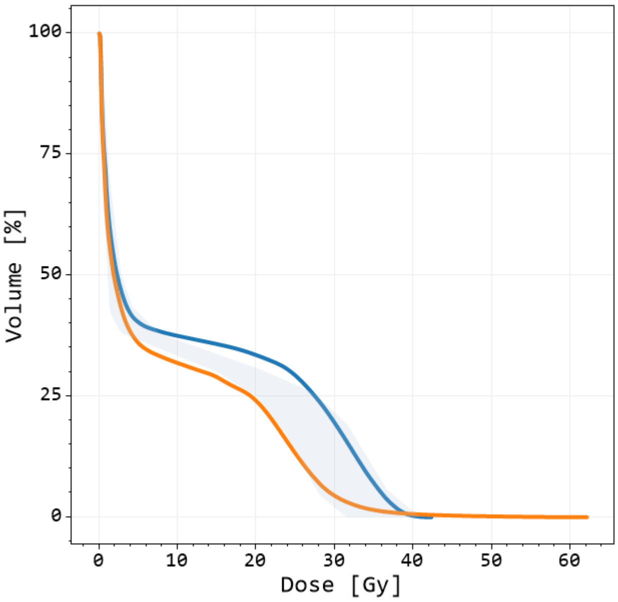


12

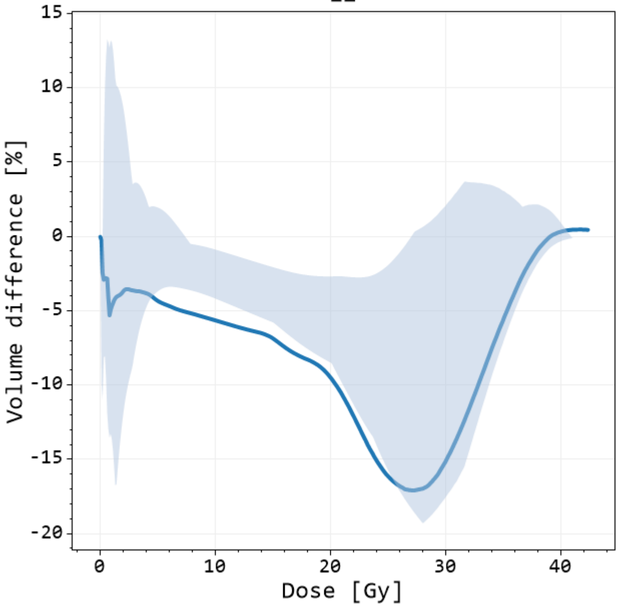

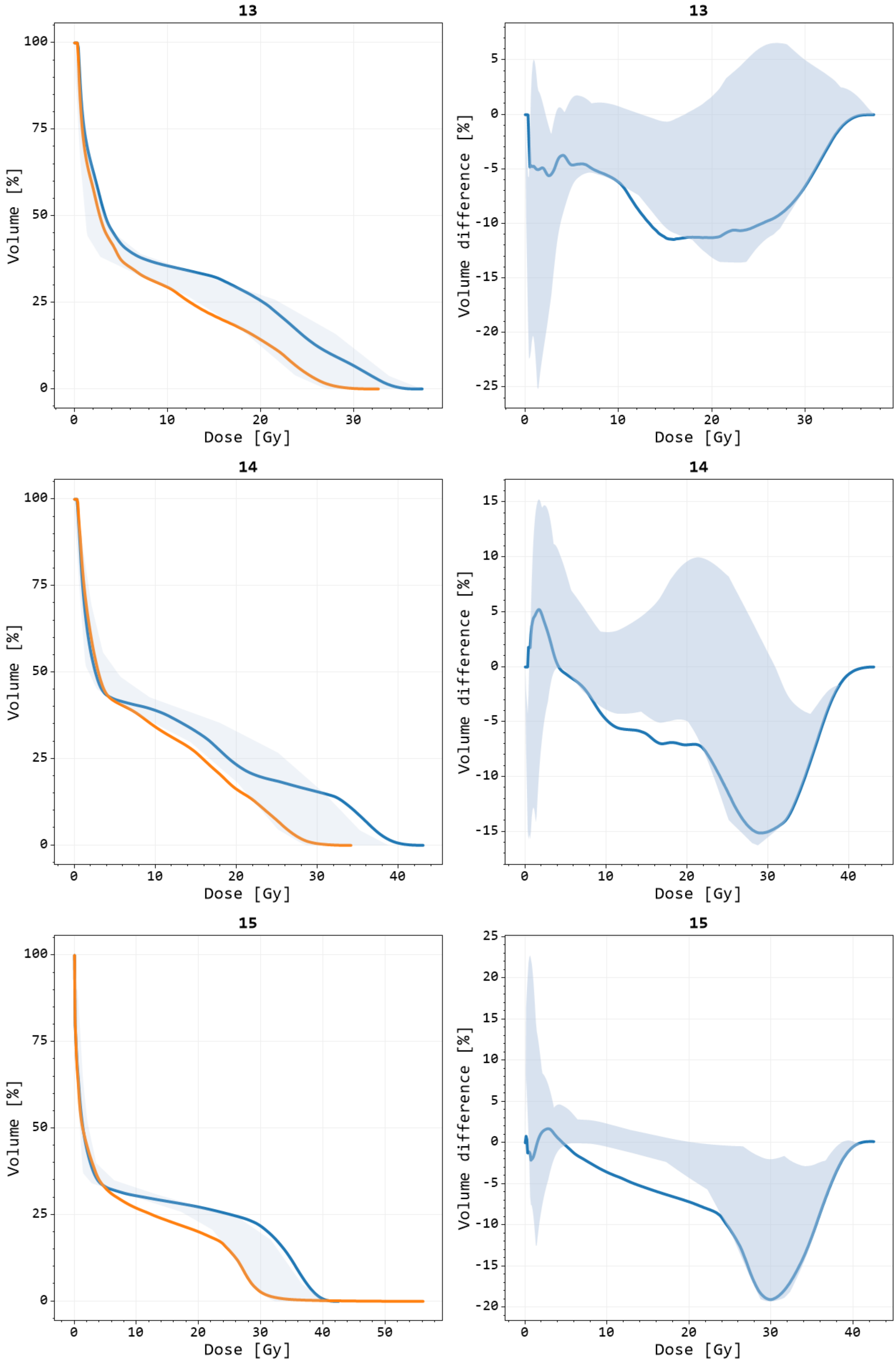
13
Volume [%]
Dose [Gy]
Volume difference [%]
14
Volume [%]
Dose [Gy]
Volume difference [%]
15
Volume [%]
Dose [Gy]
Volume difference [%]

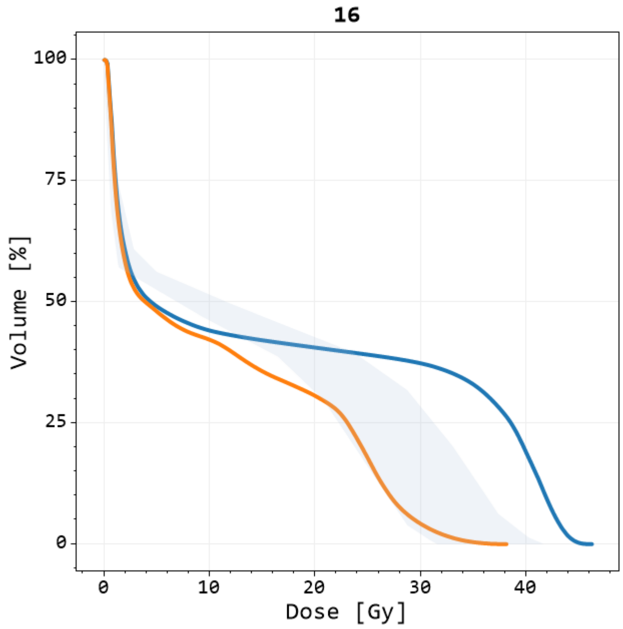
16
100
75
50
25
0
Volume [%]
0
10
20
30
40
Dose [Gy]

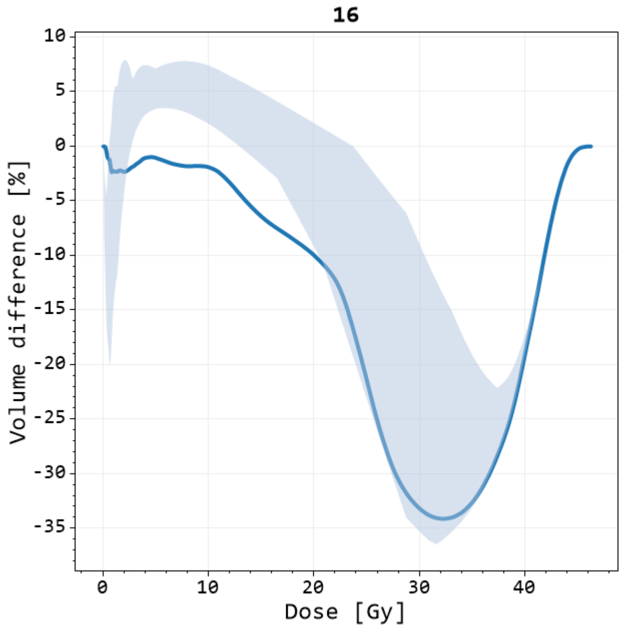
16
10
5
0
-5
-10
-15
-20
-25
-30
-35
Volume difference [%]
0
10
20
30
40
Dose [Gy]

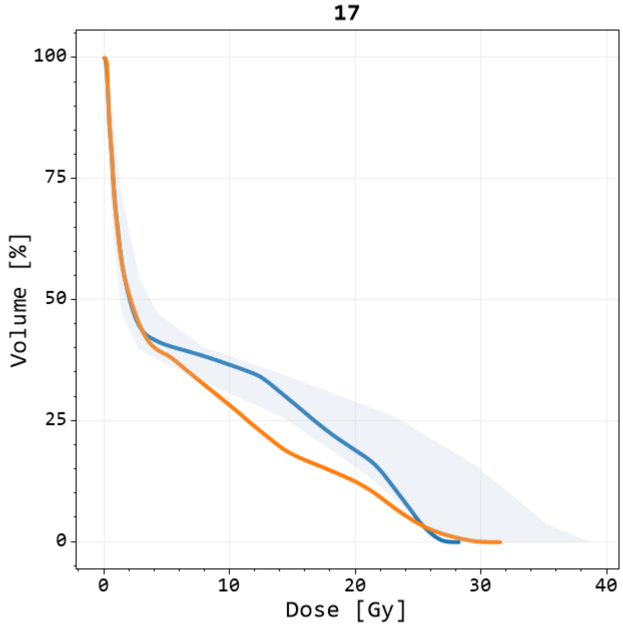
17
100
75
50
25
0
Volume [%]
0
10
20
30
40
Dose [Gy]

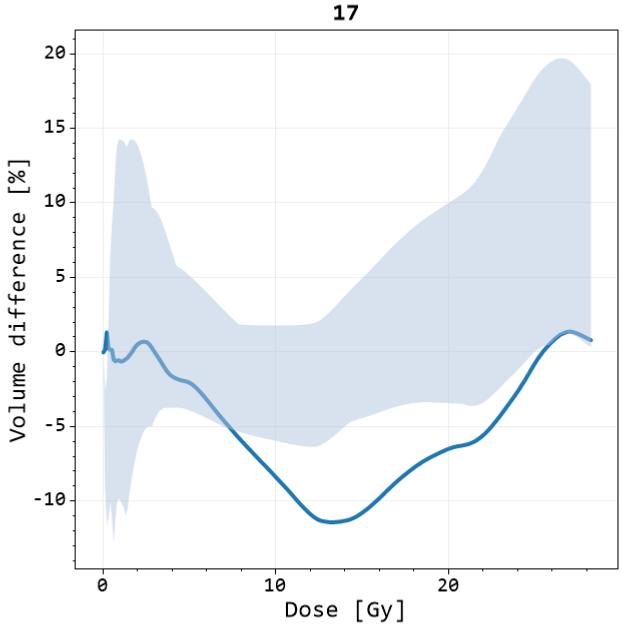
17
20
15
10
5
0
-5
-10
Volume difference [%]
0
10
20
Dose [Gy]

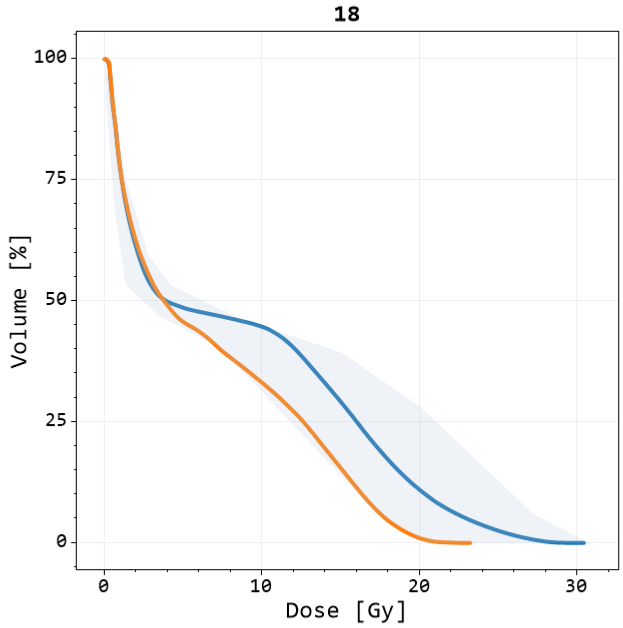
18
100
75
50
25
0
Volume [%]
0
10
20
30
Dose [Gy]

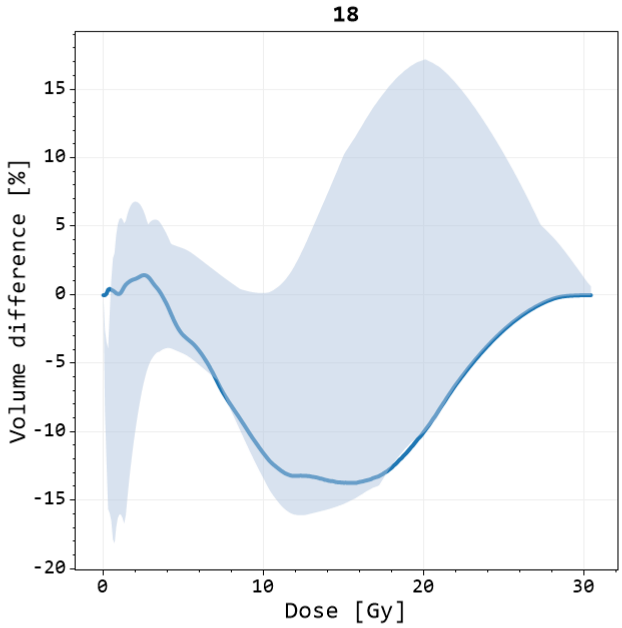
18
15
10
5
0
-5
-10
-15
-20
Volume difference [%]
0
10
20
30
Dose [Gy]

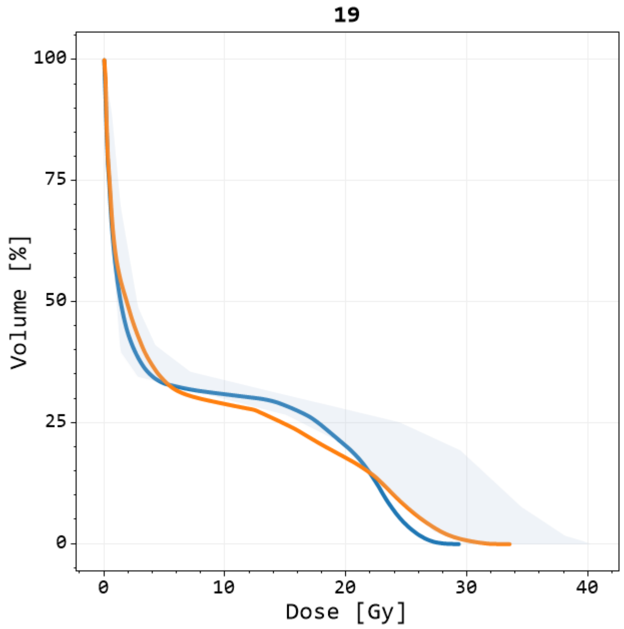
19
Volume [%]
Dose [Gy]

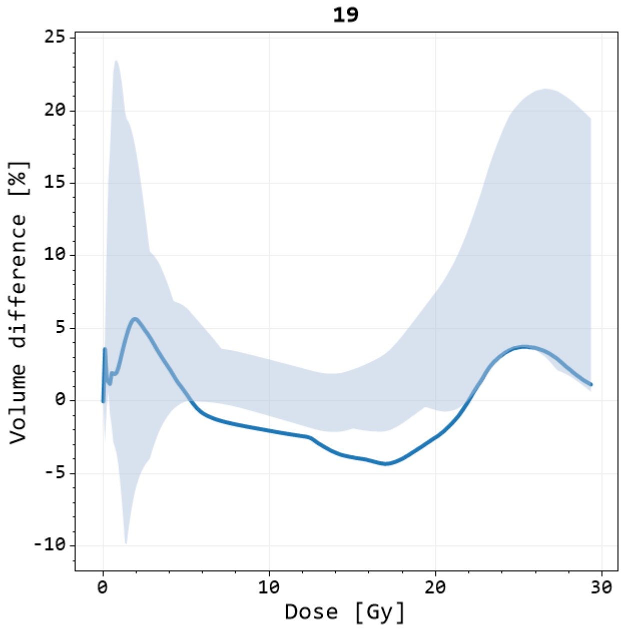
19
Volume difference [%]
Dose [Gy]

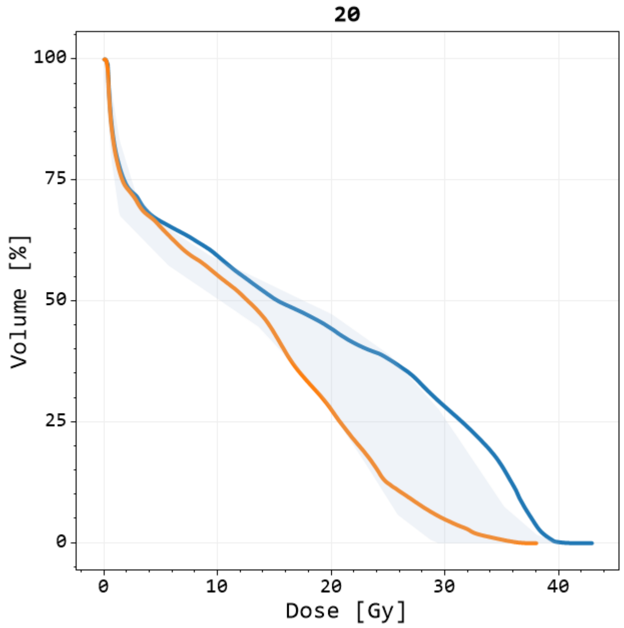
20
Volume [%]
Dose [Gy]

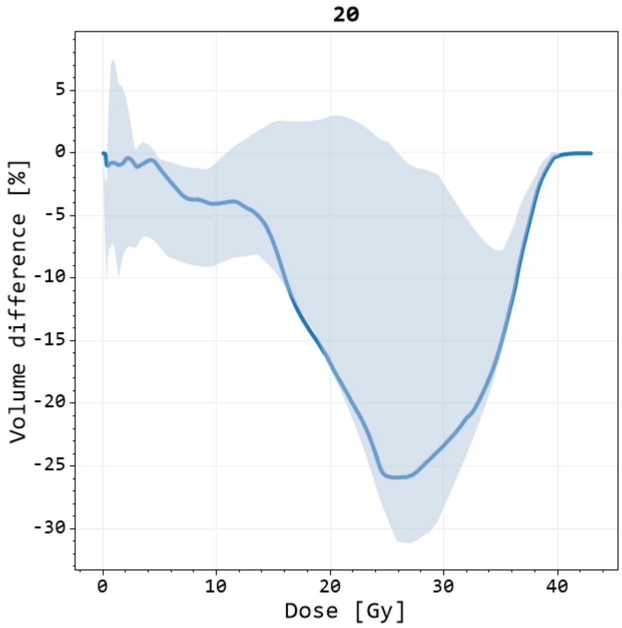
20
Volume difference [%]
Dose [Gy]

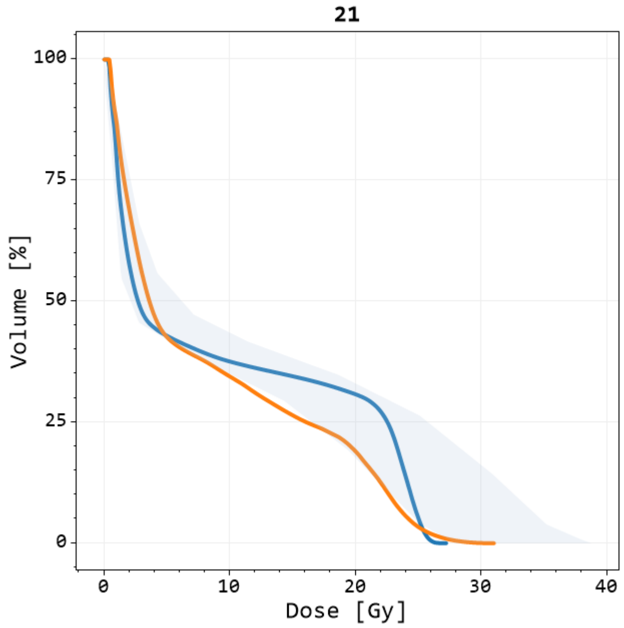
21
Volume [%]
Dose [Gy]

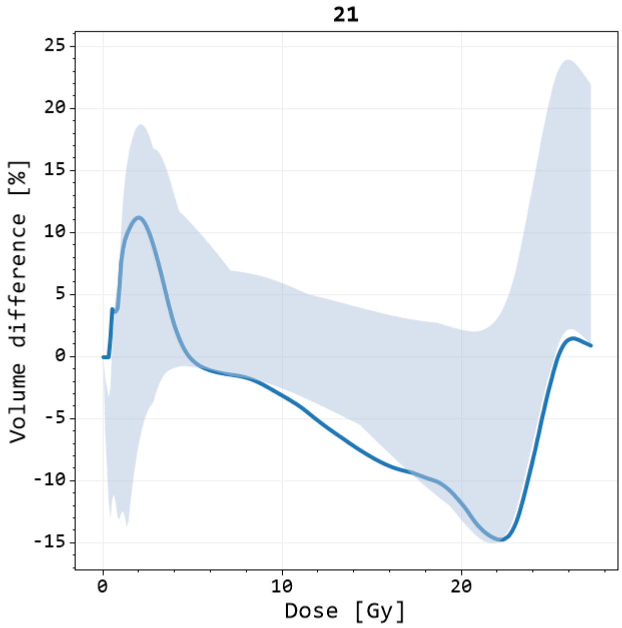
21
Volume difference [%]
Dose [Gy]

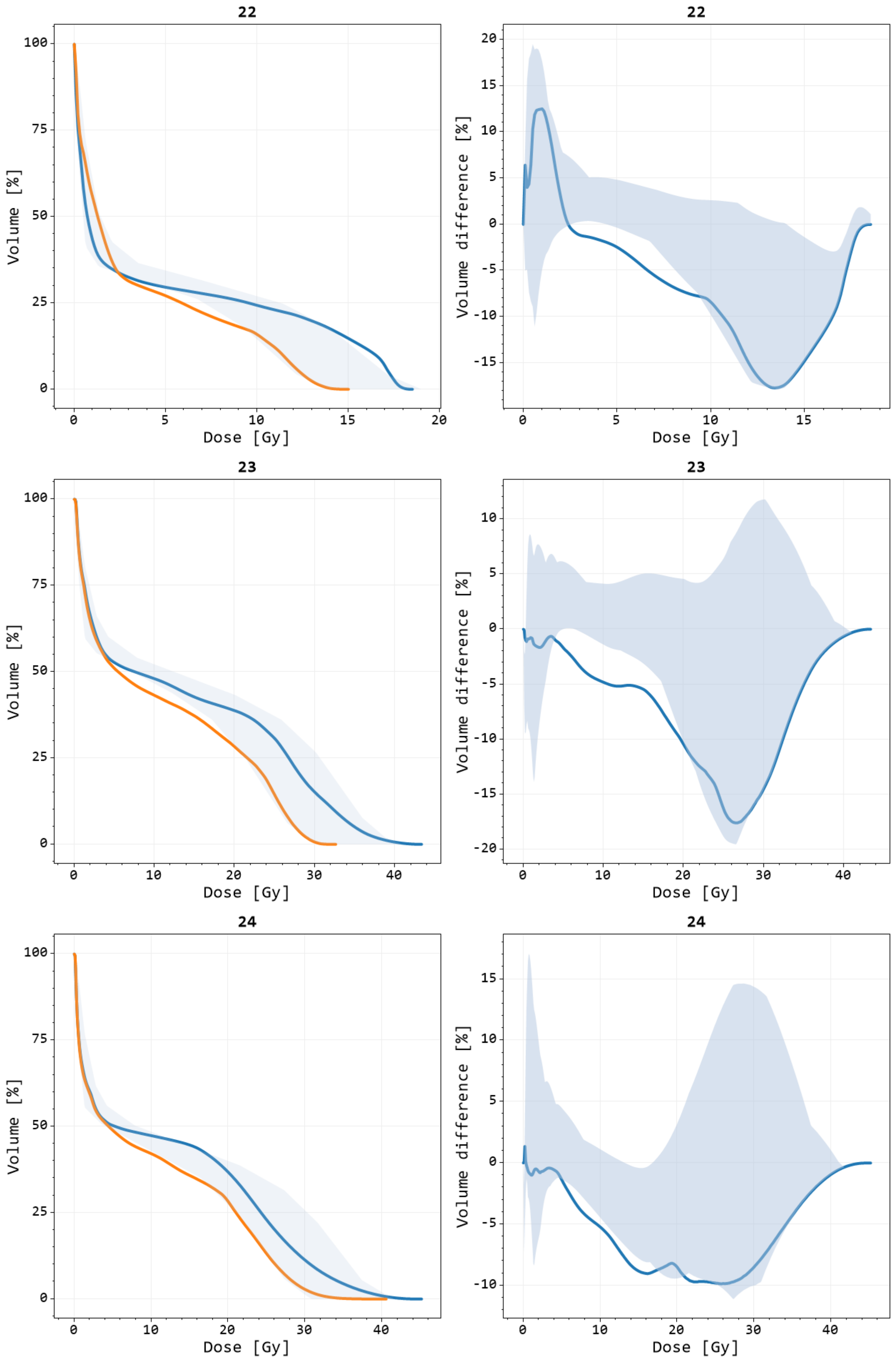
22
Volume [%]
Dose [Gy]
22
Volume difference [%]
Dose [Gy]
23
Volume [%]
Dose [Gy]
23
Volume difference [%]
Dose [Gy]
24
Volume [%]
Dose [Gy]
24
Volume difference [%]
Dose [Gy]

# DVHs

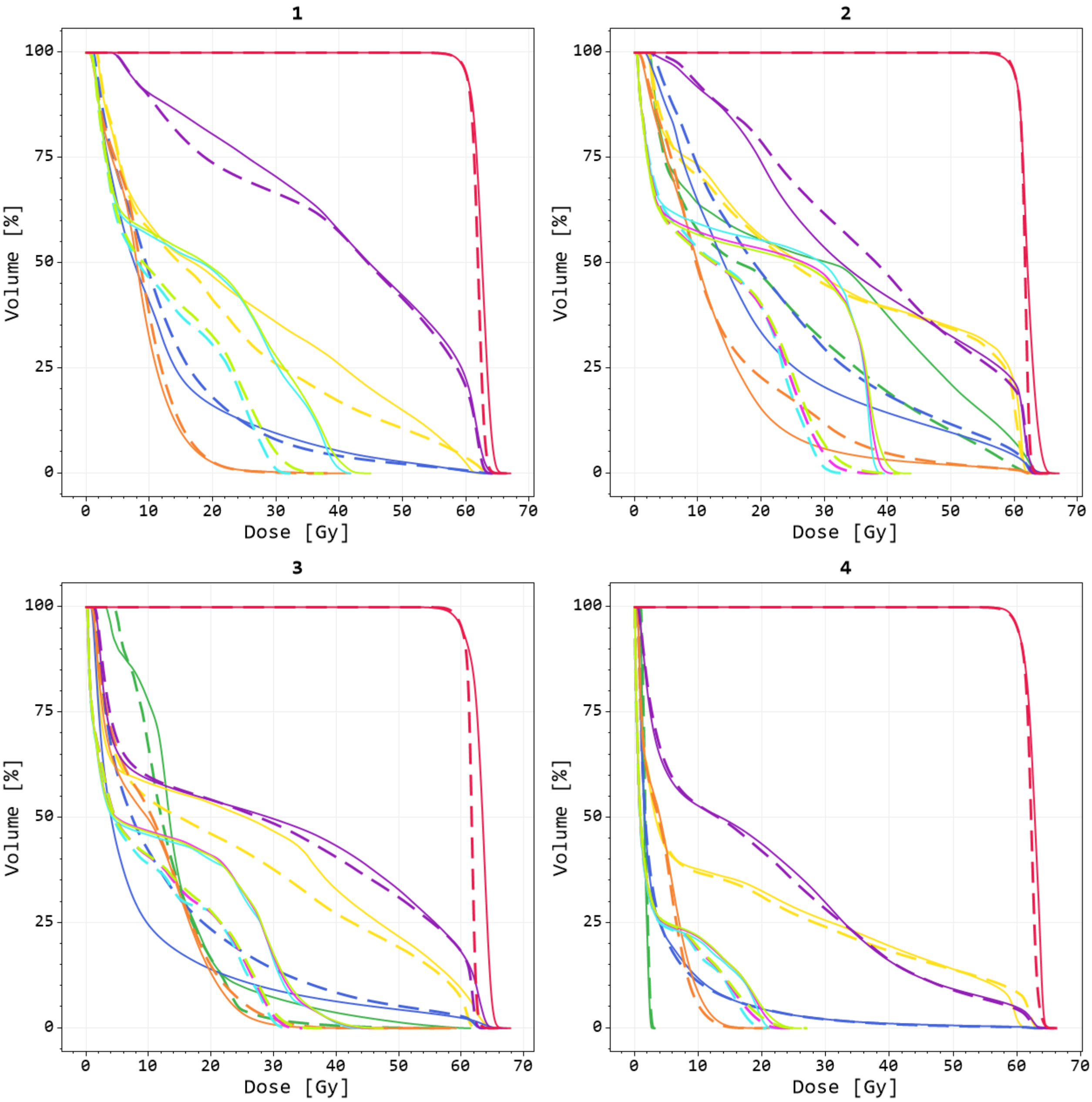

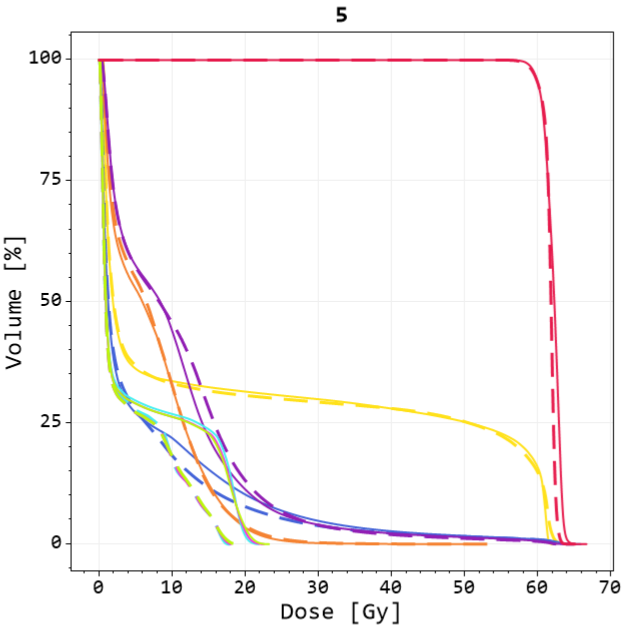
5
100
75
50
25
0
Volume [%]
0 10 20 30 40 50 60 70
Dose [Gy]

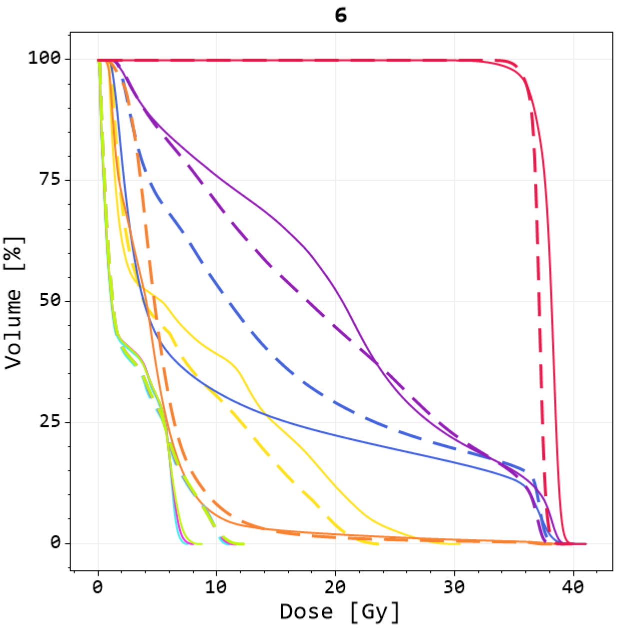
6
100
75
50
25
0
Volume [%]
0 10 20 30 40
Dose [Gy]

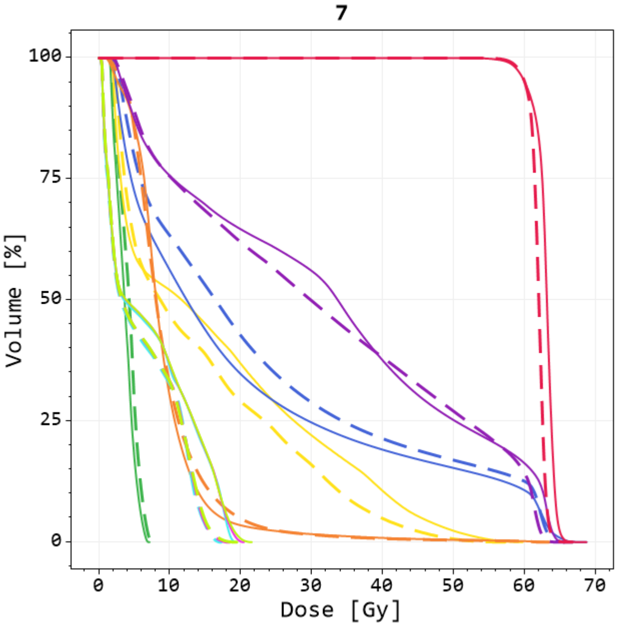
7
100
75
50
25
0
Volume [%]
0 10 20 30 40 50 60 70
Dose [Gy]

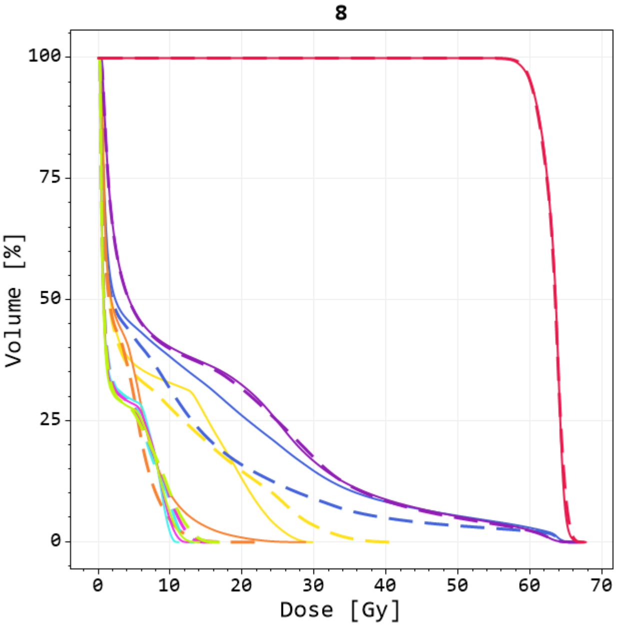
8
100
75
50
25
0
Volume [%]
0 10 20 30 40 50 60 70
Dose [Gy]

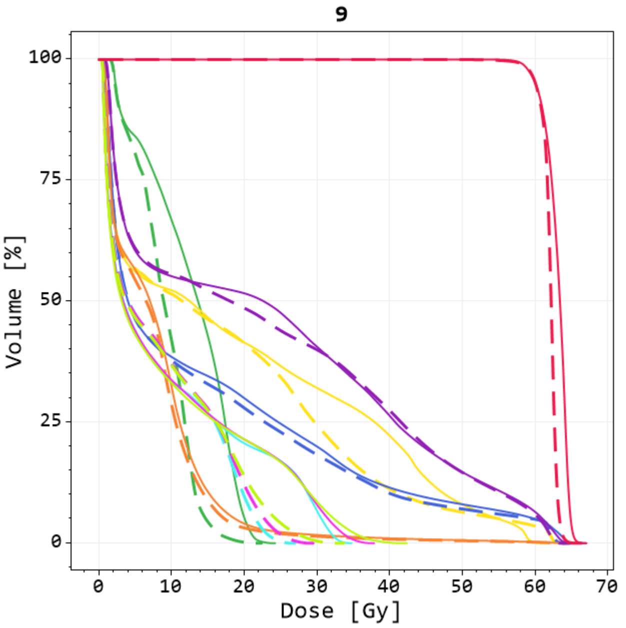
9
100
75
50
25
0
Volume [%]
0 10 20 30 40 50 60 70
Dose [Gy]

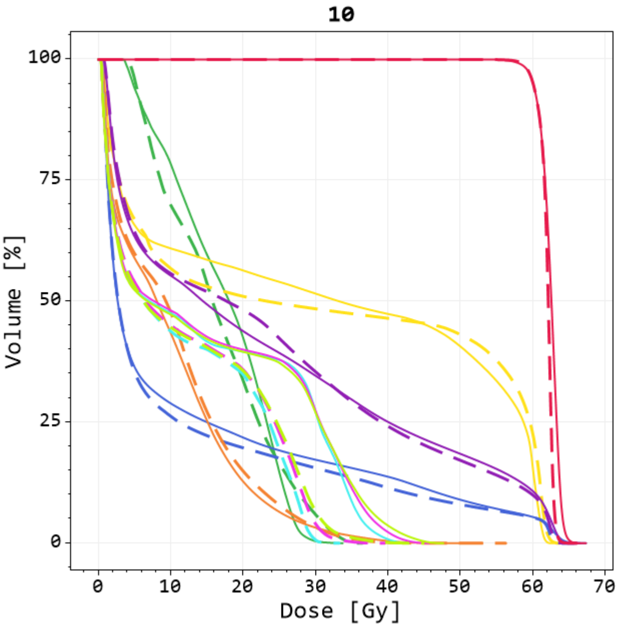
10
100
75
50
25
0
Volume [%]
0 10 20 30 40 50 60 70
Dose [Gy]

11

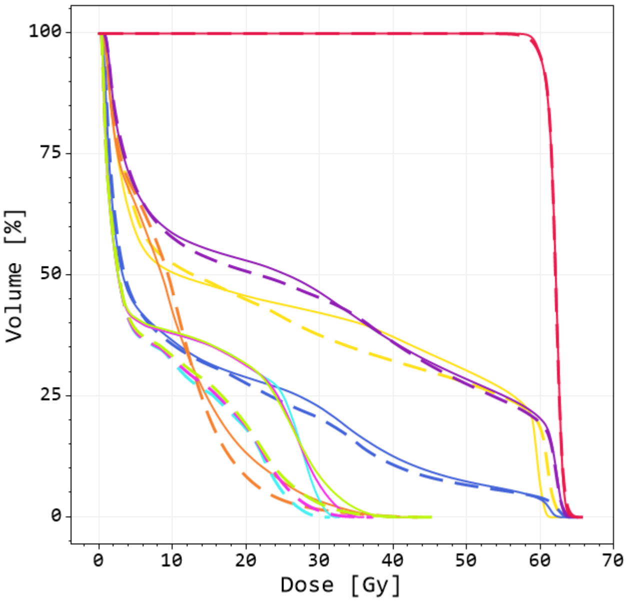


12

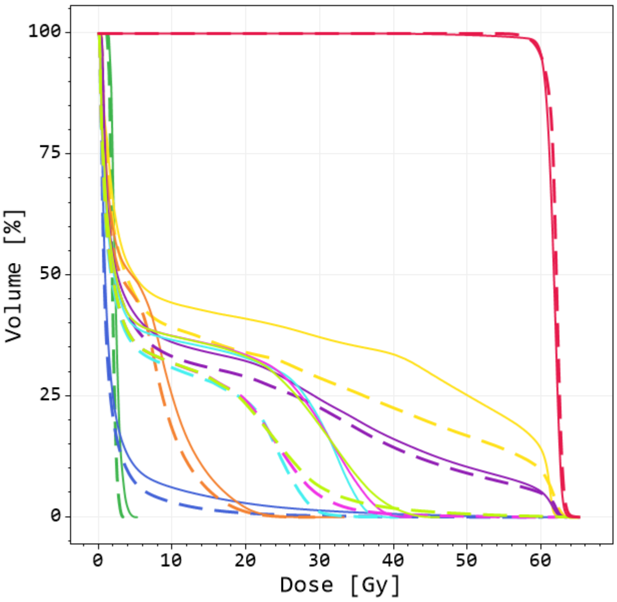


13

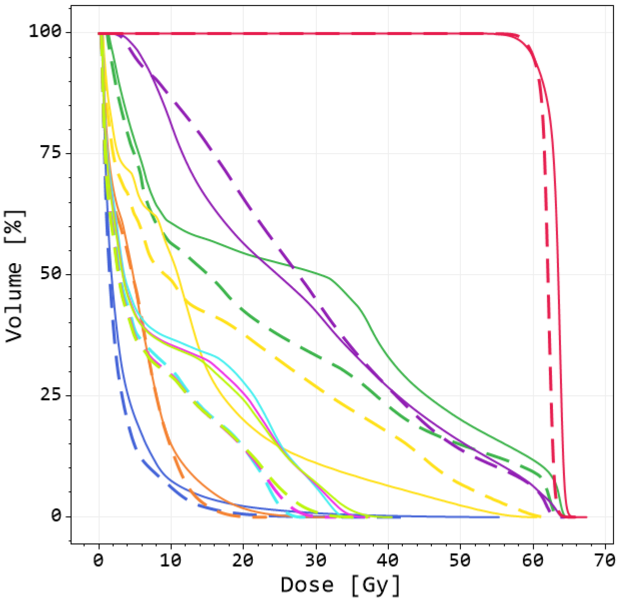


14

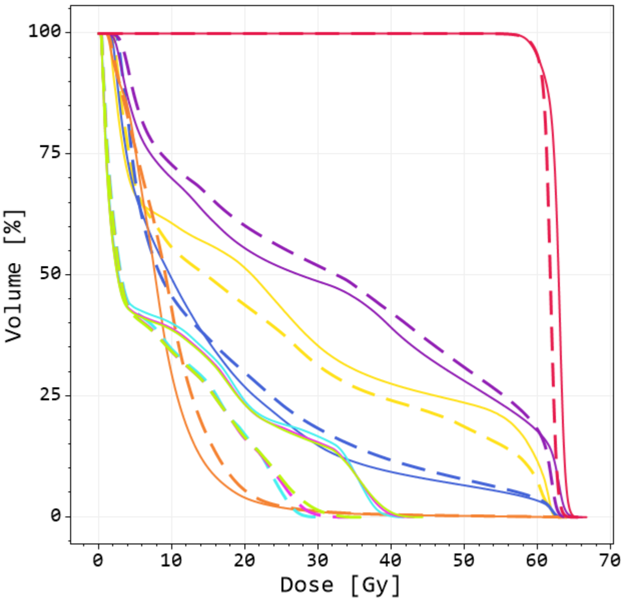


15

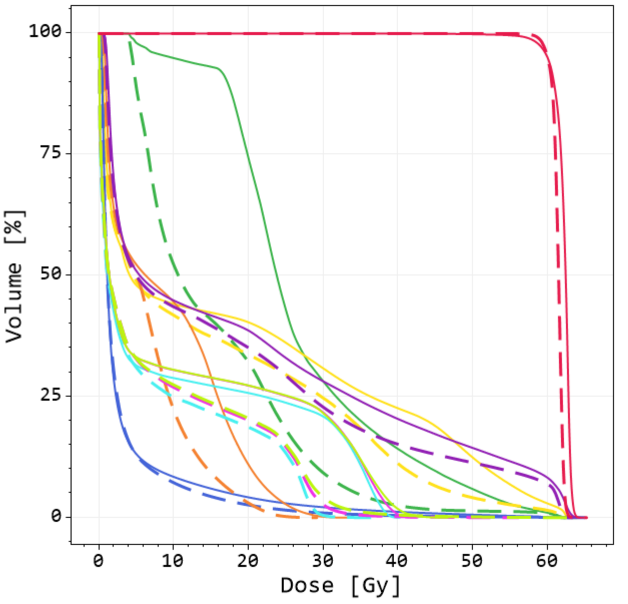


16

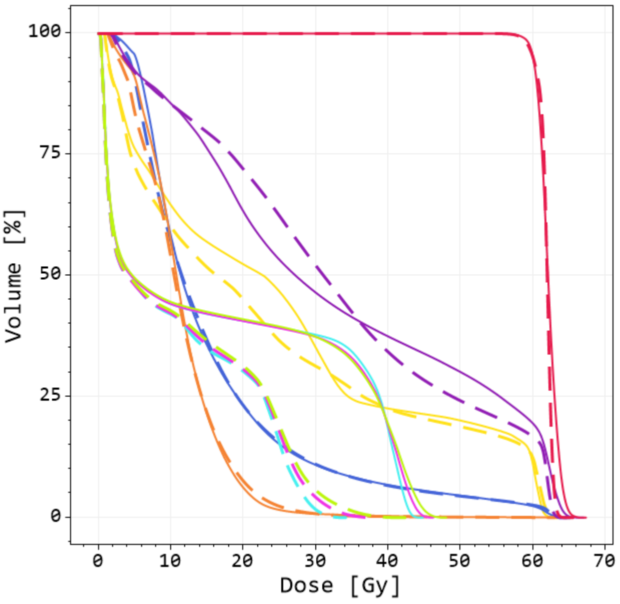

17

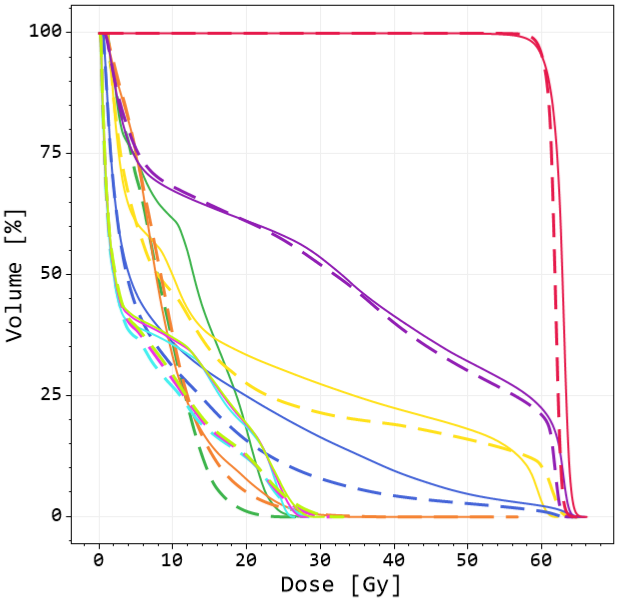


18

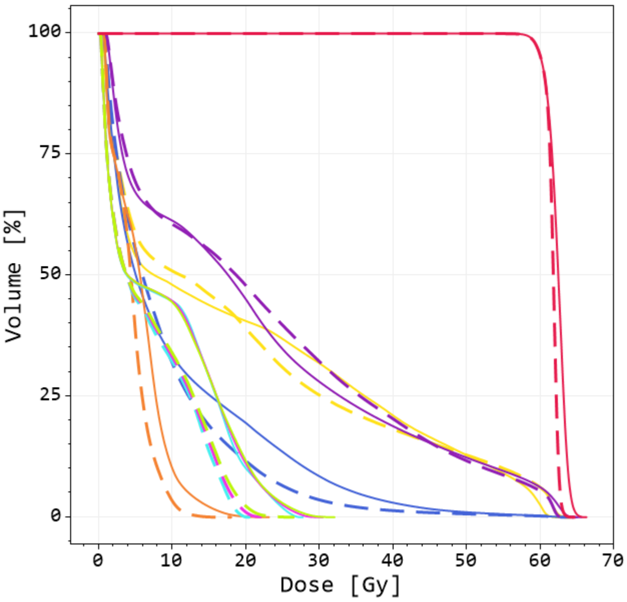


19

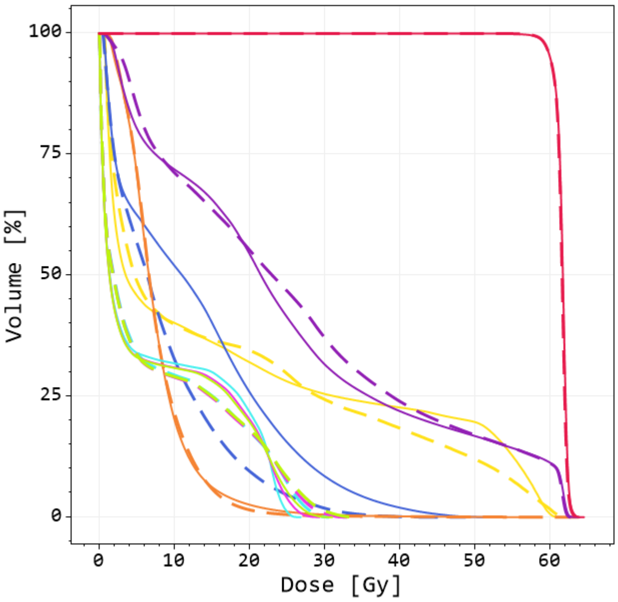


20

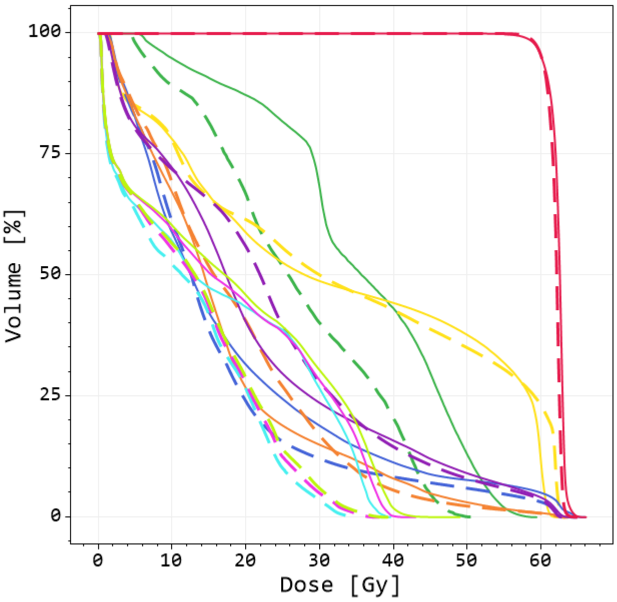


21

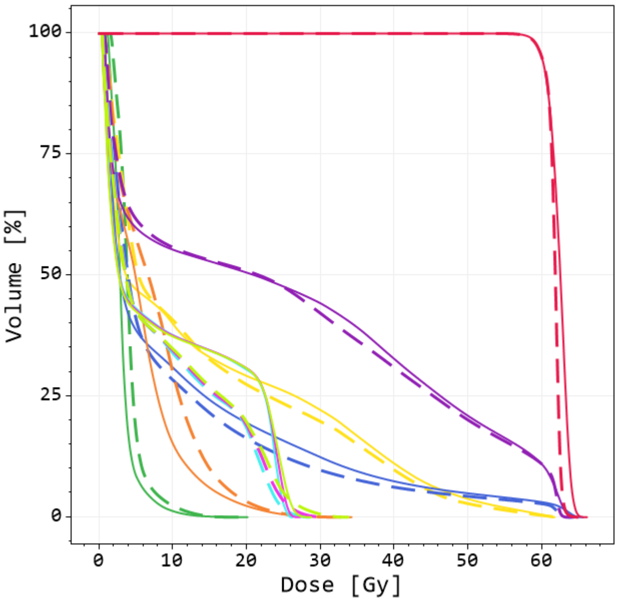


22

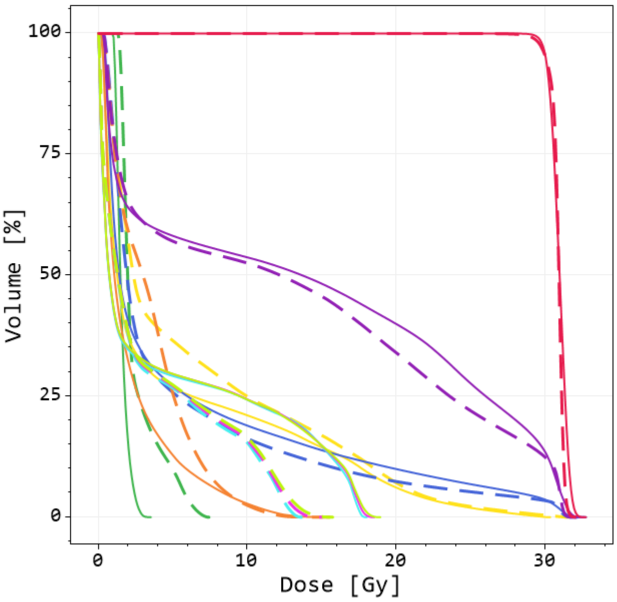

23

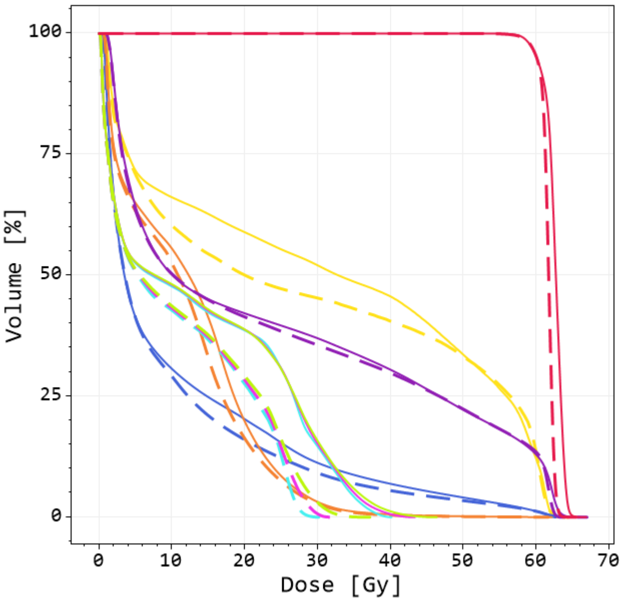
100
75
50
25
0
Volume [%]
0
10
20
30
40
50
60
70
Dose [Gy]

24

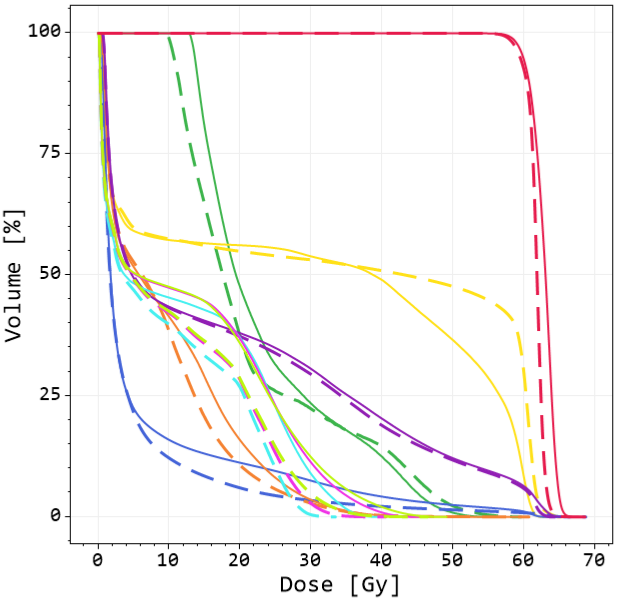
100
75
50
25
0
Volume [%]
0
10
20
30
40
50
60
70
Dose [Gy]

25

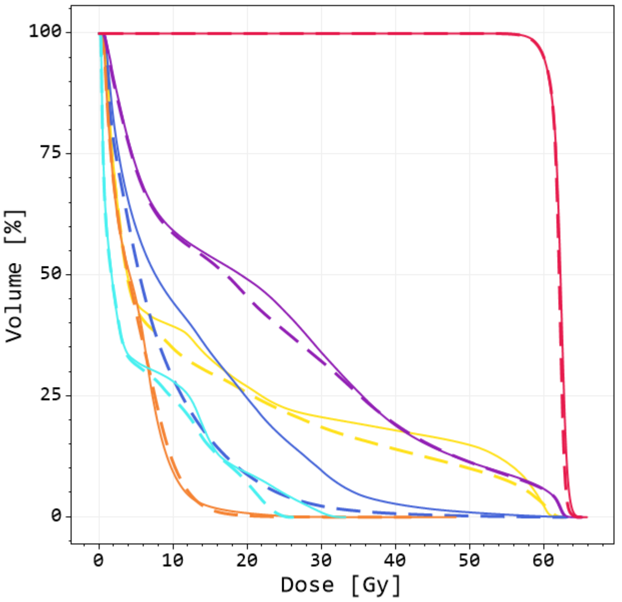
100
75
50
25
0
Volume [%]
0
10
20
30
40
50
60
Dose [Gy]
